# Anionic T‐Shaped Platinum(0) Pincer Complexes and Platinum(I) Intermediates in Radical Reactions With Alkyl Halides

**DOI:** 10.1002/anie.3980943

**Published:** 2026-06-03

**Authors:** Vincenz J. Kohler, Julia Rößling, Tim M. Diederich, Joachim Ballmann, Regine Herbst‐Irmer, Dietmar Stalke, Lutz H. Gade

**Affiliations:** ^1^ Anorganisch‐Chemisches Institut Ruprecht‐Karls‐Universität Heidelberg Heidelberg Germany; ^2^ Institut für Anorganische Chemie Georg‐August‐Universität Göttingen Göttingen Germany

**Keywords:** metalloradicals, pincer ligands, platinum, radical coupling, T‐shaped complexes

## Abstract

Two anionic T‐shaped d^10^ platinum(0) complexes supported by a sterically demanding carbazole‐based PNP ligand were obtained by reduction of a platinum(II) chloride precursor and characterized as contact ion aggregates **2‐Na** and **2‐Mg** with the Na^+^ and [MgCl(thf)_2_]^+^ counterions, respectively. Reactions with organyl bromides produced the homocoupled hydrocarbons and the platinum(II) bromide and alkyl species, depending on the stability of the generated radicals. In situ EPR spectroscopic studies allowed the detection of the transient T‐shaped 15‐electron platinum(I) metalloradical (g_iso_ = 2.219 and a hyperfine coupling (HFC) interaction with a ^195^Pt atom A_iso_ of 2124 MHz) involved in the sequential single‐electron transfer steps.

## Graphical abstract

Two anionic, T‐shaped, d^10^ platinum(0) complexes containing a PNP pincer ligand have been isolated and characterized. These complexes react with aliphatic C‐Br bonds under formation of a platinum(II) bromide and an alkyl complex, as well as the homocoupled hydrocarbons. In situ EPR experiments detected a transient, T‐shaped, 15‐electron platinum(I) metalloradical involved in the sequential single‐electron transfer reaction.

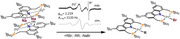

Metal(I) intermediates have been proposed in numerous chemical transformations involving group 10 metals. Low‐valent platinum complexes, in particular, play a pivotal role in hydroformylation, hydrosilylation, and hydrogenation reactions [1]. Furthermore, it has been suggested that platinum(I) is important for the anticancer effects of platinum‐based drugs [2, 3, 4]. Molecular models of these species provide valuable insight and have therefore been extensively synthesized and characterized for nickel [5, 6, 7, 8, 9, 10, 11] and, more recently, palladium [12, 13, 14, 15, 16, 17]. Notably, palladium(I) and palladium(0) complexes (Figure 1a) have been shown to activate aryl and alkyl halides, highlighting the relevance of low‐valent species for bond activation chemistry [14, 18, 19]. In contrast, only very few platinum metalloradical species have been reported to date [20, 21, 22, 23]. In particular, the controlled one‐electron oxidation of neutral linear bis(phosphine)platinum(0) precursors to isolable monocationic platinum(I) radical species has been described by Chaplin [23]. Depending on the ligand environment, platinum(0) complexes may be either neutral [24, 25, 26, 27, 28] or anionic. However, the isolation of electron‐rich anionic platinum(0) species remains intrinsically challenging due to strong Pauli repulsion between the metal center and donor ligands [13]. Milstein et al. demonstrated that such compounds can be generated via direct reduction of a platinum(II) chloride precursor using a PCP pincer ligand, providing the first isolated example along with a preliminary investigation of its reactivity [29]. More recently, Bourissou and coworkers reported anionic platinum(0) complexes stabilized by a PBP pincer ligand (Figure 1b) [30, 31]. Herein, we report the full structural characterization of anionic PNP platinum(0) pincer complexes, their cleavage of C─Br bonds via one‐electron radical pathways and the direct in situ detection by EPR of the T‐shaped 15‐electron platinum(I) metalloradical intermediate.

**FIGURE 1:**
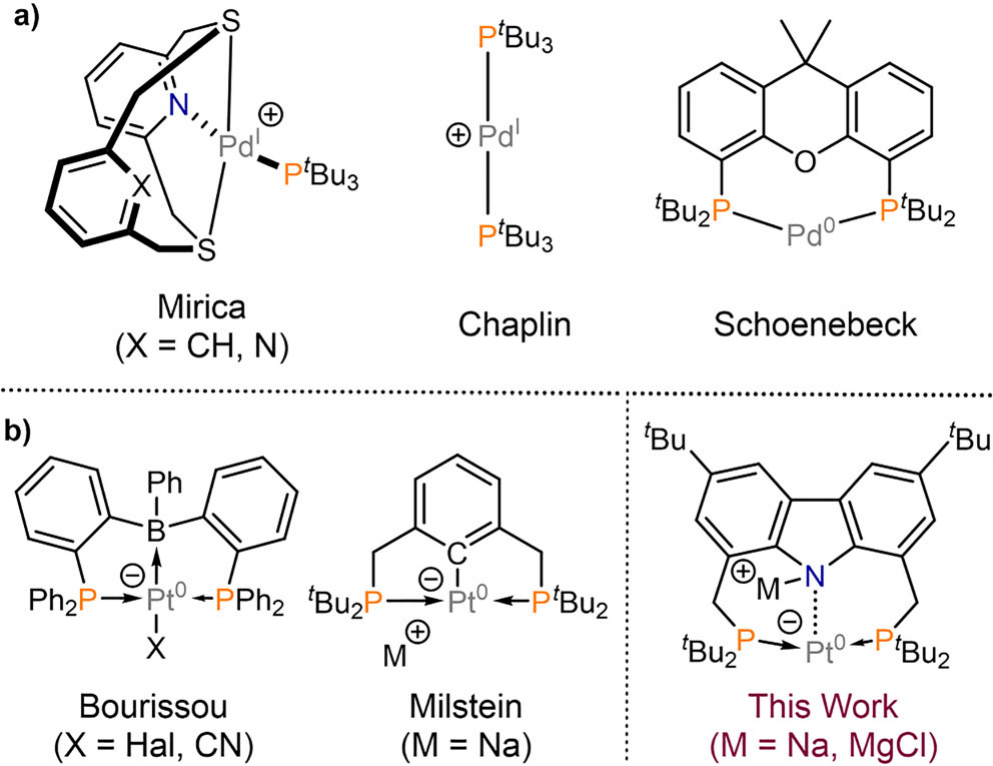
(a) Selected low‐valent palladium species capable of aryl/alkyl halide activation. (b) Anionic platinum(0) pincer complexes from literature related to this work.

Following a previously reported method [32] the chloridoplatinum(II) precursor **1‐Cl** was synthesized by deprotonation of the protioligand ^Cbz^(*^t^* ^Bu^PNP)H with lithium bis(trimethylsilyl)amide (LiHMDS) and subsequent salt metathesis with [Pt^II^Cl_2_(SEt_2_)_2_] (see Supporting Information). Reduction with an excess of NaPb alloy or a mixture of Mg and HgCl_2_ in THF led to a change of color from yellow to deep orange and the formation of the respective diamagnetic platinum(0) complexes **2‐Na** or **2‐Mg** in nearly quantitative yield (Scheme 1).

**SCHEME 1:**
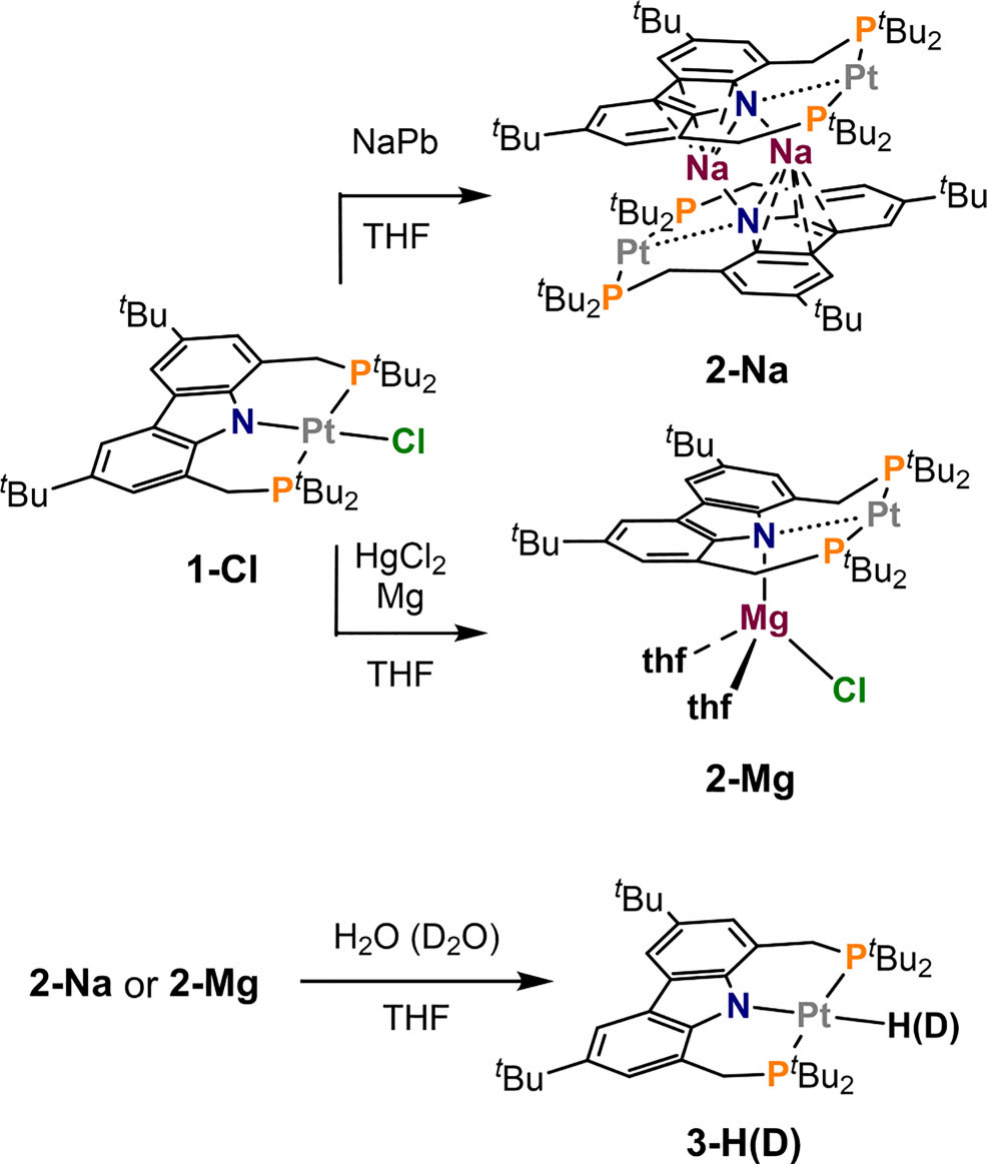
Top: Synthesis of platinum(0) complexes 2‐Na and 2‐Mg via reduction of the chlorido precursor 1‐Cl with NaPb alloy or Mg/HgCl_2_. Bottom: Controlled protonation (deuteration) of 2‐Na and 2‐Mg with H_2_O (D_2_O) at Pt, demonstrating the metal‐centered Brønsted‐basicity.

In the ^31^P NMR spectra of both compounds, singlet resonances at 93.7 ppm (**2‐Na**) and 86.2 ppm (**2‐Mg**) were observed, flanked by pairs of ^195^Pt satellites (^1^J_P‐Pt _= 4964 Hz for **2‐Na**; ^1^J_P‐Pt _= 4497 Hz for **2‐Mg)**. Their ^195^Pt NMR spectra displayed signals at −5328 ppm (**2‐Na**) and −5704 ppm (**2‐Mg**) as triplets with significantly higher coupling constants of ^1^J_Pt‐P _= 4959 Hz for **2‐Na** and ^1^J_Pt‐P _= 4487 Hz for **2‐Mg** compared to the platinum(II) complex **1‐Cl** (^1^J_Pt‐P _= 2723 Hz). This is consistent with previous observations and has been attributed to the higher s‐character of the Pt─P bonds in the zero‐valent complexes and the higher electron density at the metal [29].

The molecular structures of **2‐Na** and **2‐Mg** were established by single crystal x‐ray diffraction (Figure 2a). In both cases the platinum center is coordinated by the two phosphine side arms, while the carbazole nitrogen atoms coordinate weakly, completing the T‐shaped coordination sphere of the anionic complex unit. Since in case of **2‐Na** the entire molecule is disordered about a mirror plane, the metric parameters have to be treated with care. In case of **2‐Mg** the alkaline earth metal bound to nitrogen is coordinatively saturated by two units of THF and one chloride ion. For **2‐Na** no coordination of solvent was observed in the crystal. Instead, the nitrogen and sodium atoms of two molecular units form a square, with the sodium atoms additionally interacting with the central carbazole ring of the opposite molecule. In contrast to the dimeric arrangement observed in the solid state, **2‐Na** exists as a monomer in solution, as demonstrated by ^1^H and ^31^P DOSY (Diffusion‐Ordered NMR Spectroscopy) using **3‐H** as a diffusion standard (see Supporting Information for details), reflecting in nearly identical behavior of **2‐Na** and **2‐Mg**. As expected, the softer Na^+^ prefers the *η* ^5^‐coordination to the heteroaromatic NC_4_ perimeter while the much harder Mg^2+^ favors exclusive N‐coordination [33, 34]. The distances between Na and C/N atoms from the central ring of the carbazole unit of 2.33‐2.86 Å are within the range of known *η* ^5^‐coordination of alkali metals to carbazole. The coordination of the Na^+^ and [MgCl(thf)_2_]^+^ cations to the carbazole nitrogen atoms draw them away from the zero‐valent platinum centers, resulting in relatively large interatomic Pt─N distances of 2.71(4) Å (**2‐Na**) and 2.796(5) Å (**2‐Mg**) compared to the square planar platinum(II) precursor complexes (d_Pt‐N_ ∼ 2.0–2.2 Å) [32]. In contrast, the Pt─P bond lengths of both low‐valent complexes are slightly shortened by about 0.1 Å.

**FIGURE 2:**
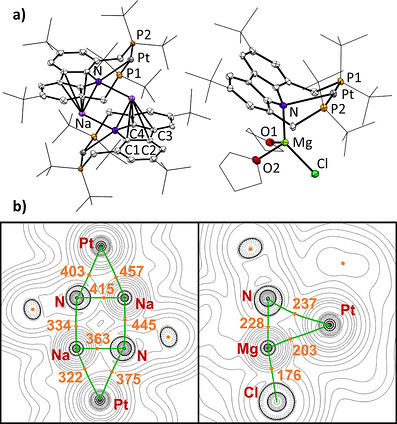
(a) Molecular structure [49] of 2‐Na (left) and 2‐Mg (right) with anisotropic displacement parameters set to 30 % probability. Hydrogen atoms and disorders are omitted for clarity. *Tert*‐butyl groups are shown in wireframe style. Selected bond lengths (Å) and angles (°) for 2‐Na: Pt‐P1 2.261(2), Pt‐P2 2.261(2), Pt‐Na 2.746(17), Pt‐N 2.71(4), Na‐N 2.33(2), N‐Pt‐P1 84.4(3), N‐Pt‐P2 84.4(3), P1‐Pt‐P2 165.4(6), P1‐Pt‐Na 90.4(2), P2‐Pt‐Na 89.74(19). Selected bond lengths (Å) and angles (°) for 2‐Mg: Pt‐P1 2.2405(14), Pt‐P2 2.2372(14), Pt‐Mg 3.851(2), Pt‐N 2.796(5), N‐Pt‐P1 87.67(13), N‐Pt‐P2 88.38(13), P1‐Pt‐P2 175.59(5), P1‐Pt‐Mg 88.02(6), P2‐Pt‐Mg 87.58(6). (b) Laplacian distribution (∇^2^ρ(r)) for 2‐Na (left) and for 2‐Mg (right) and the corresponding critical points (orange dots for BCPs) were computed by QTAIM analysis [40, 41] on optimized structures (see Supporting Information for details). Solid contour lines indicate regions with charge depletion (∇^2^ρ(r)>0) and dashed contour lines indicate regions with charge accumulation (∇^2^ρ(r)<0).

To obtain additional insight into the bonding between the ligand and metal atoms, and to clarify the nature and relevance of the unusually long Pt─N interactions observed crystallographically as well as the influence of the counterions on the electronic structure, both molecular structures were modeled by density functional theory (DFT) and optimized at the r^2^SCAN‐3c [35, 36, 37]/Def2‐mTZVP [38, 39] level of theory (see Supporting Information for details). Based on these optimized models quantum theory of atoms in molecules (QTAIM) analyses [40, 41] were performed [B3LYP‐D3(BJ) [42, 43, 44, 45] including relativistic effects at the Zero‐Order Regular Approximation (ZORA) level] [46, 47, 48]. The Laplacian distributions for **2‐Na** and **2‐Mg** are depicted below (Figure 2b) and the parameters relevant for interpreting the bond critical points (BCPs) are listed in the Supporting Information. Given the interatomic Pt‐N distances of 2.7–2.8 Å, their role in the coordination of the platinum(0) atoms was of particular interest. All Pt‐N BCPs exhibit moderate electron density values, ρ(r) ≈ 0.028‐0.029 a.u., indicating appreciable electron accumulation in the bonding region while the Laplacian of the electron density ∇^2^ρ(r) is positive for all three BCPs, reflecting local charge depletion at the bond critical point and a significant closed‐shell contribution, as commonly observed for transition metal‐ligand bonds. A small negative value of the total energy density H(r) was obtained for the Pt─N contacts, demonstrating that potential energy dominates over kinetic energy which is consistent with a weak covalent, shared‐shell character. Overall, the combination of moderate electron density, positive Laplacian and negative energy density classifies the Pt‐N interactions as polar, partially covalent metal‐ligand bonds rather than purely electrostatic contacts. This finding is significant, as it demonstrates that the carbazole nitrogen atoms remain electronically engaged with the platinum center despite the long interatomic distances, thereby supporting the description of a weakly, but meaningfully coordinated T‐shaped platinum(0) center. In contrast, Pt‐Na contacts show low ρ(r), positive ∇^2^ρ(r), and positive H(r), consistent with closed‐shell, electrostatic interactions, while an intermediate ρ(r) and a small negative H(r) value were obtained for the Pt‐Mg interaction in **2‐Mg**, suggesting a weak covalent (donor‐acceptor) contribution to the metal‐metal contact in that case.

The low‐valent platinum compounds proved to be very sensitive to moisture. The formation of the hydride complex **3‐H** occurs rapidly (Scheme 1, bottom, and Supporting Information) and was observed as a minor byproduct of around 2% during the initial reduction. Controlled stoichiometric protonation (deuteration) of **2‐Na** and **2‐Mg** with H_2_O (D_2_O) at platinum demonstrated metal‐centred Brønsted‐basicity. Alternatively, it was possible to obtain the hydride complex by reaction of the protioligand ^Cbz^(*^t^* ^Bu^PNP)H with Pt^0^(P*^t^* Bu_3_)_2_ as precursor.

The PNP ligand system employed in this study has been shown to stabilize the T‐shaped metal(I) complexes of the lighter group 10 homologs nickel and palladium [16, 50]. The potential role that platinum(I) species might play in single electron redox steps competing with the two‐electron reactions such as the oxidative addition of alkyl halides to platinum(0) was thus of special interest. Platinum(I) intermediates were postulated early on in studies of the oxidative addition of organohalides to platinum(0) complexes via radical mechanisms which are in competition with the conventional platinum(0/II) closed shell pathways depending of the substrates involved [51, 52]. Such a metalloradical intermediate was also postulated for the conversion of benzyl bromide to bis(benzyl) in the reactions of Milstein's PCP platinum(0) compound [29]. We therefore initially investigated the reaction of **2‐Na** and **2‐Mg** with alkyl bromides **S1‐S4**, the corresponding radicals of which are stabilized in benzylic positions, favoring single‐electron chain mechanisms. Complete conversion of the platinum(0) species to [(PNP)Pt^II^Br] (**1‐Br**) and the selective formation of the coupling product R‐R was observed in all cases (Scheme 2).

**SCHEME 2:**
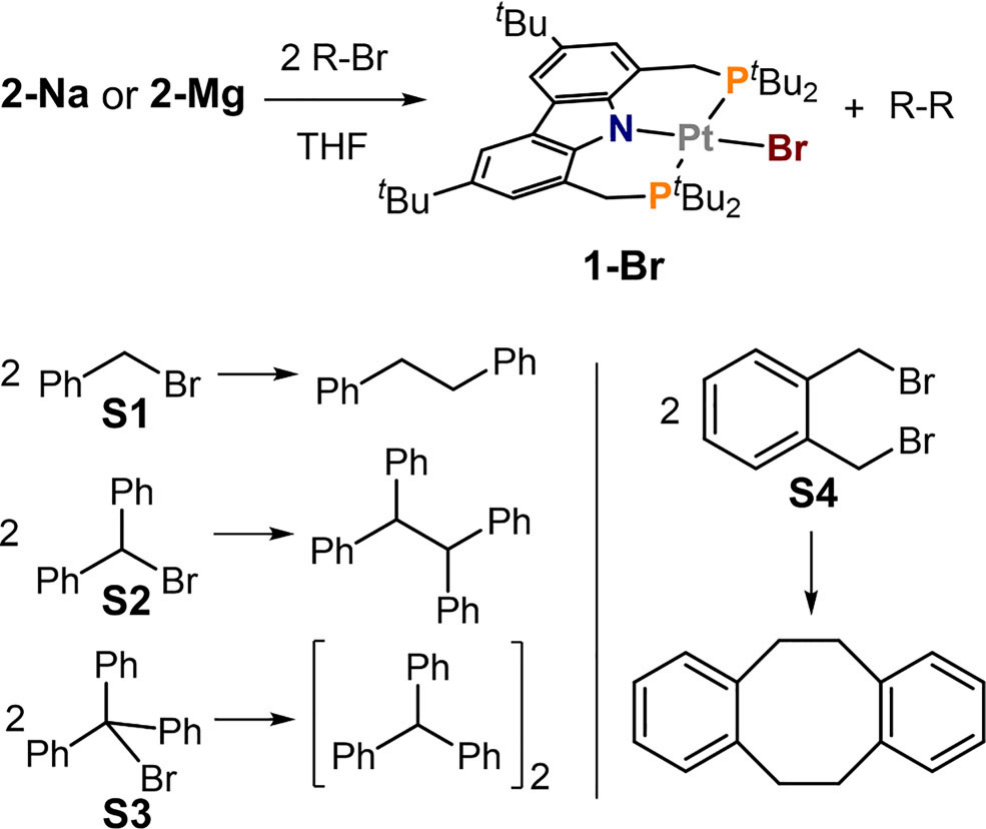
Reaction of 2‐Na or 2‐Mg with various organyl bromides yielding 1‐Br and the coupling product (R‐R).

For the most sterically demanding trityl bromide **S3**, Gomberg's dimer was formed in direct equilibrium with the trityl radical, which was detected via EPR and ultimately yielded the peroxide by oxidation in the presence of oxygen. In addition, substrate **S4** demonstrated that the presence of an additional *ortho*‐bromomethyl group led to two‐fold radical coupling to form an eight‐membered ring. If two different substrates were used in equivalent amounts in a cross‐over experiment, a reaction involving free radicals would give rise to a statistical 1:2:1 mixture of coupling products, provided the radicals possess similar structures and life times. In this case, the radical generated from diphenylbromomethane (**S2**) is slightly more stabilized by the additional phenyl group than the benzyl radical, leading to a minor increase in tetraphenylethane and decrease in diphenylethane. To probe this, 1:1 mixtures of substrates **S1** and **S2** were reacted as described above. The average result from eight runs was a distribution of 15 ± 1% diphenylethane, 59 ± 2% triphenylethane and 26 ± 1% tetraphenylethane (Scheme 3), which is consistent with a mechanism involving single‐electron transfer steps and free radicals.

**SCHEME 3:**
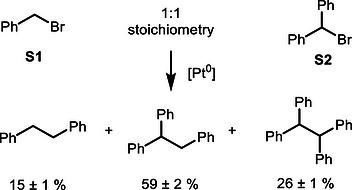
Crossover experiment between two different organyl bromides (**S1**; **S2**) and yielded coupling products.

In an in situ EPR experiment, we succeeded in detecting the postulated platinum(I) species along the free benzyl radical. For this purpose, a frozen solution of **2‐Mg** was layered with a solution of **S1** and frozen directly before mixing of the two phases and thus a reaction could take place. By slowly thawing the sample in the spectrometer, we were able to observe the processes at the interface between both phases. The X‐band EPR spectrum of the T‐shaped platinum(I) compound 2* (Figure 3a) displayed an isotropic signal with g_iso_ = 2.219 and a hyperfine coupling (HFC) interaction with a ^195^Pt atom (*I* = 1/2, A_iso_ = 2124 MHz), which is in the range of the only HFC reported for a platinum(I) species in the literature (A_iso_ = 1864 MHz) [22]. The EPR‐spectrum was computationally modeled by DFT [B3LYP‐D3(BJ) [42, 43, 44, 45] including relativistic effects at the zero‐order regular approximation (ZORA) level of theory] [46, 47, 48]. The computed isotropic g‐value of 2.298 for the 15‐electron T‐shaped platinum(I) complex corresponds well to the experimental data, while the HFC constant is slightly overestimated at this level of theory (A_iso_ = 2909 MHz). Modeling a T‐shaped platinum(I) radical based on ab initio ligand field theory (AILFT) [53] at the same computational level (Figure 3c) showed the same energetic sequence of d‐orbitals as for palladium, with the d_x_^2^_‐y_^2^ orbital as the singly occupied molecular orbital (SOMO). Compared with the recently published palladium(I) analogues [16], a Mulliken population analysis revealed that the spin density is even more localized at the metal center (approximately 72% at Pt compared to 64% at Pd, Figure 3b). Along with the resonance of the metalloradical, a sharp signal with a g‐value of 2.004 was observed in the recorded EPR spectra, consistent with a carbon‐centered organic radical. Due to the isotropic nature of the solution‐phase spectra, HFCs are not resolved, precluding an unambiguous spectroscopic identification. However, considering the reaction conditions and the absence of plausible alternative radical species, this signal is assigned to a benzyl radical [54].

**FIGURE 3:**
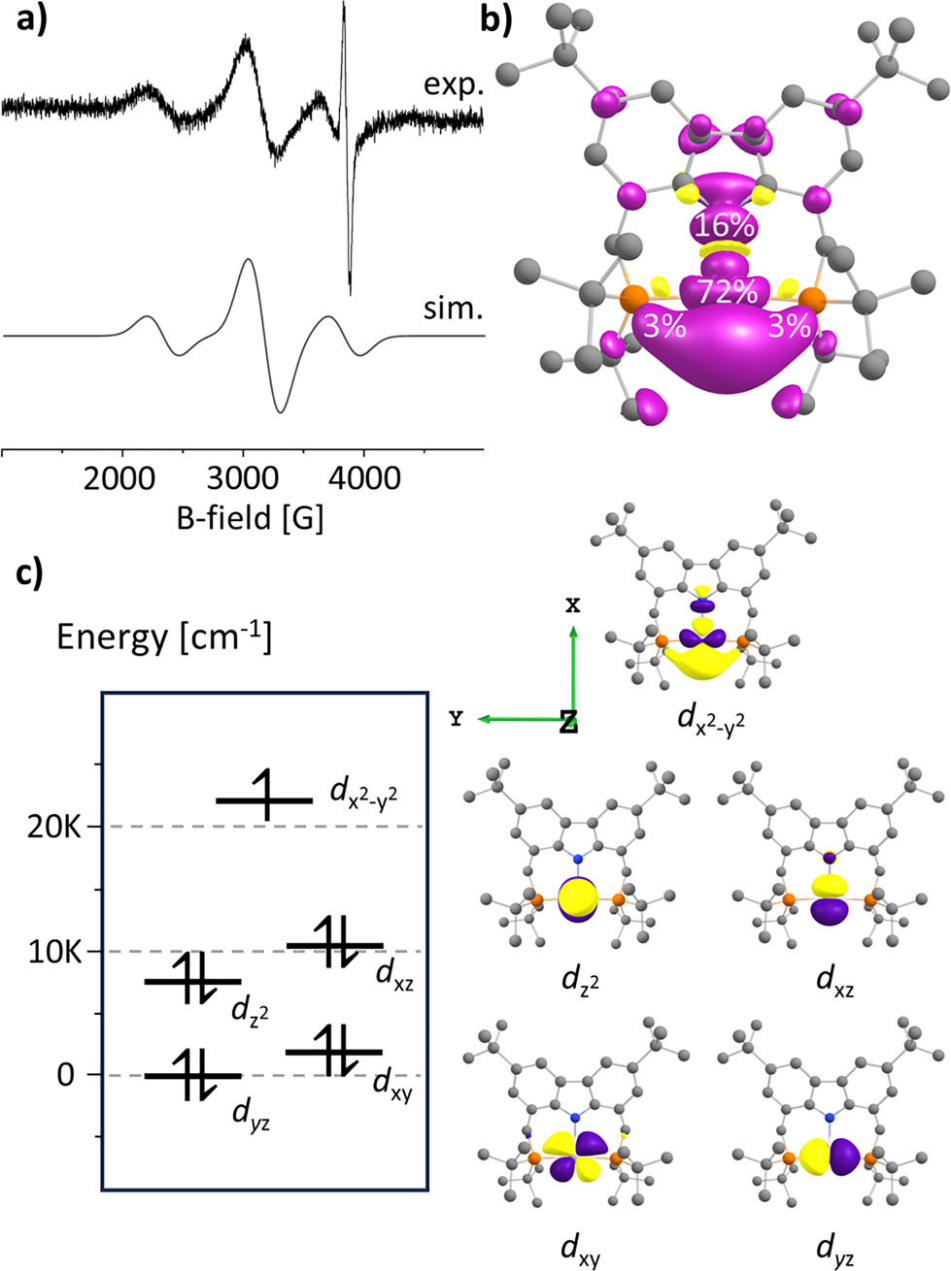
(a) X‐band EPR spectrum in THF at ∼170 K (top) with simulated EPR parameters (bottom) for the T‐shaped platinum(I) intermediate 2*. (b) Spin density (contour value = 0.001) and Mulliken spin densities [B3LYP‐D3(BJ)/SARC‐ZORA‐TZVP [55] (Pt), ZORA‐Def2‐TZVP [38] (C, N, P)]. (c) AILFT [53] computed d‐orbital energy levels and corresponding natural orbitals within the active space (derived from a SC‐NEVPT2 [56, 57, 58] corrected CAS(9,5) [59, 60], isovalue for printing = 0.03).

For non‐benzylic alkyl bromides a different stoichiometry of the coupling reactions was observed. In addition to the bromido complex **1‐Br**, the corresponding platinum(II) organyl complex **4‐R** was also formed along with a reduced yield of the R‐R coupling product (Figure 4, left, see Supporting Information for details). To obtain additional evidence for the assumed radical mechanism additional substrates, that act as radical clocks, were examined. The cyclopropylmethyl radical formed from **S5** readily underwent rapid rearrangement through ring opening, relieving the ring strain, to give the platinum(II) complex **4a**. On the other hand, the entropically and enthalpically driven cyclization of the 5‐hexenyl radical, obtained from **S6**, was also observed in compound **4b** (Figure 4, middle). The molecular structures for **4a** and **4b**, and thus the rearrangement of the radical clocks was confirmed by x‐ray diffraction (Figure 4, right).

**FIGURE 4:**
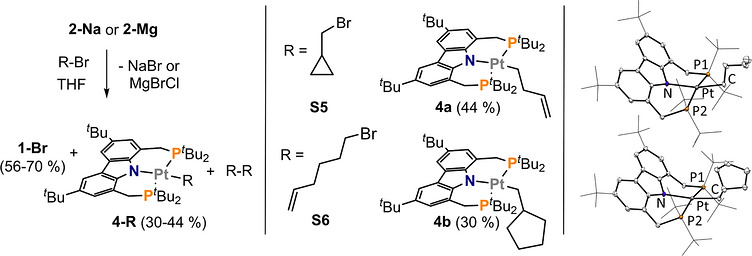
Left: Reaction of 2‐Na or 2‐Mg with various organyl bromides yielding mixtures of 1‐Br, 4‐R and R‐R (see Supporting Information). Middle: Reaction of 2‐Na or 2‐Mg with various organyl bromides acting as radical clocks. Right: Molecular structures [49] of the alkyl complexes 4a (top) and 4b (bottom) with anisotropic displacement parameters set to 30 % probability. Hydrogen atoms and disorders are omitted for clarity. *Tert*‐butyl groups are shown in wireframe style. Selected bond lengths and angles are given in the Supporting Information.

The findings discussed above are consistent with a reaction pathway involving single‐electron steps: Pt^0^→Pt^I^→Pt^II^ and the involvement of free organic radicals (Scheme 4) [29]. In that context, complexes **2‐Na** or **2‐Mg** first reduce one equivalent of RBr to a radical anion, producing a neutral platinum(I) compound 2* as an intermediate. In a subsequent step, this platinum(I) species reduces a second equivalent of RBr and the resulting platinum(II) complex **2^+^** coordinates a bromide to give **1‐Br**. The produced alkylbromide radical anions then decay to organyl radicals and bromide ions. The free radicals either recombine to form the coupling product or form the corresponding (alkyl)platinum(II) complexes **4‐R** with the platinum(I) species. Additional support for this mechanism was obtained from cyclic voltammetry (CV) measurements of **2‐Na** and **2‐Mg** (see Supporting Information for details). For **2‐Na**, a quasi‐reversible oxidation corresponding to the Pt^0^/Pt^I^ couple was observed at E_1/2_ = −0.16 V, while for **2‐Mg** a similar event occurs at E_1/2_ = −0.26 V. These values are in good agreement with those reported by Chaplin for related platinum(0) complexes [20].

**SCHEME 4:**
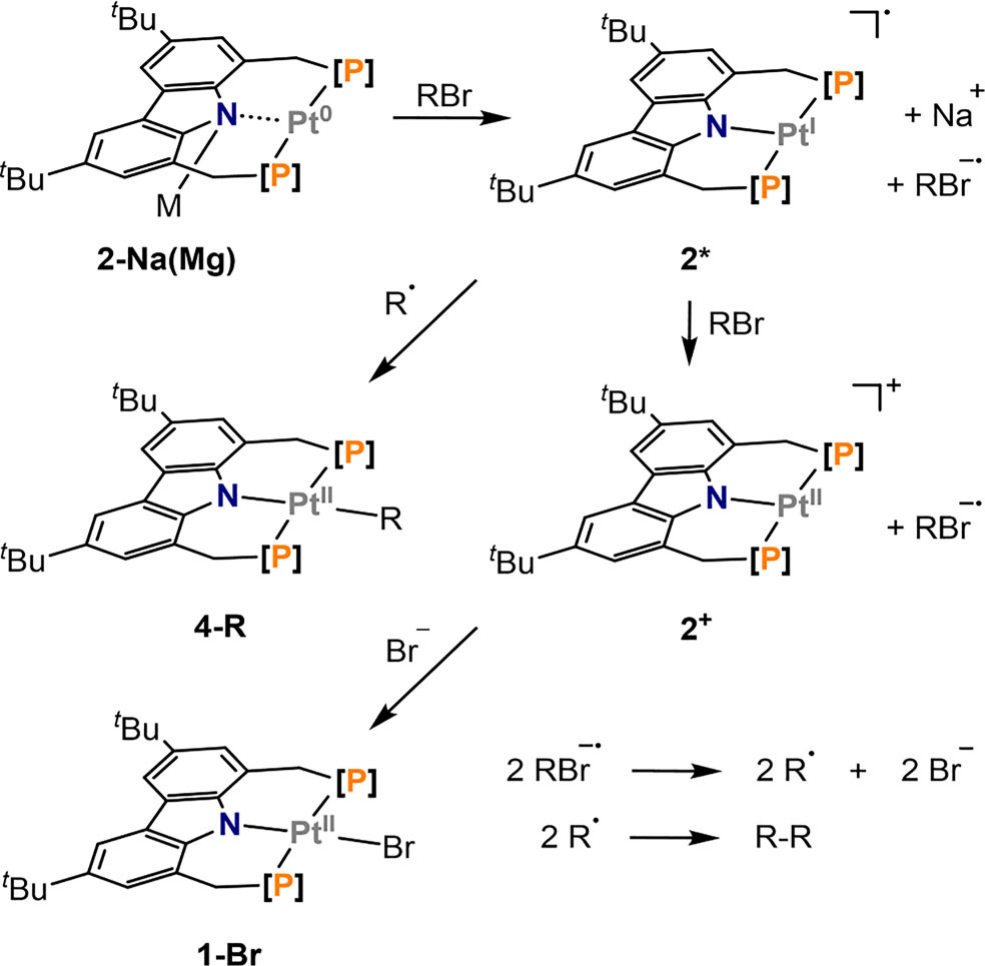
Proposed mechanism for reaction of 2‐Na or 2‐Mg with organyl bromides.

To rule out the possible involvement of platinum(IV), the previously isolated alkyl complexes **4‐R** were reacted with an excess of the corresponding organyl bromides. No reaction was observed even at temperatures up to 80 °C. Only for the reaction of **2‐Na** and **2‐Mg** with H_3_C‐Br a selective conversion to the alkyl complex **4‐CH_3_** as only product was observed, and no traces of **1‐Br** or ethane (as the expected C─C coupling product) were detected. In this case, the anionic platinum(0) complex reacts as a metal nucleophile, being transformed to the square‐planar [(PNP)Pt(CH_3_)] product, a reaction behavior which is the common alternative for zero‐valent platinum complexes [1].

In conclusion, the synthesis and full characterization of two complexes containing T‐shaped anionic PNP platinum(0) pincer units has allowed the targeted in situ EPR detection of a key neutral platinum(I) intermediate in the radical homocoupling of organyl bromides via single‐electron transfer steps. Further investigations into the reactivity of our platinum(0) species and their application in catalytic reactions are currently underway.

## Author Contributions

**Vincenz J. Kohler**: writing – original draft, investigation, methodology, data curation, formal analysis, validation, and visualization. **Julia Rößling**: investigation. **Tim M. Diederich**: visualization, and formal analysis. **Joachim Ballmann**: investigation. **Regine Herbst‐irmer**: investigation, data curation, and formal analysis. **Dietmar Stalke**: investigation, data curation, and formal analysis. **Lutz H. Gade**: writing – review and editing, writing – original draft, resources, project administration, supervision, conceptualization, and methodology.

## Conflicts of Interest

The authors declare no conflicts of interest.

## Supporting information




**Supporting File 1**: The authors have cited additional references within the Supporting Information [61, 62, 63, 64, 65, 66, 67, 68, 69, 70, 71, 72, 73, 74, 75, 76, 77, 78, 79, 80, 81, 82, 83, 84, 85, 86, 87, 88, 89, 90, 91, 92, 93, 94, 95, 96, 97, 98, 99, 100]. The data that support the findings of this study are available in the supplementary material of this article.

**Supporting File 2**: anie73022‐sup‐0002‐cif.zip.

## Data Availability

The data that supports the findings of this study are available in the supplementary material of this article
