# Supplementary material for: Anionic T‐Shaped Platinum(0) Pincer Complexes and Platinum(I) Intermediates in Radical Reactions With Alkyl Halides
Source: Angew Chem Int Ed Engl. 2026 Jun 3;65(30):e3980943. doi: 10.1002/anie.3980943 (PMC13383245; doi:10.1002/anie.3980943)
Supplement: Supplementary file 1 — Supporting File 1: The authors have cited additional references within the Supporting Information [61, 62, 63, 64, 65, 66, 67, 68, 69, 70, 71, 72, 73, 74, 75, 76, 77, 78, 79, 80, 81, 82, 83, 84, 85, 86, 87, 88, 89, 90, 91, 92, 93, 94, 95, 96, 97, 98, 99, 100]. The data that support the findings of this study are available in the supplementary material of this article. [file ANIE-65-e3980943-s002.pdf]

Supporting Information

Anionic T-shaped Platinum(0) Pincer Complexes and Platinum(I) Intermediates in Radical Reactions with Alkyl Halides

Vincenz J. Kohler[a], Julia Rößling[a], Tim M. Diederich[a], Joachim Ballmann[a†], Regine Herbst-Irmer[b], Dietmar Stalke[b], and Lutz H. Gade[a*]

a) Anorganisch-Chemisches Institut, Universität Heidelberg, Im Neuenheimer Feld 276, 69120 Heidelberg, Germany.
b) Institut für Anorganische Chemie, Georg-August-Universität Göttingen, Tammannstraße 4, 37077 Göttingen, Germany.

Table of Contents

1.) Experimental Procedures 3

i. General remarks 3

ii. Synthesis and characterization data for precursor and Platinum(0) complexes 4

iii. Synthesis of bromide and organyl complexes and characterization data 7

iv. Radical coupling products of organyl bromides and characterization data 10

v. Crossover experiments 12

2.) NMR spectra 13

3.) Variable Temperature NMR and DOSY experiments 44

4.) EPR spectra 49

5.) IR spectra 51

6.) X-ray data 57

7.) Cyclic voltammetry 64

8.) Computational Details 70

i. Geometry optimizations of complexes 71

ii. QTAIM 83

iii. CAS-SCF 84

9.) References 85

1.) Experimental Procedures

i. General remarks

All manipulations were performed under dry and oxygen-free argon using standard Schlenk techniques or within a glovebox (MBRAUN UNIlab) unless otherwise specified, using oven-dried glassware. Isolation of radical coupling products, either by flash column chromatography or vacuum distillation, was performed under non-inert conditions. All solvents were dried and distilled prior to use and degassed by three freeze-pump-thaw cycles. C_6D_6, tetrahydrofuran-d_8 (thf-d_8) and toluene-d_8 (tol-d_8) were dried over potassium. Anhydrous diethyl ether (Et_2O), *n*-pentane, *n*-hexane and toluene were obtained anhydrous from Honeywell and purified via an MBraun Solvent Purification System (SPS 800) incorporating activated alumina columns. THF for reduction was specifically dried over potassium for several days prior to use. All solvents were stored in argon-filled ampules sealed with Teflon plugs.

The protioligand $^{Cbz}(^{tBu}PNP)H$[1] and NaPb (10 w% Na)[2] were synthesized according to literature procedures. Solid organyl bromides for radical coupling were dried under vacuum and liquids were degassed by three freeze-pump-thaw cycles and stored under argon.

Samples for nuclear magnetic resonance (NMR) spectroscopy were prepared under inert atmosphere using 5 mm Wilmad J. Young Tubes. One-dimensional and two-dimensional ^{1}H, ^{2}H, ^{13}C, ^{31}P and ^{195}Pt NMR spectra were recorded using Bruker Avance II (400 MHz) or Bruker Avance III (600 MHz) spectrometers. Chemical shifts δ are reported in parts per million (ppm) and referenced to residual proton and carbon signals of the respective undeuterated solvent. Coupling constants J are given in Hertz (Hz). ^{31}P and ^{195}Pt NMR spectra were externally referenced to $P(OMe)_3$ (141.0 ppm with respect to 85 % H_3PO_4 at 0.0 ppm) and 1.2 M $Na_2[PtCl_6]$ in D_2O. Signal multiplicities are abbreviated as follows: singlet (s), broad singlet (bs), doublet (d), triplet (t), …, combinations thereof (e.g., dt for doublet of triplets), and multiplet (m).

Mass spectra were recorded at the Department of Organic Chemistry at the University of Heidelberg. Air-sensitive complexes were analyzed by Liquid Injection Field Desorption Ionization (LIFDI) and air-stable complexes by Matrix-Assisted Laser Desorption/Ionization (MALDI). Solid products from radical coupling reactions were analyzed by Electron ionization (EI) and liquid products by Gas chromatography-mass spectrometry (GC-MS). LIFDI, EI and GC-MS were recorded with a JEOL AccuTOF GCx orthogonal-acceleration time-of-flight (oaTOF) mass spectrometer and MALDI with a Bruker timsTOFfleX ion mobility-quadrupole-time-of-flight (IM-Q-TOF).

Elemental analyses (C, H, N) were performed at the Department of Inorganic Chemistry, University of Heidelberg, using an Elementar varia MICRO cube analyzer. X-band electron paramagnetic resonance (EPR) spectra were recorded under inert argon atmosphere utilizing a Bruker ELEX-SYS-E 500 spectrometer equipped with a premium bridge operating between 6-180 K or a Bruker ESP 300E instrument measuring at room temperature (295 K) and were simulated with the EasySpin v5.2.35 program in MATLAB. Infrared spectra were recorded on an Agilent Cary 630 spectrometer (ATR) within an argon-filled glovebox and processed using Origin 2024b.

ii. Synthesis and characterization data for precursor and Platinum(0) complexes

Synthesis of ^{Cbz}[^{tBu}PNP]Pt^{II}Cl (1-Cl)

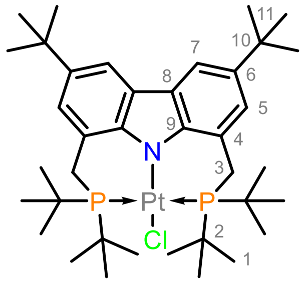

To a solution of ^{Cbz}[^{tBu}PNP]H (2.49 g, 4.18 mmol, 1.00 eq) in toluene (20 mL), LiHMDS (758 mg, 97%, 4.39 mmol, 1.05 eq) was added in portions and the yellow solution was stirred for 20 minutes. Then $(Et_2S)_2PtCl_2$ (2.05 g, 4.60 mmol, 1.10 eq) was added subsequently resulting in an color change to orange. After stirring over night, the reaction mixture was filtrated over Celite® and the pad was rinsed with additional toluene (20 mL). After evaporation of the solvent, the crude product was washed with cold *n*-pentane (2x 20 mL) and dried *in vacuo* to yield ^{Cbz}[^{tBu}PNP]Pt^{II}Cl as yellow solid (3.10 g, 3.76 mmol, 90%). **^{1}H NMR** (thf-d_8, 600 MHz, 295K): δ [ppm] = 7.91 (d, $^{4}J_{H\text{-}H}$ = 1.7 Hz, 2H, H^{7}), 7.16 (s, 2H, H^{5}), 3.50 (s, 4H, H^{3}), 1.49 (t, $^{3}J_{H\text{-}P}$ = 6.6 Hz, 36H, H^{1}), 1.42 (s, 18H, H^{11}). **^{13}C{^{1}H} NMR** (thf-d_8, 151 MHz, 295K): δ [ppm] = 147.9 (t, $^{3}J_{C\text{-}P}$ = 3.4 Hz, Cq, C^{9}), 139.9 (s, Cq, C^{6}), 126.1 (s, Cq, C^{8}), 123.9 (t, $^{3}J_{C\text{-}P}$ = 3.4 Hz, CH, C^{5}), 121.3 (s, Cq, C^{4}), 114.7 (s, CH, C^{7}), 38.0 (t, $^{1}J_{C\text{-}P}$ = 10.7 Hz, Cq, C^{2}), 34.7 (s, Cq, C^{10}), 32.3 (s, CH_3, C^{11}), 30.8 (s, CH_3, C^{1}), 21.8 (t, $^{1}J_{C\text{-}P}$ = 10.9 Hz, CH_2, C^{3}). **^{31}P{^{1}H} NMR** (thf-d_8, 243 MHz, 295K): δ [ppm] = 47.7 (s, $^{1}J_{P\text{-}Pt}$ = 2725 Hz). **^{195}Pt{^{1}H} NMR** (thf-d_8, 129 MHz, 295K): δ [ppm] = −3654.9 (t, $^{1}J_{Pt\text{-}P}$ = 2723 Hz). **HR-MS** (MALDI, DCTB in DCM): calc. for $[C_{38}H_{62}ClNP_2Pt]^+$: 824.3688, found m/z: 824.3686 $[M]^+$. The expected isotopic pattern agrees well with the measurement. **EA**: calc. for $C_{38}H_{62}ClNP_2Pt$: C 55.30, H 7.57, N 1.70. Found: C 54.90, H 7.34, N 1.70. **IR** [cm^{-1}]: ṽ = 2948, 2896, 2863, 1584, 1476, 1428, 1390, 1361, 1327, 1282, 1267, 1226, 1200, 1174, 1148, 1100, 1018, 936, 865, 813, 734.

Synthesis of ^{Cbz}[^{tBu}PNP]Pt^{0}Na (2-Na)

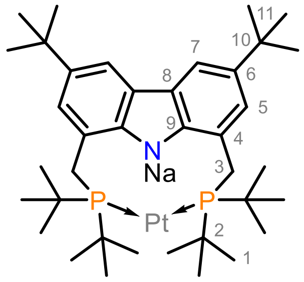

^{Cbz}[^{tBu}PNP]Pt^{II}Cl (100 mg, 121 µmol, 1.00 eq) was dissolved in THF (1.0 mL) and NaPb (139 mg, 10 w% Na, 608 µmol, 5.00 eq) was added. After stirring over night at rt, the reaction mixture was filtrated into a J. Young Tube. For reactions requiring exact stoichiometry, the conversion was determined by addition of hexamethyldisilane (24.8 µL, 121 µmol, 1.00 eq) as internal standard for integration in ^{1}H NMR. The *in situ* generated ^{Cbz}[^{tBu}PNP]Pt^{0}Na was used without further purification for reactivity studies. It should be noted that an average of two percent of the hydride species **3-H** are formed during reduction. For NMR analysis, the reaction was carried out on a half-scale basis in deuterated THF (0.5 mL), and NMR spectra were measured after filtration. IR spectra were obtained by evaporating the solution directly on the device. Single crystals suitable for X-ray diffraction were grown from a saturated 1,4-Dioxane solution by carefully overlaying with *n*-hexane at rt. For further analysis (MS and EA), the crystals obtained were first dried under vacuum. **^{1}H NMR** (thf-d_8, 600 MHz, 295K): δ [ppm] = 7.91 (d, $^{4}J_{H\text{-}H}$ = 1.8 Hz, 2H, H^{7}), 7.19 (s, 2H, H^{5}), 3.45 (bs, 4H, H^{3}), 1.44 (s, 18H, H^{11}), 1.25 (bs, 36H, H^{1}). **^{13}C{^{1}H} NMR** (thf-d_8, 151 MHz, 295K): δ [ppm] = 147.9 (s, Cq, C^{9}), 134.8 (s, Cq, C^{6}), 125.6 (s, Cq, C^{8}), 123.3 (s, CH, C^{5}), 122.8 (s, Cq, C^{4}), 114.0 (s, CH, C^{7}), 34.8 (s, Cq, C^{10}), 32.8 (s, CH_3, C^{11}), 30.6 (bs, Cq, C^{2}), 29.6-29.8 (m, CH_3, C^{1}), 28.3 (t, $^{1}J_{C\text{-}P}$ = 5.6 Hz, CH_2, C^{3}). **^{31}P{^{1}H} NMR** (thf-d_8, 243 MHz, 295K): δ [ppm] = 93.7 (s, $^{1}J_{P\text{-}Pt}$ = 4964 Hz). **^{195}Pt{^{1}H} NMR** (thf-d_8, 129 MHz, 295K): δ [ppm] = −5327.9 (t, $^{1}J_{Pt\text{-}P}$ = 4959 Hz). **MS** (LIFDI, THF): calc. for $[C_{38}H_{62}NNaP_2Pt]^+$: 812.4, found m/z: 790.4 $[M\text{-}Na\text{+}H]^+$ (decomposition). **EA**: calc. for $C_{38}H_{62}NNaP_2Pt$: C 56.14, H 7.69, N 1.72. Found: C 55.77, H 7.71, N 1.60. **IR** [cm^{-1}]: ṽ = 2948, 2896, 2863, 1558, 1472, 1457, 1428, 1405, 1390, 1361, 1301, 1282, 1234, 1200, 1182, 1074, 1018, 932, 913, 865, 850, 809, 701.

Synthesis of ^{Cbz}[^{tBu}PNP]Pt^{0}MgCl (2-Mg)

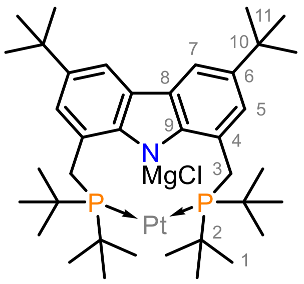

^{Cbz}[^{tBu}PNP]Pt^{II}Cl (100 mg, 121 µmol, 1.00 eq) was dissolved in THF (1.0 mL), then Mg (8.83 mg, 364 µmol, 3.00 eq) and $HgCl_2$ (32.9 mg, 121 µmol, 1.00 eq) were added. After stirring for 3h at rt, the reaction mixture was filtrated into a J. Young Tube. For reactions requiring exact stoichiometry, the conversion was determined by addition of hexamethyldisilane (24.8 µL, 121 µmol, 1.00 eq) as internal standard for integration in ^{1}H NMR. The *in situ* generated ^{Cbz}[^{tBu}PNP]Pt^{0}MgCl was used without further purification for reactivity studies. It should be noted that an average of two percent of the hydride species **3-H** are formed during reduction. For NMR analysis, the reaction was carried out on a half-scale basis in deuterated THF (0.5 mL), and NMR spectra were measured after filtration. IR spectra were obtained by evaporating the solution directly on the device. Single crystals suitable for X-ray diffraction were grown from a THF solution by carefully overlaying with *n*-pentane at −40 °C. For further analysis (MS and EA), the crystals obtained were first dried under vacuum. **^{1}H NMR** (thf-d_8, 600 MHz, 295K): δ [ppm] = 7.89 (s, 2H, H^7), 7.29 (s, 2H, H^5), 3.85 (d, $^2J_{H\text{-}H}$ = 13.8 Hz, 2H, H^3), 3.44 (d, $^2J_{H\text{-}H}$ = 13.8 Hz, 2H, H^3), 1.59 (t, $^3J_{H\text{-}P}$ = 6.1 Hz, 18H, H^1), 1.42 (s, 18H, H^{11}), 0.73 (t, $^3J_{H\text{-}P}$ = 6.0 Hz, 18H, H^1). **^{13}C{^{1}H} NMR** (thf-d_8, 151 MHz, 295K): δ [ppm] = 146.7 (s, Cq, C^9), 138.2 (s, Cq, C^6), 126.6 (s, Cq, C^8), 125.1 (s, CH, C^5), 122.2 (s, Cq, C^4), 114.4 (s, CH, C^7), 36.4 (t, $^1J_{C\text{-}P}$ = 9.1 Hz, Cq, C^2), 35.9 (t, $^1J_{C\text{-}P}$ = 8.9 Hz, Cq, C^2), 34.8 (s, Cq, C^{10}), 32.4 (s, CH_3, C^{11}), 31.9 (t, $^2J_{C\text{-}P}$ = 3.8 Hz, CH_3, C^1), 31.0 (t, $^1J_{C\text{-}P}$ = 5.9 Hz, CH_2, C^3), 30.3 (s, CH_3, C^1). **^{31}P{^{1}H} NMR** (thf-d_8, 243 MHz, 295K): δ [ppm] = 86.2 (s, $^1J_{P\text{-}Pt}$ = 4497 Hz). **^{195}Pt{^{1}H} NMR** (thf-d_8, 129 MHz, 295K): δ [ppm] = −5703.8 (t, $^1J_{Pt\text{-}P}$ = 4487 Hz). **MS** (LIFDI, THF): calc. for $[C_{38}H_{62}NMgClP_2Pt]^+$: 848.4, found m/z: 790.4 $[M\text{-}MgCl+H]^+$ (decomposition). **EA**: calc. for $C_{38}H_{62}NMgClP_2Pt$: C 53.71, H 7.36, N 1.65. Found: C 53.76, H 7.13, N 1.57. **IR** [cm^{-1}]: ṽ = 2948, 2896, 2863, 2236, 2188, 2162, 2125, 2113, 2084, 1562, 1476, 1461, 1413, 1387, 1361, 1308, 1282, 1230, 1178, 1100, 1047, 999, 936, 865, 850, 828, 809, 753.

Synthesis of ^{Cbz}[^{tBu}PNP]Pt^{II}H (3-H)

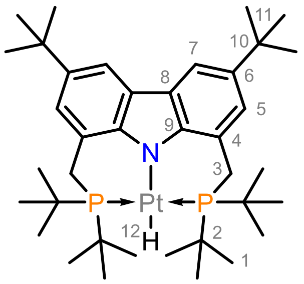

To a solution of ^{Cbz}[^{tBu}PNP]H (135 mg, 227 µmol, 1.00 eq) in toluene (2.0 mL), (^{t}Bu$_3$P)$_2$Pt (139 mg, 231 µmol, 1.02 eq) was added and the colorless solution was stirred over night. After evaporation of the solvent, the crude product was washed with cold *n*-hexane (2x 2.0 mL) and dried *in vacuo* to yield ^{Cbz}[^{tBu}PNP]Pt^{II}H as white solid (152 mg, 192 µmol, 85%). Single crystals suitable for X-ray diffraction were grown from a saturated Et_2O solution at −40 °C. **^{1}H NMR** (thf-d_8, 600 MHz, 295K): δ [ppm] = 7.96 (s, 2H, H^7), 7.17 (s, 2H, H^5), 3.55 (s, 4H, H^3), 1.42 (s, 18H, H^{11}), 1.30 (t, $^3J_{H\text{-}P}$ = 6.6 Hz, 36H, H^1), −15.00 (t, $^2J_{H\text{-}P}$ = 15.4 Hz, $^1J_{H\text{-}Pt}$ = 911 Hz, 1H, H^{12}). **^{13}C{^{1}H} NMR** (thf-d_8, 151 MHz, 295K): δ [ppm] = 147.2 (t, $^3J_{C\text{-}P}$ = 3.2 Hz, Cq, C^9), 137.8 (s, Cq, C^6), 125.6 (s, $^3J_{C\text{-}Pt}$ = 14.1 Hz, Cq, C^8), 123.8 (t, $^3J_{C\text{-}P}$ = 3.7 Hz, CH, C^5), 122.1 (s, $^3J_{C\text{-}Pt}$ = 18.1 Hz, Cq, C^4), 114.8 (s, CH, C^7), 35.7 (t, $^1J_{C\text{-}P}$ = 12.7 Hz, $^2J_{C\text{-}Pt}$ = 45.9 Hz, Cq, C^2), 34.7 (s, Cq, C^{10}), 32.5 (s, CH_3, C^{11}), 29.7 (t, $^2J_{C\text{-}P}$ = 2.4 Hz, $^3J_{C\text{-}Pt}$ = 20.5 Hz, CH_3, C^1), 21.3 (t, $^1J_{C\text{-}P}$ = 10.3 Hz, CH_2, C^3). **^{31}P{^{1}H} NMR** (thf-d_8, 243 MHz, 295K): δ [ppm] = 71.4 (s, $^1J_{P\text{-}Pt}$ = 2884 Hz). **^{195}Pt{^{1}H} NMR** (thf-d_8, 129 MHz, 295K): δ [ppm] = −5160.5 (t, $^1J_{Pt\text{-}P}$ = 2882 Hz). **HR-MS** (MALDI, DCTB in DCM): calc. for $[C_{38}H_{63}NP_2Pt]^+$: 790.4078, found m/z: 790.4110 $[M]^+$. The expected isotopic pattern agrees well with the measurement. **EA**: calc. for $C_{38}H_{63}NP_2Pt$: C 57.70, H 8.03, N 1.77. Found: C 57.93, H 7.79, N 1.75. **IR** [cm^{-1}]: ṽ = 2945, 2896, 2863, 2177, 1573, 1472, 1424, 1387, 1361, 1316, 1282, 1230, 1197, 1178, 1148, 1100, 1018, 954, 936, 865, 828, 813, 734.

Synthesis of ^{Cbz}[^{tBu}PNP]Pt^{II}D (3-D)

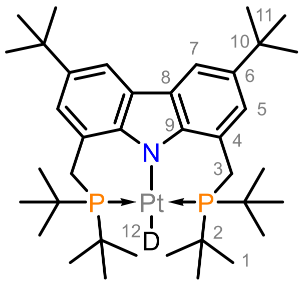

^{Cbz}[^{tBu}PNP]Pt^{II}Cl (50.0 mg, 60.6 µmol, 1.00 eq) was dissolved in THF (0.8 mL) and NaPb (69.7 mg, 10 w% Na, 303 µmol, 5.00 eq) was added. After stirring over night at rt, the reaction mixture was filtrated into a J. Young Tube. Some drops of D_2O (excess, ≈ 0.1 mL) were added, causing a color change from orange-red to pale yellow. All volatiles were removed *in vacuo* and the residue was taken up in benzene (0.5 mL). The yellow solutions was filtrated by syringe and the solvent was removed again. ^{Cbz}[^{tBu}PNP]Pt^{II}D was obtained as off-white solid (39.0 mg, 49.2 µmol, 81%). **^{1}H NMR** (thf-d_8, 600 MHz, 295K): δ [ppm] = 7.96 (d, $^{4}J_{H-H}$ = 1.6 Hz, 2H, H^{7}), 7.17 (s, 2H, H^{5}), 3.55 (s, 4H, H^{3}), 1.43 (s, 18H, H^{11}), 1.30 (t, $^{3}J_{H-P}$ = 6.6 Hz, 36H, H^{1}). **^{2}H NMR** (thf-d_8, 92 MHz, 295K): δ [ppm] = −14.9 (s, $^{1}J_{D-Pt}$ = 139 Hz, D^{12}). **^{13}C{^{1}H}NMR** (thf-d_8, 151 MHz, 295K): δ [ppm] = 147.2 (t, $^{3}J_{C-P}$ = 3.2 Hz, Cq, C^{9}), 137.8 (s, Cq, C^{6}), 125.6 (s, $^{3}J_{C-Pt}$ = 14.3 Hz, Cq, C^{8}), 123.8 (t, $^{3}J_{C-P}$ = 3.7 Hz, CH, C^{5}), 122.1 (s, $^{3}J_{C-Pt}$ = 18.0 Hz, Cq, C^{4}), 114.8 (s, CH, C^{7}), 35.7 (t, $^{1}J_{C-P}$ = 12.7 Hz, $^{2}J_{C-Pt}$ = 46.1 Hz, Cq, C^{2}), 34.7 (s, Cq, C^{10}), 32.5 (s, CH_3, C^{11}), 29.7 (t, $^{2}J_{C-P}$ = 2.4 Hz, $^{3}J_{C-Pt}$ = 20.7 Hz, CH_3, C^{1}), 21.3 (t, $^{1}J_{C-P}$ = 10.3 Hz, CH_2, C^{3}). **^{31}P{^{1}H} NMR** (thf-d_8, 243 MHz, 295K): δ [ppm] = 73.3 (s, $^{1}J_{P-Pt}$ = 2886 Hz). **^{195}Pt{^{1}H} NMR** (thf-d_8, 129 MHz, 295K): δ [ppm] = −5167.1 (tt, $^{1}J_{Pt-P}$ = 2886 Hz, $^{1}J_{Pt-D}$ = 139 Hz). **HR-MS** (MALDI, DCTB in DCM): calc. for $[C_{38}H_{62}DNP_2Pt]^+$: 791.4141, found m/z: 791.4170 $[M]^+$. The expected isotopic pattern agrees well with the measurement. **EA**: calc. for $C_{38}H_{62}DNP_2Pt$: C 57.63, H 8.15, N 1.77. Found: C 57.82, H 7.84, N 1.79. **IR** [cm^{-1}]: ṽ = 2948, 2900, 2866, 1562, 1476, 1428, 1390, 1361, 1316, 1282, 1264, 1234, 1208, 1182, 1148, 1100, 1018, 958, 936, 861, 809, 738.

iii. Synthesis of bromide and organyl complexes and characterization data

Synthesis of ^{Cbz}[^{tBu}PNP]Pt^{II}Br (1-Br)

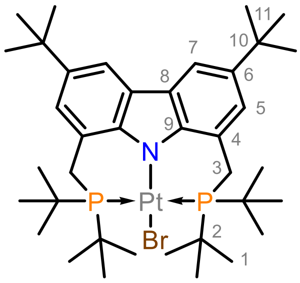

As stated before, **2-Na** or **2-Mg** was generated *in situ* from ^{Cbz}[^{tBu}PNP]Pt^{II}Cl (25.0 mg, 30.3 µmol, 1.00 eq) in THF (0.5 mL). Benzyl bromide (7.20 µL, 60.6 µmol, 2.00 eq) was added and the J. Young Tube was shaken vigorously. After indicating full conversion by ^{31}P NMR, the crude solution was filtrated by syringe and all volatiles were removed *in vacuo*. To remove 1,2-Diphenylethane, the crude product was purified by flash column chromatography (*n*-hexane:DCM = 3:1). ^{Cbz}[^{tBu}PNP]Pt^{II}Br was obtained as yellow solid (22.8 mg, 26.2 µmol, 87%). Single crystals suitable for X-ray diffraction were grown from a saturated Et_2O solution at −40 °C. **^{1}H NMR** (C_6D_6, 600 MHz, 295K): δ [ppm] = 8.31 (d, $^{4}J_{H\text{-}H}$ = 1.6 Hz, 2H, H^{7}), 7.24 (s, 2H, H^{5}), 3.25 (s, 4H, H^{3}), 1.54 (s, 18H, H^{11}), 1.43 (t, $^{3}J_{H\text{-}P}$ = 6.7 Hz, 36H, H^{1}). **^{13}C{^{1}H} NMR** (C_6D_6, 151 MHz, 295K): δ [ppm] = 148.3 (t, $^{3}J_{C\text{-}P}$ = 3.4 Hz, Cq, C^{9}), 140.1 (s, Cq, C^{6}), 126.1 (s, Cq, C^{8}), 123.8 (t, $^{3}J_{C\text{-}P}$ = 3.2 Hz, CH, C^{5}), 120.7 (s, Cq, C^{4}), 115.1 (s, CH, C^{7}), 38.0 (t, $^{1}J_{C\text{-}P}$ = 10.5 Hz, Cq, C^{2}), 34.6 (s, Cq, C^{10}), 32.4 (s, CH_3, C^{11}), 31.1 (s, CH_3, C^{1}), 22.0 (t, $^{1}J_{C\text{-}P}$ = 10.5 Hz, CH_2, C^{3}). **^{31}P{^{1}H} NMR** (C_6D_6, 243 MHz, 295K): δ [ppm] = 48.8 (s, $^{1}J_{P\text{-}Pt}$ = 2718 Hz). **^{195}Pt{^{1}H} NMR** (C_6D_6, 129 MHz, 295K): δ [ppm] = −3885.6 (t, $^{1}J_{Pt\text{-}P}$ = 2710 Hz). **HR-MS** (MALDI, DCTB in DCM): calc. for $[C_{38}H_{62}BrNP_2Pt]^+$: 868.3183, found m/z: 868.3184 $[M]^+$. The expected isotopic pattern agrees well with the measurement. **EA**: calc. for $C_{38}H_{62}BrNP_2Pt$: C 52.47, H 7.18, N 1.61. Found: C 52.99, H 7.20, N 1.32. **IR** [cm^{-1}]: ṽ = 2952, 2900, 2866, 1588 1476, 1431, 1390, 1364, 1327, 1286, 1267, 1230, 1204, 1174, 1103, 1021, 962, 936, 865, 809, 734.

Synthesis of ^{Cbz}[^{tBu}PNP]Pt^{II}$CH_2CH_2CH{=}CH_2$ (4a)

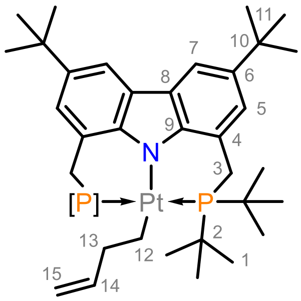

As stated before, **2-Na** or **2-Mg** was generated *in situ* from ^{Cbz}[^{tBu}PNP]Pt^{II}Cl (100 mg, 121 µmol, 1.00 eq) in THF (1.0 mL). (Bromomethyl)cyclopropane (22.8 µL, 242 µmol, 2.00 eq) was added and the J. Young Tube was shaken vigorously, causing a color change from orange-red to yellow and the formation of a precipitate. The crude solution was filtrated by syringe and all volatiles were removed *in vacuo*. The remaining solid was purified by flash column chromatography (*n*-hexane:DCM = 1:1 to pure EtOAc). ^{Cbz}[^{tBu}PNP]Pt^{II}$CH_2CH_2CH{=}CH_2$ was obtained as off-white solid (45.3 mg, 53.6 µmol, 44%). Single crystals suitable for X-ray diffraction were grown from a saturated Et_2O solution at −40 °C. **^{1}H NMR** (C_6D_6, 600 MHz, 295K): δ [ppm] = 8.42 (d, $^{4}J_{H\text{-}H}$ = 1.3 Hz, 2H, H^{7}), 7.32 (s, 2H,H^{5}), 5.89-5.84 (m, 1H, H^{14}), 5.03 (dd, $^{3}J_{H\text{-}H}$ = 17.1 Hz, $^{2}J_{H\text{-}H}$ = 1.8 Hz, 1H, H^{15}), 4.94 (dd, $^{3}J_{H\text{-}H}$ = 10.2 Hz, $^{2}J_{H\text{-}H}$ = 1.8 Hz, 1H, H^{15}), 3.32 (s, 4H, H^{3}), 2.46-2.31 (m, 2H, H^{12}), 2.30-2.24 (m, 2H, H^{13}), 1.61 (s, 18H, H^{11}), 1.28 (t, $^{3}J_{H\text{-}P}$ = 5.7 Hz, 36H, H^{1}). **^{13}C{^{1}H} NMR** (C_6D_6, 151 MHz, 295K): δ [ppm] = 150.0 (t, $^{3}J_{C\text{-}P}$ = 2.9 Hz, Cq, C^{9}), 142.0 (s, $^{3}J_{C\text{-}Pt}$ = 79.1Hz, CH, C^{14}), 138.3 (s, Cq, C^{6}), 125.8 (s, Cq, C^{8}), 122.1 (t, $^{3}J_{C\text{-}P}$ = 2.9 Hz, CH, C^{5}), 120.4 (s, Cq, C^{4}), 115.1 (s, CH, C^{7}), 112.2 (s, CH_2, C^{15}), 37.3 (t, $^{1}J_{C\text{-}P}$ = 9.6 Hz, Cq, C^{2}), 37.1 (s, CH_2, C^{13}), 34.7 (s, Cq, C^{10}), 32.7 (s, CH_3, C^{11}), 30.6 (s, CH_3, C^{1}), 22.8 (t, $^{1}J_{C\text{-}P}$ = 10.9 Hz, CH_2, C^{3}), −21.7 (t, $^{2}J_{C\text{-}P}$ = 8.0 Hz, $^{1}J_{C\text{-}Pt}$ = 601 Hz, CH_2, C^{12}). **^{31}P{^{1}H} NMR** (C_6D_6, 243 MHz, 295K): δ [ppm] = 53.8 (s, $^{1}J_{P\text{-}Pt}$ = 3183 Hz). **^{195}Pt{^{1}H} NMR** (thf-d_8, 129 MHz, 295K): δ [ppm] = −4345.3 (t, $^{1}J_{Pt\text{-}P}$ = 3180 Hz). **HR-MS** (MALDI, DCTB in DCM): calc. for $[C_{42}H_{69}NP_2Pt]^+$: 844.4548, found m/z: 844.4557 $[M]^+$. The expected isotopic pattern agrees well with the measurement. **EA**: calc. for $C_{42}H_{69}NP_2Pt$: C 59.70, H 8.23, N 1.66. Found: C 59.14, H 7.91, N 1.63. **IR** [cm^{-1}]: ṽ = 2952, 2900, 2870, 1633, 1588, 1476, 1431, 1390, 1361, 1320, 1282, 1234, 1174, 1144, 1096, 1018, 936, 902, 861, 809, 731.

Synthesis of $^{Cbz}[^{tBu}PNP]Pt^{II}CH_2CH(CH_2)_4$ (4b)

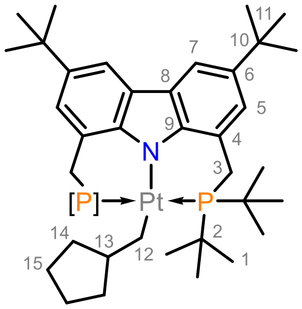

As stated before, **2-Na** or **2-Mg** was generated *in situ* from $^{Cbz}[^{tBu}PNP]Pt^{II}Cl$ (100 mg, 121 µmol, 1.00 eq) in THF (1.0 mL). (Bromomethyl)cyclopentane (29.9 µL, 242 µmol, 2.00 eq) or 6-Brom-1-hexen (32.4 µL, 242 µmol, 2.00 eq) was added and the J. Young Tube was shaken vigorously, causing a color change from orange-red to yellow and the formation of a precipitate. The crude solution was filtrated by syringe and all volatiles were removed *in vacuo*. The remaining solid was purified by flash column chromatography (*n*-hexane:DCM = 1:1 to pure EtOAc). $^{Cbz}[^{tBu}PNP]Pt^{II}CH_2CH_2CH{=}CH_2$ was obtained as off-white solid (31.4 mg, 36.0 µmol, 30%). Single crystals suitable for X-ray diffraction were grown from a saturated Et_2O solution at −40 °C. **^{1}H NMR** (C_6D_6, 600 MHz, 295K): δ [ppm] = 8.41 (d, $^4J_{H\text{-}H}$ = 1.5 Hz, 2H, H^7), 7.33 (s, 2H,H^5), 3.38 (bs, 4H, H^3), 2.40-2.23 (m, 2H, H^{12}), 2.19-2.13 (m, 1H, H^{13}), 1.82-1.76 (m, 2H, H^{14}), 1.63-1.58 (m, 20H, H^{11}/H^{15}), 1.41 (bs, 20H, H^1/H^{15}), 1.26 (bs, 20H, H^1/H^{14}). **^{13}C{^{1}H} NMR** (C_6D_6, 151 MHz, 295K): δ [ppm] = 150.1 (t, $^3J_{C\text{-}P}$ = 2.6 Hz, Cq, C^9), 138.1 (s, Cq, C^6), 125.7 (s, Cq, C^8), 122.6 (t, $^3J_{C\text{-}P}$ = 3.1 Hz, CH, C^5), 120.3 (s, Cq, C^4), 114.9 (s, CH, C^7), 48.3 (t, $^3J_{C\text{-}P}$ = 3.5 Hz, CH, C^{13}), 37.3 (bs, Cq, C^2), 36.8 (s, CH$_2$, C^{14}), 37.3 (bs, Cq, C^2), 34.7 (s, Cq, C^{10}), 32.7 (s, CH$_3$, C^{11}), 31.0 (bs, CH$_3$, C^1), 30.4 (bs, CH$_3$, C^1), 25.4 (s, CH$_2$, C^{15}), 23.8 (t, $^1J_{C\text{-}P}$ = 10.8 Hz, CH$_2$, C^3), −11.3 (t, $^2J_{C\text{-}P}$ = 7.4 Hz, $^1J_{C\text{-}Pt}$ = 597 Hz, CH$_2$, C^{12}). **^{31}P{^{1}H} NMR** (C_6D_6, 243 MHz, 295K): δ [ppm] = 50.7 (s, $^1J_{P\text{-}Pt}$ = 3244 Hz). **^{195}Pt{^{1}H} NMR** (thf-d$_8$, 129 MHz, 295K): δ [ppm] = −4289.4 (t, $^1J_{Pt\text{-}P}$ = 3242 Hz). **HR-MS** (MALDI, DCTB in DCM): calc. for $[C_{44}H_{73}NP_2Pt]^+$: 872.4861, found m/z: 872.4865 [M]$^+$. The expected isotopic pattern agrees well with the measurement. **EA**: calc. for $C_{44}H_{73}NP_2Pt$: C 60.53, H 8.43, N 1.60. Found: C 61.18, H 8.98, N 1.56. **IR**[cm^{-1}]: ṽ = 2952, 2904, 2870, 1588, 1476, 1435, 1390, 1361, 1320, 1286, 1238, 1208, 1174, 1144, 1100, 1018, 936, 865, 824, 809, 727.

Synthesis of $^{Cbz}[^{tBu}PNP]Pt^{II}CH_2CH{=}CH_2$ (4c)

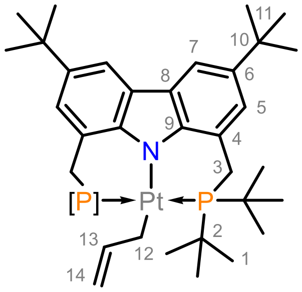

As stated before, **2-Na** or **2-Mg** was generated *in situ* from $^{Cbz}[^{tBu}PNP]Pt^{II}Cl$ (100 mg, 121 µmol, 1.00 eq) in THF (1.0 mL). Allyl bromide (20.9 µL, 242 µmol, 2.00 eq) was added and the J. Young Tube was shaken vigorously, causing a color change from orange-red to yellow and the formation of a precipitate. The crude solution was filtrated by syringe and all volatiles were removed *in vacuo*. The remaining solid was purified by flash column chromatography (*n*-hexane:DCM = 1:1 to pure EtOAc). $^{Cbz}[^{tBu}PNP]Pt^{II}CH_2CH{=}CH_2$ was obtained as off-white solid (42.7 mg, 51.4 µmol, 42%). **^{1}H NMR** (C_6D_6, 600 MHz, 295K): δ [ppm] = 8.40 (s, 2H, H^7), 7.32 (s, 2H, H^5), 6.18-6.12 (m, 1H, H^{13}), 4.98 (d, $^3J_{H\text{-}H}$ = 17.9 Hz, 1H, H^{14}), 4.85 (d, $^3J_{H\text{-}H}$ = 10.3 Hz, 1H, H^{14}), 3.40-3.22 (m, 2H, H^{12}), 3.33 (s, 4H, H^3), 1.61 (s, 18H, H^{11}), 1.29 (t, $^3J_{H\text{-}P}$ = 6.1 Hz, 36H, H^1). **^{13}C{^{1}H} NMR** (C_6D_6, 151 MHz, 295K): δ [ppm] = 150.0 (t, $^3J_{C\text{-}P}$ = 3.0 Hz, Cq, C^9), 147.5 (s, $^2J_{C\text{-}Pt}$ = 51.2 Hz, CH, C^{13}), 138.5 (s, Cq, C^6), 125.8 (s, Cq, C^8), 122.3 (t, $^3J_{C\text{-}P}$ = 3.1 Hz, CH, C^5), 120.4 (s, Cq, C^4), 115.0 (s, CH, C^7), 112.3 (s, $^3J_{C\text{-}Pt}$ = 61.8 Hz, CH$_2$, C^{14}), 37.4 (t, $^1J_{C\text{-}P}$ = 9.7 Hz, Cq, C^2), 34.7 (s, Cq, C^{10}), 32.7 (s, CH$_3$, C^{11}), 30.6 (s, CH$_3$, C^1), 22.8 (t, $^1J_{C\text{-}P}$ = 10.9 Hz, CH$_2$, C^3), −17.0 (t, $^2J_{C\text{-}P}$ = 7.9 Hz, $^1J_{C\text{-}Pt}$ = 581 Hz, CH$_2$, C^{12}). **^{31}P{^{1}H} NMR** (C_6D_6, 243 MHz, 295K): δ [ppm] = 53.6 (s, $^1J_{P\text{-}Pt}$ = 3150 Hz). **^{195}Pt{^{1}H} NMR** (thf-d$_8$, 129 MHz, 295K): δ [ppm] = −4321.7 (t, $^1J_{Pt\text{-}P}$ = 3148 Hz). **HR-MS** (MALDI, DCTB in DCM): calc. for $[C_{41}H_{67}NP_2Pt]^+$: 830.4391, found m/z: 830.4401 [M]$^+$. The expected isotopic pattern agrees well with the measurement. **EA**: calc. for $C_{41}H_{67}NP_2Pt$: C 59.26, H 8.13, N 1.69. Found: C 58.91, H 7.74, N 1.67. **IR** [cm^{-1}]: ṽ = 2952, 2900, 2866, 1610, 1588, 1476, 1431, 1390, 1361, 1320, 1282, 1234, 1174, 1144, 1100, 1018, 992, 936, 887, 861, 809, 727.

Synthesis of $^{Cbz}[^{tBu}PNP]Pt^{II}CH_2CH_2CH_2CH_3$ (4d)

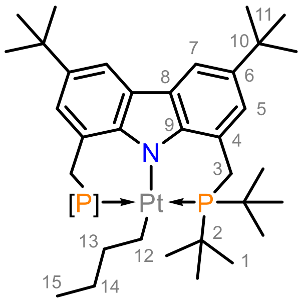

As stated before, **2-Na** or **2-Mg** was generated *in situ* from $^{Cbz}[^{tBu}PNP]Pt^{II}Cl$ (100 mg, 121 µmol, 1.00 eq) in THF (1.0 mL). 1-Bromobutane (23.1 µL, 242 µmol, 2.00 eq) was added and the J. Young Tube was shaken vigorously, causing a color change from orange-red to yellow and the formation of a precipitate. The crude solution was filtrated by syringe and all volatiles were removed *in vacuo*. The remaining solid was purified by flash column chromatography (*n*-hexane:DCM = 1:1 to pure EtOAc). $^{Cbz}[^{tBu}PNP]Pt^{II}CH_2CH_2CH_2CH_3$ was obtained as off-white solid (32.7 mg, 38.6 µmol, 32%). **^{1}H NMR** (C_6D_6, 600 MHz, 295K): δ [ppm] = 8.44 (d, $^4J_{H\text{-}H}$ = 1.5 Hz, 2H, H^7), 7.33 (s, 2H,H^5), 3.34 (s, 4H, H^3), 2.30-2.24 (m, 2H, H^{12}), 1.62 (s, 18H, H^{11}), 1.49-1.43 (m, 2H, H^{13}), 1.37-1.32 (m, 1H, H^{14}), 1.29 (t, $^3J_{H\text{-}P}$ = 5.7 Hz, 36H, H^1), 0.90 (t, $^3J_{H\text{-}H}$ = 7.3 Hz, 3H, H^{15}). **^{13}C{^{1}H} NMR** (C_6D_6, 151 MHz, 295K): δ [ppm] = 150.1 (t, $^3J_{C\text{-}P}$ = 3.0 Hz, Cq, C^9), 138.3 (s, Cq, C^6), 125.8 (s, Cq, C^8), 122.1 (t, $^3J_{C\text{-}P}$ = 3.1 Hz, CH, C^5), 120.4 (s, Cq, C^4), 115.0 (s, CH, C^7), 37.3 (t, $^1J_{C\text{-}P}$ = 9.5 Hz, Cq, C^2), 35.4 (s, CH_2, C^{13}), 34.7 (s, Cq, C^{10}), 32.7 (s, CH_3, C^{11}), 30.7 (s, CH_3, C^1), 27.1 (s, $^3J_{C\text{-}Pt}$ = 85.7 Hz, CH_2, C^{14}), 22.9 (t, $^1J_{C\text{-}P}$ = 10.8 Hz, CH_2, C^3), 14.3 (s, CH_3, C^{15}), −19.2 (t, $^2J_{C\text{-}P}$ = 7.8 Hz, $^1J_{C\text{-}Pt}$ = 592 Hz, CH_2, C^{12}). **^{31}P{^{1}H} NMR** (C_6D_6, 243 MHz, 295K): δ [ppm] = 53.8 (s, $^1J_{P\text{-}Pt}$ = 3221 Hz). **^{195}Pt{^{1}H} NMR** (thf-d_8, 129 MHz, 295K): δ [ppm] = −4358.9 (t, $^1J_{Pt\text{-}P}$ = 3217 Hz). **HR-MS** (MALDI, DCTB in DCM): calc. for $[C_{42}H_{71}NP_2Pt]^+$: 846.4704, found m/z: 846.4714 $[M]^+$. The expected isotopic pattern agrees well with the measurement. **EA**: calc. for $C_{42}H_{71}NP_2Pt$: C 59.55, H 8.45, N 1.65. Found: C 59.90, H 9.06, N 1.77. **IR** [cm^{-1}]: ṽ = 2956, 2900, 2870, 1588, 1476, 1431, 1390, 1361, 1320, 1282, 1260, 1234, 1174, 1096, 1018, 936, 861, 809, 727.

Synthesis of $^{Cbz}[^{tBu}PNP]Pt^{II}CH_3$ (4e)

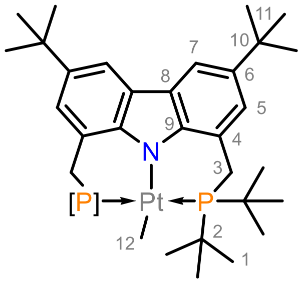

As stated before, **2-Na** or **2-Mg** was generated *in situ* from $^{Cbz}[^{tBu}PNP]Pt^{II}Cl$ (100 mg, 121 µmol, 1.00 eq) in THF (1.0 mL). Bromomethane (≈ 1-2 bar) was added and the Pressure J. Young Tube was shaken vigorously, causing a color change from orange-red to yellow and the formation of a precipitate. The crude solution was filtrated by syringe and all volatiles were removed *in vacuo*. $^{Cbz}[^{tBu}PNP]Pt^{II}CH_3$ was obtained as off-white solid (85.7 mg, 107µmol, 88%). **^{1}H NMR** (C_6D_6, 600 MHz, 295K): δ [ppm] = 8.42 (d, $^4J_{H\text{-}H}$ = 1.2 Hz, 2H, H^7), 7.33 (s, 2H,H^5), 3.39 (s, 4H, H^3), 1.61 (s, 18H, H^{11}), 1.24 (t, $^3J_{H\text{-}P}$ = 6.3 Hz, 36H, H^1), 1.21 (t, $^3J_{H\text{-}P}$ = 6.7 Hz, 3H, H^{12}). **^{13}C{^{1}H} NMR** (C_6D_6, 151 MHz, 295K): δ [ppm] = 149.1 (t, $^3J_{C\text{-}P}$ = 3.0 Hz, Cq, C^9), 138.1 (s, Cq, C^6), 125.6 (s, Cq, C^8), 123.1 (t, $^3J_{C\text{-}P}$ = 3.2 Hz, CH, C^5), 120.3 (s, Cq, C^4), 115.2 (s, CH, C^7), 36.8 (t, $^1J_{C\text{-}P}$ = 10.2 Hz, Cq, C^2), 34.7 (s, Cq, C^{10}), 32.6 (s, CH_3, C^{11}), 30.6 (s, CH_3, C^1), 23.0 (t, $^1J_{C\text{-}P}$ = 10.6 Hz, CH_2, C^3), −35.4 (t, $^2J_{C\text{-}P}$ = 9.3 Hz, $^1J_{C\text{-}Pt}$ = 558 Hz, CH_2, C^{12}). **^{31}P{^{1}H} NMR** (C_6D_6, 162 MHz, 295K): δ [ppm] = 52.6 (s, $^1J_{P\text{-}Pt}$ = 3015 Hz). **^{195}Pt{^{1}H} NMR** (thf-d_8, 129 MHz, 295K): δ [ppm] = −4411.2 (t, $^1J_{Pt\text{-}P}$ = 3013 Hz). **HR-MS** (MALDI, DCTB in DCM): calc. for $[C_{39}H_{65}NP_2Pt]^+$: 804.4235, found m/z: 804.4245 $[M]^+$. The expected isotopic pattern agrees well with the measurement. **EA**: calc. for $C_{39}H_{65}NP_2Pt$: C 58.19, H 8.14, N 1.74. Found: C 58.18, H 7.86, N 1.73. **IR** [cm^{-1}]: ṽ = 2952, 2900, 2866, 1584, 1476, 1431, 1390, 1361, 1320, 1282, 1260, 1234, 1208, 1178, 1148, 1100, 1018, 958, 936, 865, 809, 731.

iv. Radical coupling products of organyl bromides and characterization data

General Procedure for synthesis of radical coupling products

As stated before, **2-Na** (or **2-Mg**) was generated *in situ* from $^{Cbz}[^{tBu}PNP]Pt^{II}Cl$ (50.0 or 100 mg) in THF. Organyl bromide (2.00 eq for clean reactions to **1-Br** or 1.00 eq for reactions yielding mixtures of **1-Br** and **4a/b/c/d**) was added and the J. Young Tube was shaken vigorously, causing a color change from orange-red to yellow and the formation of a precipitate. The crude solution was filtrated by syringe and the coupling products **5-A** to **5-D** were isolated by flash column chromatography (*n*-hexane:DCM = 3:1) and **5-E** to **5-H** by distillation under reduced pressure. The NMR data given below matches the literature report.[3-8] When **2-Mg** is used, minor impurities of **1-Cl** can be formed during the radical coupling reactions.

Synthesis of 1-(Diphenylmethylene)-4-trityl-2,5-cyclohexadiene (5-A')

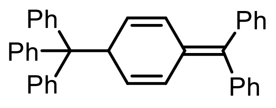

^{1}H NMR (C_6D_6, 600 MHz, 295K): δ [ppm] = 7.32-7.28 (m, 5H, H^{arom}), 7.11-6.95 (m, 20H, H^{arom}), 6.45 (dd, $^3J_{H\text{-}H}$ = 10.5 Hz, $^4J_{H\text{-}H}$ = 1.8 Hz, 2H, H^{olef}), 5.93 (dd, $^3J_{H\text{-}H}$ = 10.5 Hz, $^3J_{HH}$ = 3.7 Hz, 2H, H^{olef}), 4.93 (s, 1H, H^{aliph}). **HR-MS** (EI): calc. for $[C_{38}H_{30}]^+$: 486.2342, found m/z: 486.2347 $[M]^+$ and m/z: 243.1208 $[Ph_3C]^{\bullet+}$.

Synthesis of Bis(triphenylmethyl) peroxide (5-A)

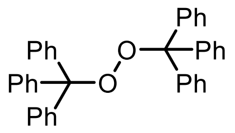

^{1}H NMR (CD_2Cl_2, 600 MHz, 295K): δ [ppm] = 7.25-7.21 (m, 18H, H^{arom}), 7.17-7.14 (m, 12H, H^{arom}). **$^{13}C\{^1H\}$ NMR** (CD_2Cl_2, 151 MHz, 295K): δ [ppm] = 143.3 (s, Cq, C^{arom}), 129.5 (s, CH, C^{arom}), 127.8 (s, CH, C^{arom}), 127.6 (s, CH, C^{arom}), 92.4 (s, Cq, C^{aliph}). **HR-MS** (EI): calc. for $[C_{38}H_{30}O_2]^+$: 518.2240, found m/z: 259.1097 $[Ph_3CO]^{\bullet+}$.

Synthesis of 1,1,2,2-Tetraphenylethane (5-B)

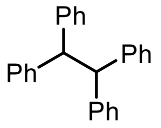

^{1}H NMR ($CDCl_3$, 600 MHz, 295K): δ [ppm] = 7.18-7.15 (m, 8H, H^{arom}), 7.13-7.09 (m, 8H, H^{arom}), 7.04-7.00 (m, 4H, H^{arom}), 4.77 (s, 2H, H^{aliph}). **$^{13}C\{^1H\}$ NMR** ($CDCl_3$, 151 MHz, 295K): δ [ppm] = 143.6 (s, Cq, C^{arom}), 128.7 (s, CH, C^{arom}), 128.3 (s, CH, C^{arom}), 126.0 (s, CH, C^{arom}), 56.5 (s, CH, C^{aliph}). **HR-MS** (EI): calc. for $[C_{26}H_{22}]^+$: 334.1716, found m/z: 334.1684 $[M]^+$ and m/z: 167.1082 $[Ph_2CH]^{\bullet+}$.

Synthesis of 1,2-Diphenylethane (5-C)

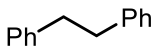

^{1}H NMR ($CDCl_3$, 600 MHz, 295K): δ [ppm] = 7.35-7.31 (m, 4H, H^{arom}), 7.26-7.22 (m, 6H, H^{arom}), 2.97 (s, 4H, H^{aliph}). **$^{13}C\{^1H\}$ NMR** ($CDCl_3$, 151 MHz, 295K): δ [ppm] = 141.9 (s, Cq, C^{arom}), 128.6 (s, CH, C^{arom}), 128.5 (s, CH, C^{arom}), 126.1 (s, CH, C^{arom}), 38.1 (s, CH_2, C^{aliph}). **HR-MS** (EI): calc. for $[C_{14}H_{14}]^+$: 182.1090, found m/z: 182.1080 $[M]^+$ and m/z: 91.0532 $[PhCH_2]^{\bullet+}$.

Synthesis of 5,6,11,12-Tetrahydrodibenzo[*a*,*e*][8]annulene (5-D)

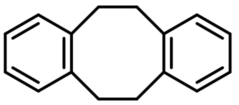

^{1}H NMR ($CDCl_3$, 600 MHz, 295K): δ [ppm] = 7.08-7.05 (m, 8H, H^{arom}), 2.80 (s, 8H, H^{aliph}). **$^{13}C\{^1H\}$ NMR** ($CDCl_3$, 151 MHz, 295K): δ [ppm] = 139.5 (s, Cq, C^{arom}), 129.3 (s, CH, C^{arom}), 126.5 (s, CH, C^{arom}), 34.3 (s, CH_2, C^{aliph}). **HR-MS** (EI): calc. for $[C_{16}H_{16}]^+$: 208.1247, found m/z: 208.1243 $[M]^+$.

Synthesis of 1,5-Hexadiene (5-E)

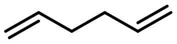

^{1}H NMR (thf-d_8, 600 MHz, 295K): δ [ppm] = 5.84-5.77 (m, 2H, H^{olef}), 5.00 (d, $^3J_{H\text{-}H}$ = 17.2 Hz, 2H, H^{olef}), 4.92 (d, $^3J_{H\text{-}H}$ = 10.5 Hz, 2H, H^{olef}), 2.13-2.12 (m, 4H, H^{aliph}). **^{13}C{^{1}H} NMR** (thf-d_8, 151 MHz, 295K): δ [ppm] = 138.8 (s, CH, C^{olef}), 114.8 (s, CH, C^{olef}), 33.9 (s, CH_2, C^{aliph}). **HR-MS** (GC): calc. for $[C_6H_{10}]^+$: 82.0777, found m/z: 81.0687 $[M\text{-}H]^+$.

Synthesis of 1,7-Octadiene (5-F)

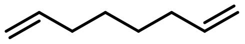

^{1}H NMR (thf-d_8, 600 MHz, 295K): δ [ppm] = 5.82-5.75 (m, 2H, H^{olef}), 4.99-4.96 (m, 2H, H^{olef}), 4.91-4.89 (m, 2H, H^{olef}), 2.06-2.03 (m, 4H, H^{aliph}), 1.41-1.39 (m, 4H, H^{aliph}). **^{13}C{^{1}H} NMR** (thf-d_8, 151 MHz, 295K): δ [ppm] = 139.4 (s, CH, C^{olef}), 114.5 (s, CH, C^{olef}), 34.4 (s, CH_2, C^{aliph}), 29.2 (s, CH_2, C^{aliph}). **HR-MS** (GC): calc. for $[C_8H_{14}]^+$: 110.1090, found m/z: 109.1066 $[M\text{-}H]^+$.

Synthesis of 1,2-Dicyclopentylethane (5-G)

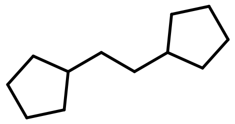

^{1}H NMR (thf-d_8, 600 MHz, 295K): δ [ppm] = 2.25-2.22 (m, 2H, H^{aliph}), 1.91-1.84 (m, 4H, H^{aliph}), 1.66-1.59 (m, 4H, H^{aliph}), 1.55-1.49 (m, 4H, H^{aliph}), 1.10-1.03 (m, 4H, H^{aliph}), 0.97 (d, $^3J_{H\text{-}H}$ = 6.7 Hz, 4H, H^{aliph}). **^{13}C{^{1}H} NMR** (thf-d_8, 151 MHz, 295K): δ [ppm] = 35.3 (s, CH_2, C^{aliph}), 33.4 (s, CH, C^{aliph}), 25.8 (s, CH_2, C^{aliph}), 20.8 (s, CH_2, C^{aliph}). **HR-MS** (GC): calc. for $[C_{12}H_{22}]^+$: 166.1716, found m/z: 166.1986 $[M]^+$.

*coupling product **5-G** was not obtained selectively and could not be isolated as a pure substance by distillation. However, the signals could be assigned to a certain extent using 2D spectra.

Synthesis of *n*-Octane (5-H)

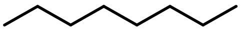

^{1}H NMR (thf-d_8, 600 MHz, 295K): δ [ppm] = 1.34-1.24 (m, 12H, H^{aliph}), 0.89 (t, $^3J_{H\text{-}H}$ = 6.9 Hz, 6H, H^{aliph}). **^{13}C{^{1}H} NMR** (thf-d_8, 151 MHz, 295K): δ [ppm] = 32.7 (s, CH_2, C^{aliph}), 30.1 (s, CH_2, C^{aliph}), 23.4 (s, CH_2, C^{aliph}), 14.3 (s, CH_3, C^{aliph}). **HR-MS** (GC): calc. for $[C_8H_{18}]^+$: 114.1403, found m/z: 114.1408 $[M]^+$.

v. Crossover experiments

Crossover of $PhCH_2Br$ (**S1**) and Ph_2CHBr (**S2**).

Following the general procedure, **2-Mg** was reacted with one equivalent of each of the two substrates and the resulting product mixture was analyzed using ^1H NMR spectroscopy. The triplet of $Ph_3C_2H_3$ was set as reference to determine the relative ratio. The data are summarized in Table 1-3.

Table 1: Integrals of the three coupling products taken from the ^1H NMR over eight runs.

Entry	Integral of $Ph_4C_2H_2$	Integral of $Ph_3C_2H_3$	Integral of $Ph_2C_2H_4$
1	1.0338	1.0000	1.2587
2	0.8571	1.0000	0.9712
3	0.8584	1.0000	0.9796
4	0.8776	1.0000	0.9938
5	0.8734	1.0000	0.9365
6	0.8530	1.0000	0.8964
7	0.9892	1.0000	1.0279
8	0.8177	1.0000	0.8416

Table 2: Relative equivalents with $Ph_3C_2H_3$ as reference and total sum of equivalents.

Entry	Eq. of $Ph_4C_2H_2$	Eq. of $Ph_3C_2H_3$	Eq. of $Ph_2C_2H_4$	Sum
1	0.5169	1.0000	0.3147	1.8316
2	0.4286	1.0000	0.2428	1.6714
3	0.4292	1.0000	0.2449	1.6741
4	0.4388	1.0000	0.2485	1.6873
5	0.4367	1.0000	0.2341	1.6708
6	0.4265	1.0000	0.2241	1.6506
7	0.4946	1.0000	0.2570	1.7516
8	0.4089	1.0000	0.2104	1.6193

Table 3: Percentage distribution of the three species with mean value ⌀ and standard deviation σ.

Entry	% of $Ph_4C_2H_2$	% of $Ph_3C_2H_3$	% of $Ph_2C_2H_4$
1	28.2%	54.6%	17.2%
2	25.6%	59.8%	14.5%
3	25.6%	59.7%	14.6%
4	26.0%	59.3%	14.7%
5	26.1%	59.9%	14.0%
6	25.8%	60.6%	13.6%
7	28.2%	57.1%	14.7%
8	25.2%	61.8%	13.0%
⌀	26%	59%	15%
σ	1%	2%	1%

2.) NMR spectra

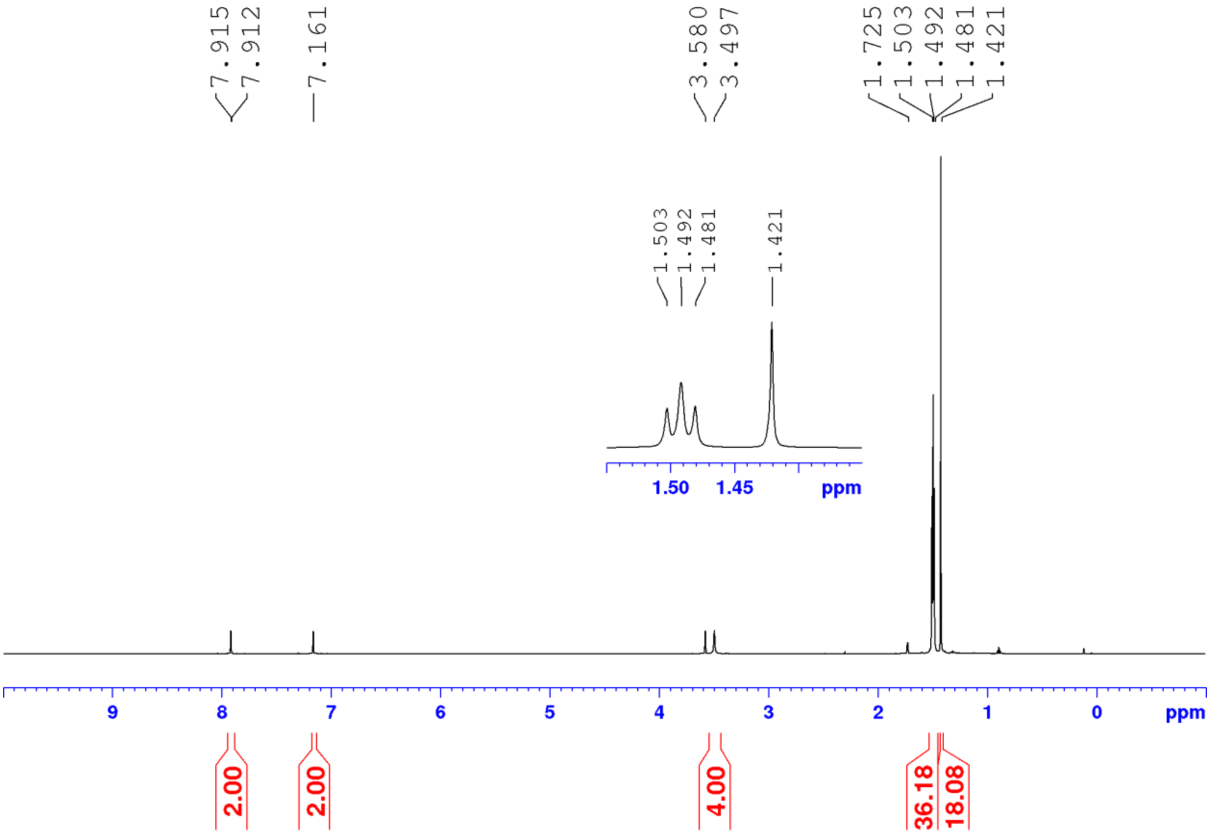

Figure 1: ^{1}H NMR (thf-d_8, 600 MHz, 295K) of ^{Cbz}[^{tBu}PNP]Pt^{II}Cl (1-Cl).

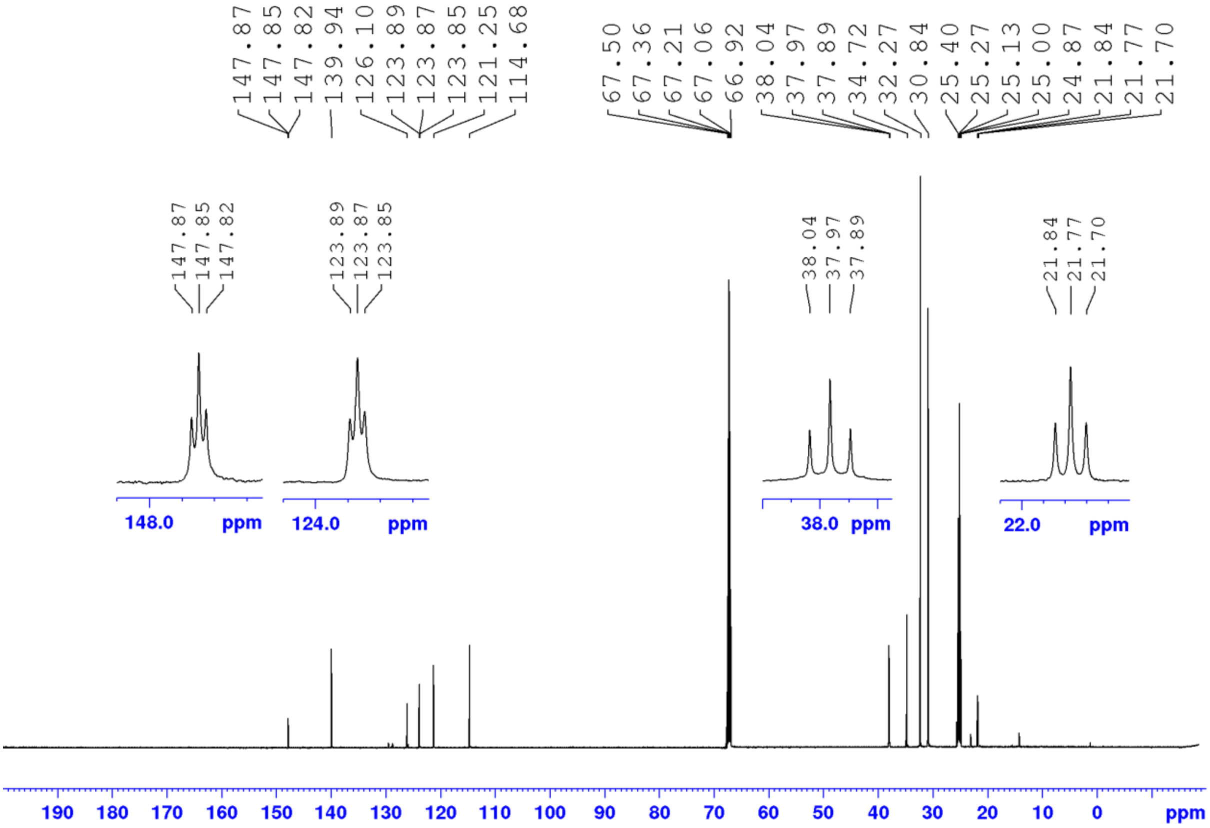

Figure 2: ^{13}C{^{1}H} NMR (thf-d_8, 151 MHz, 295K) of ^{Cbz}[^{tBu}PNP]Pt^{II}Cl (1-Cl).

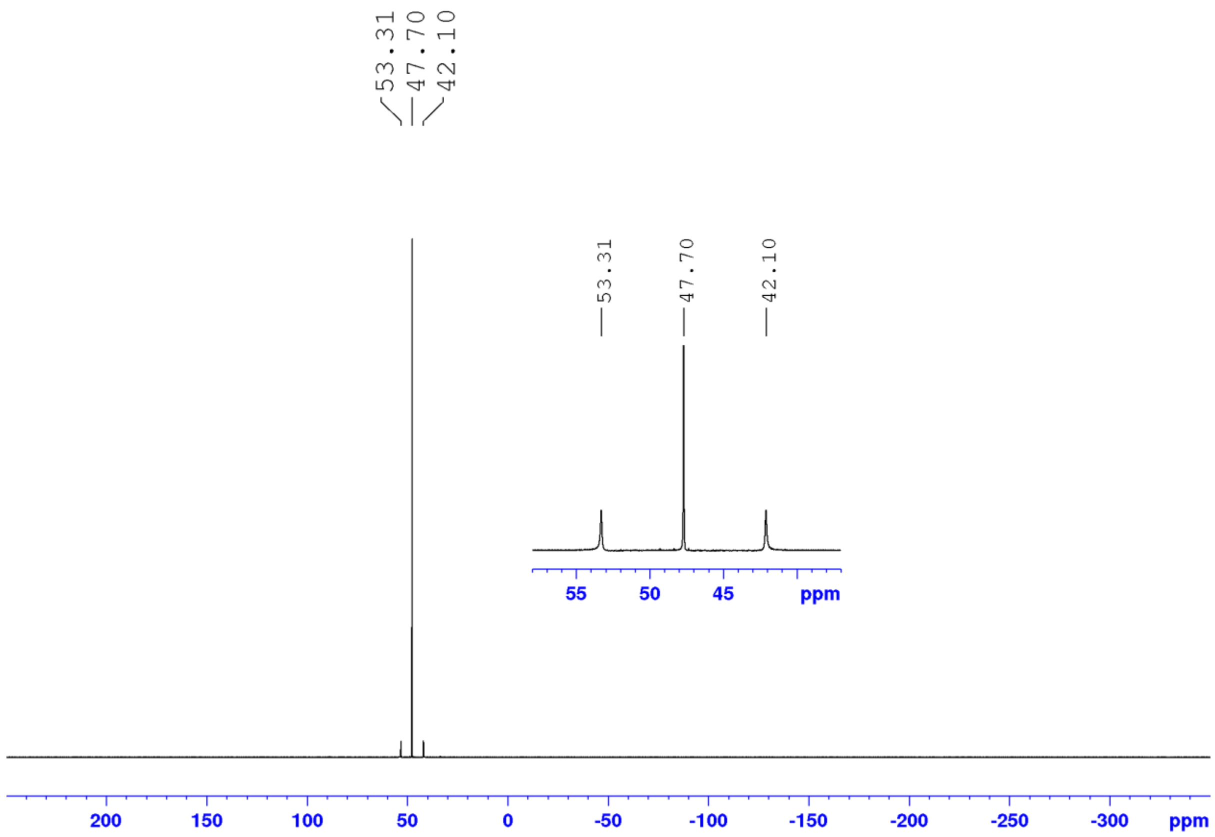

Figure 3: $^{31}P\{^{1}H\}$ NMR (thf-d_8, 243 MHz, 295K) of $^{Cbz}[^{tBu}PNP]Pt^{II}Cl$ (1-Cl).

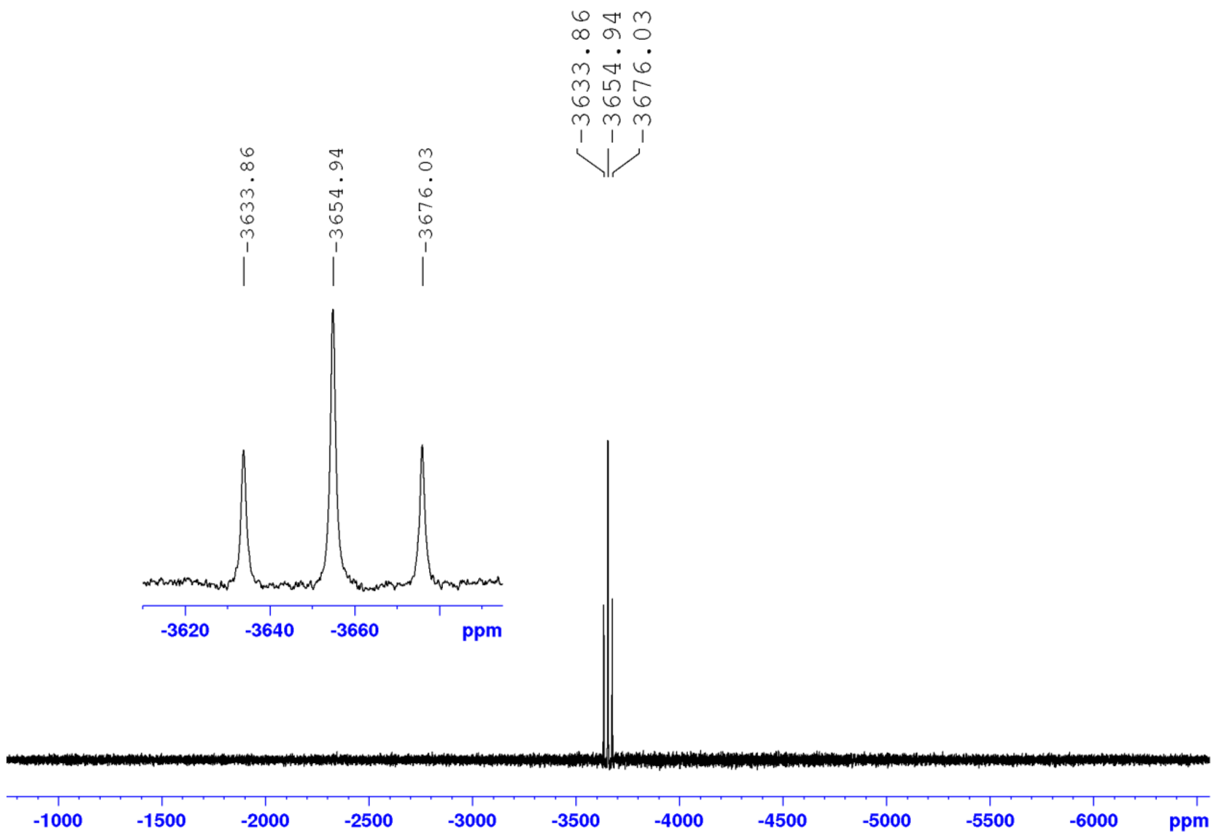

Figure 4: $^{195}Pt\{^{1}H\}$ NMR (thf-d_8, 129 MHz, 295K) of $^{Cbz}[^{tBu}PNP]Pt^{II}Cl$ (1-Cl).

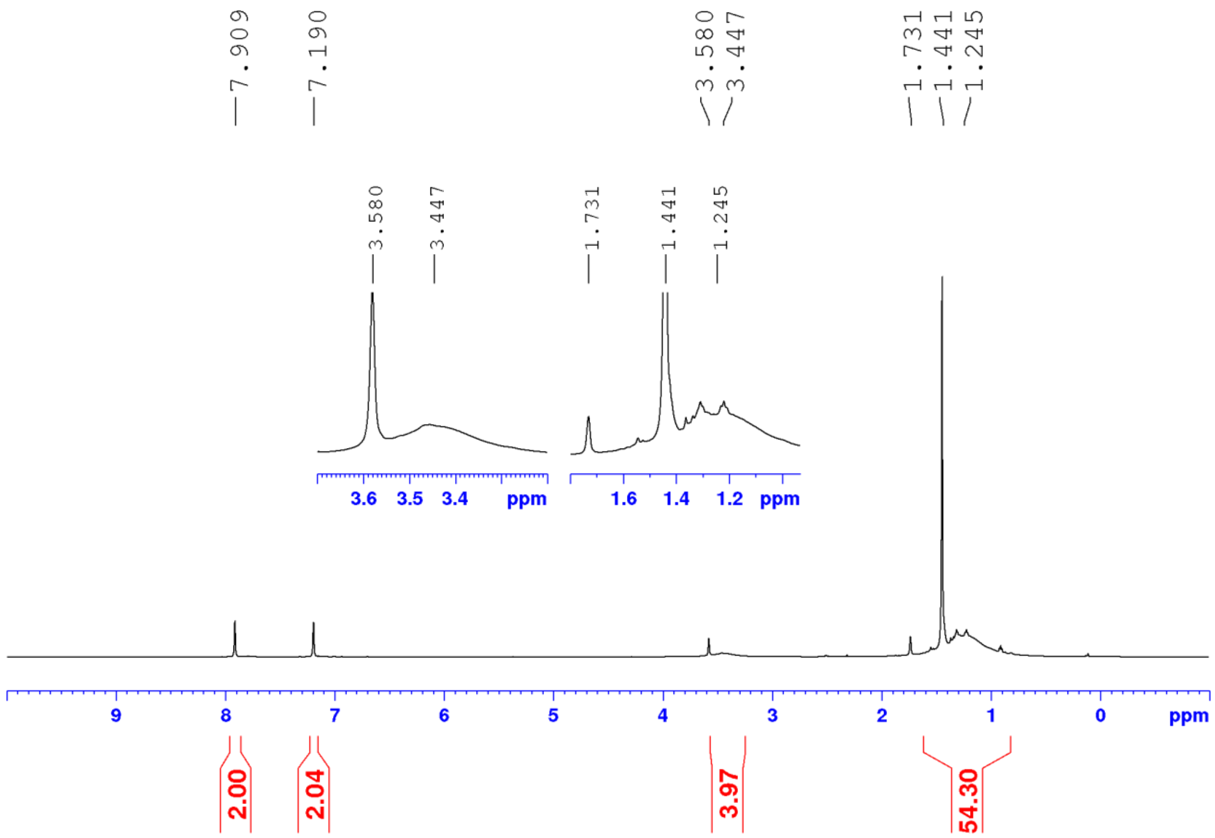

Figure 5: ^{1}H NMR (thf-d_8, 600 MHz, 295K) of $^{Cbz}[^{tBu}PNP]Pt^0Na$ (2-Na).

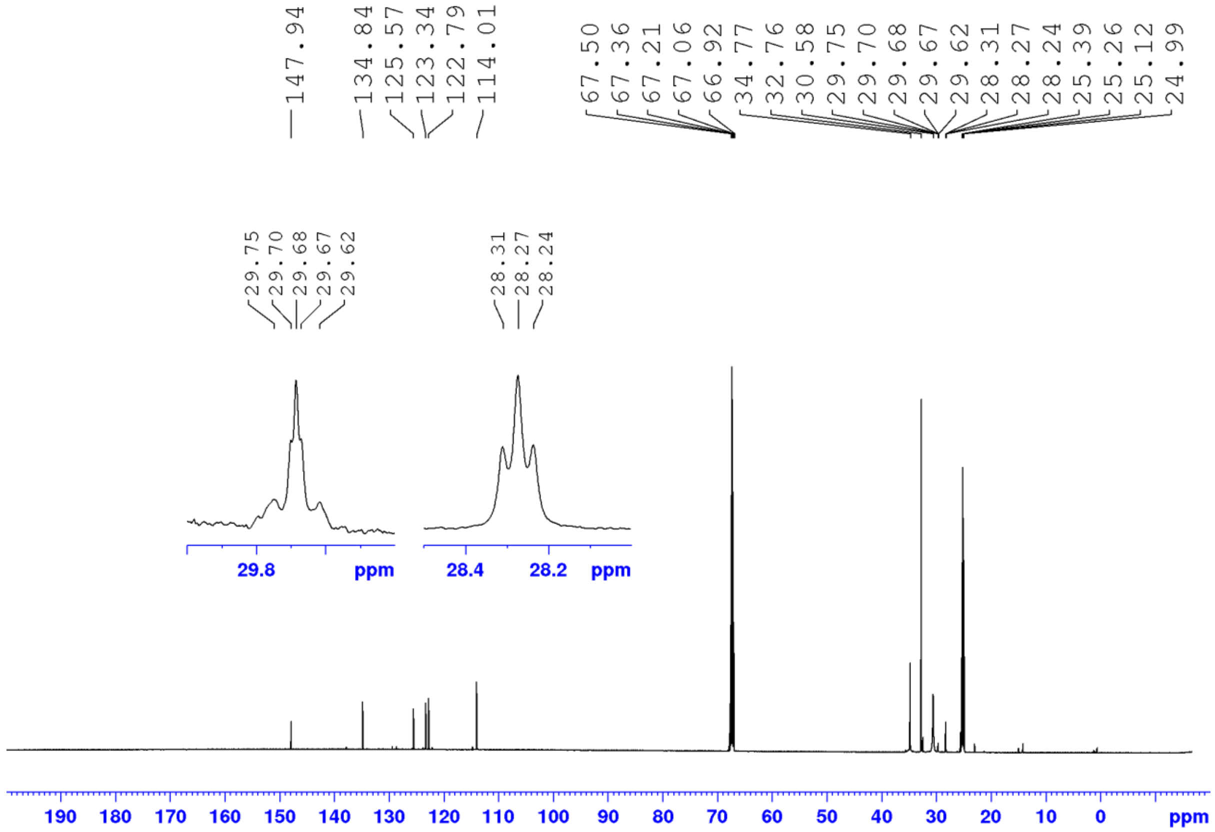

Figure 6: $^{13}C\{^{1}H\}$ NMR (thf-d_8, 151 MHz, 295K) of $^{Cbz}[^{tBu}PNP]Pt^0Na$ (2-Na).

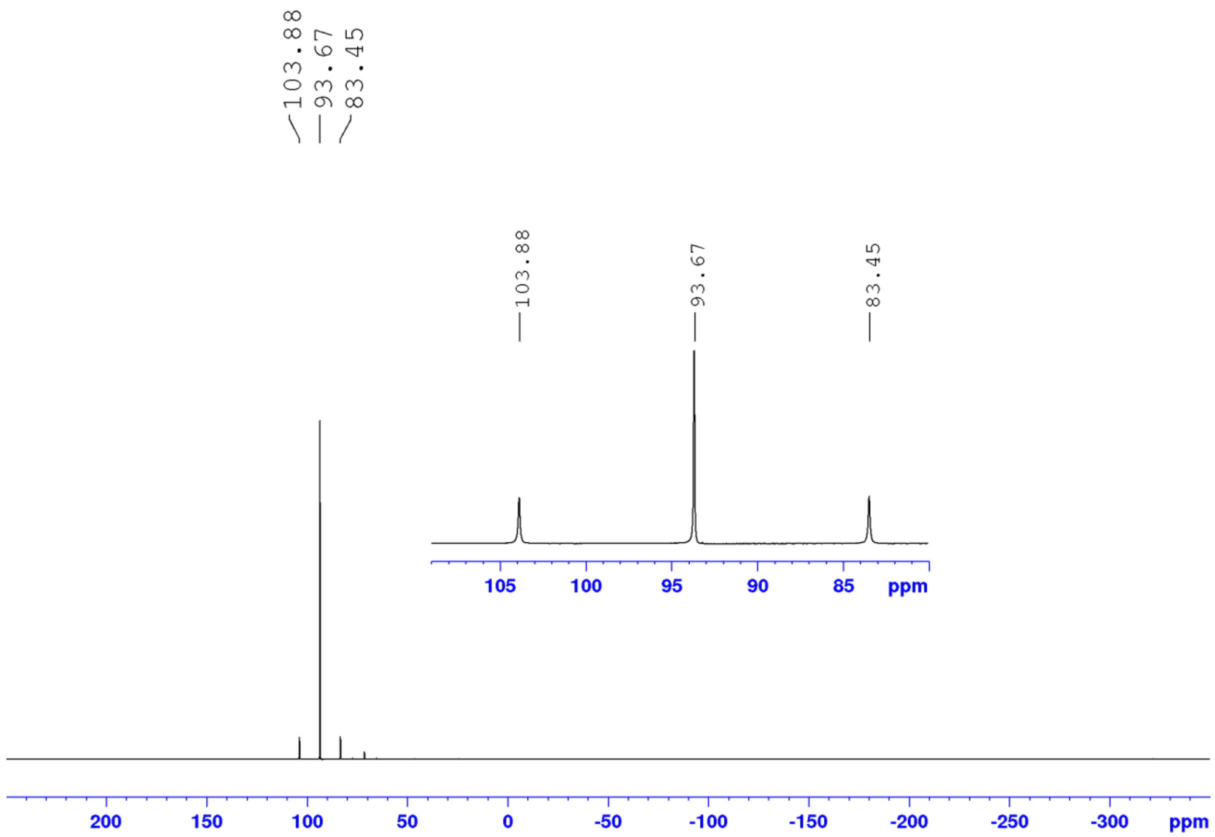

Figure 7: $^{31}P\{^{1}H\}$ NMR (thf-d_8, 243 MHz, 295K) of $^{Cbz}[^{tBu}PNP]Pt^{0}Na$ (2-Na).

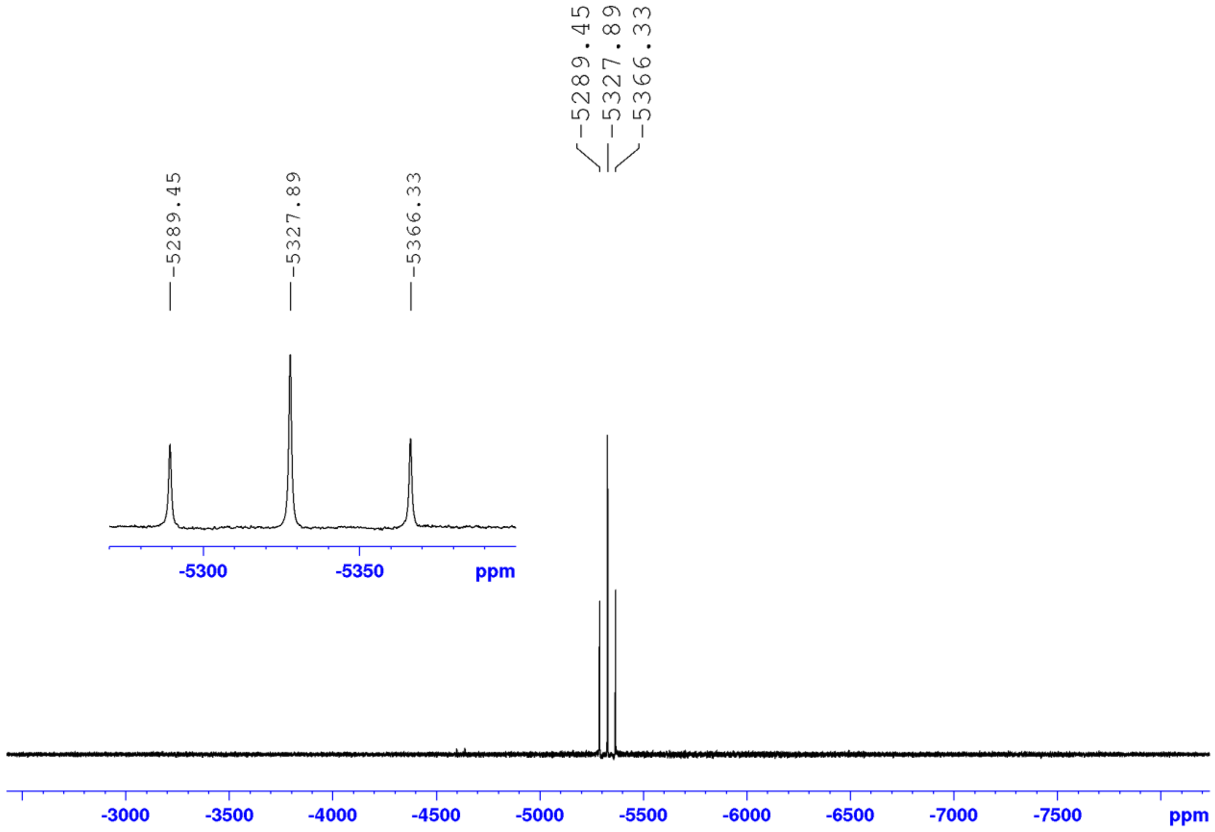

Figure 8: $^{195}Pt\{^{1}H\}$ NMR (thf-d_8, 129 MHz, 295K) of $^{Cbz}[^{tBu}PNP]Pt^{0}Na$ (2-Na).

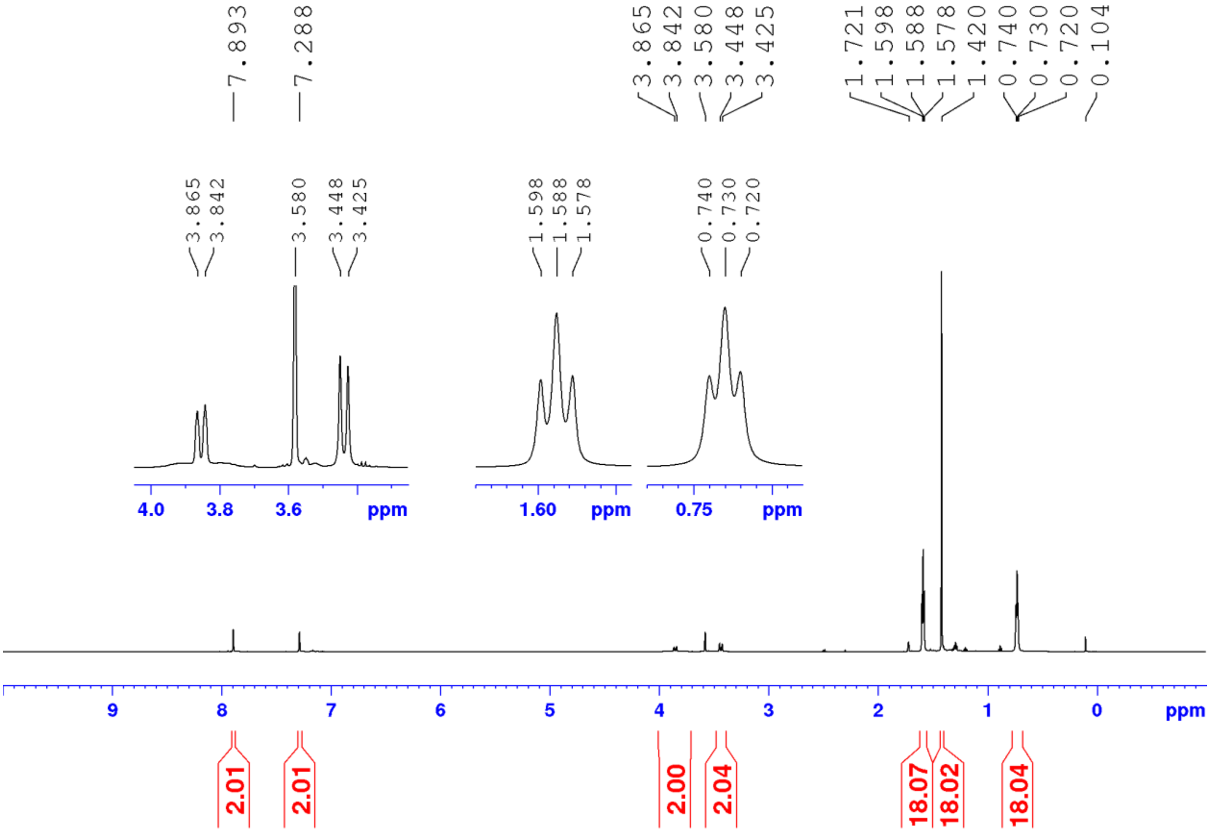

Figure 9: ^{1}H NMR (thf-d_8, 600 MHz, 295K) of ^{Cbz}[^{tBu}PNP]Pt^{0}MgCl (2-Mg).

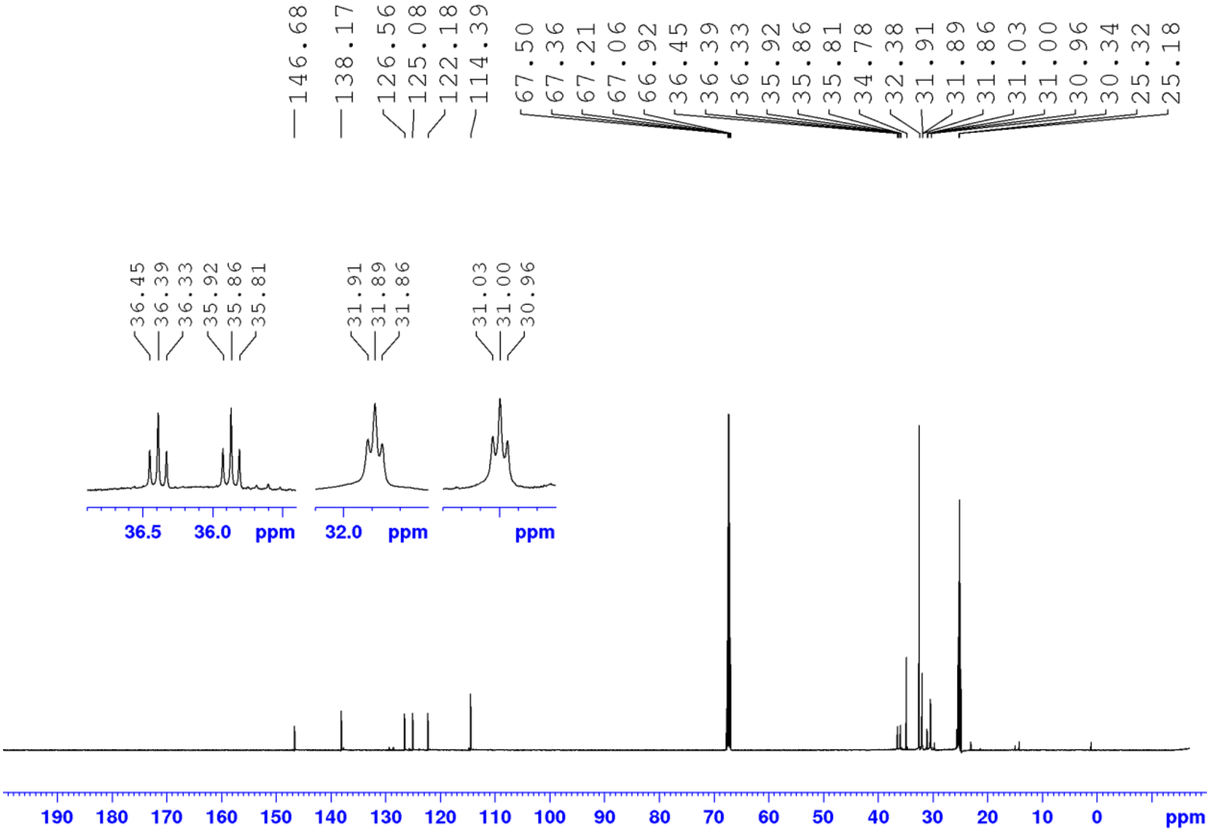

Figure 10: $^{13}C\{^{1}H\}$ NMR (thf-d_8, 151 MHz, 295K) of ^{Cbz}[^{tBu}PNP]Pt^{0}MgCl (2-Mg).

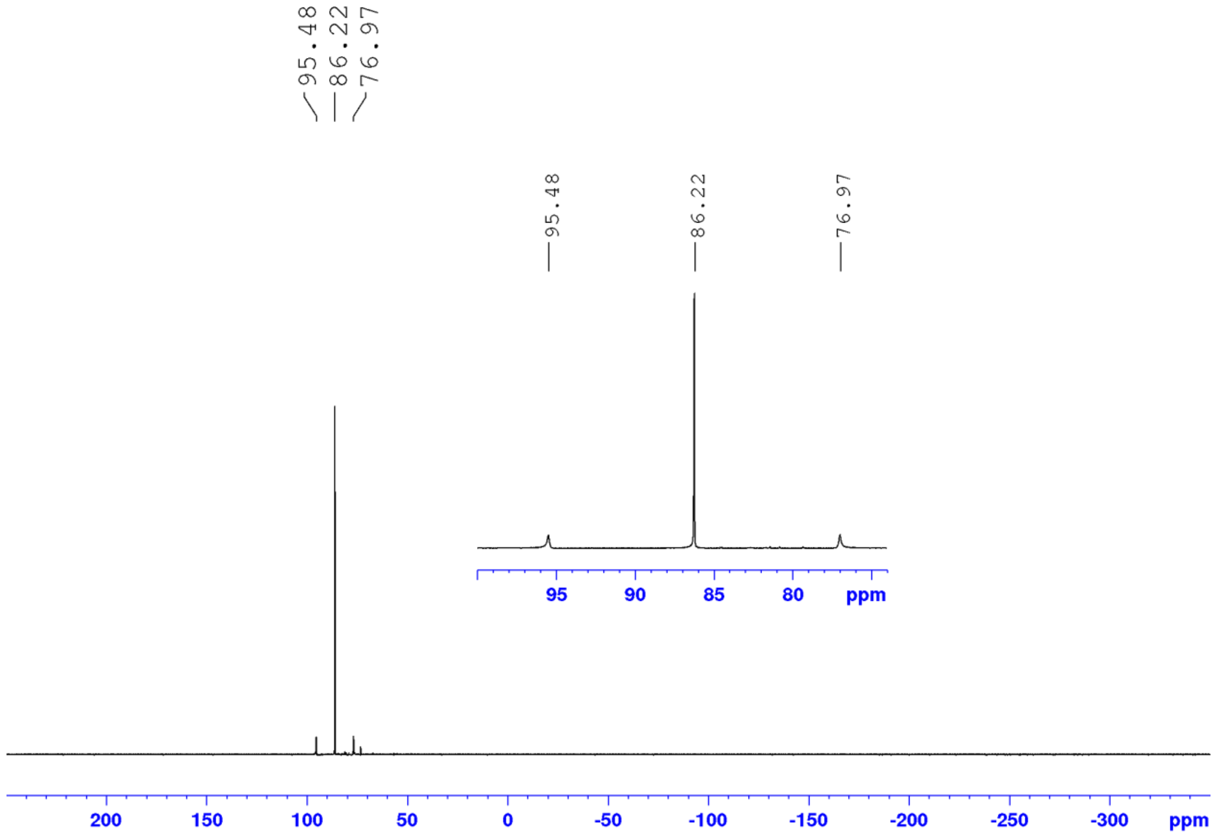

Figure 11: $^{31}P\{^{1}H\}$ NMR (thf-d_8, 243 MHz, 295K) of $^{Cbz}[^{tBu}PNP]Pt^{0}MgCl$ (2-Mg).

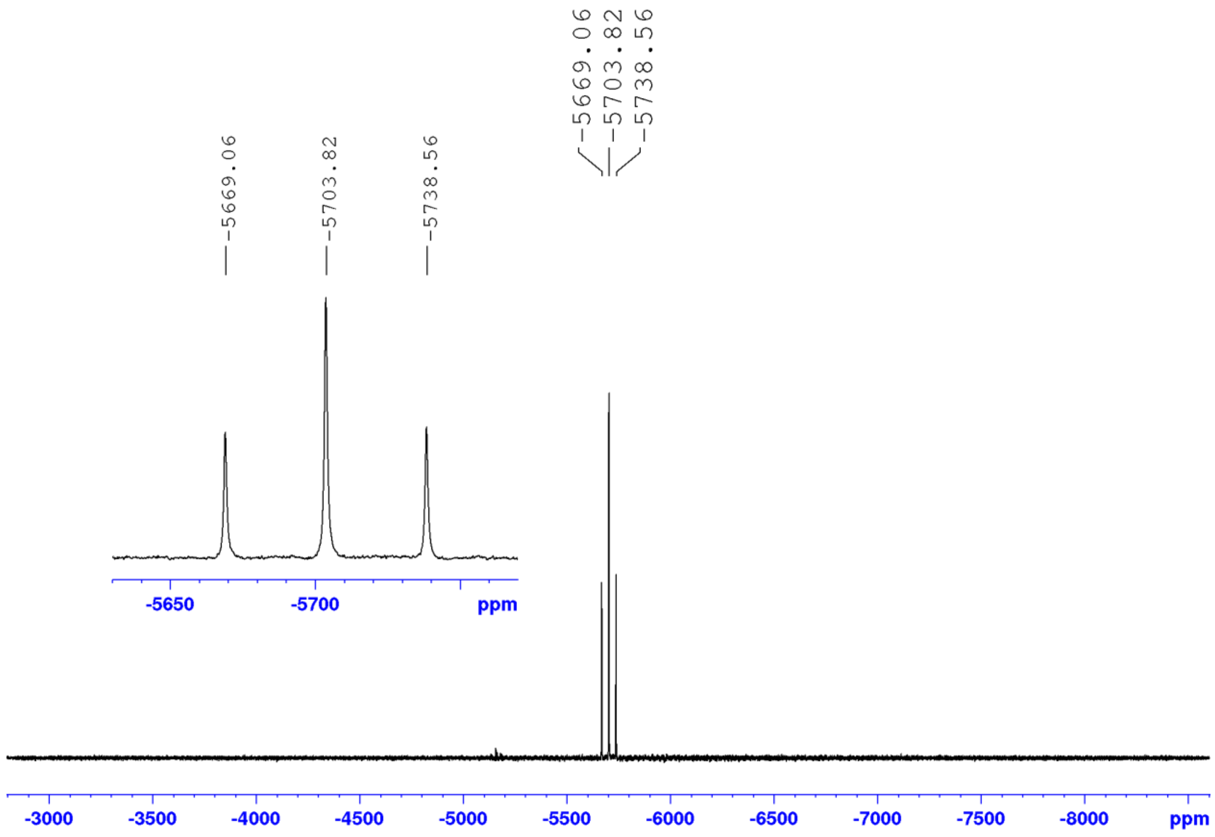

Figure 12: $^{195}Pt\{^{1}H\}$ NMR (thf-d_8, 129 MHz, 295K) of $^{Cbz}[^{tBu}PNP]Pt^{0}MgCl$ (2-Mg).

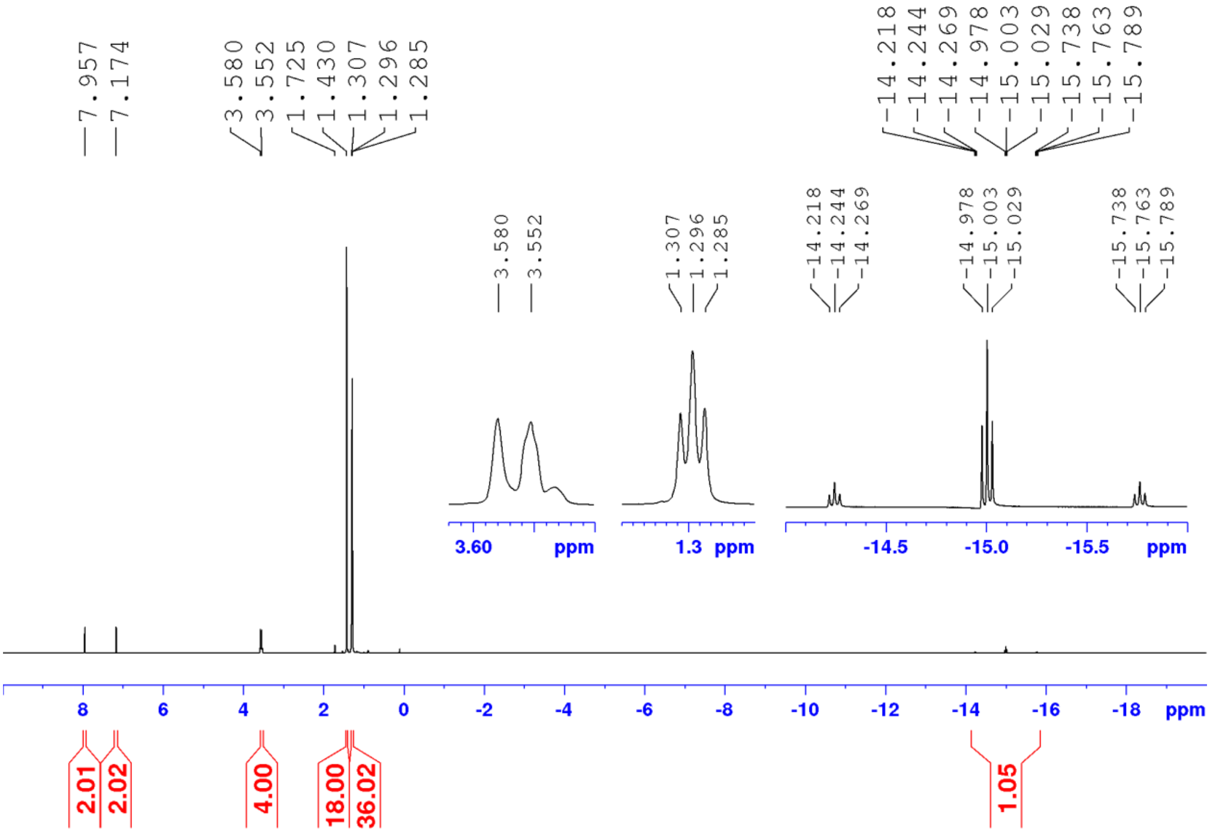

Figure 13: ^{1}H NMR (thf-d_8, 600 MHz, 295K) of ^{Cbz}[^{tBu}PNP]Pt^{II}H (3-H).

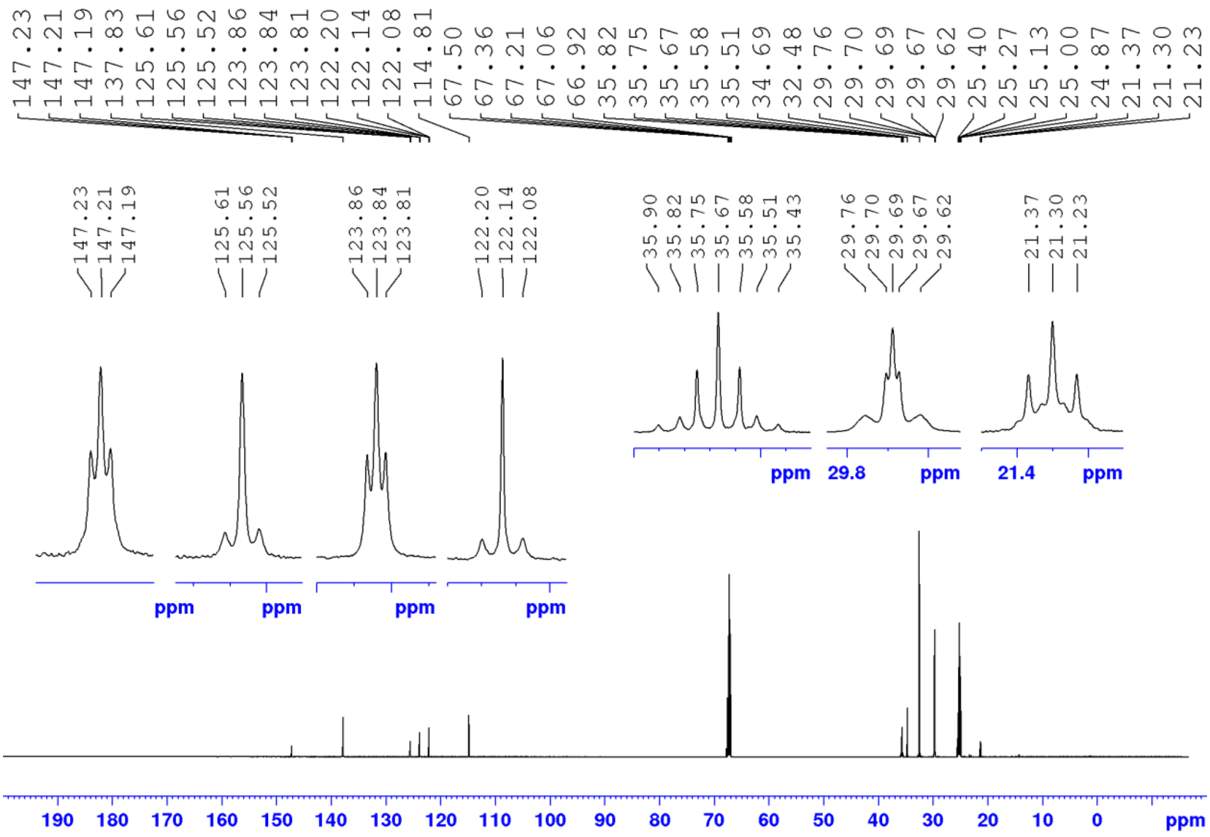

Figure 14: ^{13}C{^{1}H} NMR (thf-d_8, 151 MHz, 295K) of ^{Cbz}[^{tBu}PNP]Pt^{II}H (3-H).

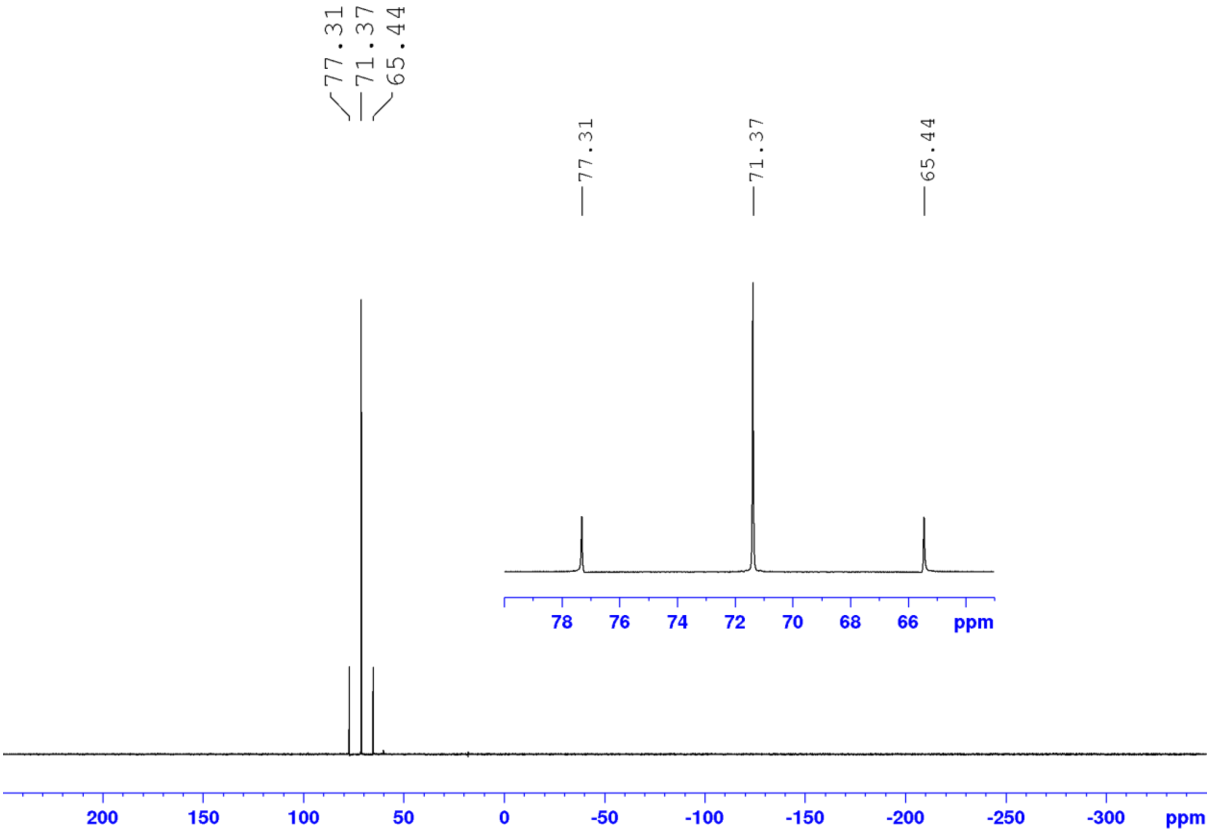

Figure 15: $^{31}P\{^{1}H\}$ NMR (thf-d$_8$, 243 MHz, 295K) of $^{Cbz}[^{tBu}PNP]Pt^{II}H$ (3-H).

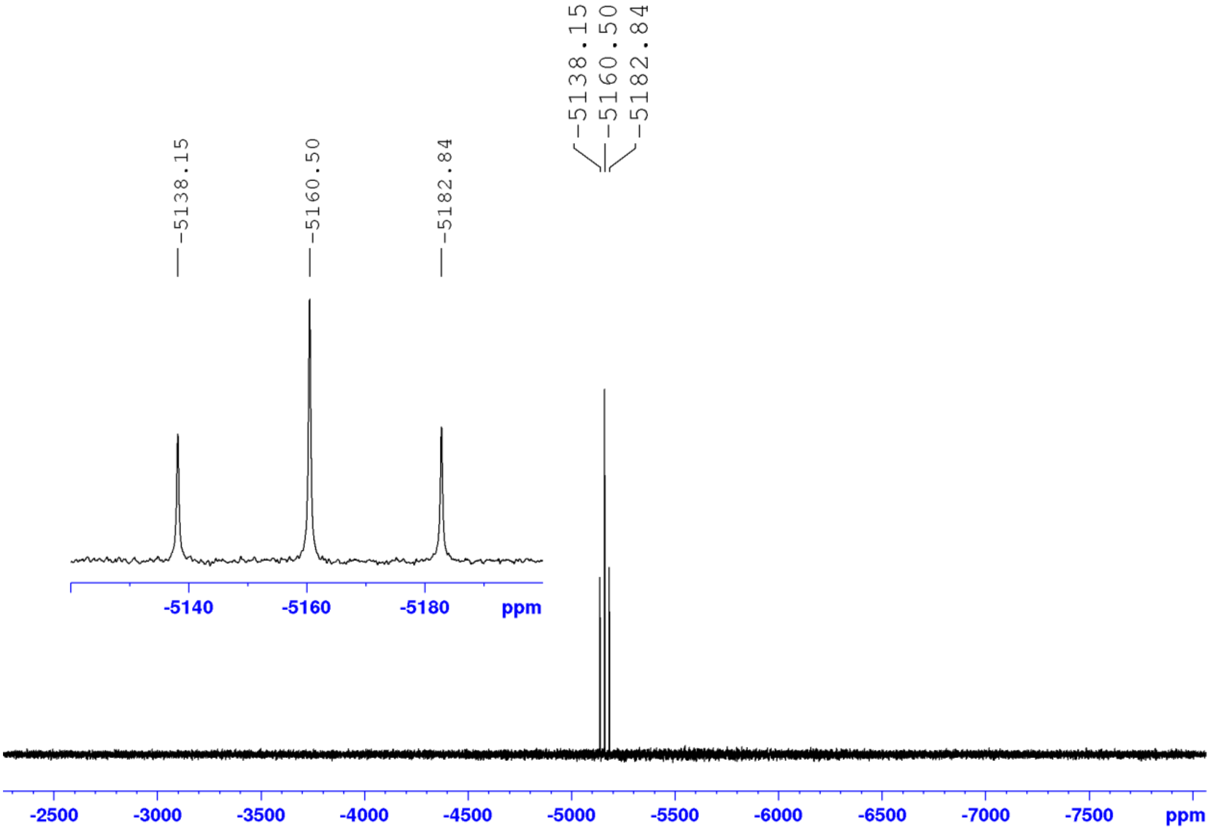

Figure 16: $^{195}Pt\{^{1}H\}$ NMR (thf-d$_8$, 129 MHz, 295K) of $^{Cbz}[^{tBu}PNP]Pt^{II}H$ (3-H).

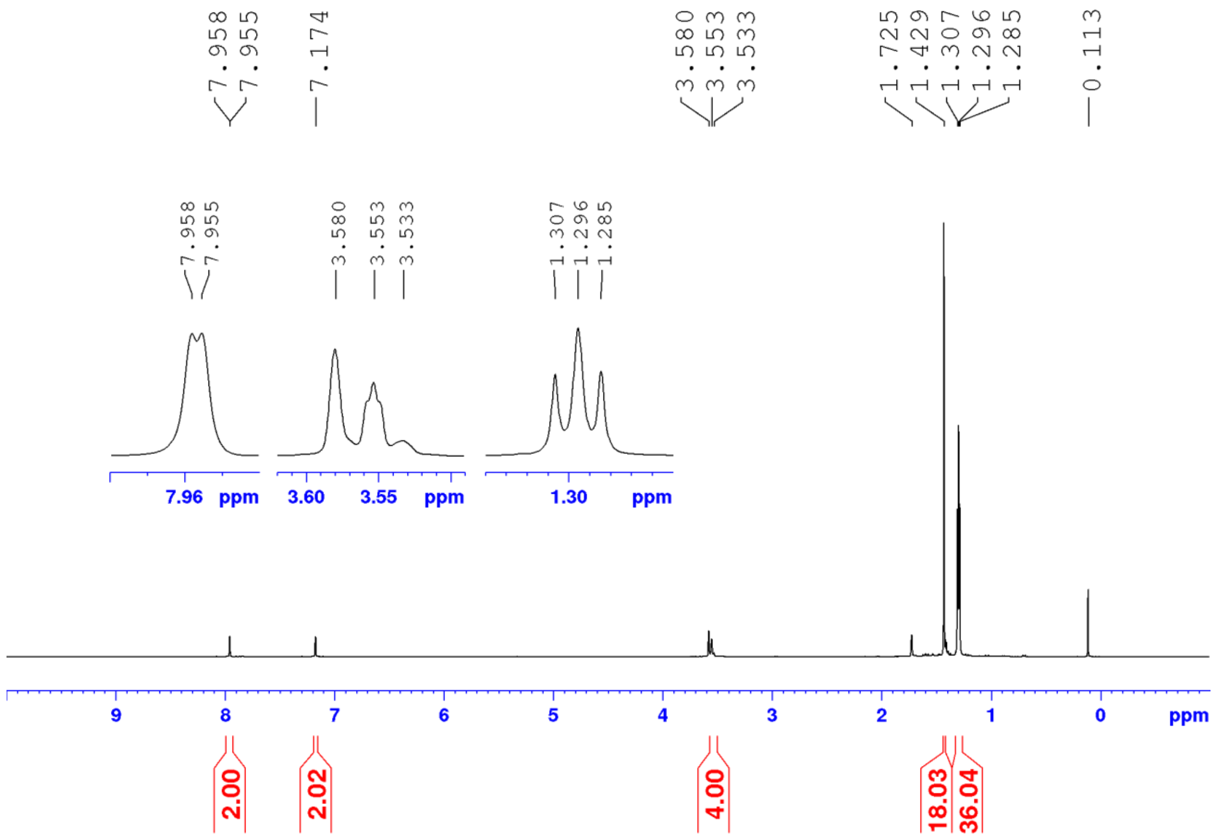

Figure 17: ^{1}H NMR (thf-d_8, 600 MHz, 295K) of $^{Cbz}[^{tBu}PNP]Pt^{II}D$ (3-D).

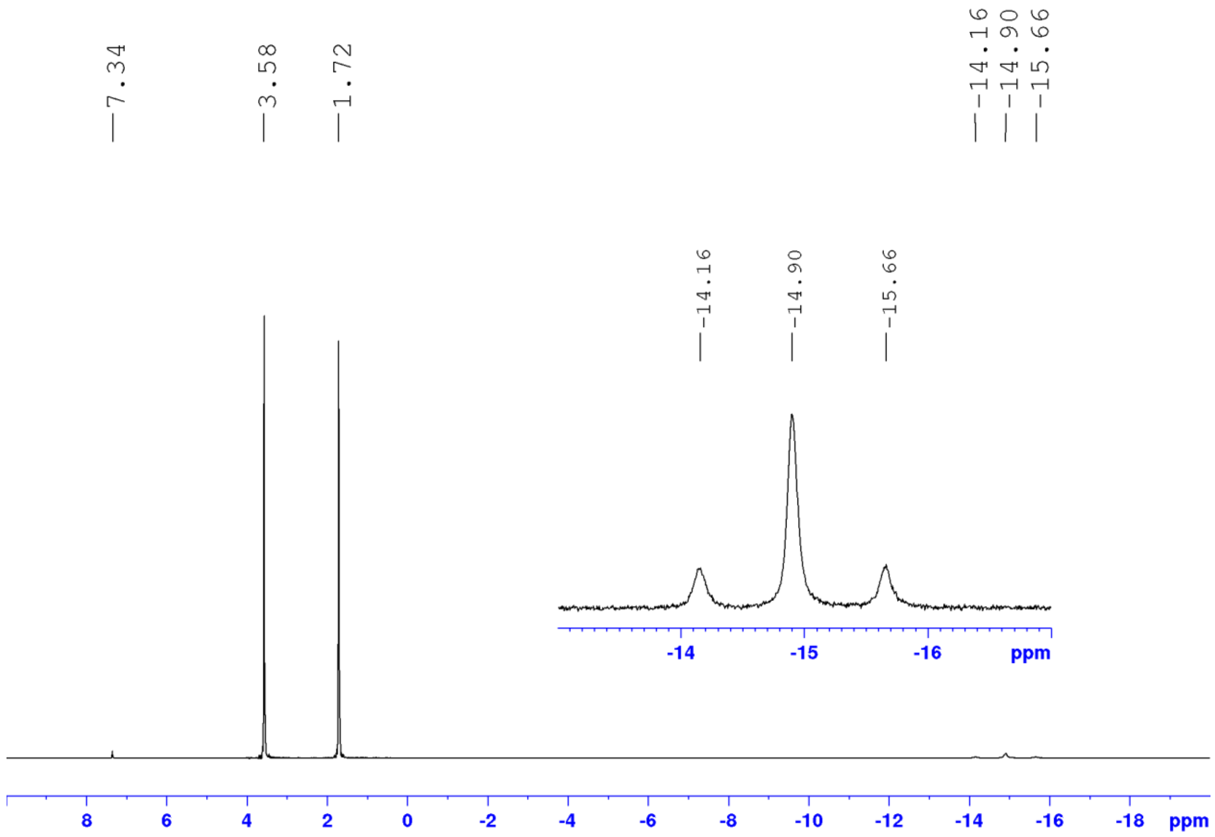

Figure 18: ^{2}H NMR (thf-d_8, 92 MHz, 295K) of $^{Cbz}[^{tBu}PNP]Pt^{II}D$ (3-D).

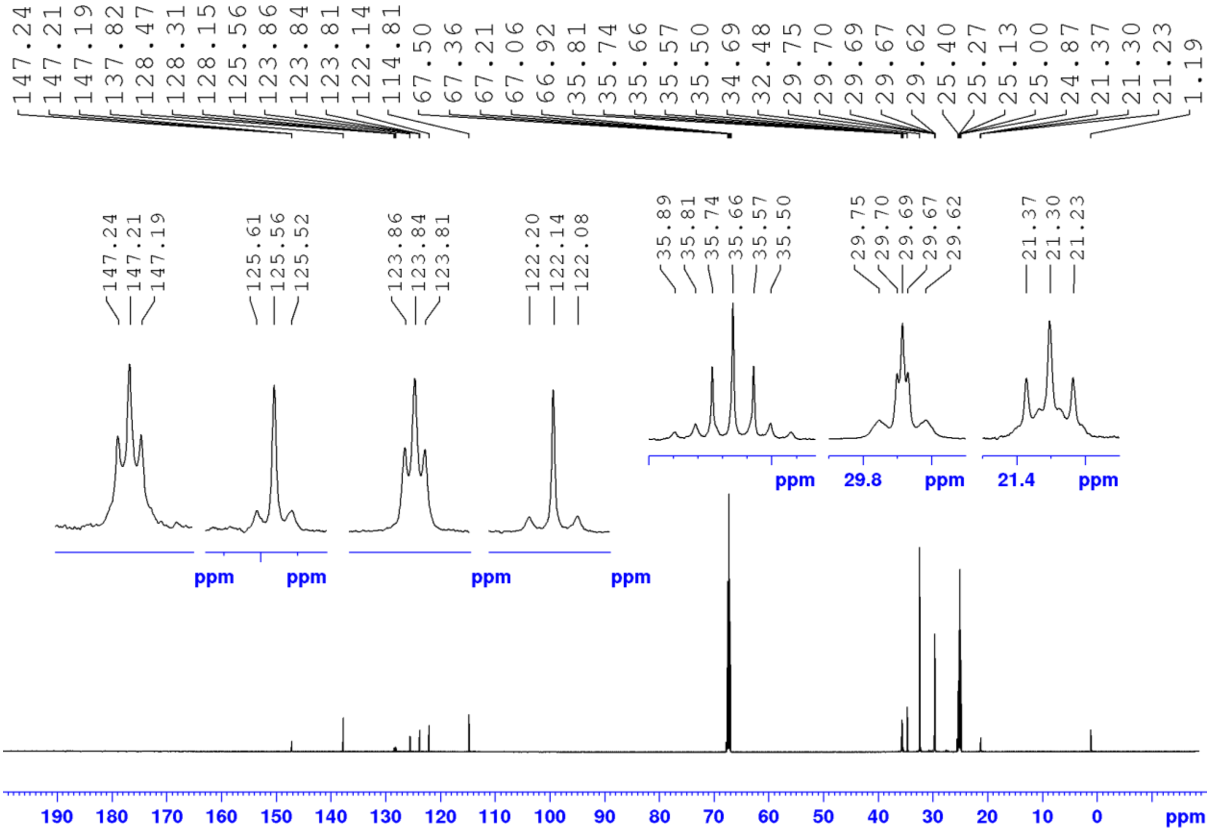

Figure 19: $^{13}C\{^1H\}$ NMR (thf-d_8, 151 MHz, 295K) of $^{Cbz}[^{tBu}PNP]Pt^{II}D$ (3-D).

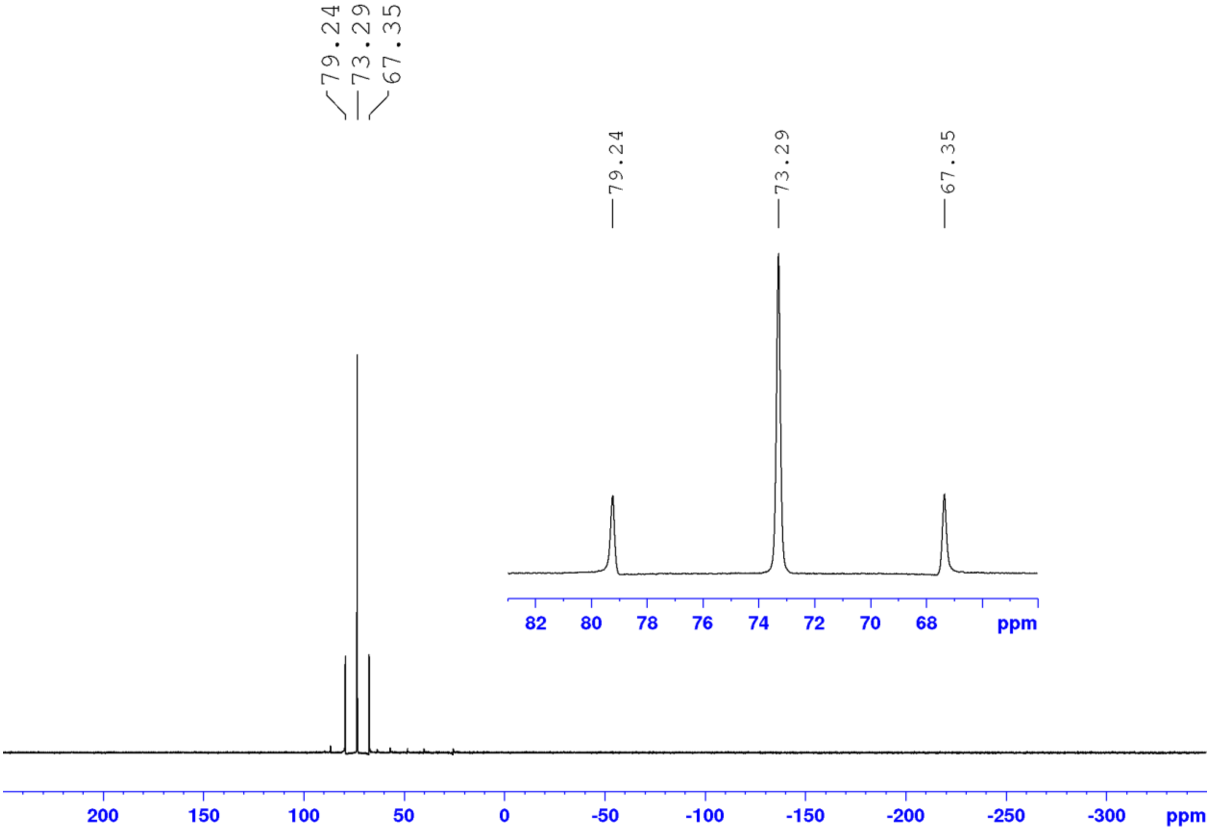

Figure 20: $^{31}P\{^1H\}$ NMR (thf-d_8, 243 MHz, 295K) of $^{Cbz}[^{tBu}PNP]Pt^{II}D$ (3-D).

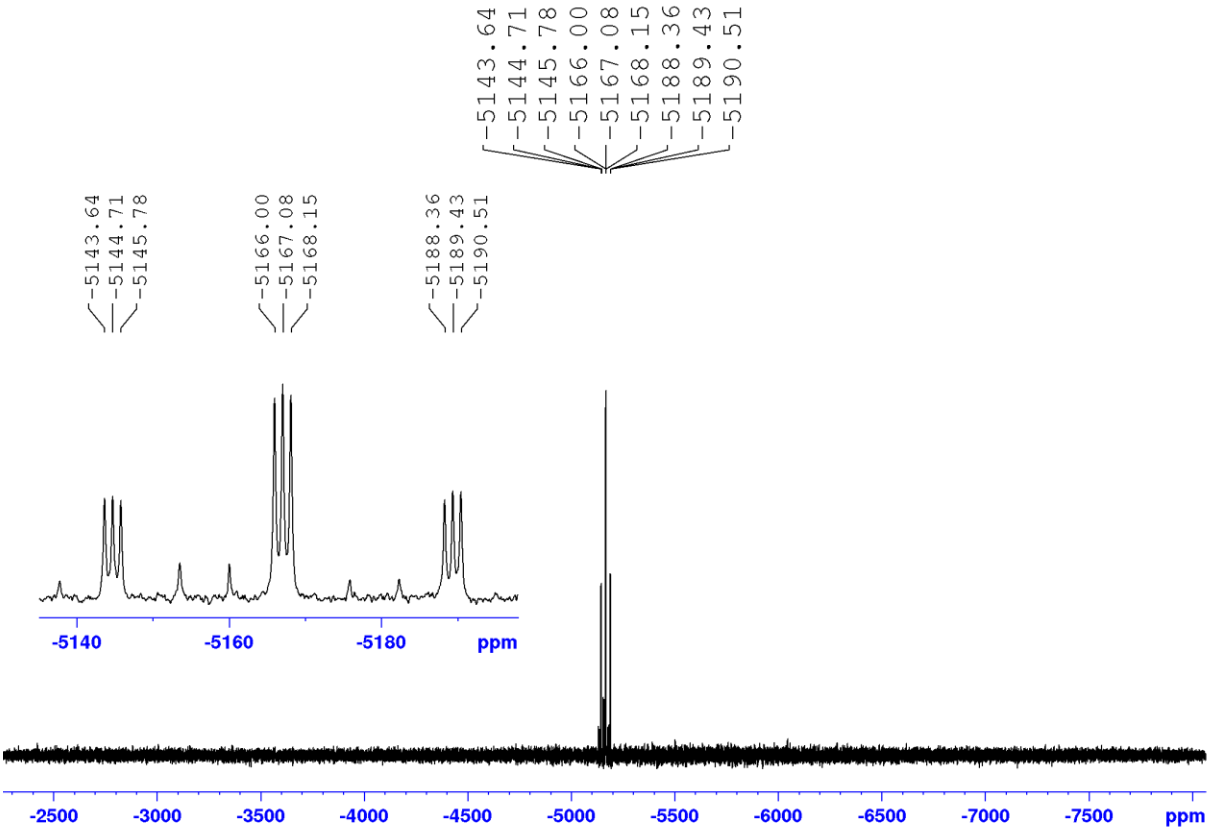

Figure 21: ^{195}Pt{^{1}H} NMR (thf-d_8, 129 MHz, 295K) of ^{Cbz}[^{tBu}PNP]Pt^{II}D (3-D).

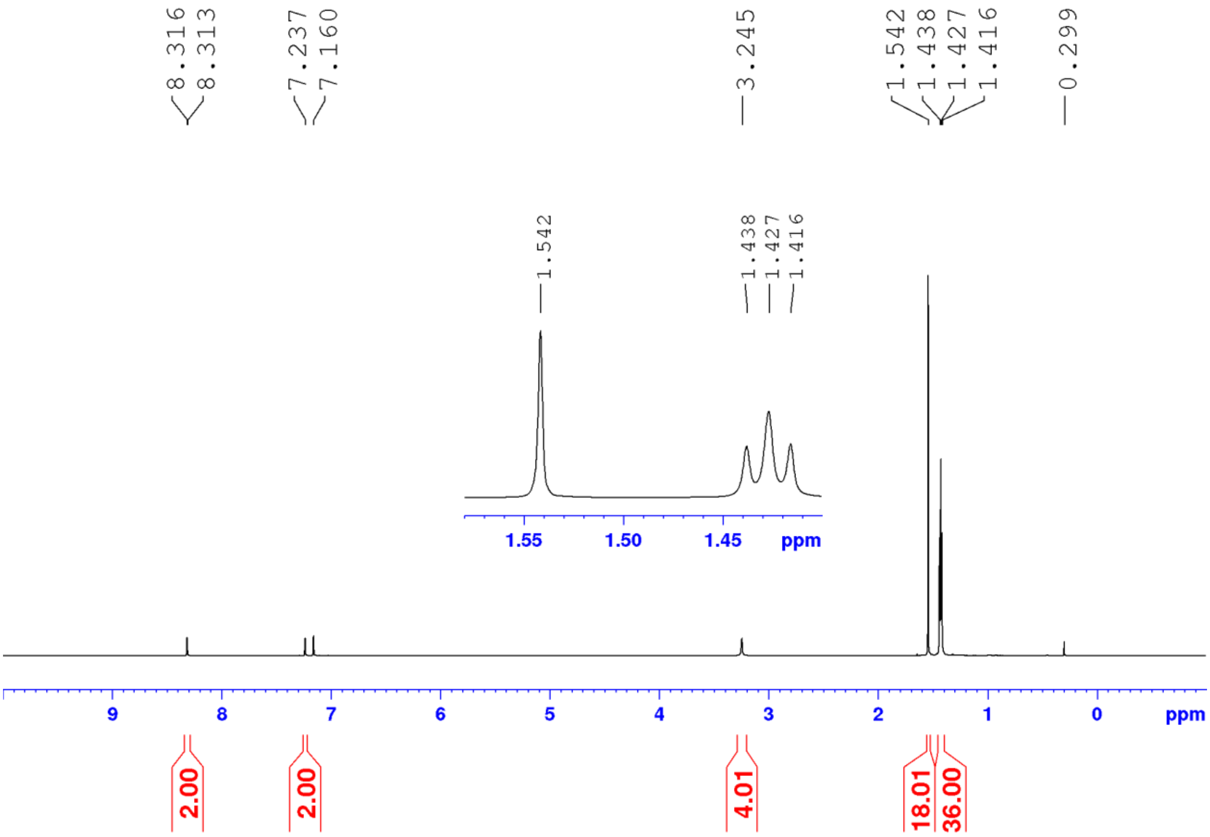

Figure 22: ^{1}H NMR (C_6D_6, 600 MHz, 295K) of ^{Cbz}[^{tBu}PNP]Pt^{II}Br (1-Br).

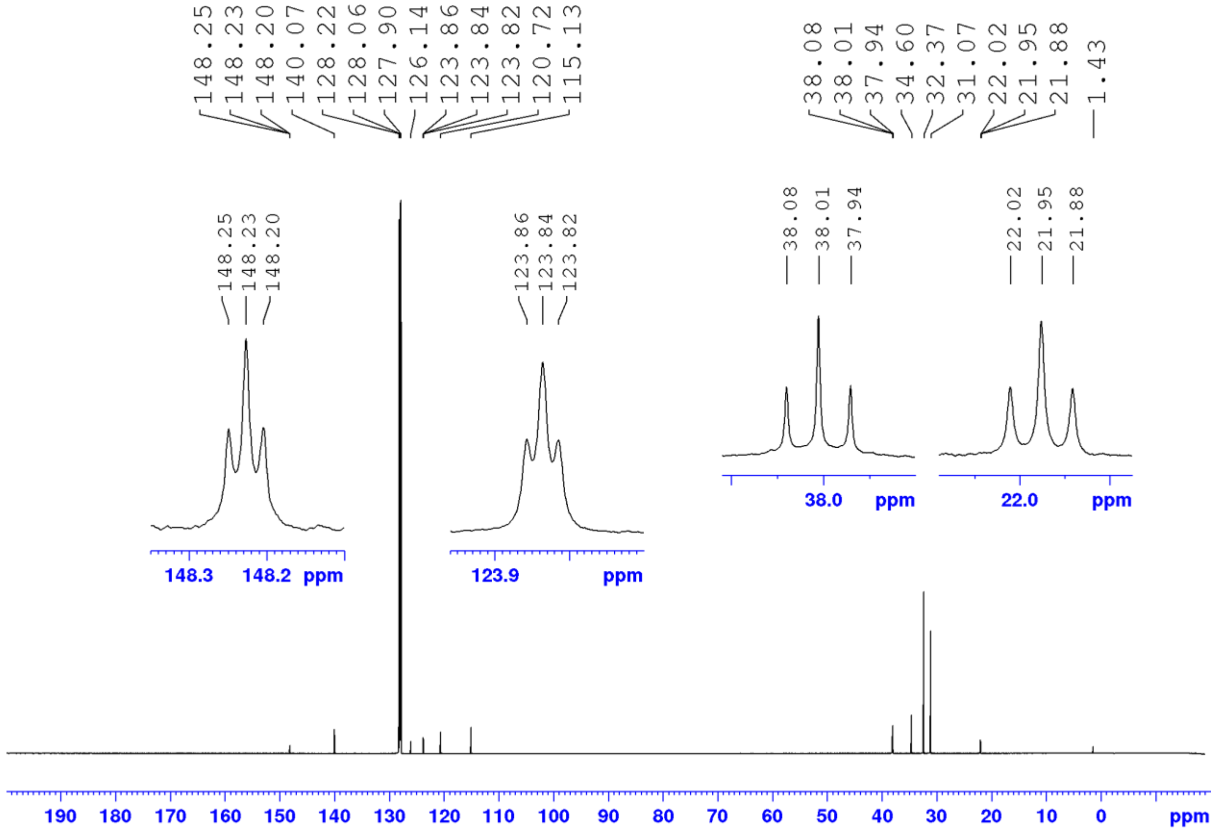

Figure 23: $^{13}C\{^1H\}$ NMR (C_6D_6, 151 MHz, 295K) of $^{Cbz}[^{tBu}PNP]Pt^{II}Br$ (1-Br).

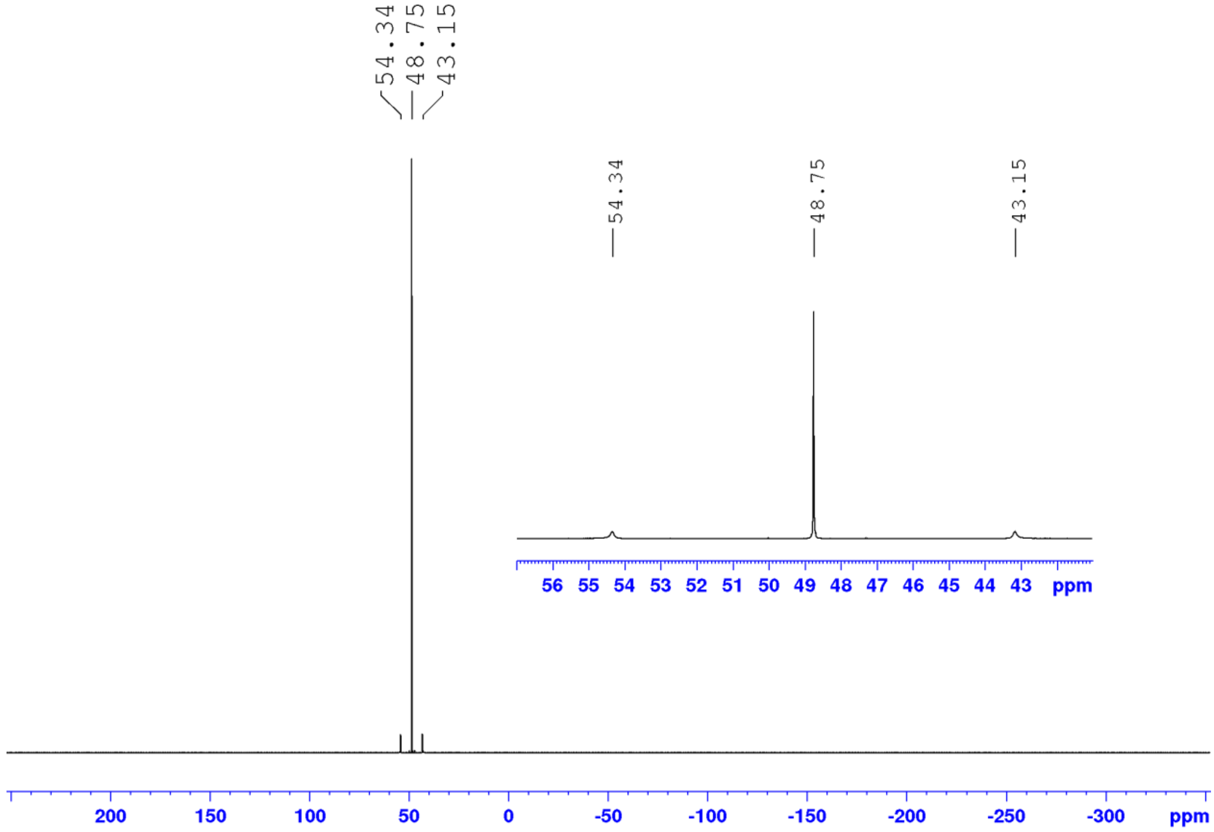

Figure 24: $^{31}P\{^1H\}$ NMR (C_6D_6, 243 MHz, 295K) of $^{Cbz}[^{tBu}PNP]Pt^{II}Br$ (1-Br).

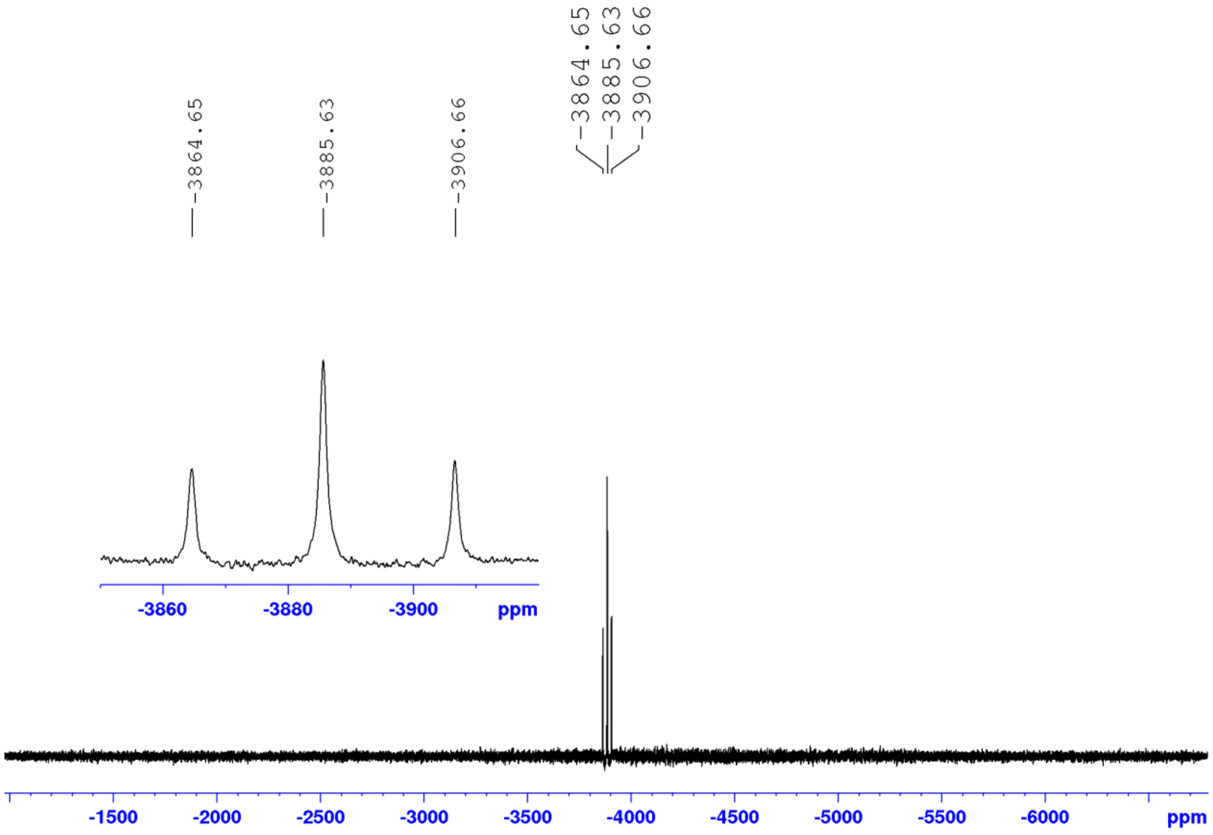

Figure 25: $^{195}Pt\{^{1}H\}$ NMR (C_6D_6, 129 MHz, 295K) of $^{Cbz}[^{tBu}PNP]Pt^{II}Br$ (1-Br).

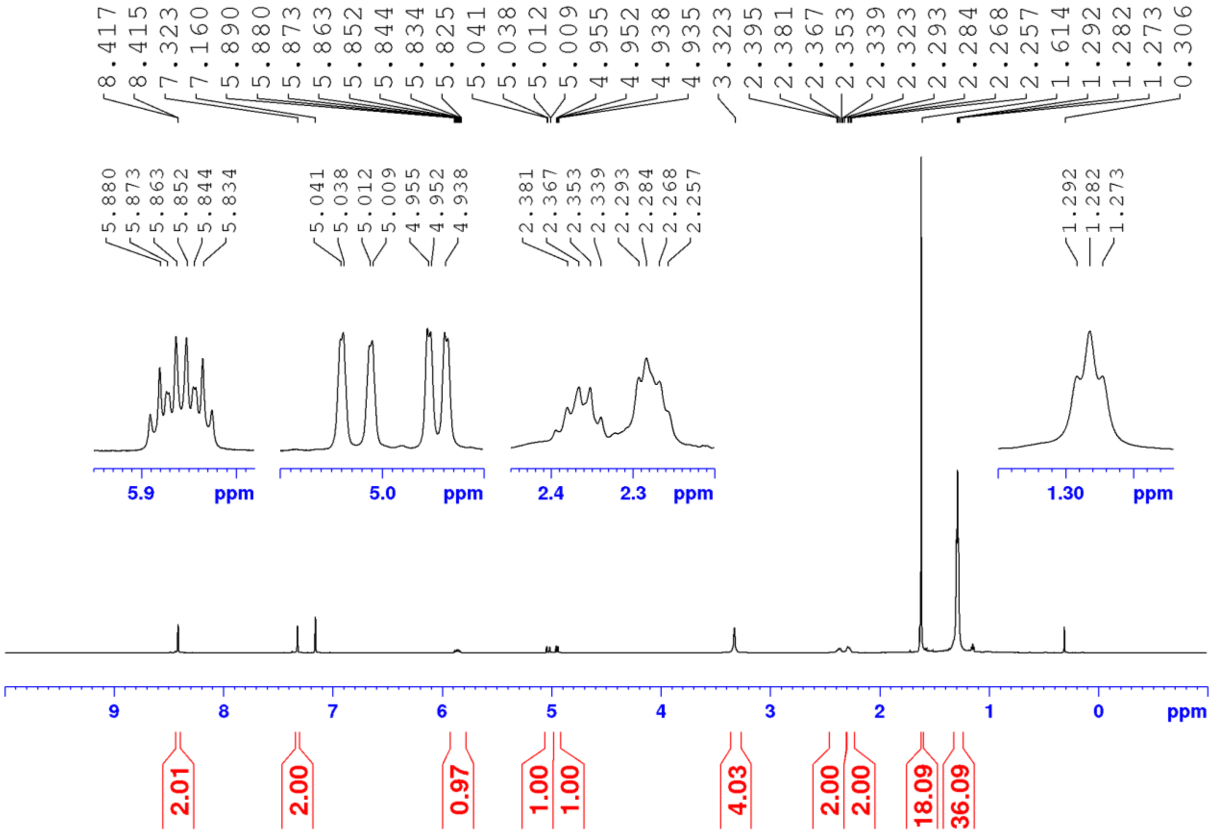

Figure 26: ^{1}H NMR (C_6D_6, 600 MHz, 295K) of $^{Cbz}[^{tBu}PNP]Pt^{II}CH_2CH_2CH{=}CH_2$ (4a).

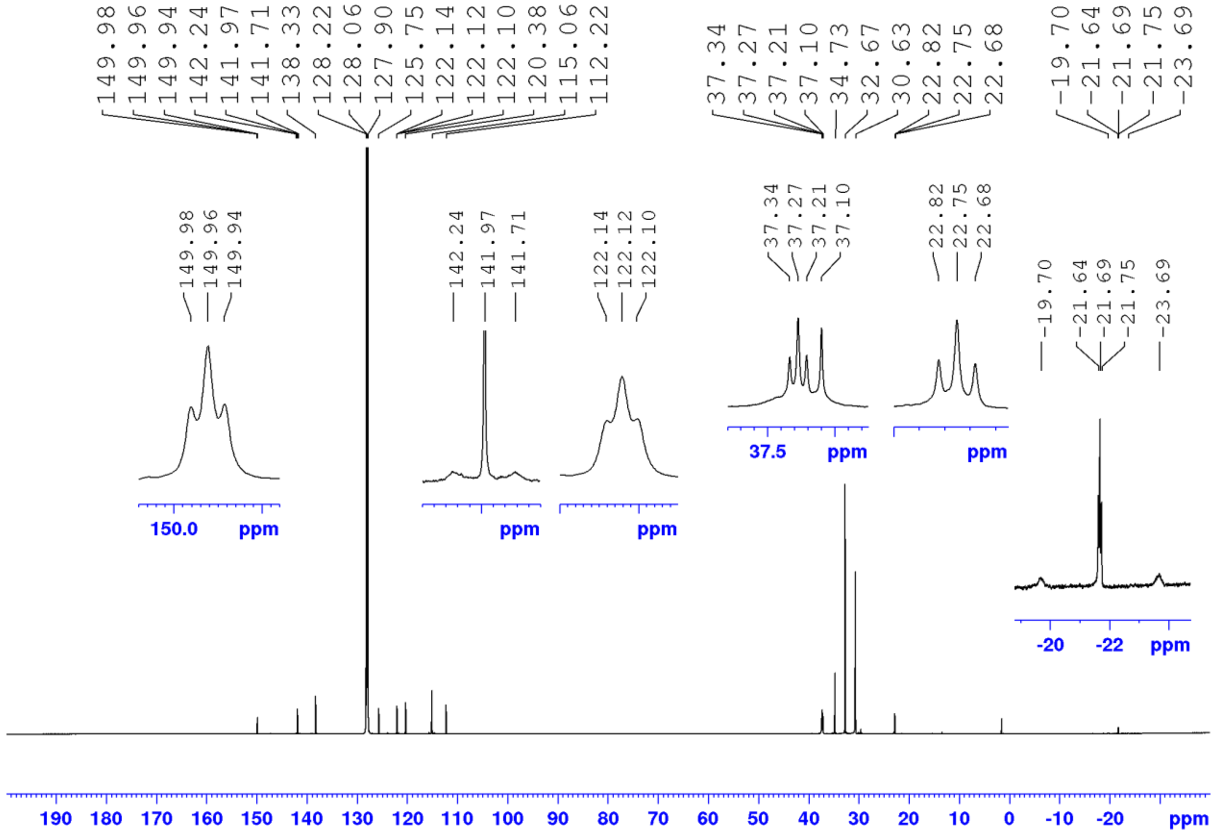

Figure 27: $^{13}C\{^1H\}$ NMR (C_6D_6, 151 MHz, 295K) of $^{Cbz}[^{tBu}PNP]Pt^{II}CH_2CH_2CH{=}CH_2$ (4a).

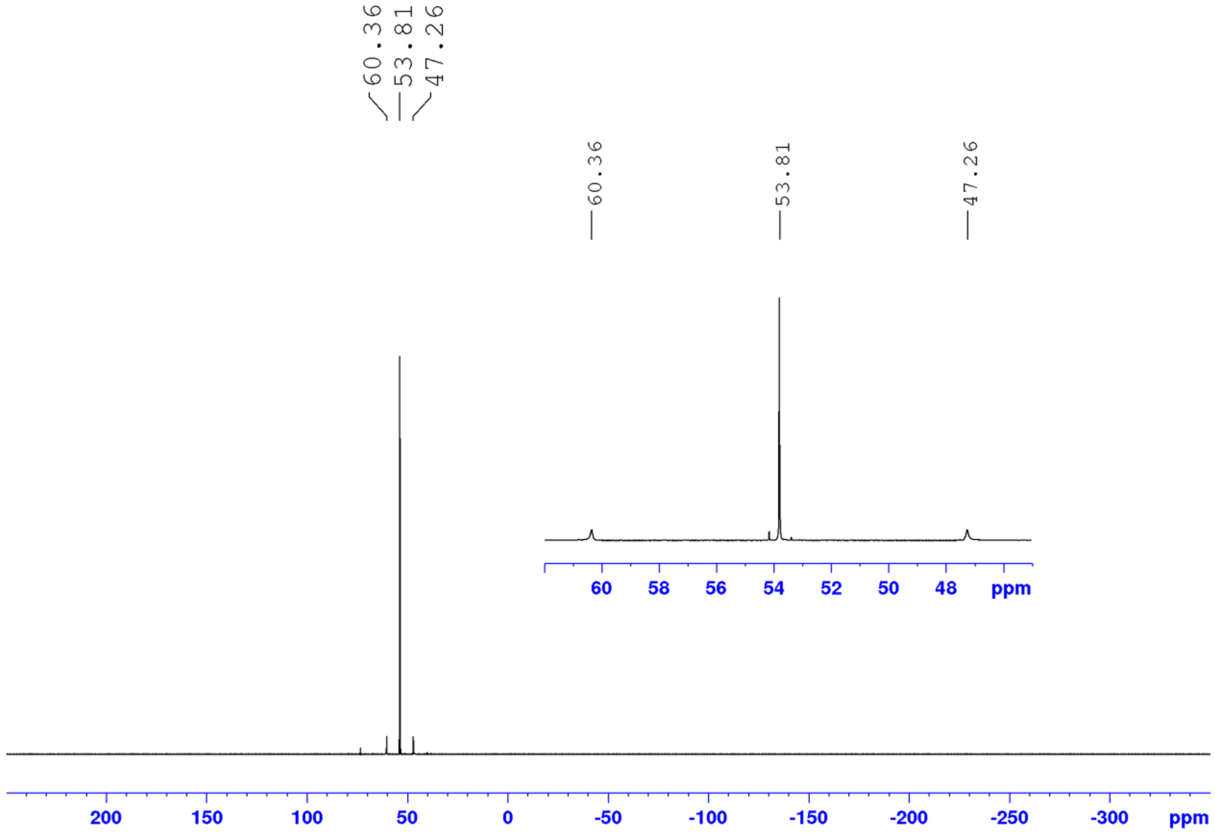

Figure 28: $^{31}P\{^1H\}$ NMR (C_6D_6, 243 MHz, 295K) of $^{Cbz}[^{tBu}PNP]Pt^{II}CH_2CH_2CH{=}CH_2$ (4a).

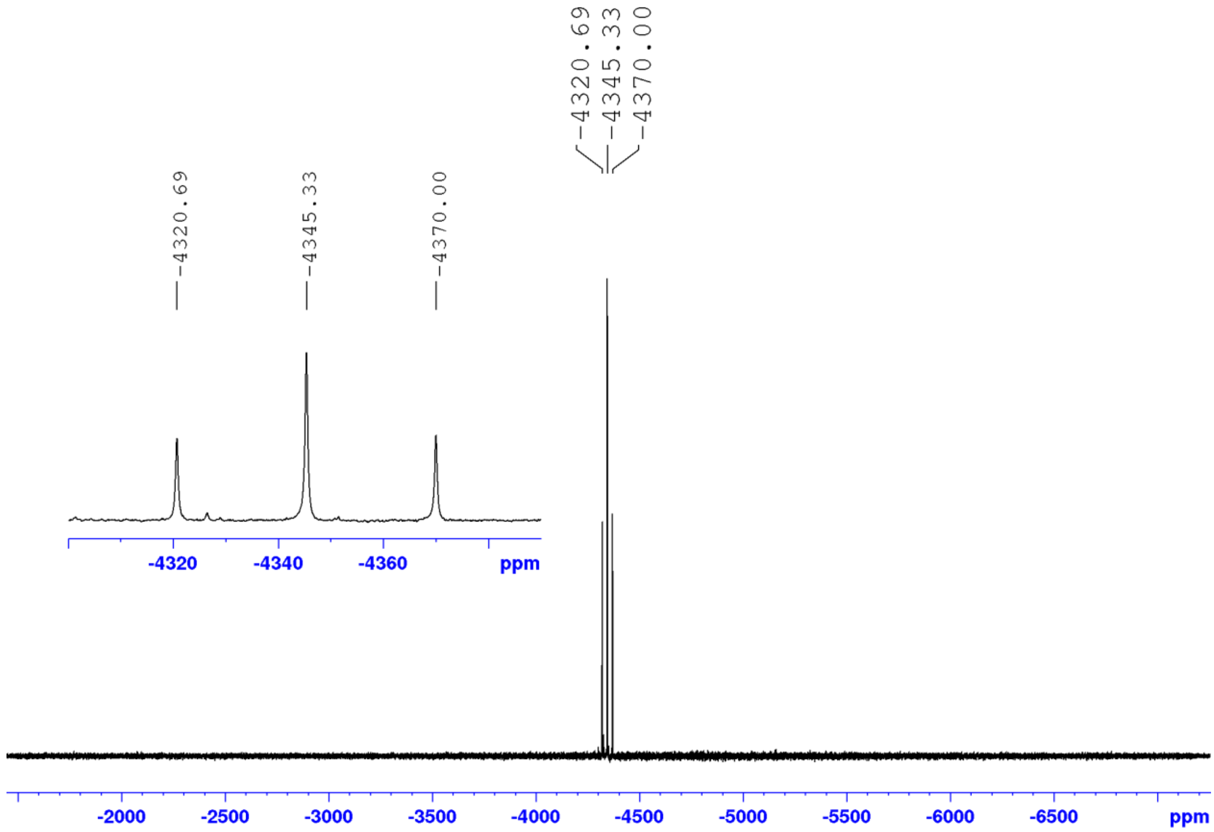

Figure 29: ^{195}Pt{^{1}H} NMR (C_6D_6, 129 MHz, 295K) of ^{Cbz}[^{tBu}PNP]Pt^{II}$CH_2CH_2CH{=}CH_2$ (4a).

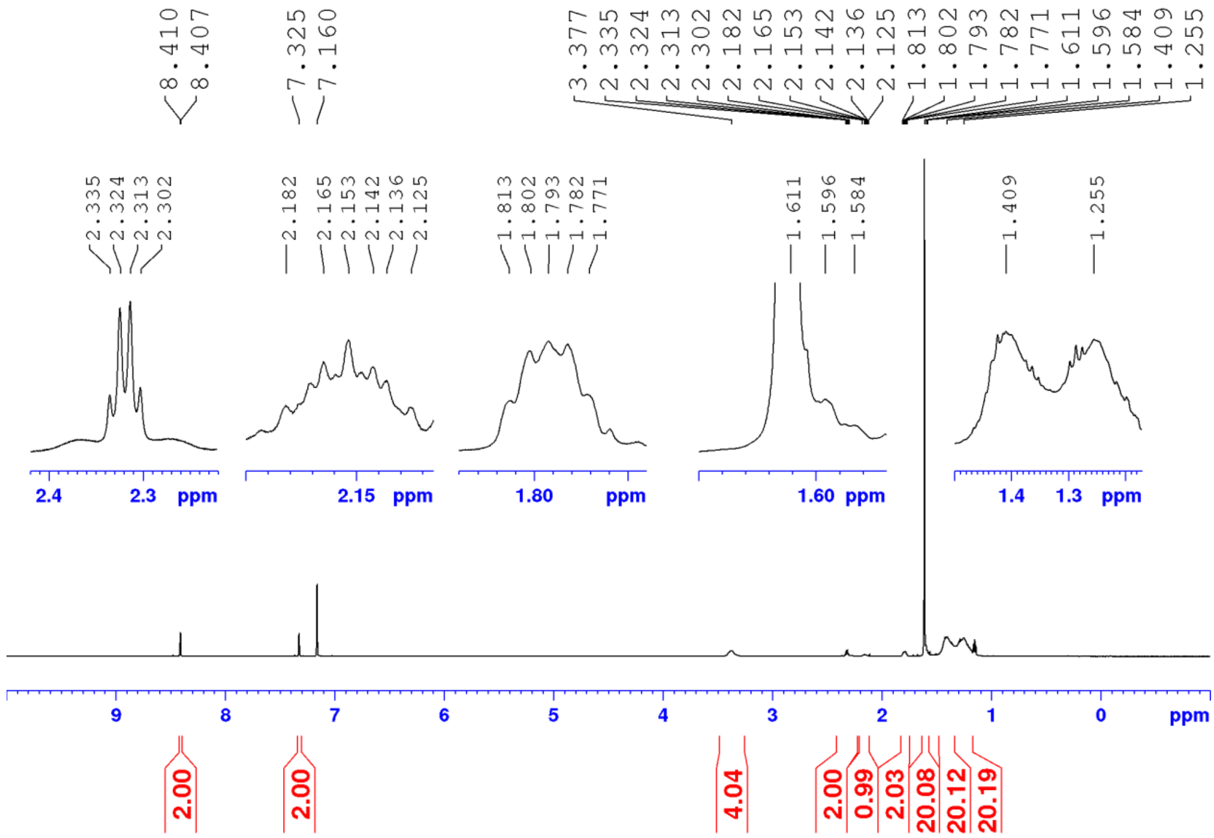

Figure 30: ^{1}H NMR (C_6D_6, 600 MHz, 295K) of ^{Cbz}[^{tBu}PNP]Pt^{II}$CH_2CH(CH_2)_4$ (4b).

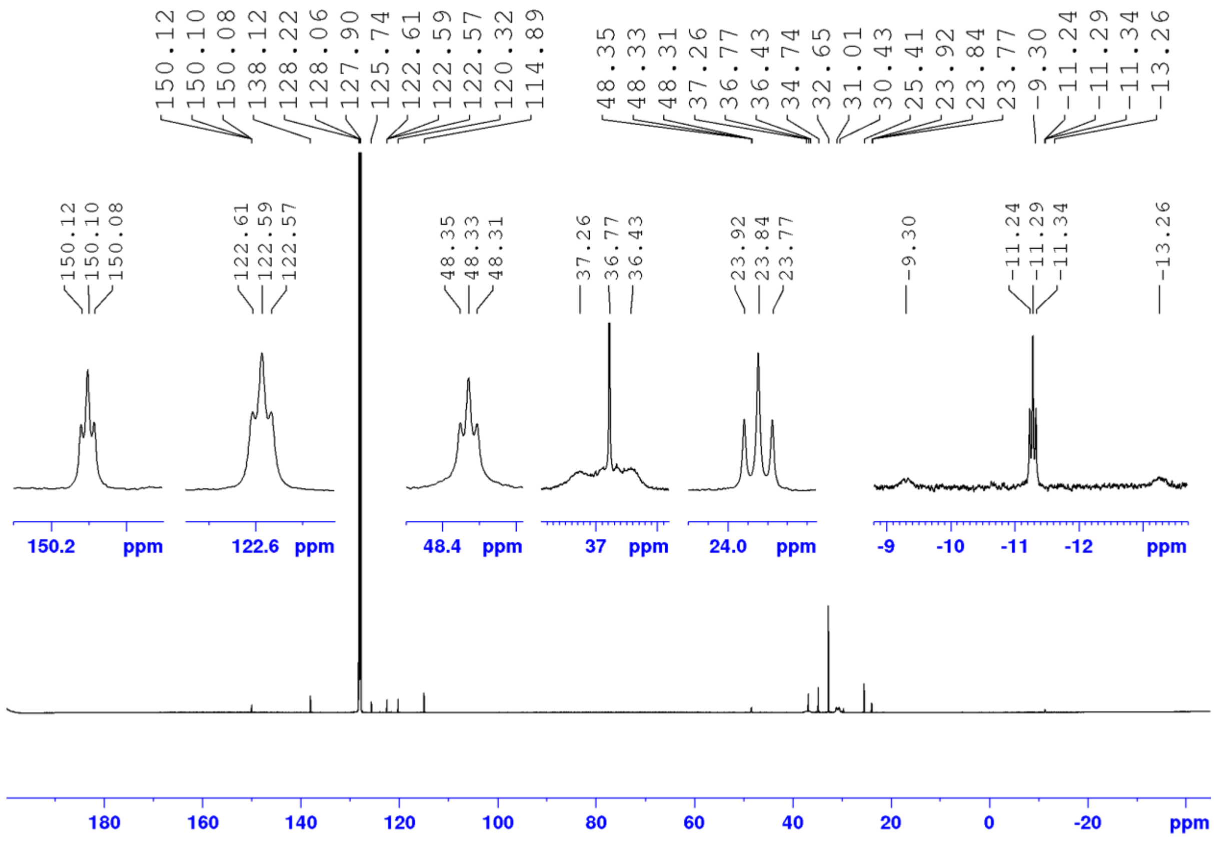

Figure 31: $^{13}C\{^1H\}$ NMR (C_6D_6, 151 MHz, 295K) of $^{Cbz}[^{tBu}PNP]Pt^{II}CH_2CH(CH_2)_4$ (4b).

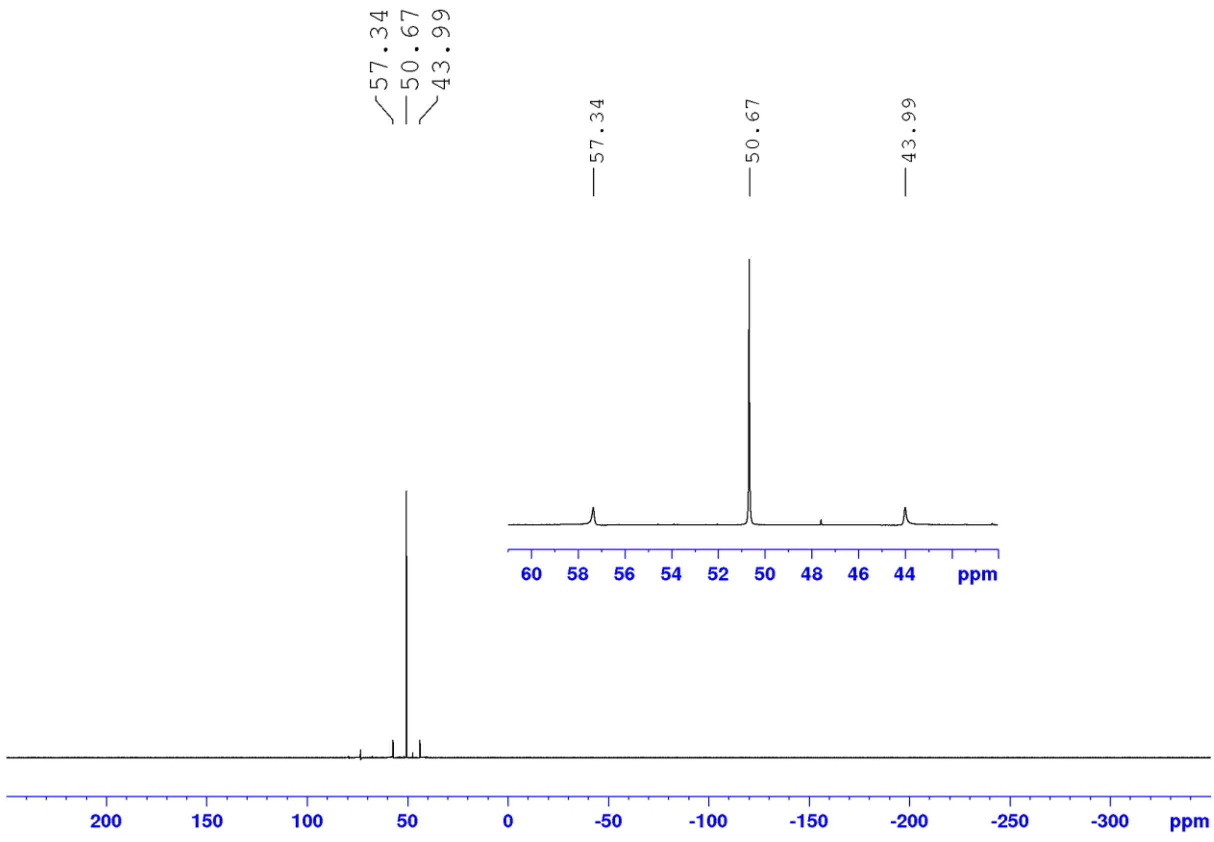

Figure 32: $^{31}P\{^1H\}$ NMR (C_6D_6, 243 MHz, 295K) of $^{Cbz}[^{tBu}PNP]Pt^{II}CH_2CH(CH_2)_4$ (4b).

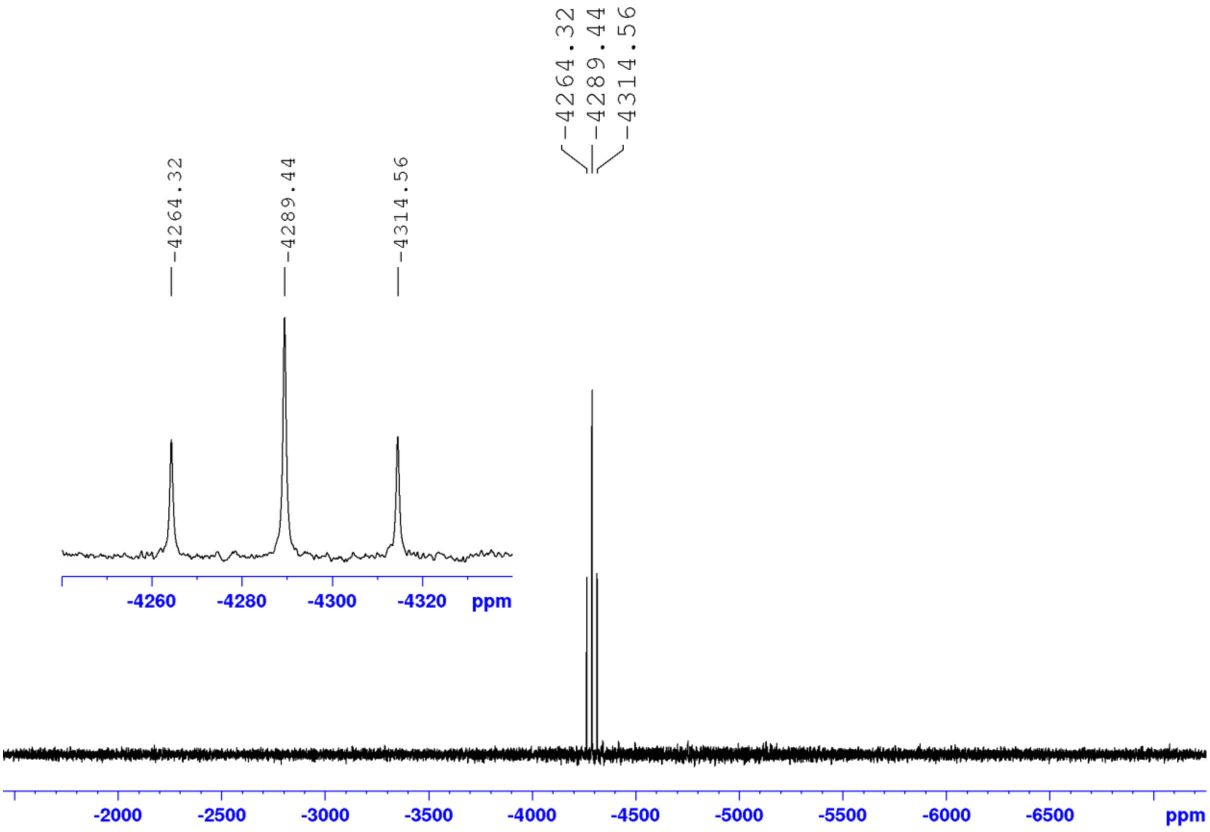

Figure 33: ^{195}Pt{^{1}H} NMR (C_6D_6, 129 MHz, 295K) of ^{Cbz}[^{tBu}PNP]$Pt^{II}CH_2CH(CH_2)_4$ (4b).

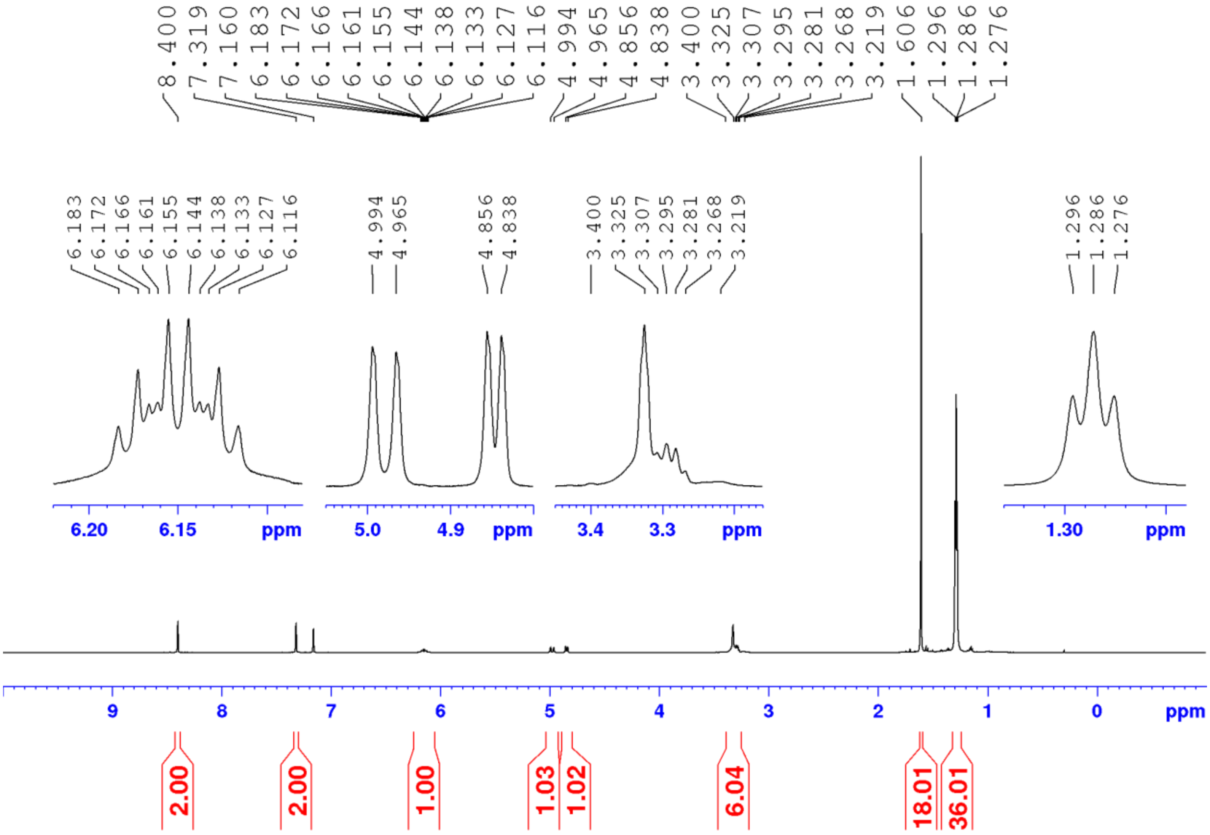

Figure 34: ^{1}H NMR (C_6D_6, 600 MHz, 295K) of ^{Cbz}[^{tBu}PNP]$Pt^{II}CH_2CH{=}CH_2$ (4c).

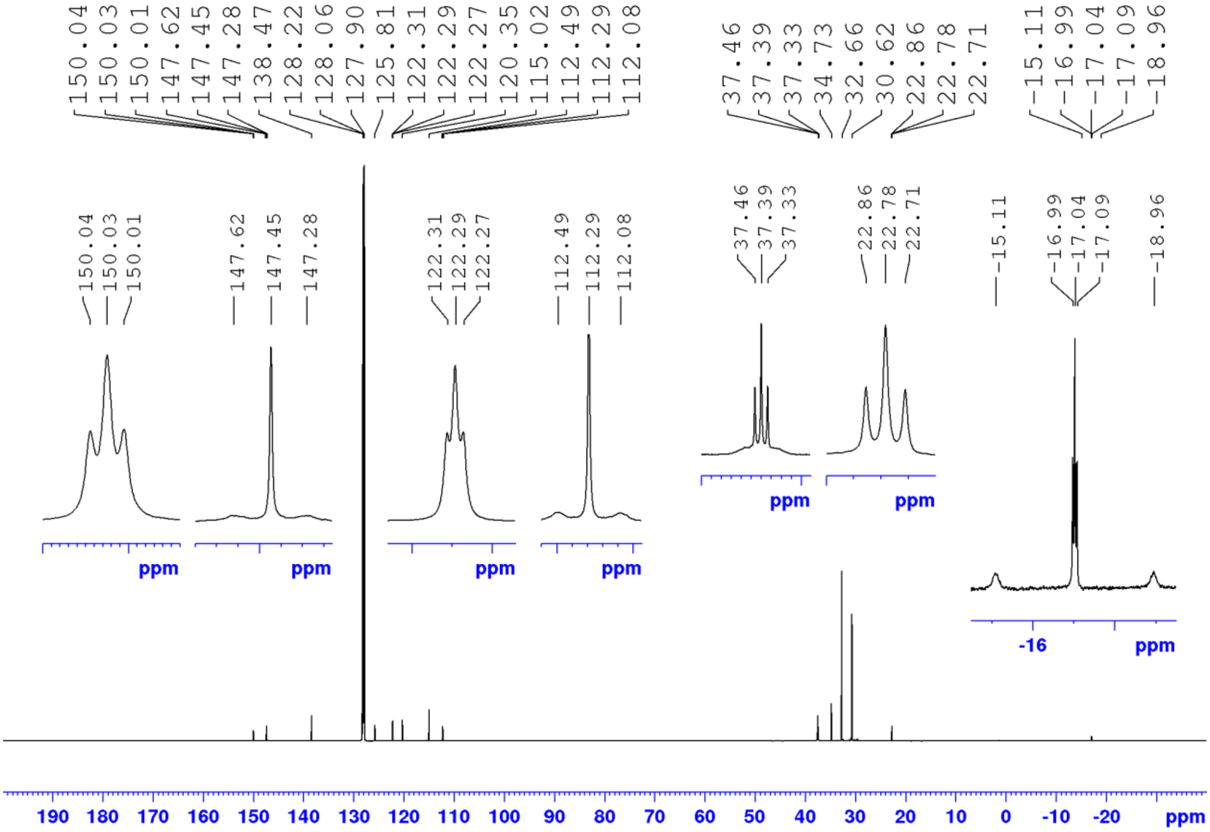

Figure 35: $^{13}C\{^1H\}$ NMR (C_6D_6, 151 MHz, 295K) of $^{Cbz}[^{tBu}PNP]Pt^{II}CH_2CH{=}CH_2$ (4c).

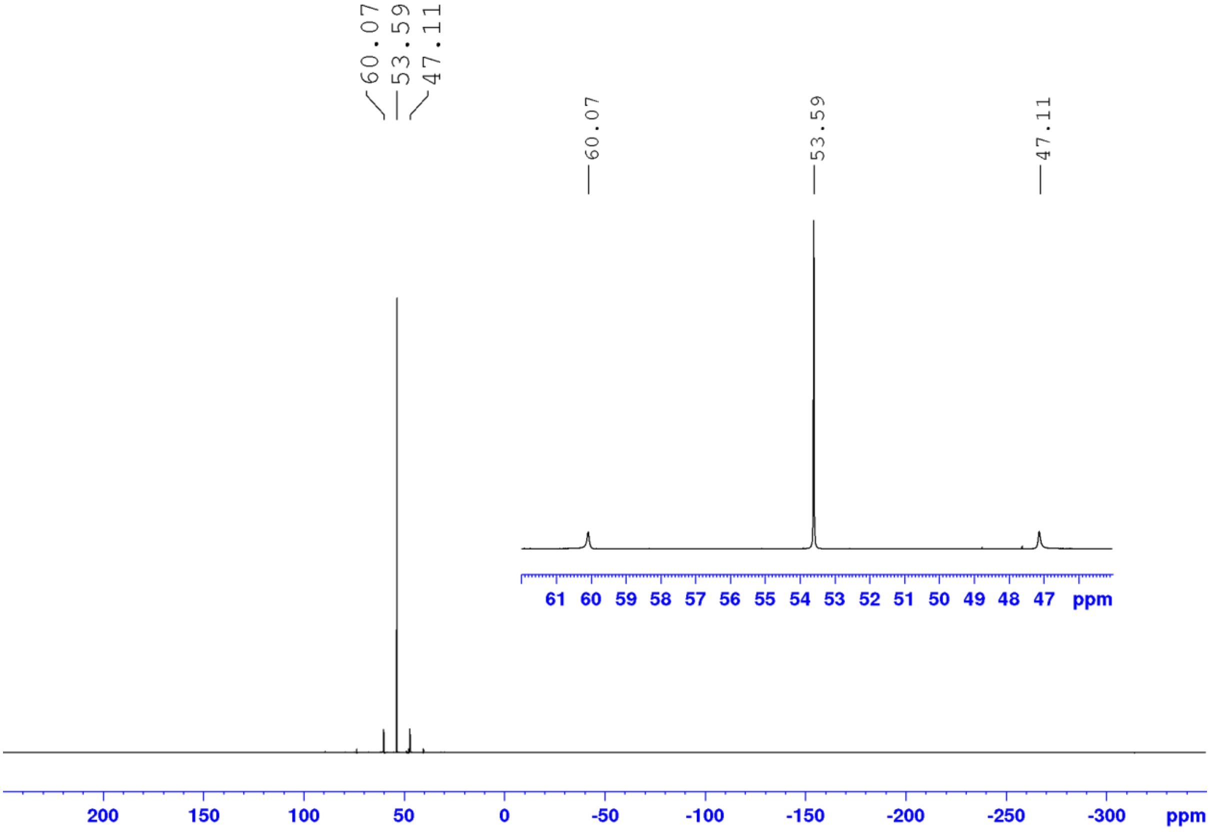

Figure 36: $^{31}P\{^1H\}$ NMR (C_6D_6, 243 MHz, 295K) of $^{Cbz}[^{tBu}PNP]Pt^{II}CH_2CH{=}CH_2$ (4c).

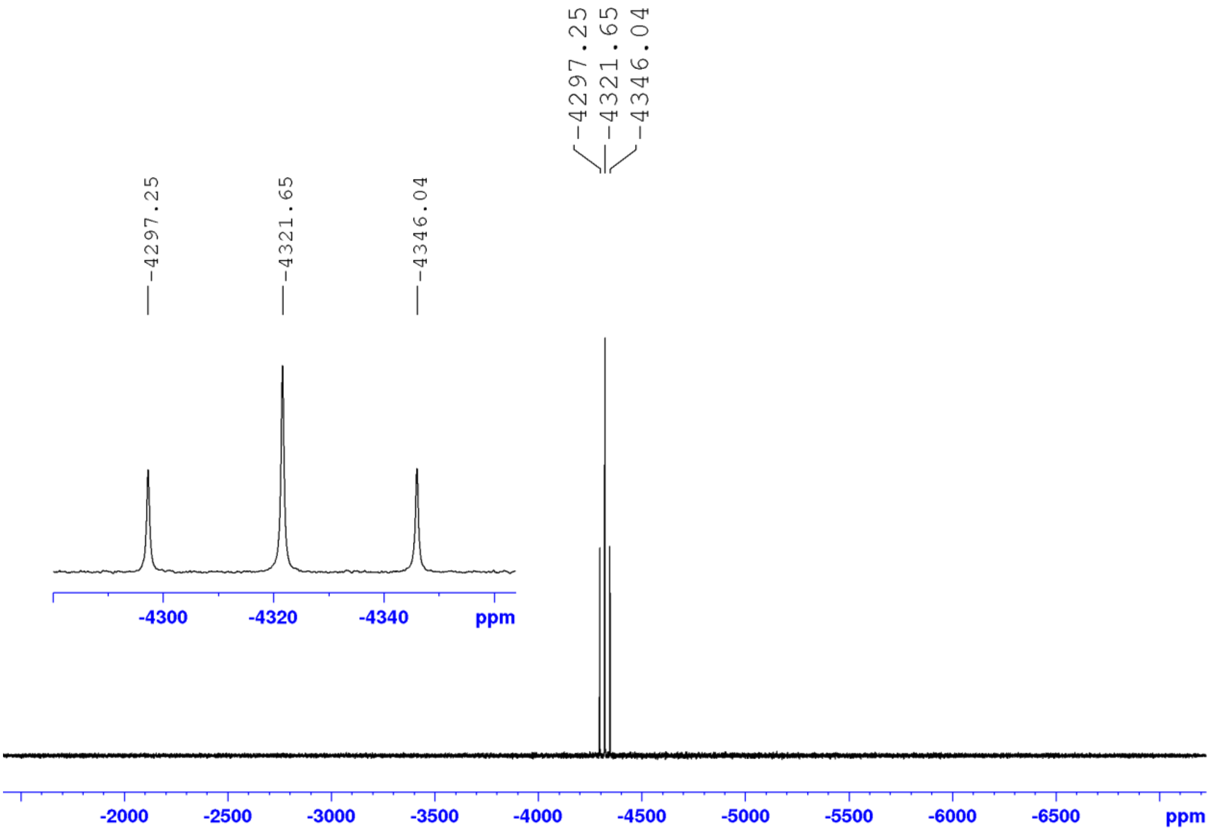

Figure 37: $^{195}Pt\{^1H\}$ NMR (C_6D_6, 129 MHz, 295K) of $^{Cbz}[^{tBu}PNP]Pt^{II}CH_2CH{=}CH_2$ (4c).

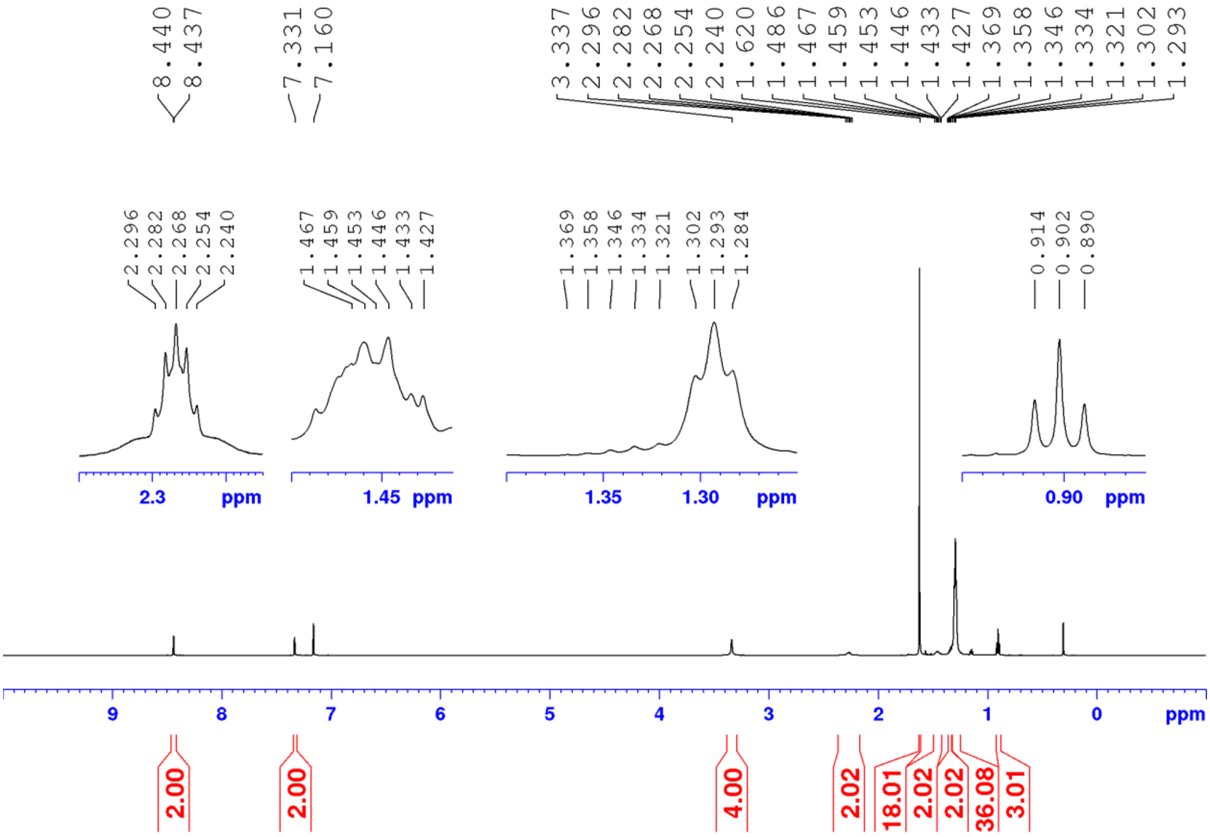

Figure 38: ^1H NMR (C_6D_6, 600 MHz, 295K) of $^{Cbz}[^{tBu}PNP]Pt^{II}CH_2CH_2CH_2CH_3$ (4d).

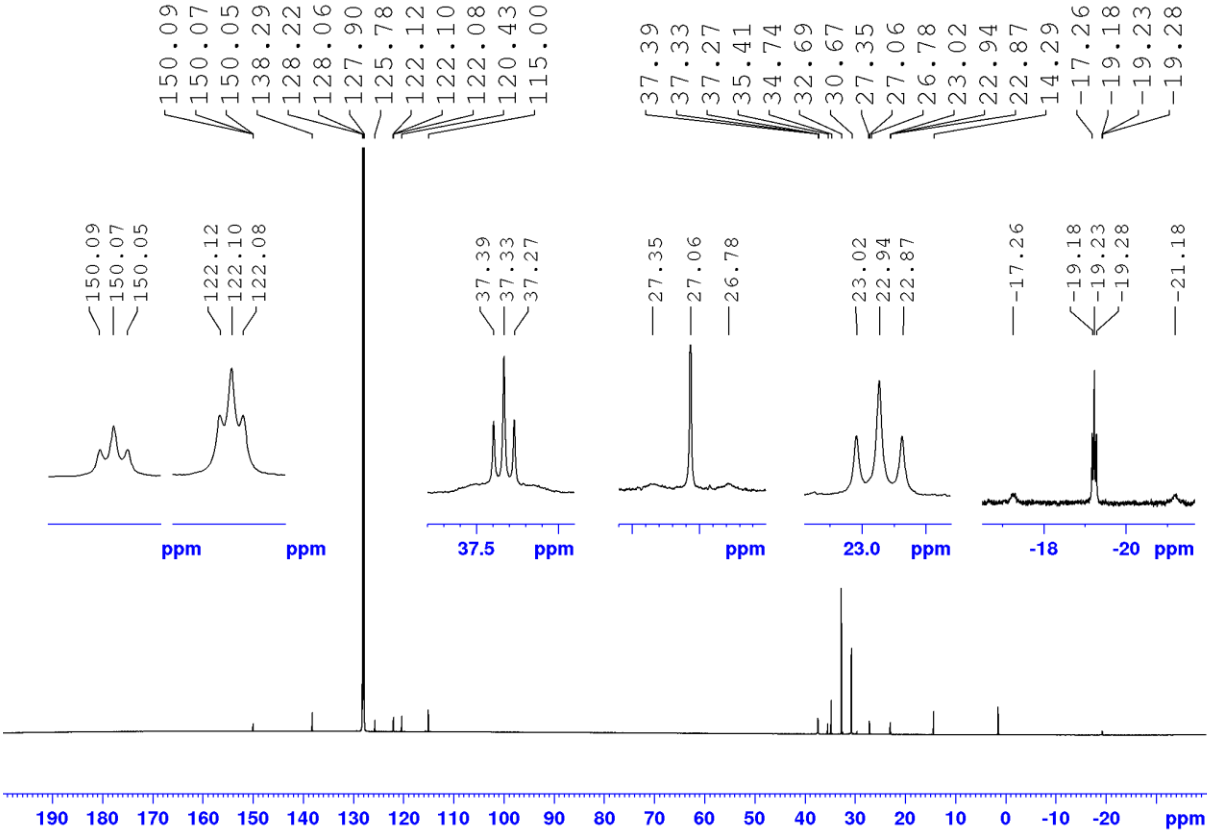

Figure 39: $^{13}C\{^1H\}$ NMR (C_6D_6, 151 MHz, 295K) of $^{Cbz}[^{tBu}PNP]Pt^{II}CH_2CH_2CH_2CH_3$ (4d).

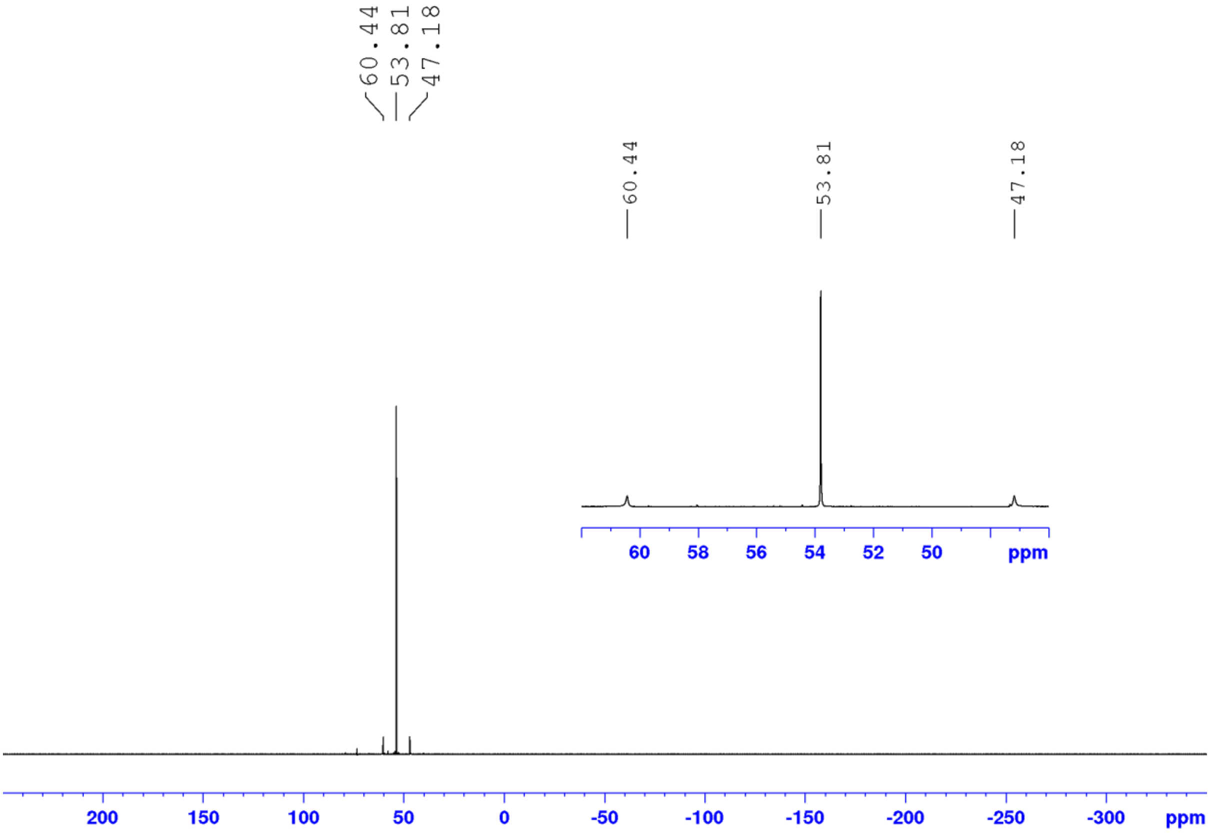

Figure 40: $^{31}P\{^1H\}$ NMR (C_6D_6, 243 MHz, 295K) of $^{Cbz}[^{tBu}PNP]Pt^{II}CH_2CH_2CH_2CH_3$ (4d).

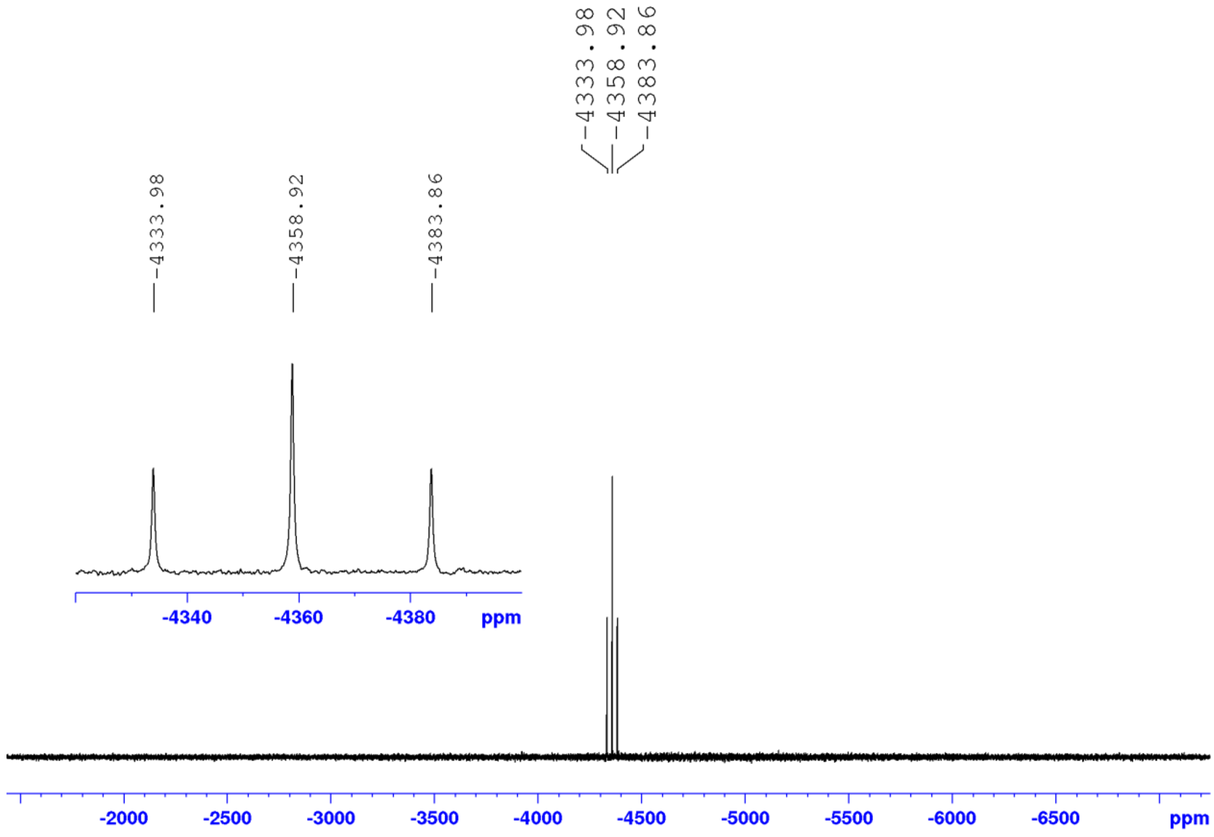

Figure 41: $^{195}Pt\{^1H\}$ NMR (C_6D_6, 129 MHz, 295K) of $^{Cbz}[^{tBu}PNP]Pt^{II}CH_2CH_2CH_2CH_3$ (4d).

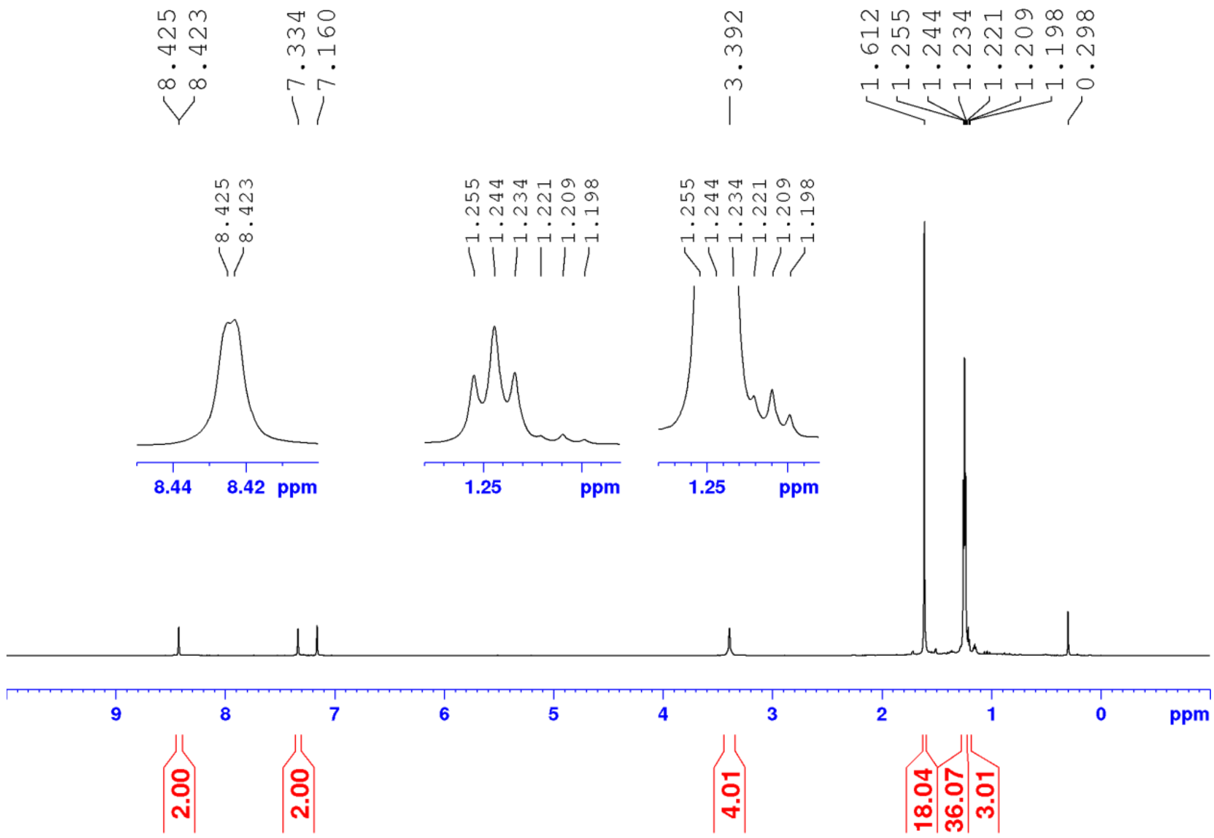

Figure 42: ^1H NMR (C_6D_6, 600 MHz, 295K) of $^{Cbz}[^{tBu}PNP]Pt^{II}CH_3$ (4e).

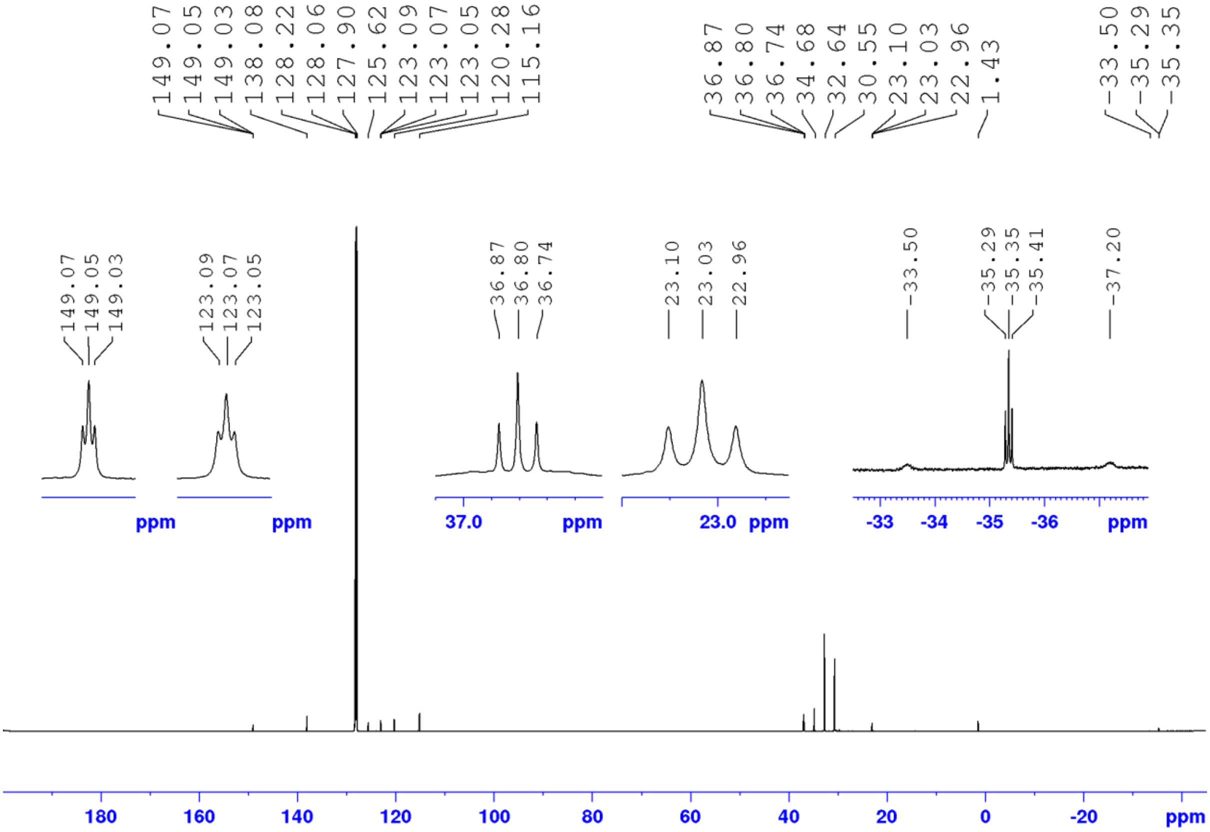

Figure 43: $^{13}C\{^{1}H\}$ NMR (C_6D_6, 151 MHz, 295K) of $^{Cbz}[^{tBu}PNP]Pt^{II}CH_3$ (4e).

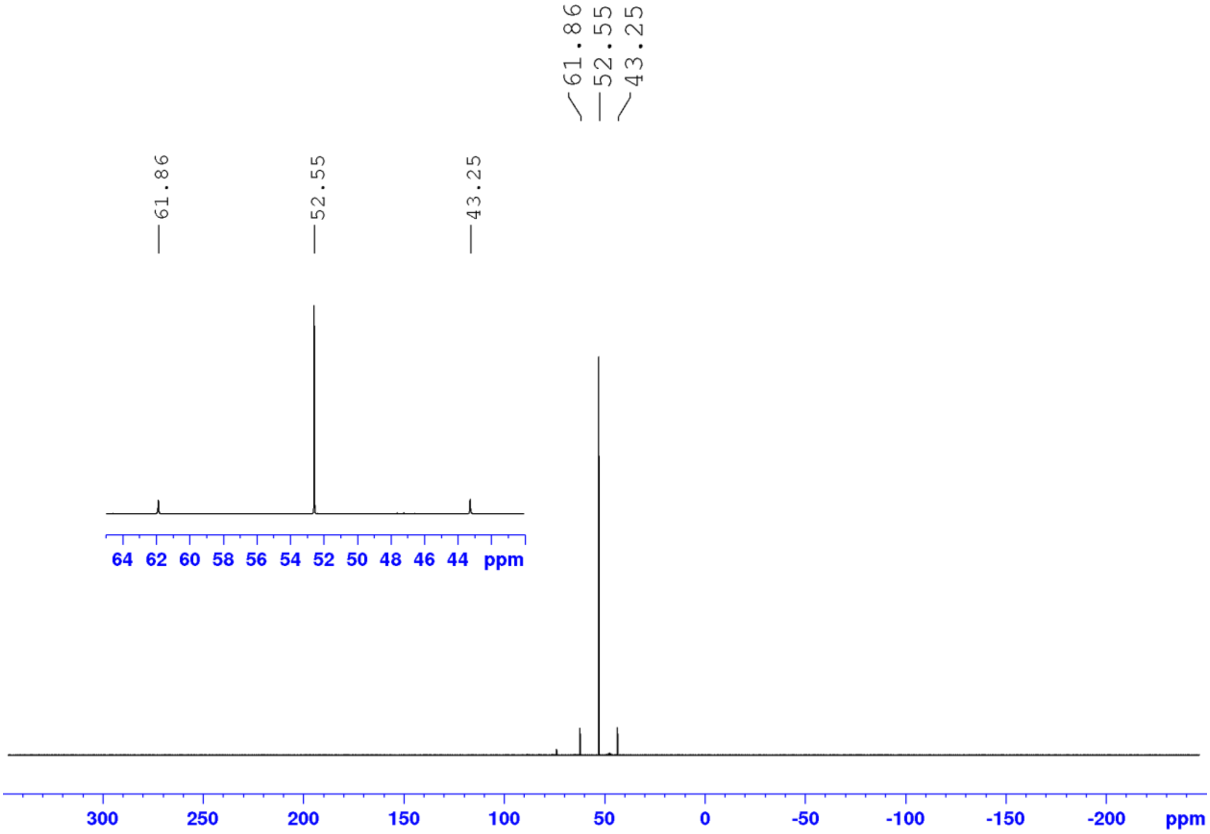

Figure 44: $^{31}P\{^{1}H\}$ NMR (C_6D_6, 162 MHz, 295K) of $^{Cbz}[^{tBu}PNP]Pt^{II}CH_3$ (4e).

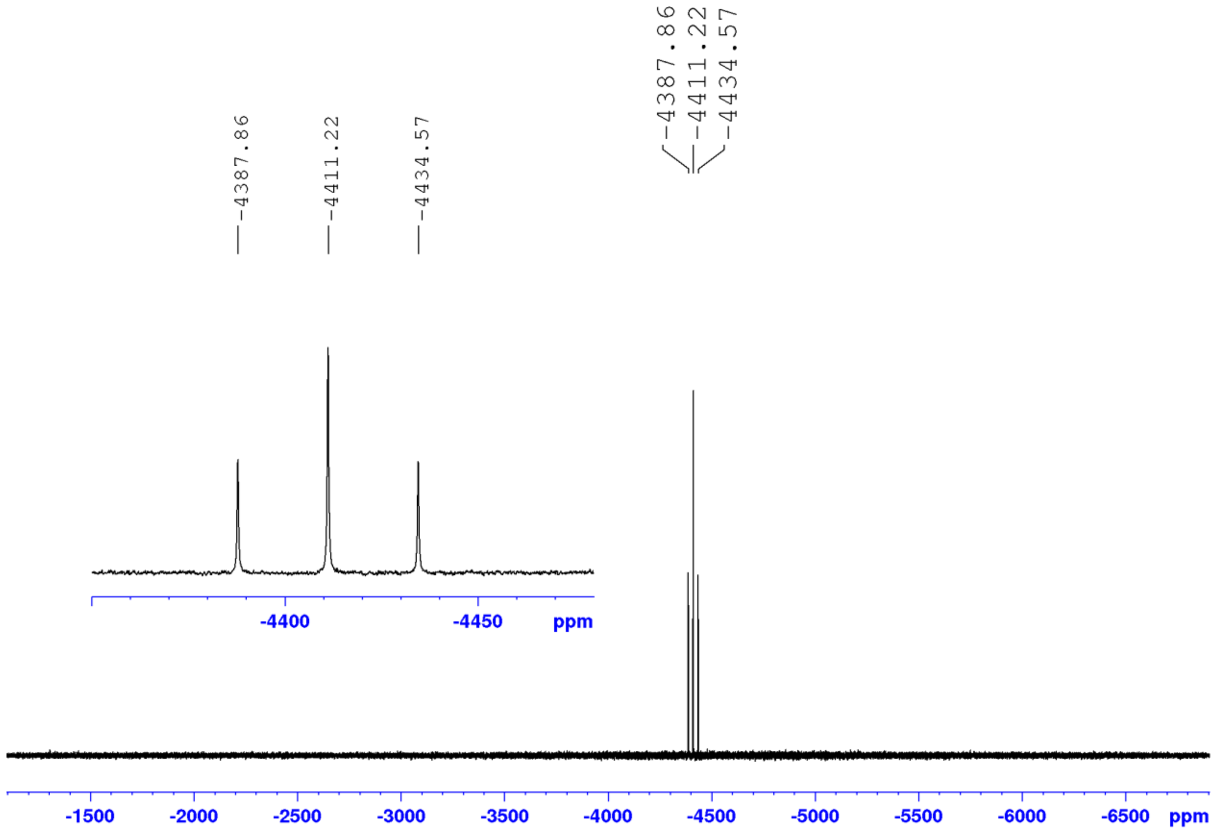

Figure 45: $^{195}Pt\{^1H\}$ NMR (C_6D_6, 129 MHz, 295K) of $^{Cbz}[^{tBu}PNP]Pt^{II}CH_3$ (4e).

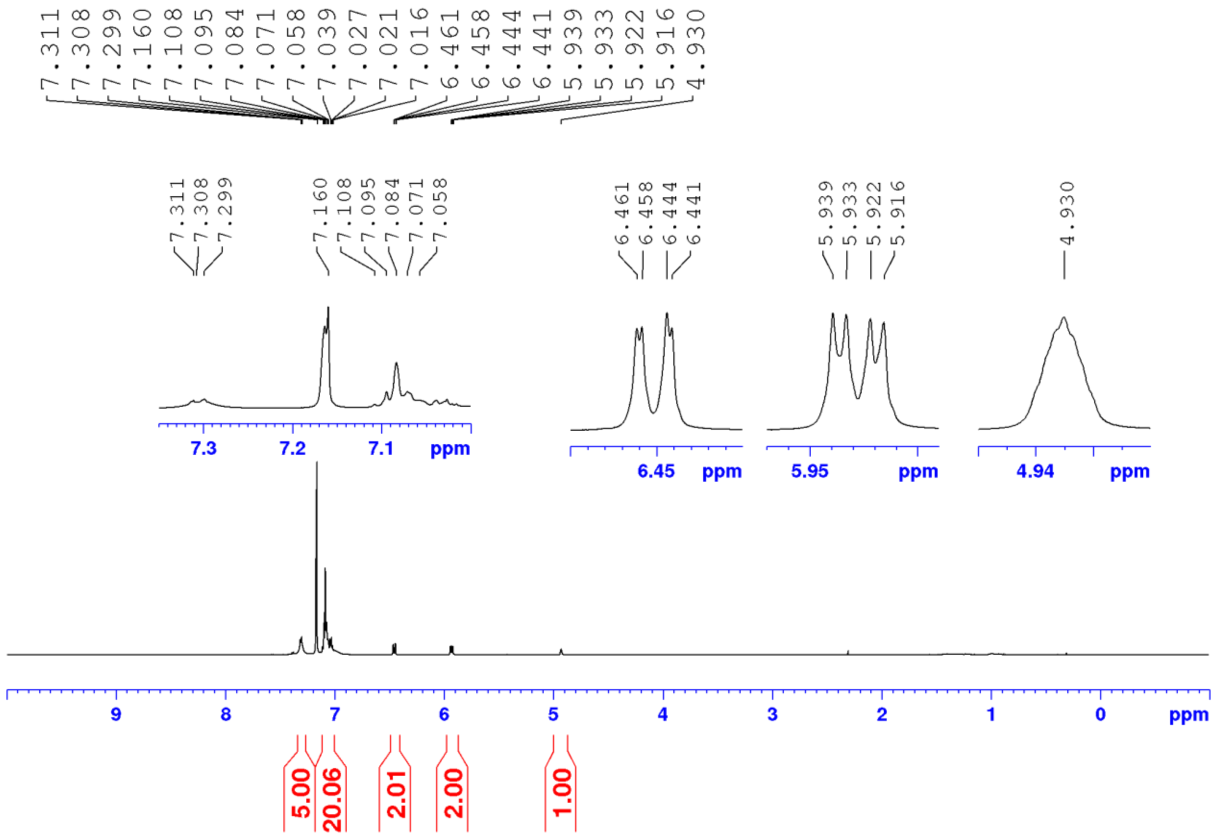

Figure 46: ^1H NMR (C_6D_6, 600 MHz, 295K) of 1-(Diphenylmethylene)-4-trityl-2,5-cyclohexadiene (5-A').

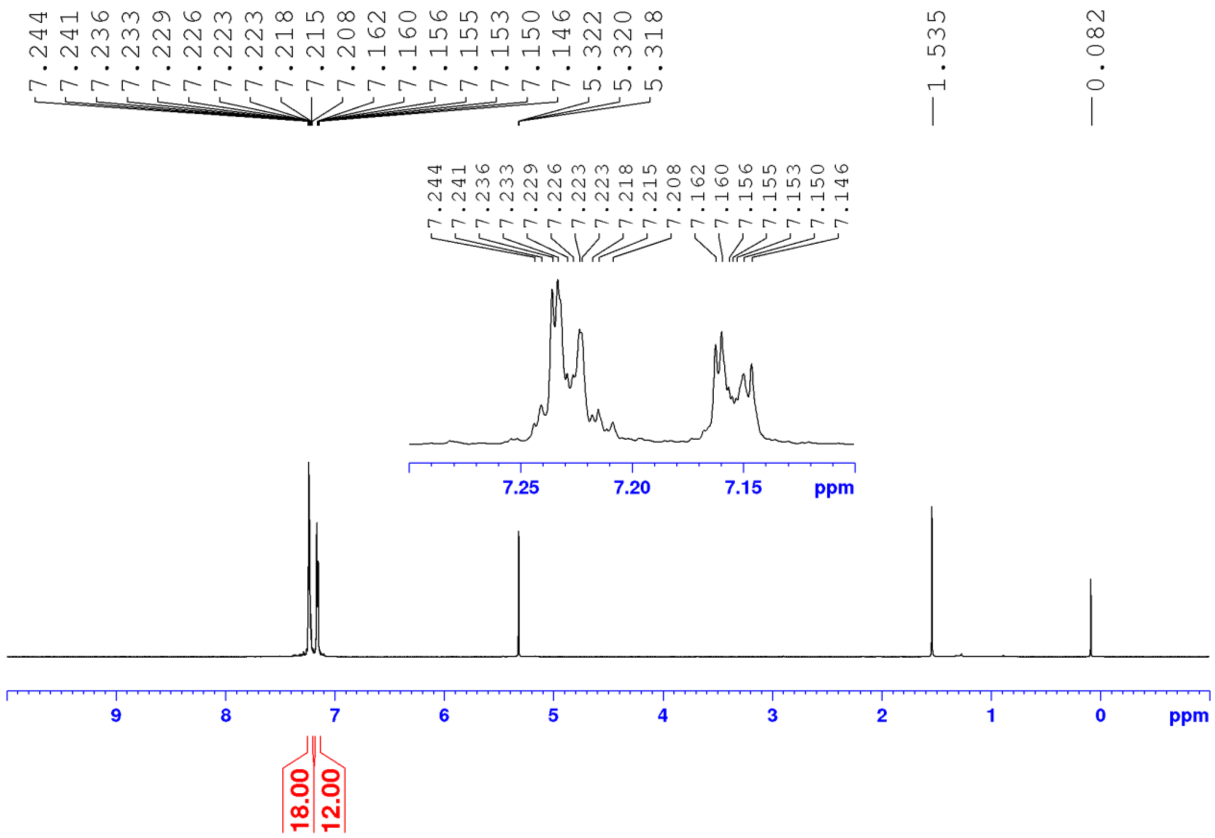

Figure 47: ^{1}H NMR (CD_2Cl_2, 600 MHz, 295K) of Bis(triphenylmethyl) peroxide (5-A).

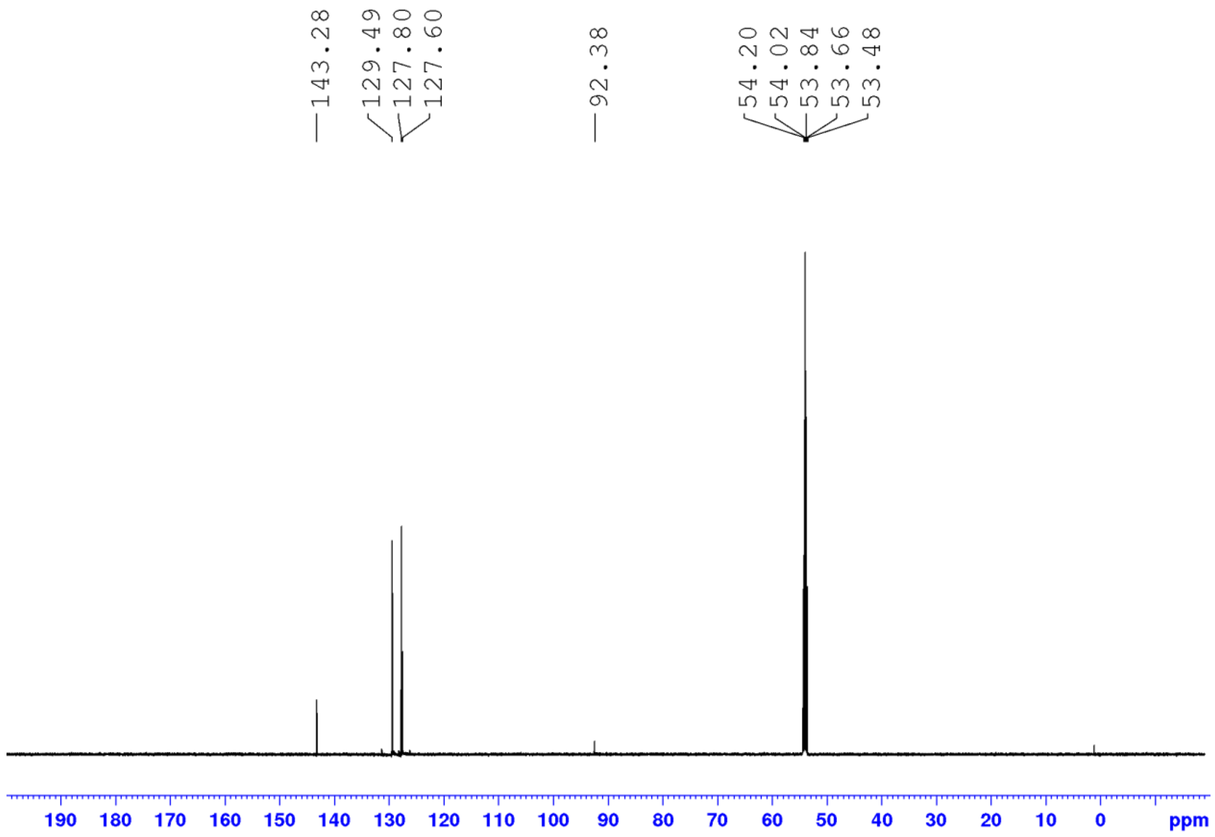

Figure 48: ^{13}C{^{1}H} NMR (CD_2Cl_2, 151 MHz, 295K) of Bis(triphenylmethyl) peroxide (5-A).

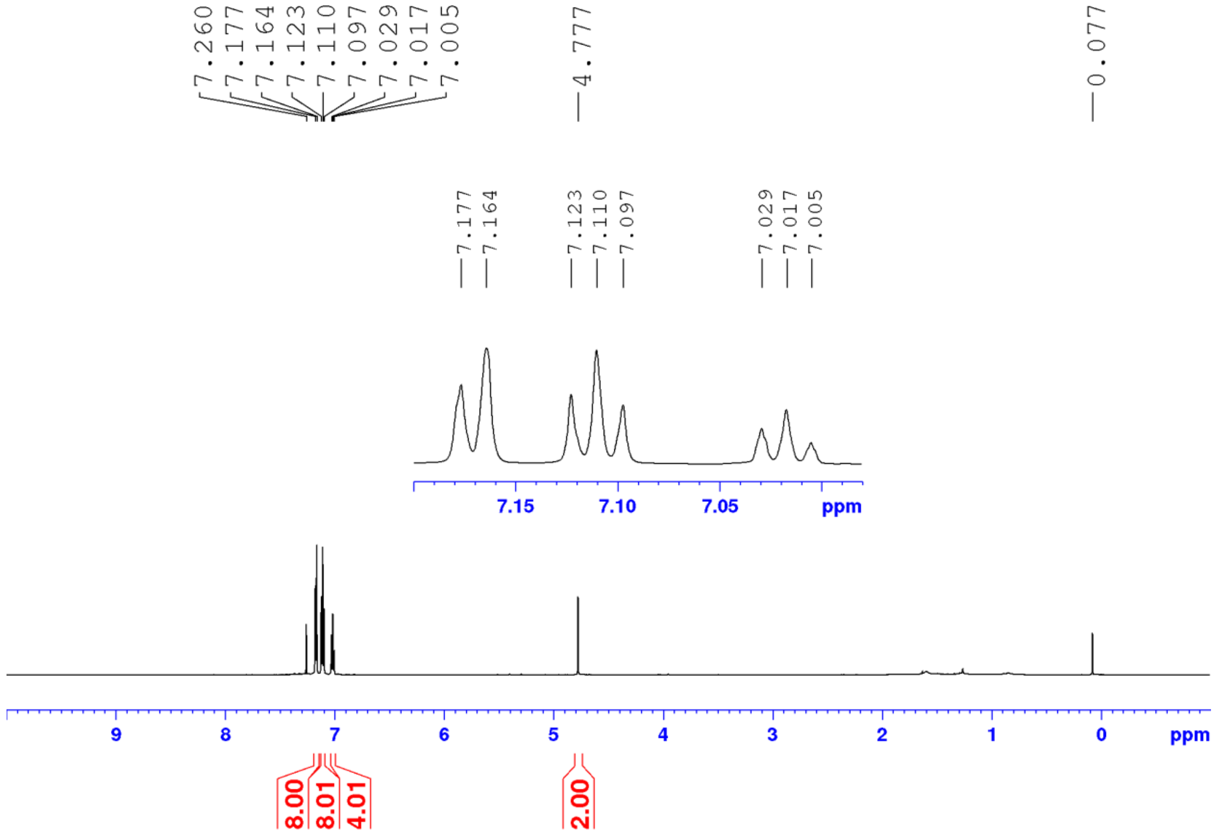

Figure 49: ^1H NMR ($CDCl_3$, 600 MHz, 295K) of 1,1,2,2-Tetraphenylethane (5-B).

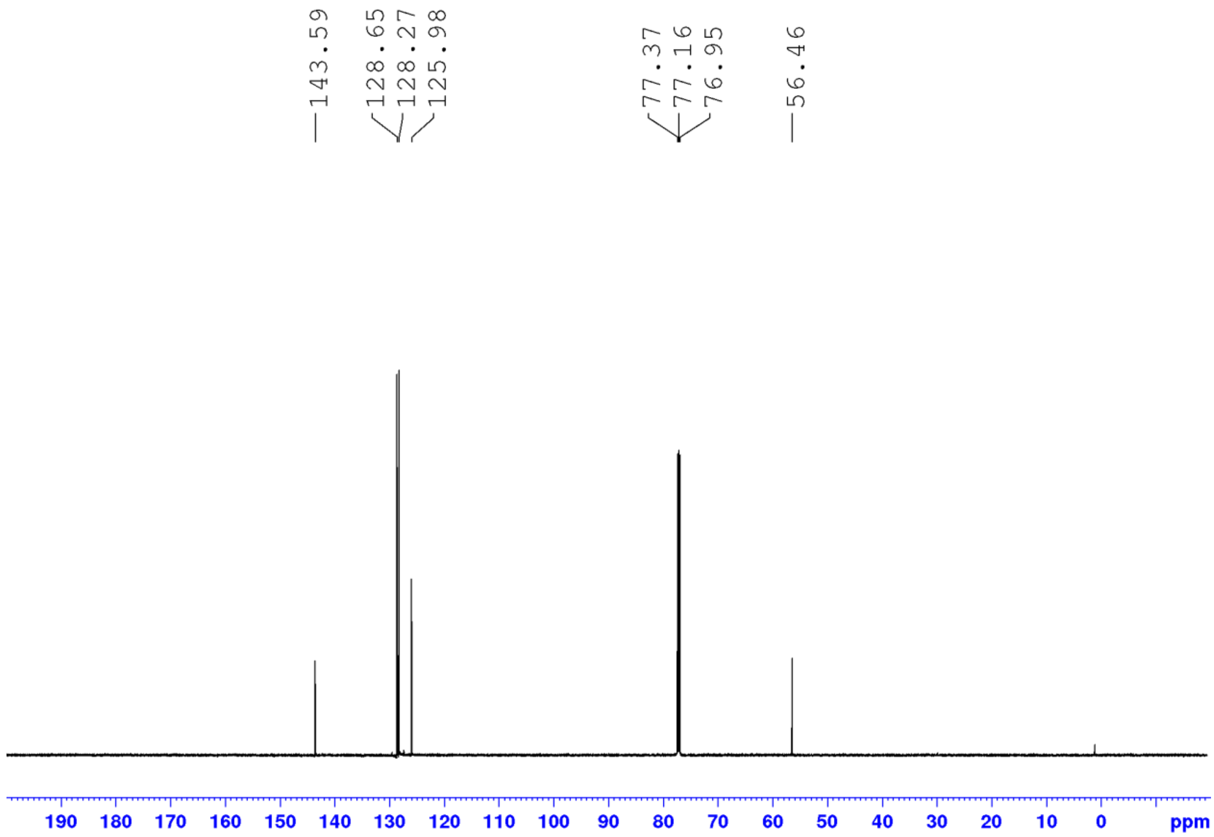

Figure 50: $^{13}C\{^1H\}$ NMR ($CDCl_3$, 151 MHz, 295K) of 1,1,2,2-Tetraphenylethane (5-B).

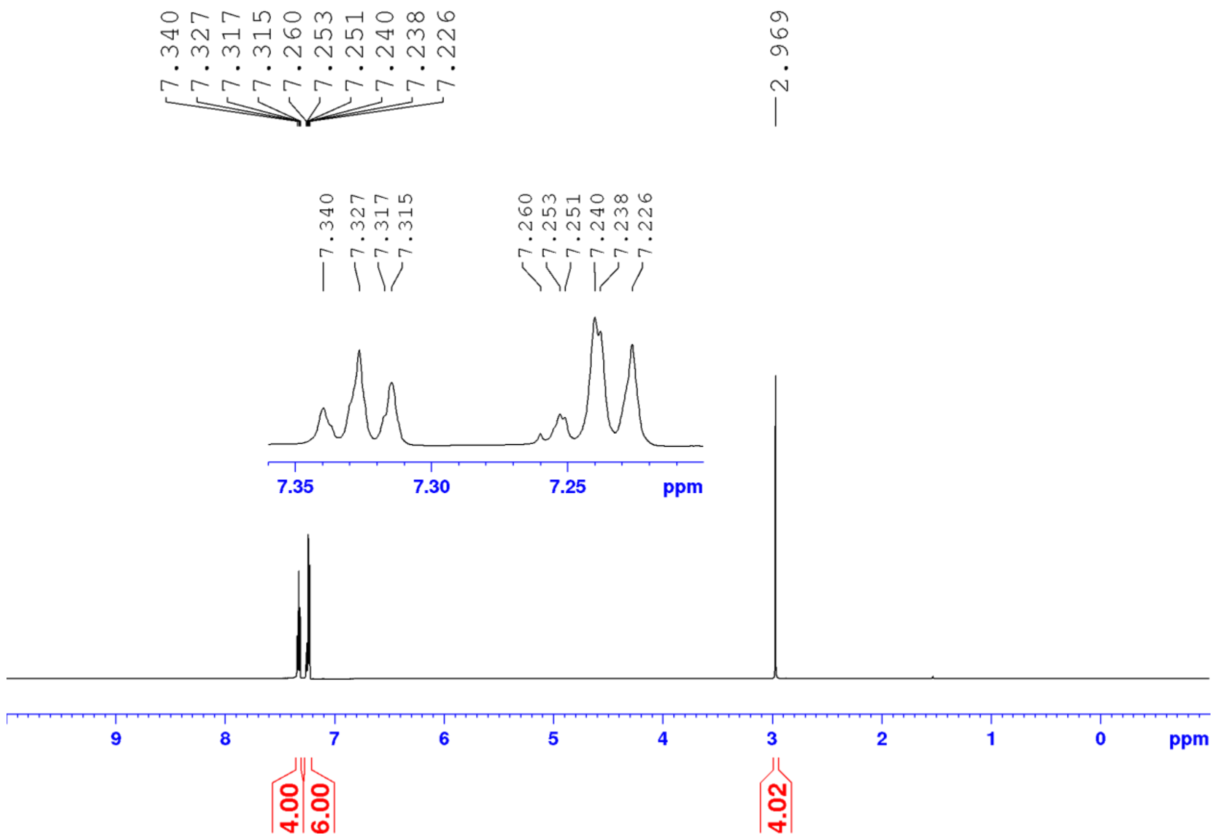

Figure 51: ^{1}H NMR ($CDCl_3$, 600 MHz, 295K) of 1,2-Diphenylethane (5-C).

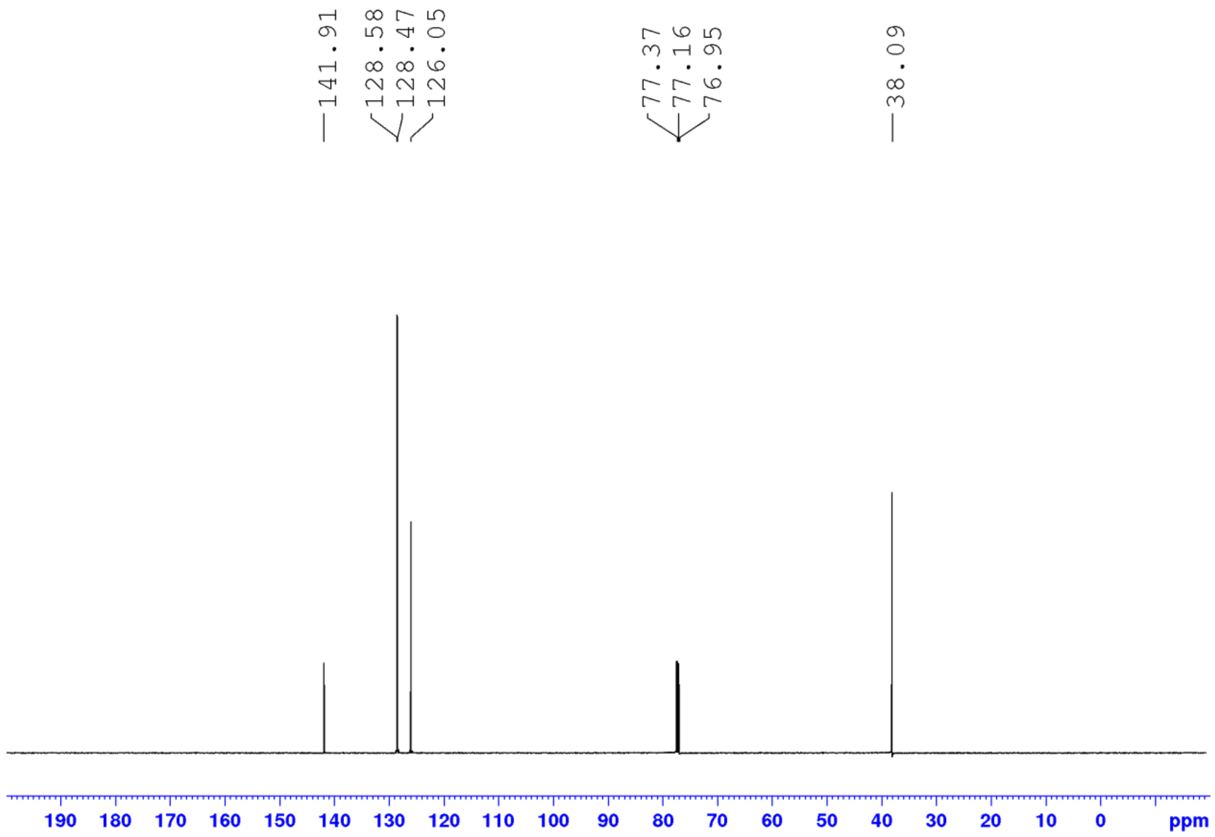

Figure 52: $^{13}C\{^{1}H\}$ NMR ($CDCl_3$, 151 MHz, 295K) of 1,2-Diphenylethane (5-C).

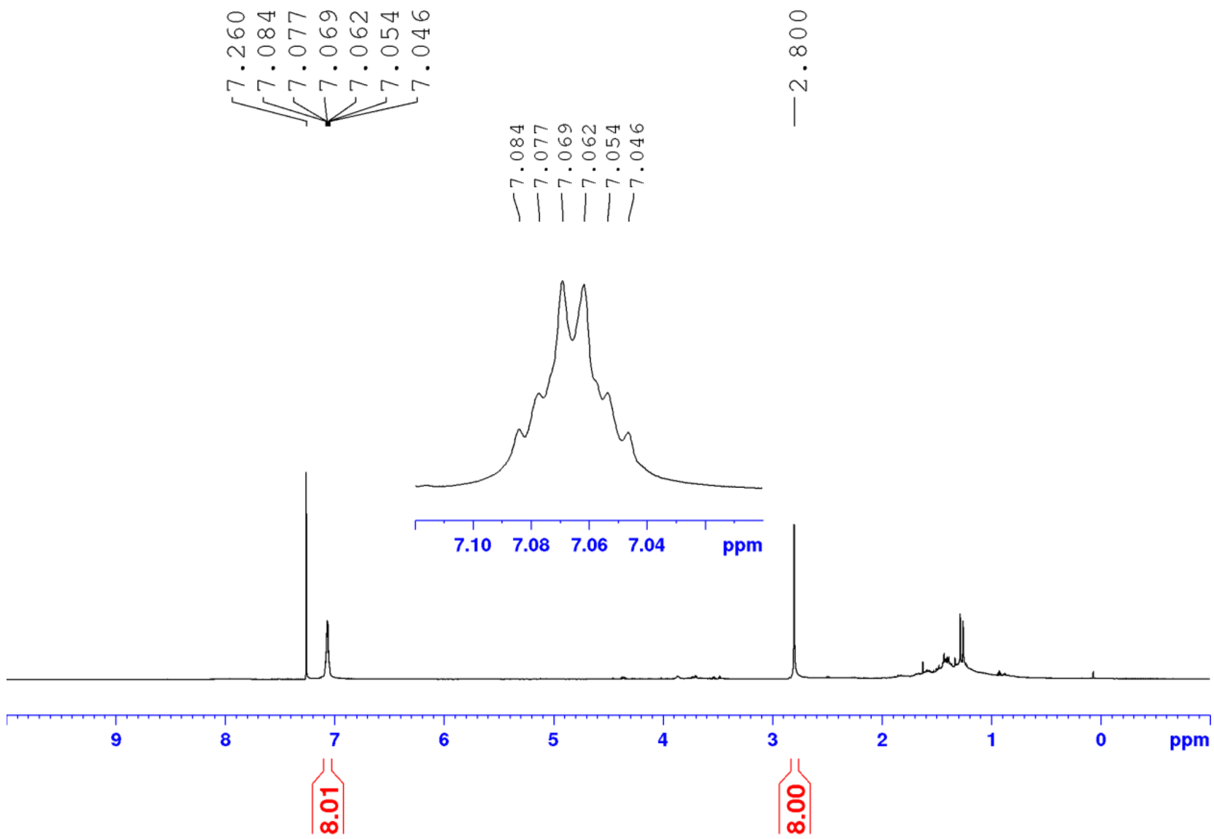

Figure 53: ^{1}H NMR ($CDCl_3$, 600 MHz, 295K) of 5,6,11,12-Tetrahydrodibenzo[*a*,*e*][8]annulene (5-D).

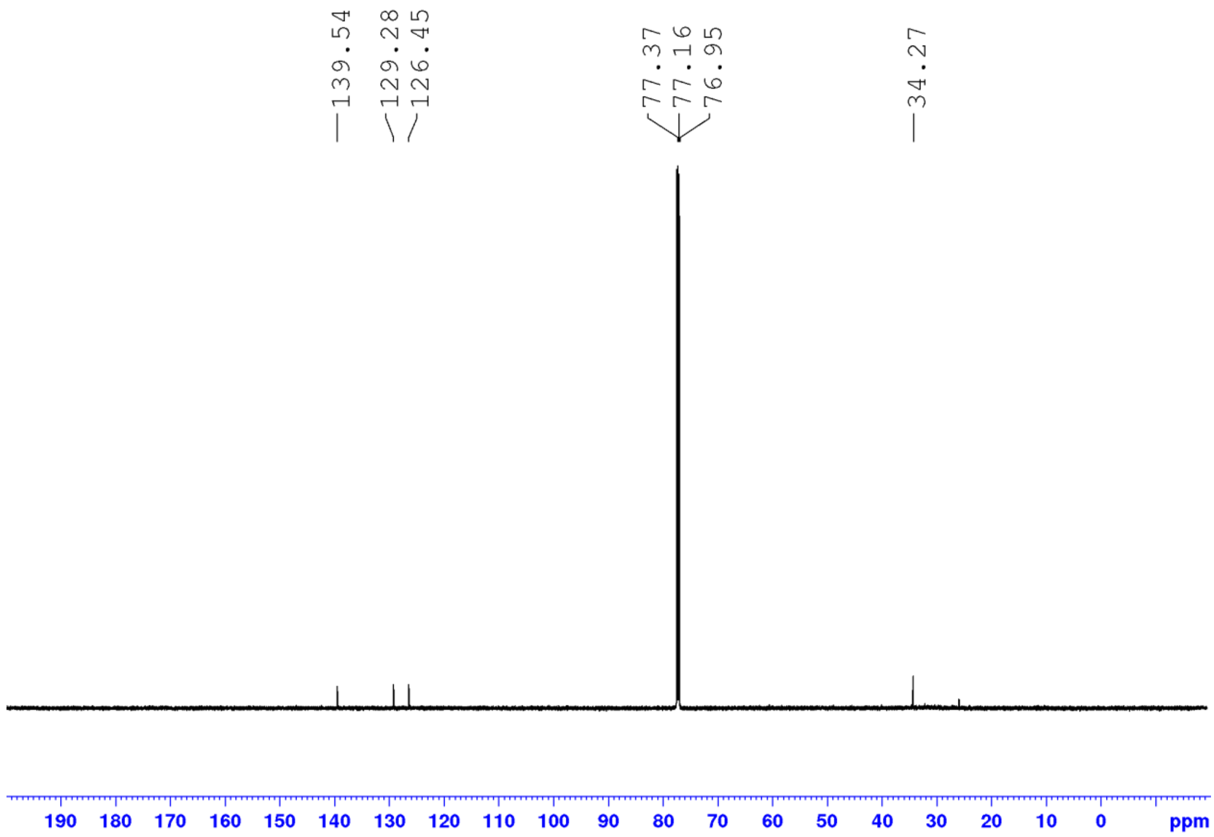

Figure 54: $^{13}C\{^{1}H\}$ NMR ($CDCl_3$, 151 MHz, 295K) of 5,6,11,12-Tetrahydrodibenzo[*a*,*e*][8]annulene (5-D).

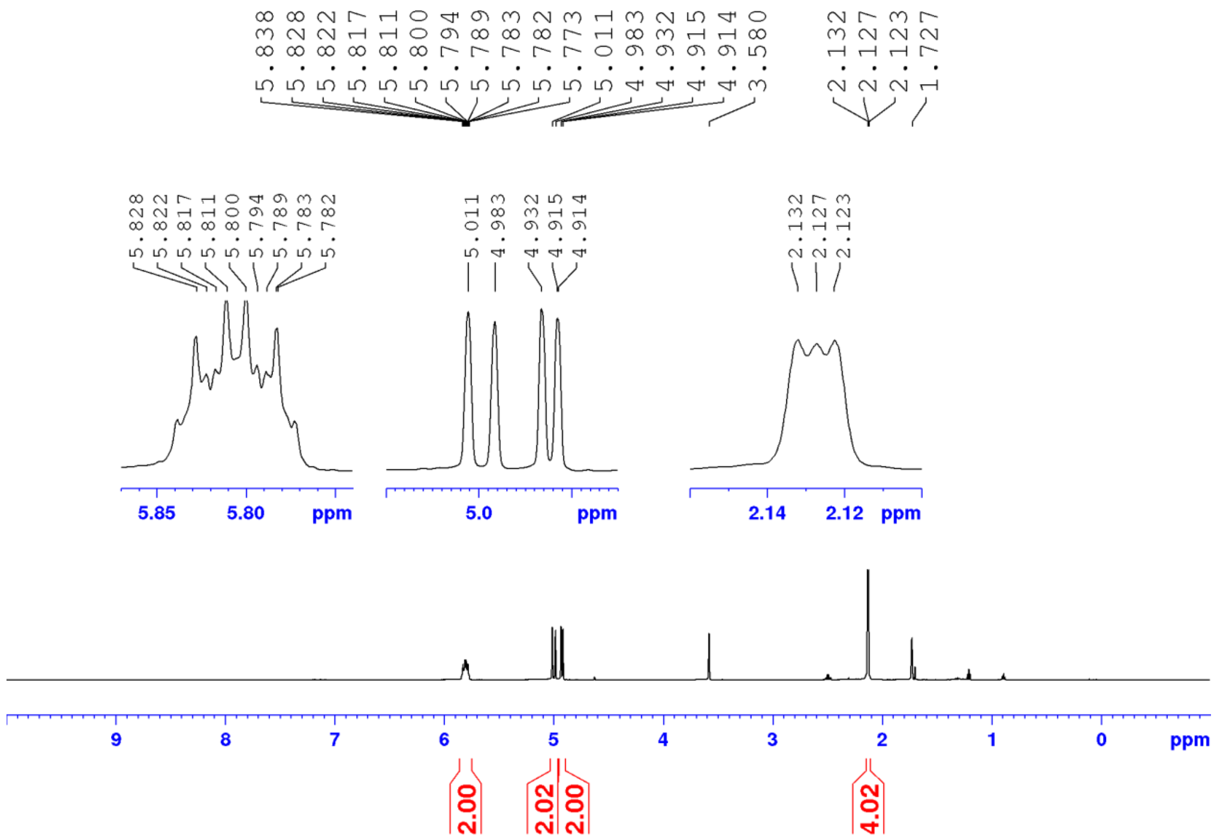

Figure 55: ^{1}H NMR (thf-d_8, 600 MHz, 295K) of 1,5-Hexadiene (5-E).

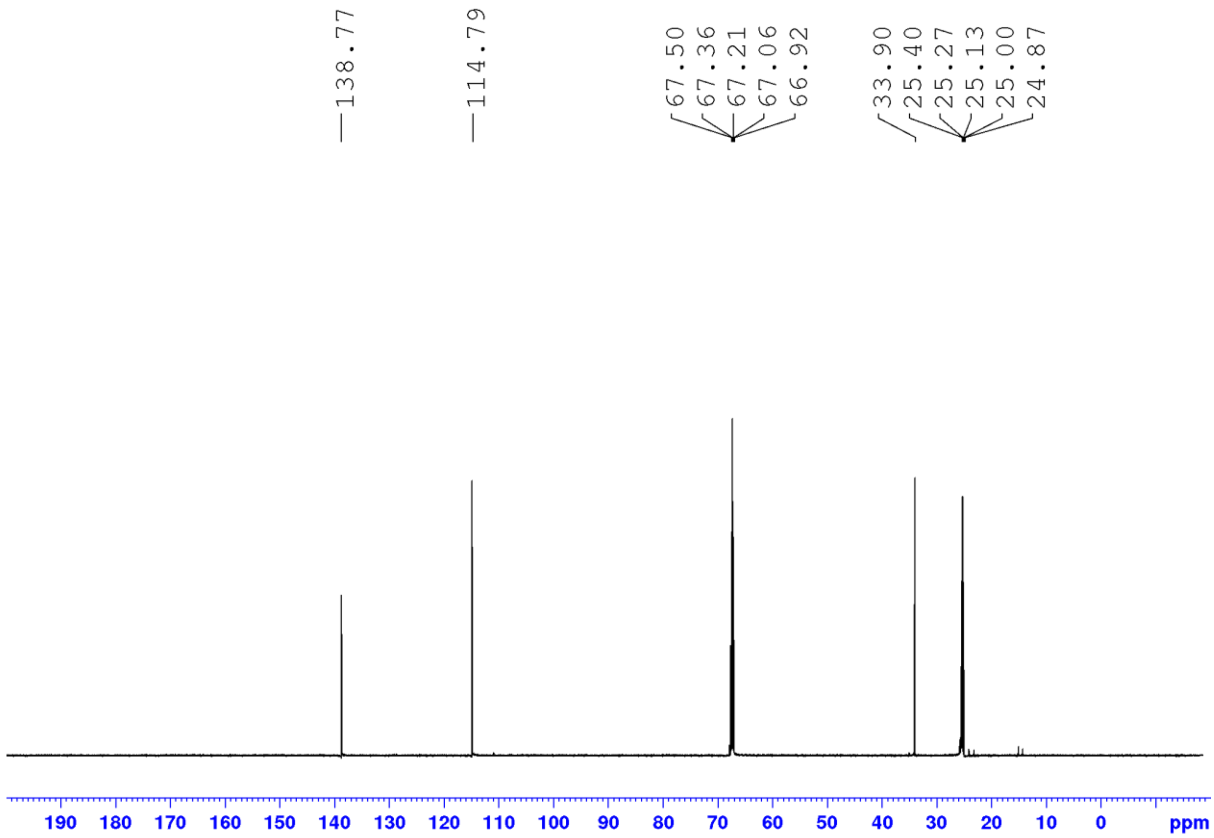

Figure 56: $^{13}C\{^{1}H\}$ NMR (thf-d_8, 151 MHz, 295K) of 1,5-Hexadiene (5-E).

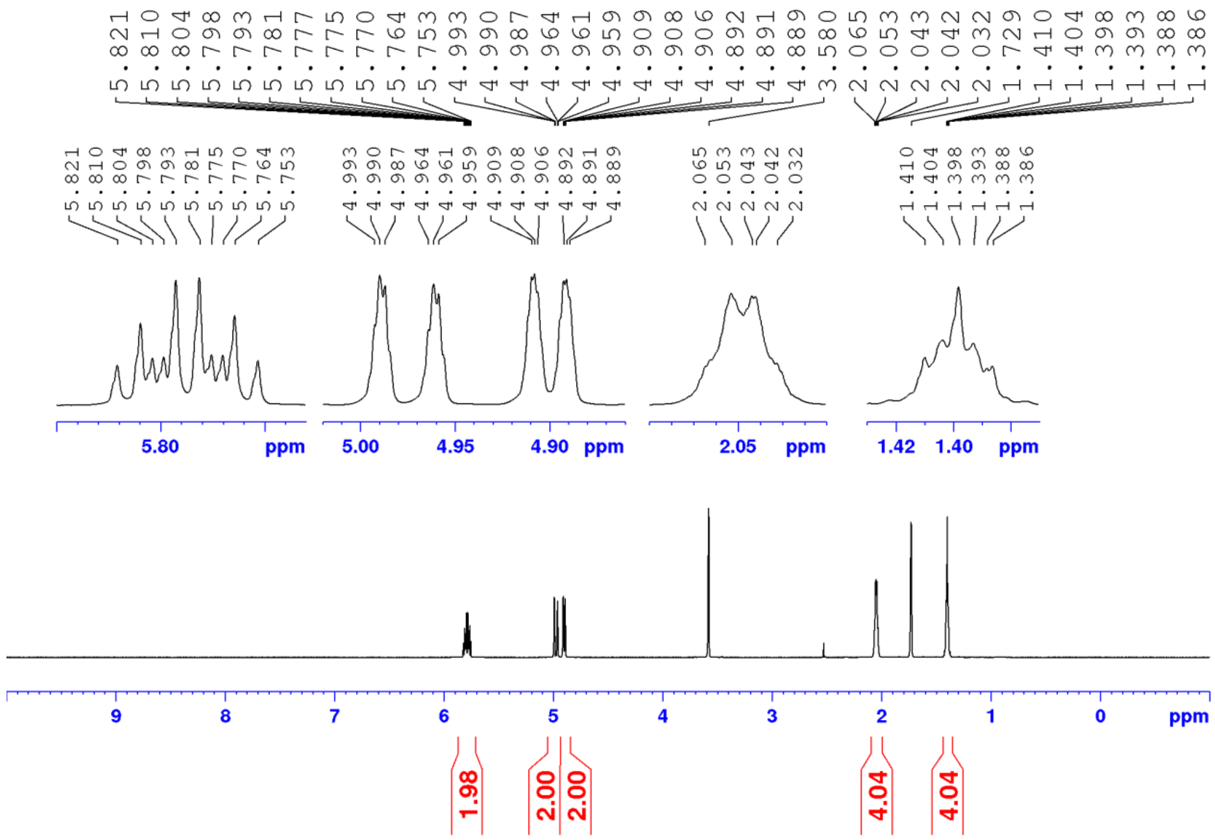

Figure 57: ^{1}H NMR (thf-d_8, 600 MHz, 295K) of 1,7-Octadiene (5-F).

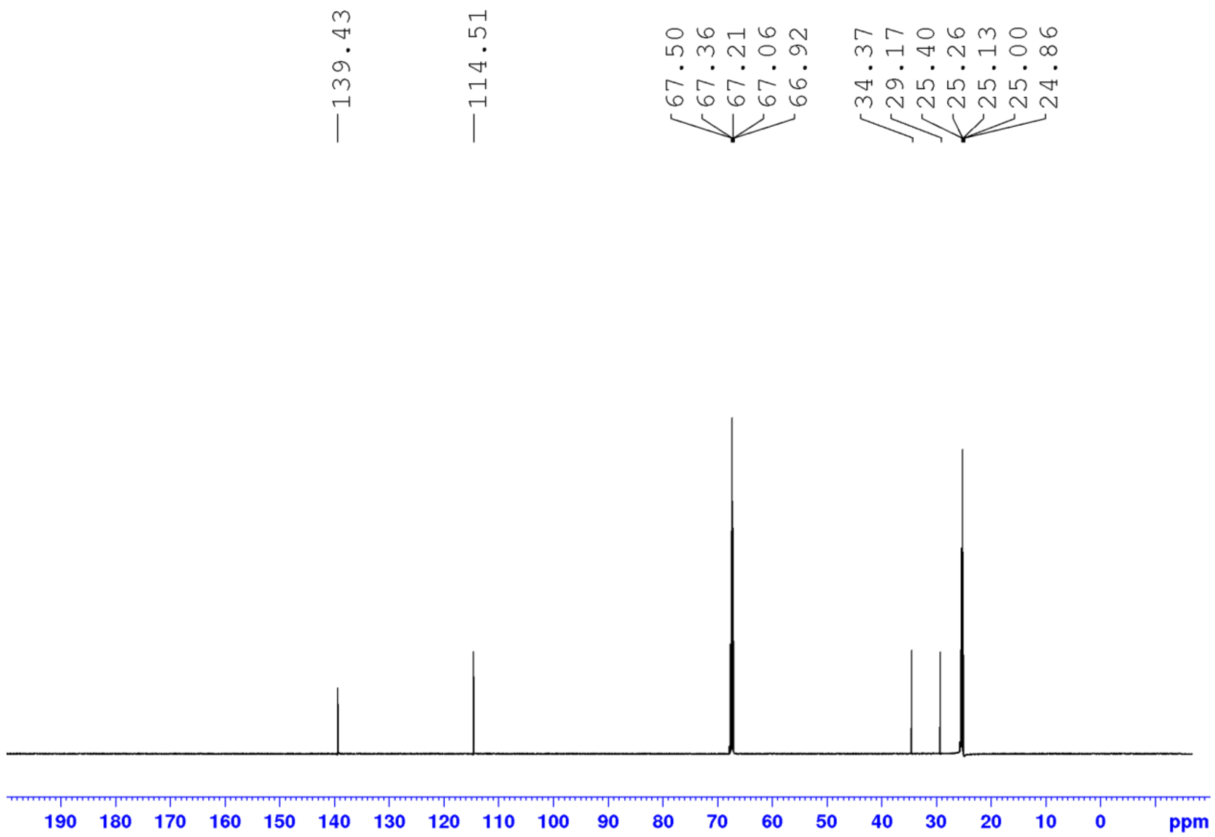

Figure 58: $^{13}C\{^{1}H\}$ NMR (thf-d_8, 151 MHz, 295K) of 1,7-Octadiene (5-F).

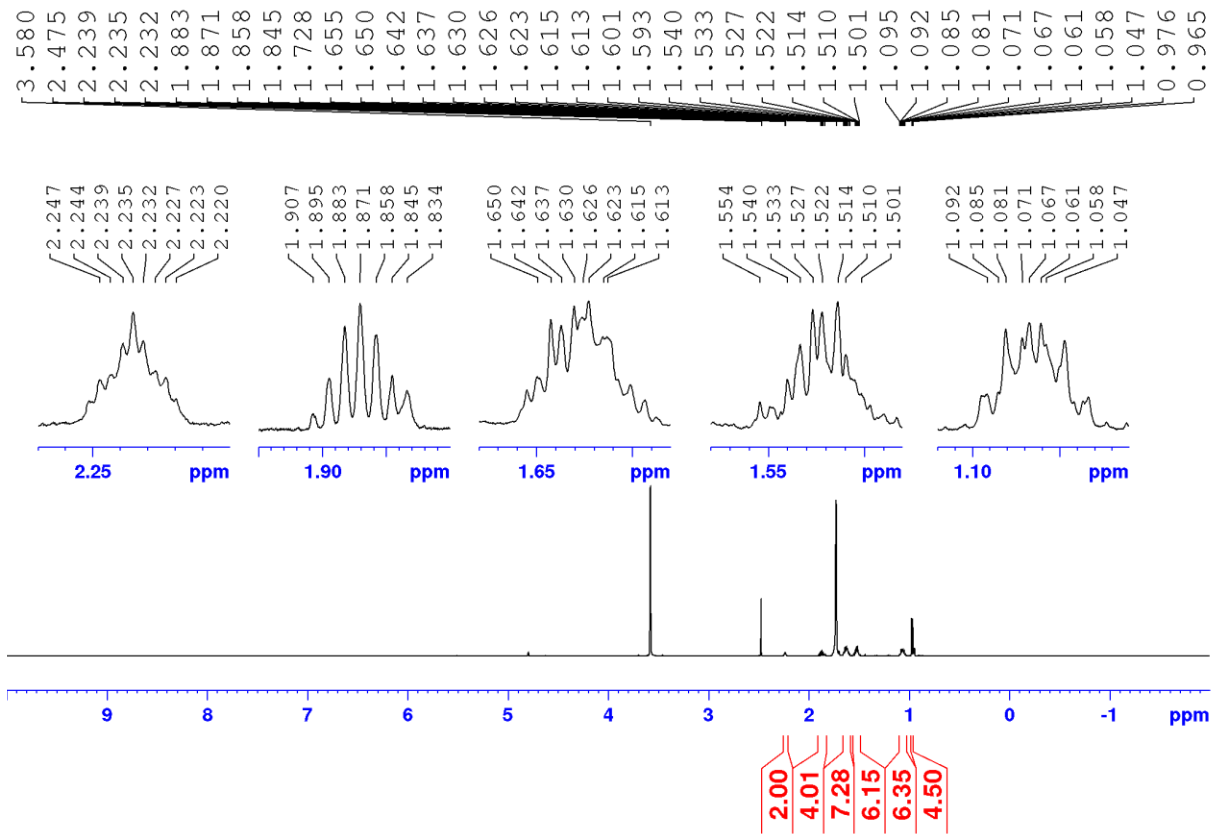

Figure 59: ^{1}H NMR (thf-d_8, 600 MHz, 295K) of 1,2-Dicyclopentylethane (5-G).

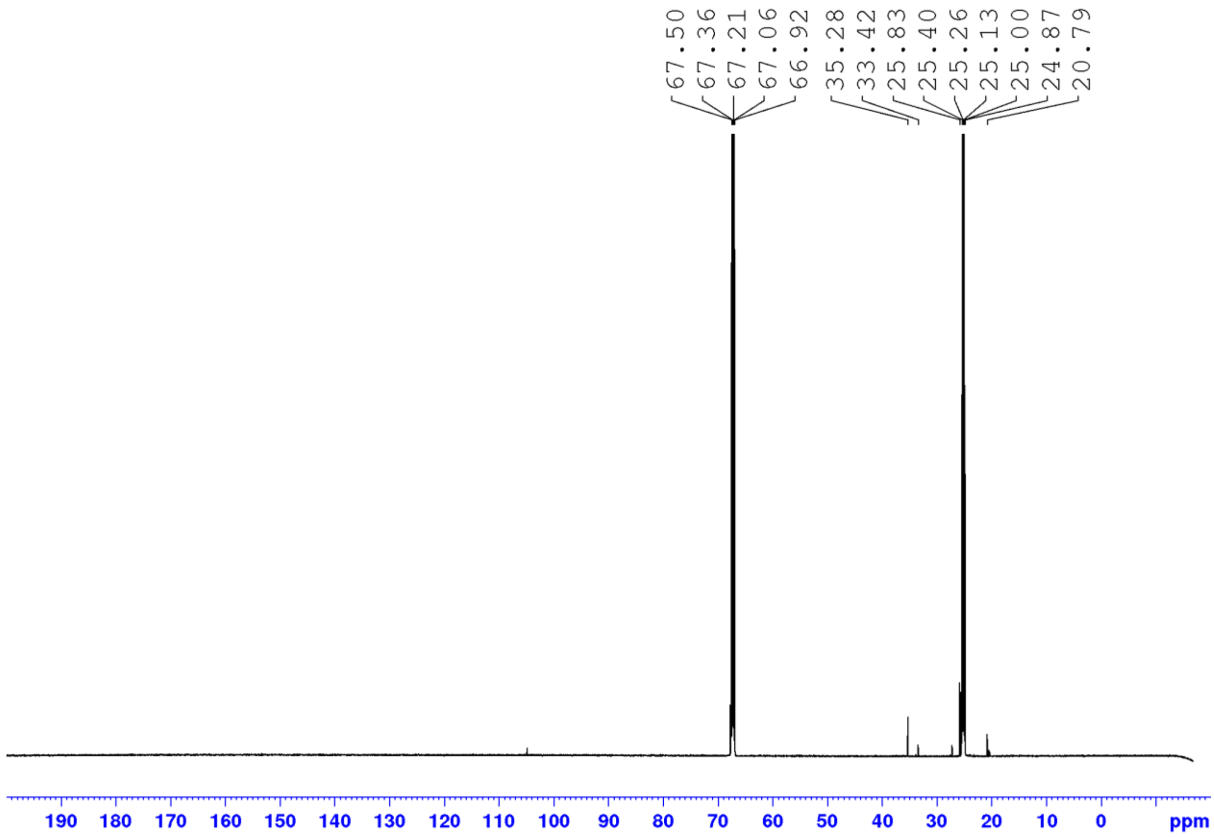

Figure 60: $^{13}C\{^{1}H\}$ NMR (thf-d_8, 151 MHz, 295K) of 1,2-Dicyclopentylethane (5-G).

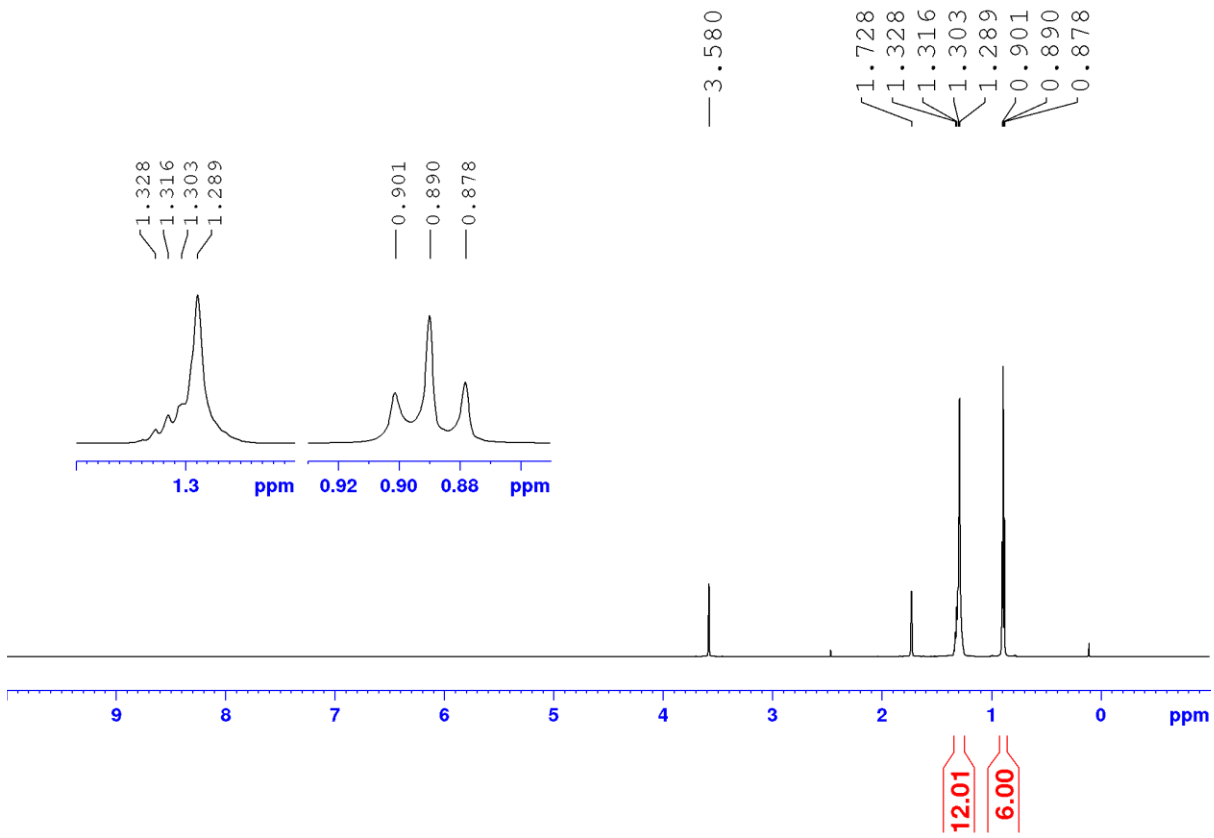

Figure 61: ^{1}H NMR (thf-d_8, 600 MHz, 295K) of *n*-Octane (5-H).

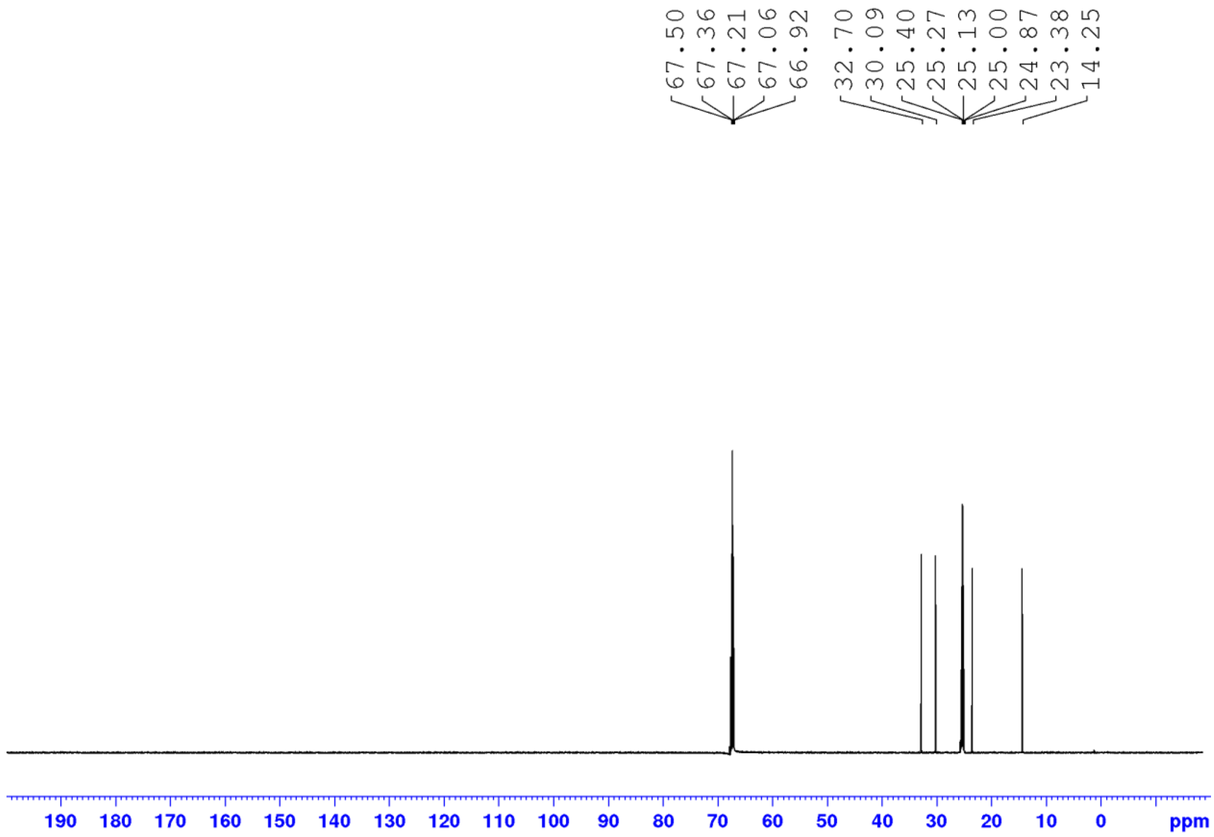

Figure 62: $^{13}C\{^{1}H\}$ NMR (thf-d_8, 151 MHz, 295K) of *n*-Octane (5-H).

3.) Variable Temperature NMR and DOSY experiments

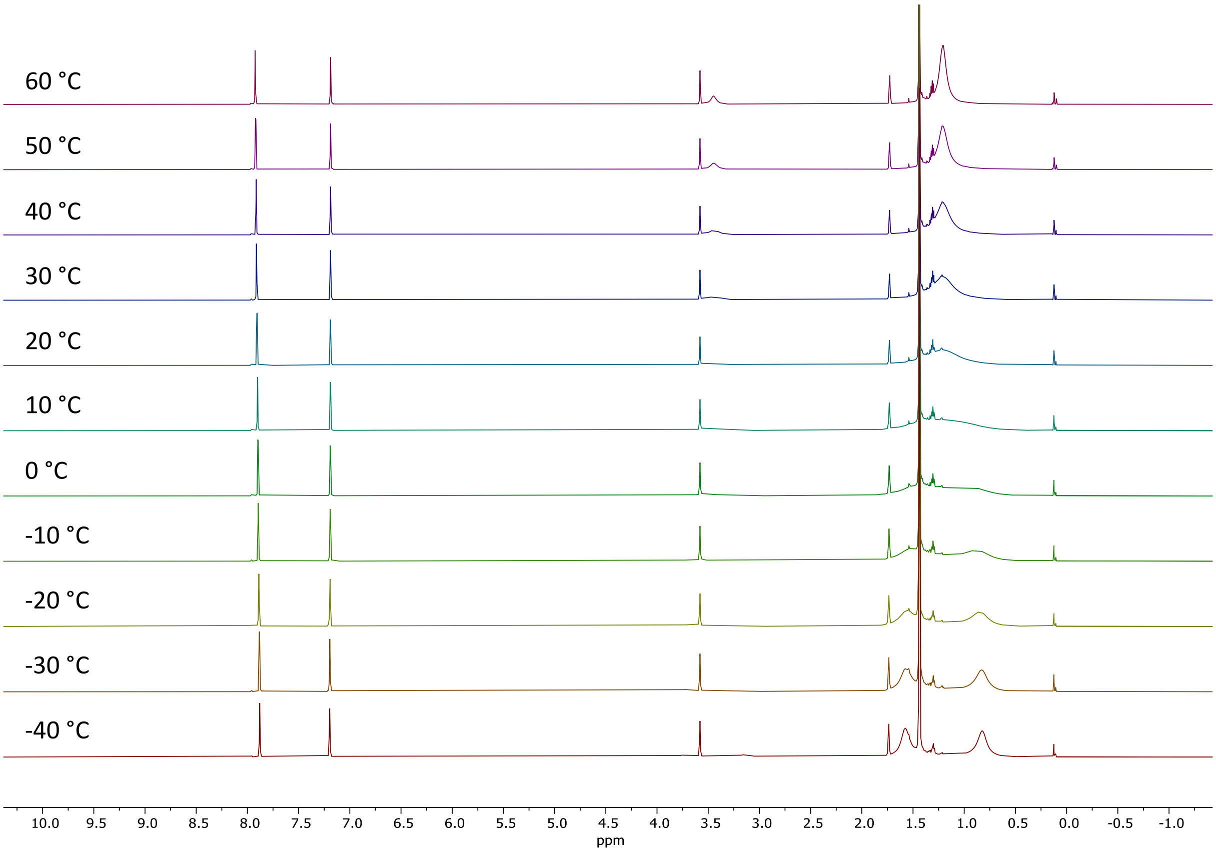

Figure 63: ^{1}H NMR (thf-d_8, 600 MHz) of $^{Cbz}[^{tBu}PNP]Pt^0Na$ (2-Na) at different temperatures.

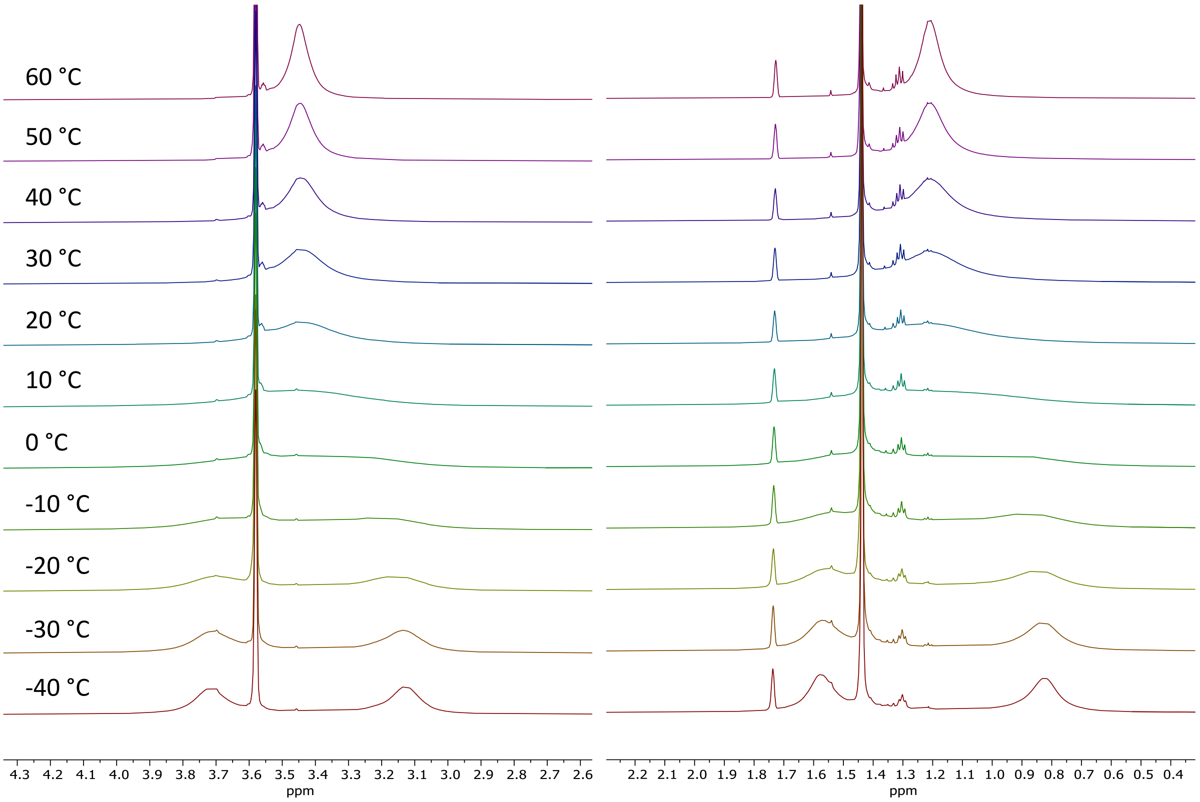

Figure 64: Excerpt from ^{1}H NMR (thf-d_8, 600 MHz) of $^{Cbz}[^{tBu}PNP]Pt^0Na$ (2-Na) at different temperatures.

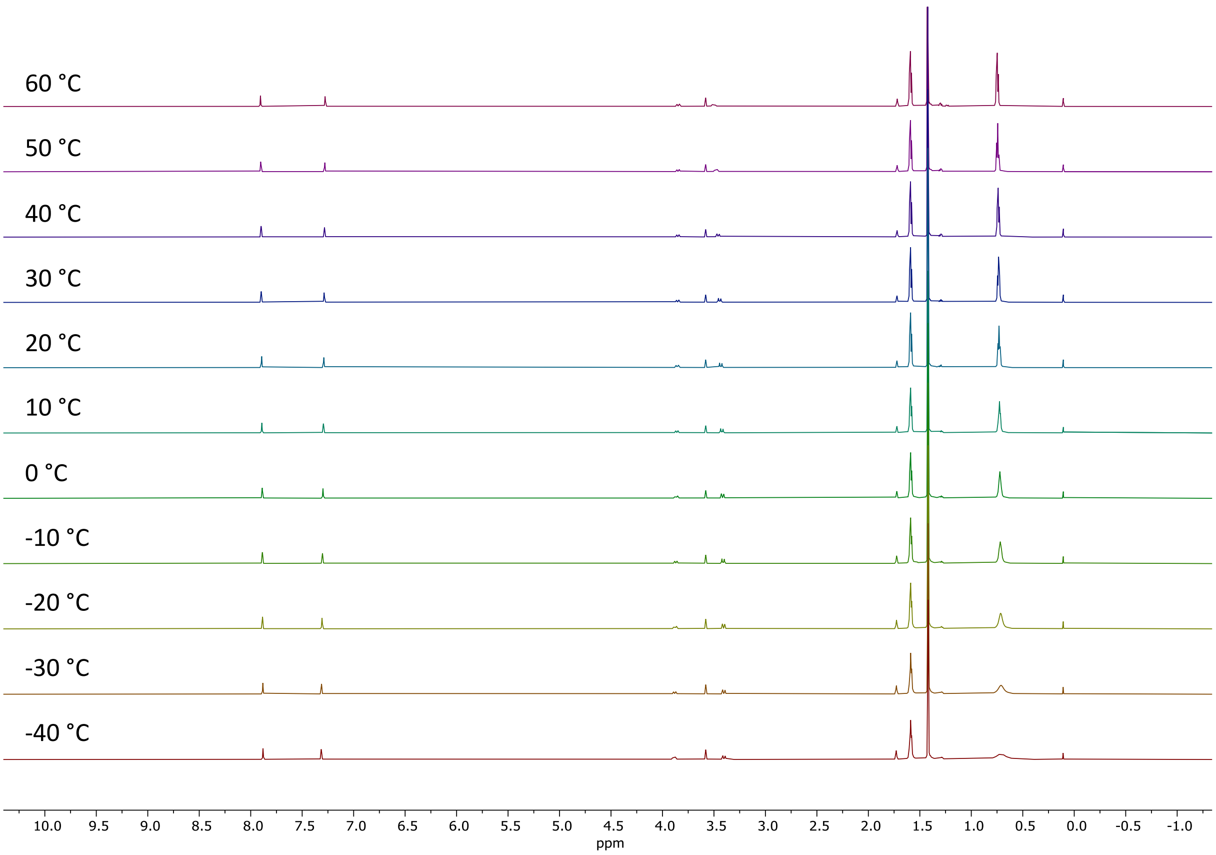

Figure 65: ^{1}H NMR (thf-d_8, 600 MHz) of ^{Cbz}[^{tBu}PNP]Pt^{0}MgCl (2-Mg) at different temperatures.

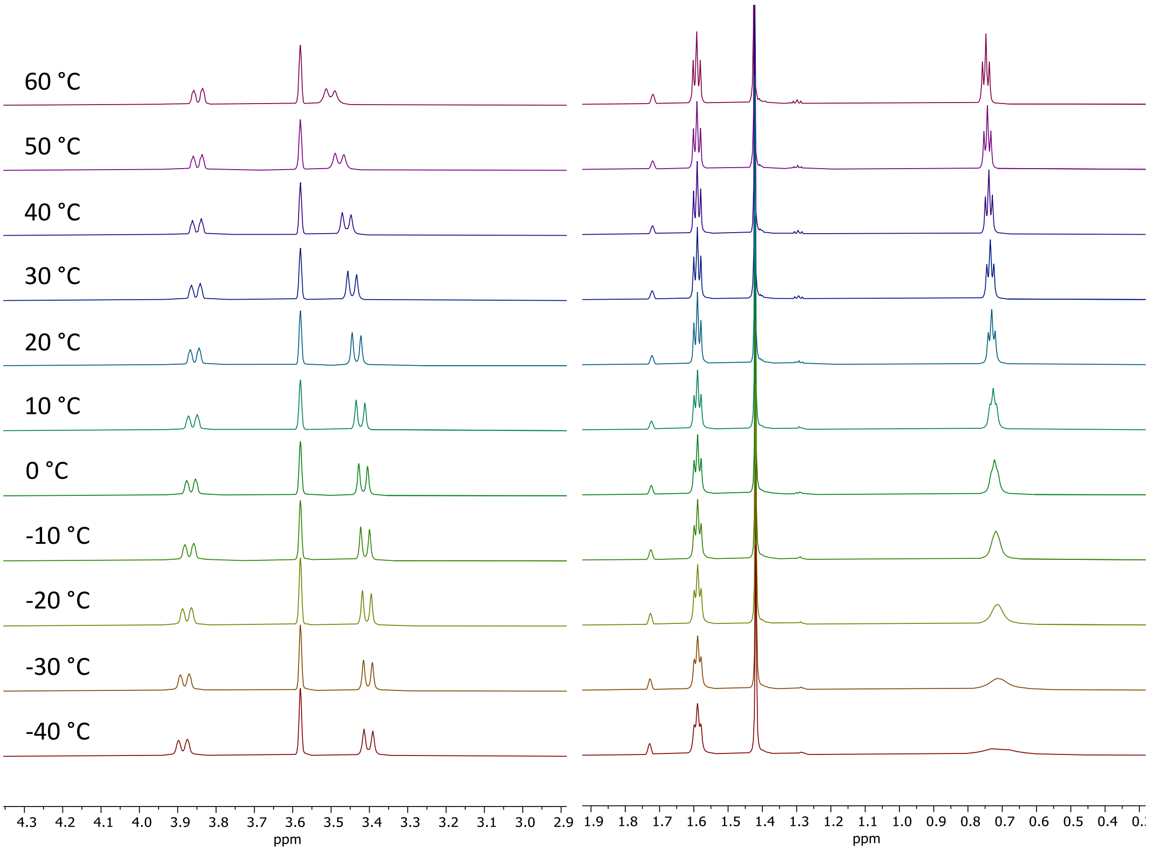

Figure 66: Excerpts from ^{1}H NMR (thf-d_8, 600 MHz) of ^{Cbz}[^{tBu}PNP]Pt^{0}MgCl (2-Mg) at different temperatures.

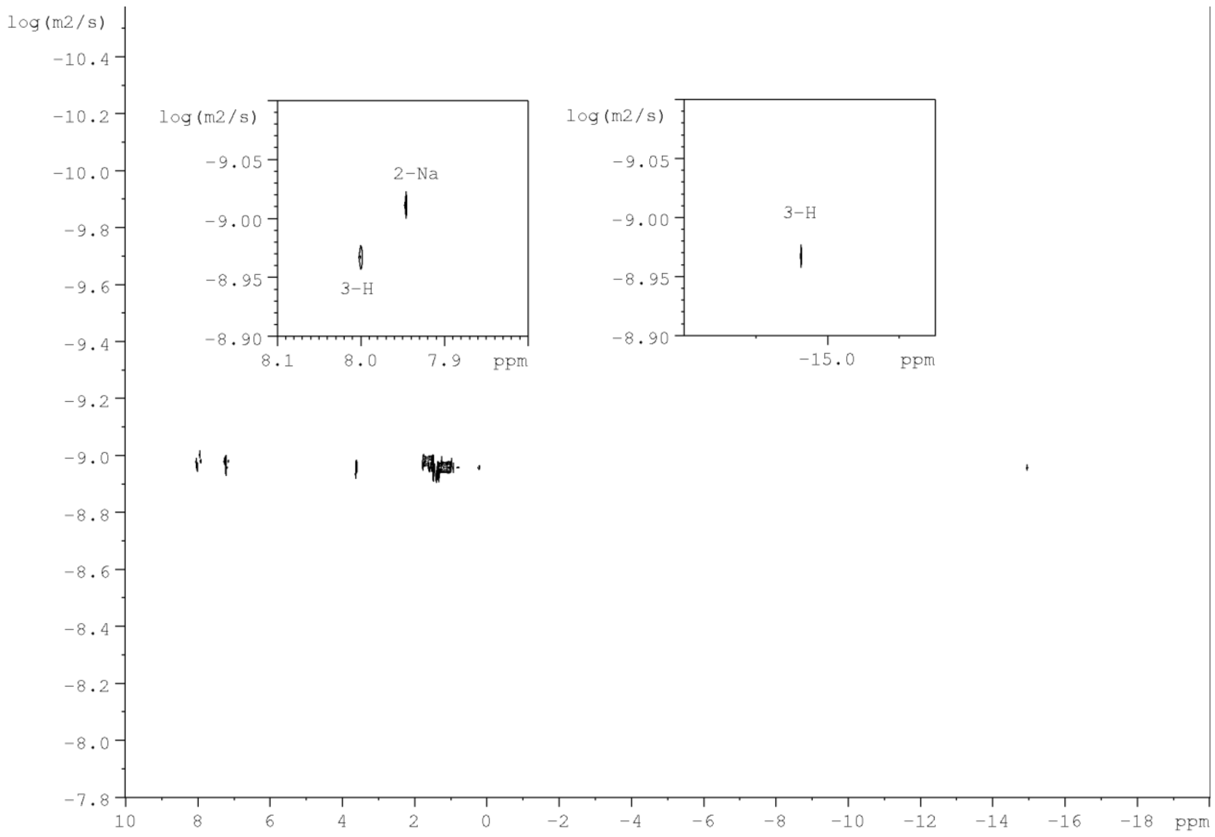

Figure 67: ^{1}H DOSY NMR (thf-d_8, 600 MHz, 295K) of 2-Na with 3-H as standard.

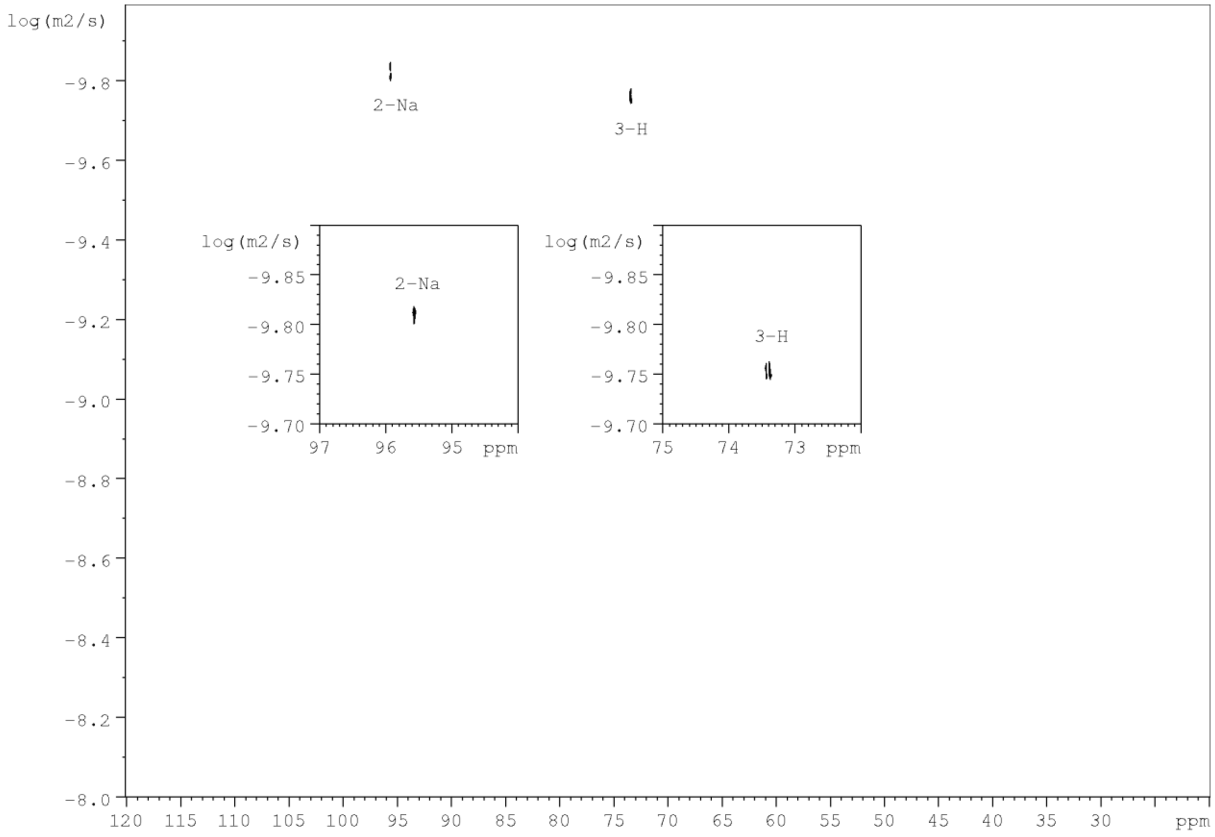

Figure 68: ^{31}P DOSY NMR (thf-d_8, 243 MHz, 295K) of 2-Na with 3-H as standard.

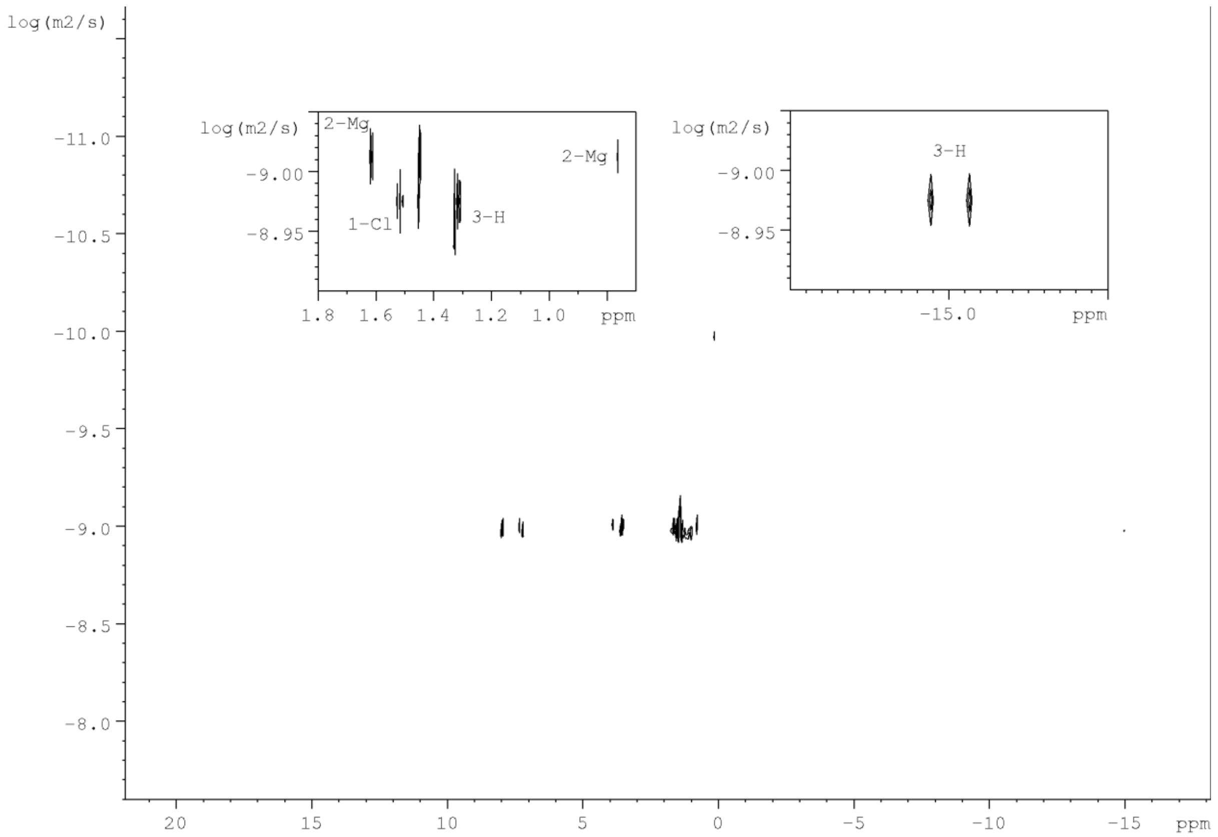

Figure 69: ^{1}H DOSY NMR (thf-d_8, 600 MHz, 295K) of 2-Mg with 3-H and 1-Cl as standard.

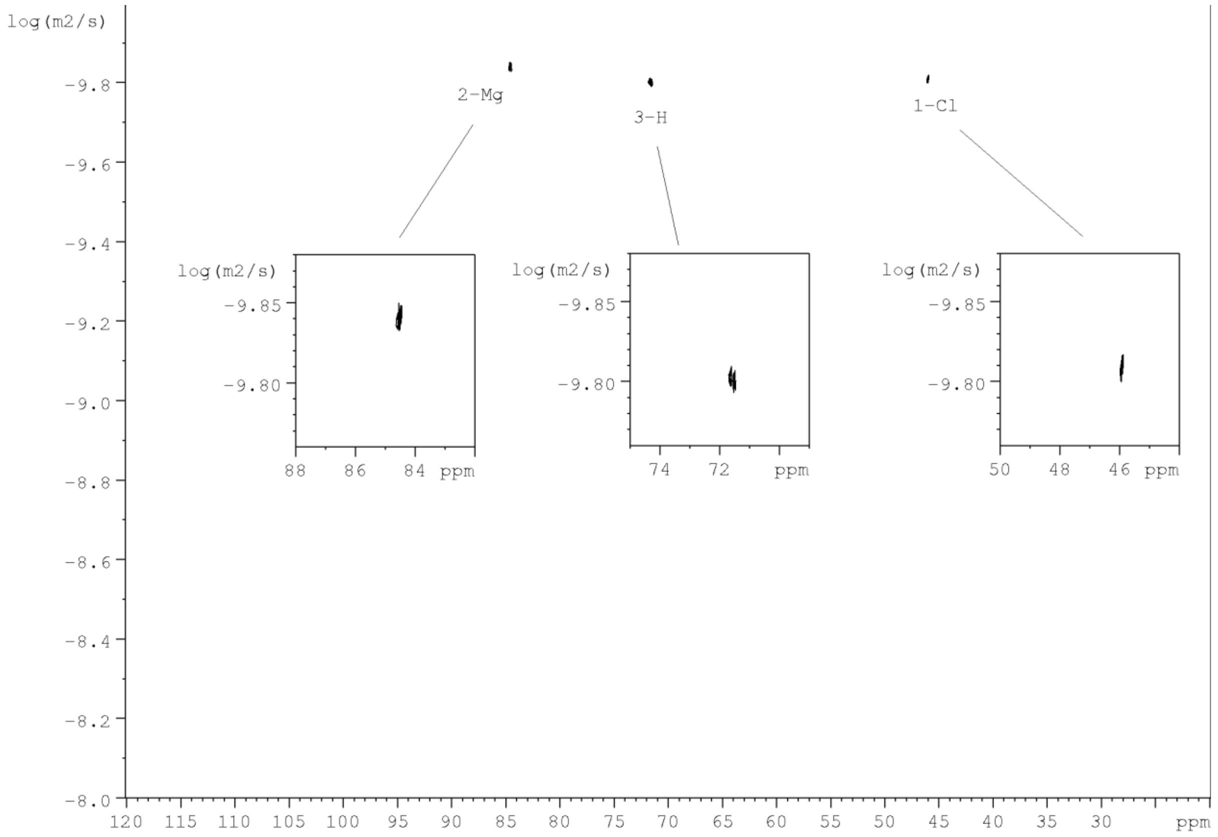

Figure 70: ^{31}P DOSY NMR (thf-d_8, 243 MHz, 295K) of 2-Mg with 3-H and 1-Cl as standard.

The signals in the DOSY (Diffusion-Ordered NMR Spectroscopy) spectra were assigned based on the known resonances from the corresponding 1D spectra. In addition, the hydride signal is shown in an enlarged view to confirm the consistency of the signal assignment within the backbone. For the measurement of **2-Na**, **3-H** was used as a reference, and for the measurement of **2-Mg**, **3-H** was likewise used (with **1-Cl** additionally included for comparison). From the difference in the signals of the respective species, the ratio of the diffusion coefficients can be determined. According to the Stokes-Einstein equation, D is proportional to 1/r, and the radius in turn correlates with the molecular volume. The obtained values show that the reduced species **2-Na** and **2-Mg** exhibit nearly identical, slower diffusion compared to **3-H**, and thus possess nearly identical larger radii/volumes than **3-H**. Based on these findings, it can be concluded that **2-Na** is definitively present as a monomer in solution as it is the case for **2-Mg**.

Evaluation of Diffusion-Ordered NMR Spectroscopy (DOSY) for 2-Na with 2-H as standard:

^{1}H log D = -8.99 (2-Na) log D = -8.95 (3-H) Δ(log D) = -0.04

Diffusion Ratio: $D_{2\text{-Na}}/D_{3\text{-H}} = 10^{\Delta(\log D)} = 0.91$ -> 2-Na diffuses 9 % slower

Radius Ratio ($r \propto D^{-1}$) $r_{2\text{-Na}}/r_{3\text{-H}} = 1/0.91 = 1.10$ -> 10 % larger for 2-Na

Volume Ratio ($V \propto r^3$) $V_{2\text{-Na}}/V_{3\text{-H}} = 1.10^3 = 1.33$ -> 33 % larger for 2-Na

^{31}P log D = -9.81 (2-Na) log D = -9.76 (3-H) Δ(log D) = -0.05

Diffusion Ratio: $D_{2\text{-Na}}/D_{3\text{-H}} = 10^{\Delta(\log D)} = 0.89$ -> 2-Na diffuses 11 % slower

Radius Ratio ($r \propto D^{-1}$) $r_{2\text{-Na}}/r_{3\text{-H}} = 1/0.89 = 1.12$ -> 12 % larger for 2-Na

Volume Ratio ($V \propto r^3$) $V_{2\text{-Na}}/V_{3\text{-H}} = 1.10^3 = 1.40$ -> 40 % larger for 2-Na

Evaluation of Diffusion-Ordered NMR Spectroscopy (DOSY) for 2-Mg with 2-H as standard:

^{1}H log D = -9.01 (2-Mg) log D = -8.98 (3-H) Δ(log D) = -0.03

Diffusion Ratio: $D_{2\text{-Mg}}/D_{3\text{-H}} = 10^{\Delta(\log D)} = 0.93$ -> 2-Mg diffuses 7 % slower

Radius Ratio ($r \propto D^{-1}$) $r_{2\text{-Mg}}/r_{3\text{-H}} = 1/0.91 = 1.08$ -> 8 % larger for 2-Mg

Volume Ratio ($V \propto r^3$) $V_{2\text{-Mg}}/V_{3\text{-H}} = 1.10^3 = 1.26$ -> 26 % larger for 2-Mg

^{31}P log D = -9.84 (2-Mg) log D = -9.80 (3-H) Δ(log D) = -0.04

Diffusion Ratio: $D_{2\text{-Mg}}/D_{3\text{-H}} = 10^{\Delta(\log D)} = 0.91$ -> 2-Mg diffuses 9 % slower

Radius Ratio ($r \propto D^{-1}$) $r_{2\text{-Mg}}/r_{3\text{-H}} = 1/0.91 = 1.10$ -> 10 % larger for 2-Mg

Volume Ratio ($V \propto r^3$) $V_{2\text{-Mg}}/V_{3\text{-H}} = 1.10^3 = 1.33$ -> 33 % larger for 2-Mg

4.) EPR spectra

For *in situ* EPR detection of the platinum(I) intermediate, a solution of **2-Mg** in THF was frozen in an EPR tube using liquid nitrogen and *n*-pentane. Under an argon atmosphere, the tube was opened and a solution of benzyl bromide in THF was added and frozen immediately. The sample from both superimposed frozen solutions was precooled with liquid nitrogen and introduced into the EPR spectrometer. No signals were detected at the initial 4 K. The sample was then slowly heated in the device in increments of 5 K. At approximately 170 K, both phases slowly mixed, enabling the detection of the platinum(I) species **2*** and the benzyl radical. Upon further heating after the measurement, both signals disappeared again. After removing the sample from the spectrometer, the expected color change from red to yellow-orange with the formation of a colorless precipitate could be observed, indicating that the reaction was complete. The measured EPR spectrum (Figure 67) and the corresponding simulation (Figure 68) are shown below.

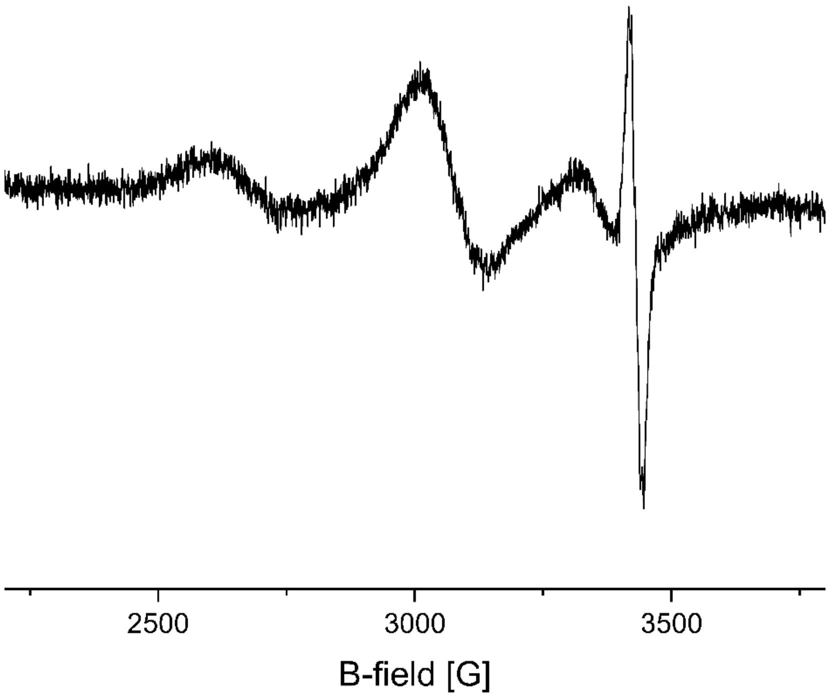

Figure 71: EPR spectrum of the platinum(I) intermediate **2*** in THF at 170 with following experimental parameters: Frequency 9.626507 GHz, B_0 3000.00 G, Sweep 2500 G, Time 20.97 s, Steps 4096, Modulation 5.000 G, Power atten 18 dB, Gain 60 dB.

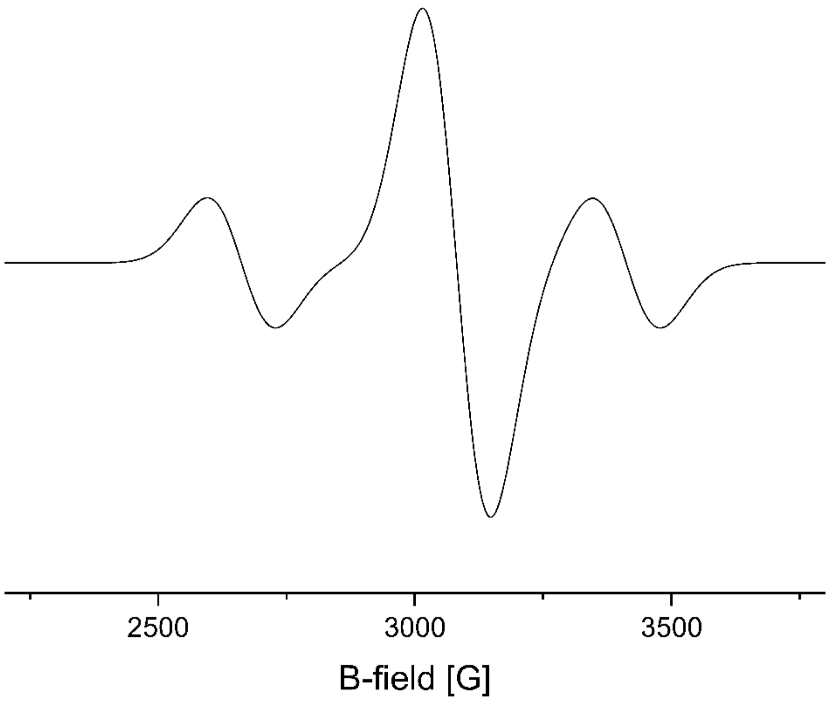

Figure 72: Simulated EPR spectrum of the platinum(I) intermediate **2***: g_{iso} = 2.21853, A_{iso}(^{195}Pt) = 2124 G, LWPP = 13.6589.

The trityl radical (**5-A'•**) formed during the reaction of **2-Na/Mg** with trityl bromide (**S3**) was also detected by EPR spectroscopy at room temperature (Figure 69). Complex **1-Br** was first removed by crystallization and washing with cold *n*-pentane.

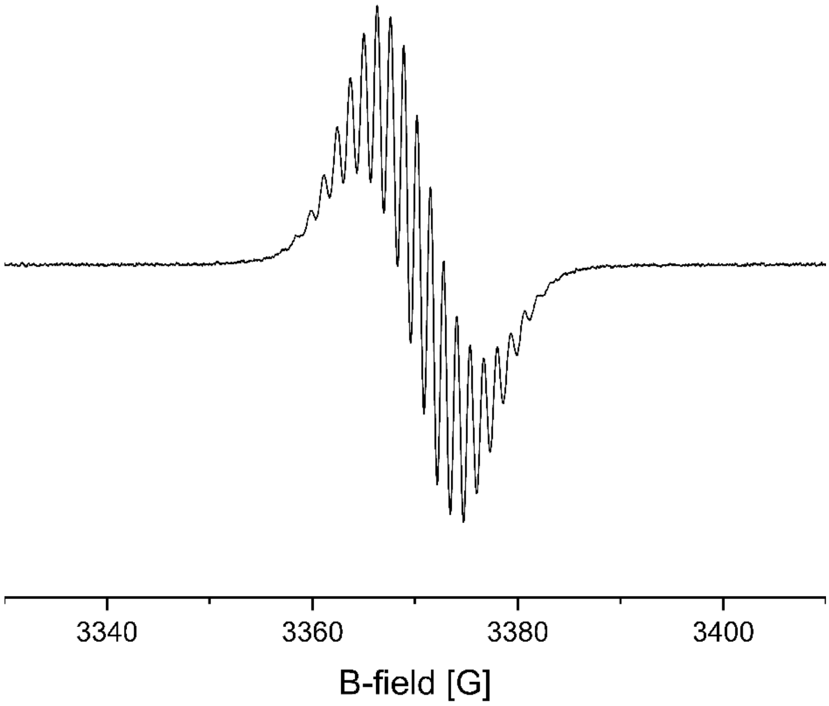

Figure 73: EPR spectrum of Tritylradical in benzene at 298 K measured with following experimental parameters: Frequency 9.443350 GHz, B_0 3373.64 G, Sweep 97.52 G, Time 120 s, Steps 4096, Modulation 50 mG, MW atten 10 dB, Gain 2E2.

5.) IR spectra

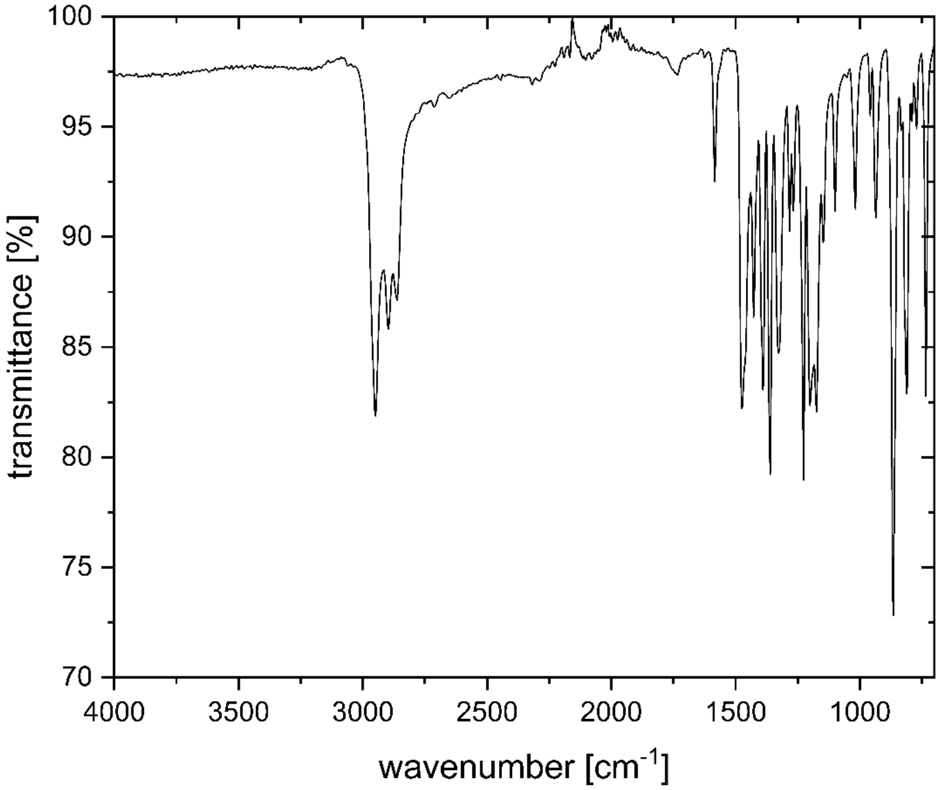

Figure 74: IR (ATR) spectrum of $^{Cbz}[^{tBu}PNP]Pt^{II}Cl$ (1-Cl) at 298 K.

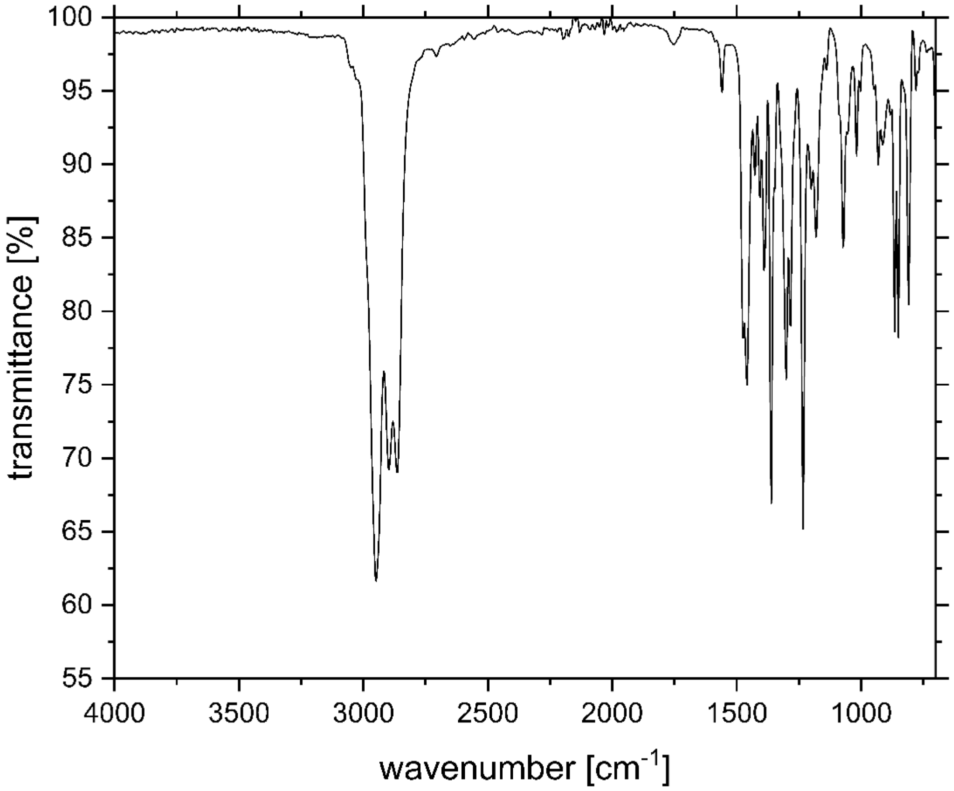

Figure 75: IR (ATR) spectrum of $^{Cbz}[^{tBu}PNP]Pt^{0}Na$ (2-Na) at 298 K.

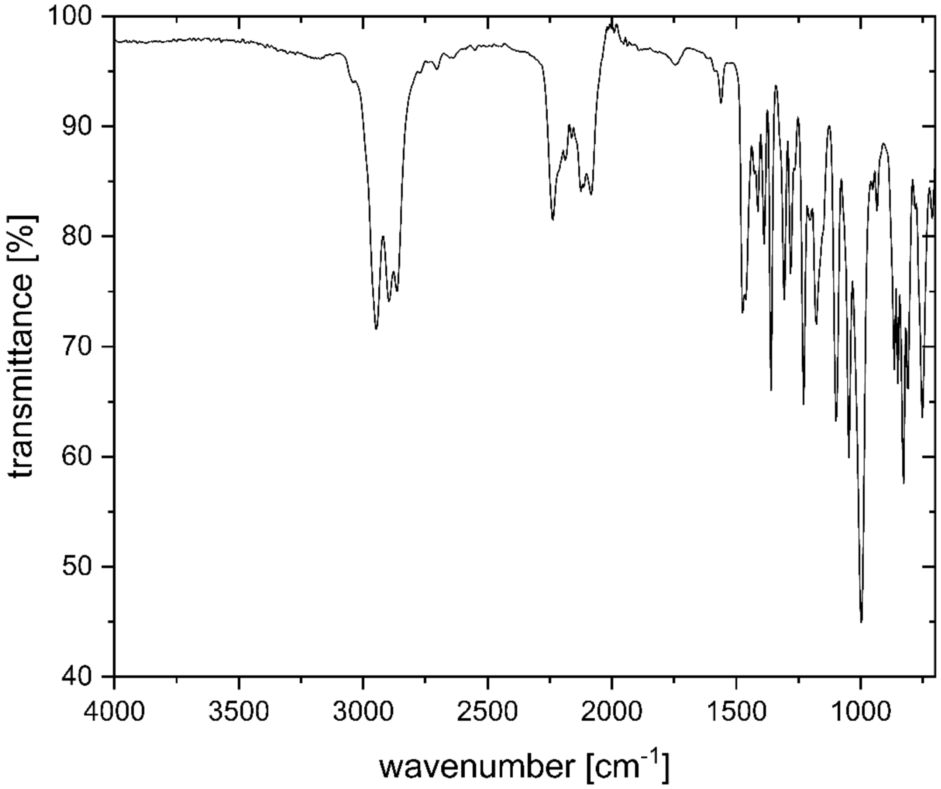

Figure 76: IR (ATR) spectrum of $^{Cbz}[^{tBu}PNP]Pt^0MgCl(thf\text{-}d_8)_2$ (2-Mg) at 298 K.

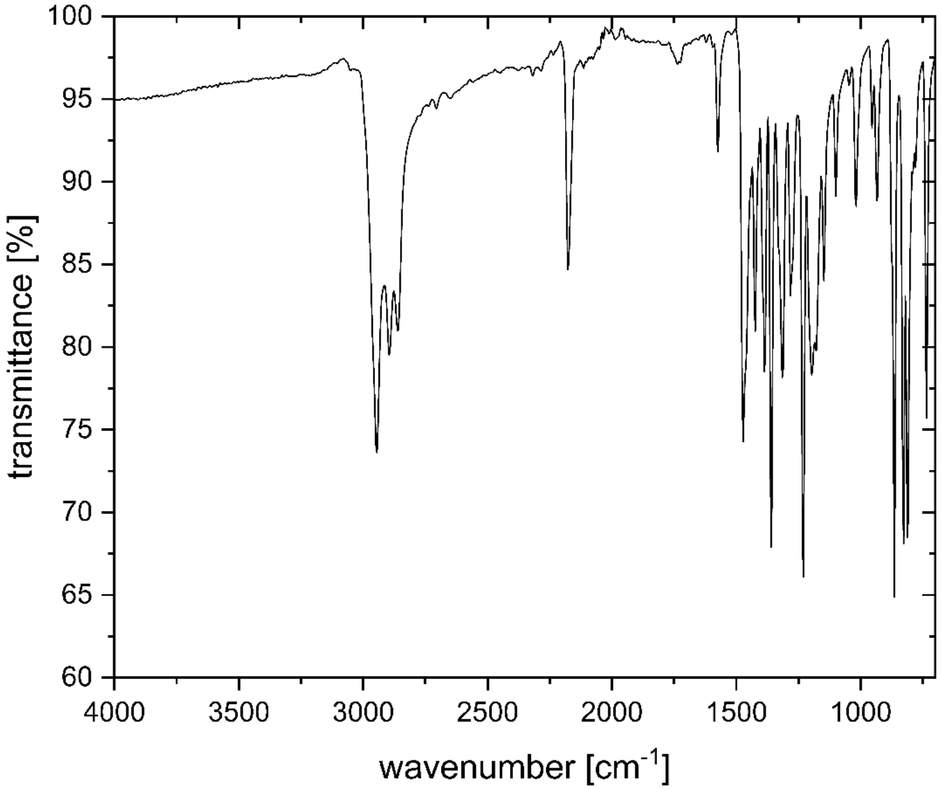

Figure 77: IR (ATR) spectrum of $^{Cbz}[^{tBu}PNP]Pt^{II}H$ (3-H) at 298 K.

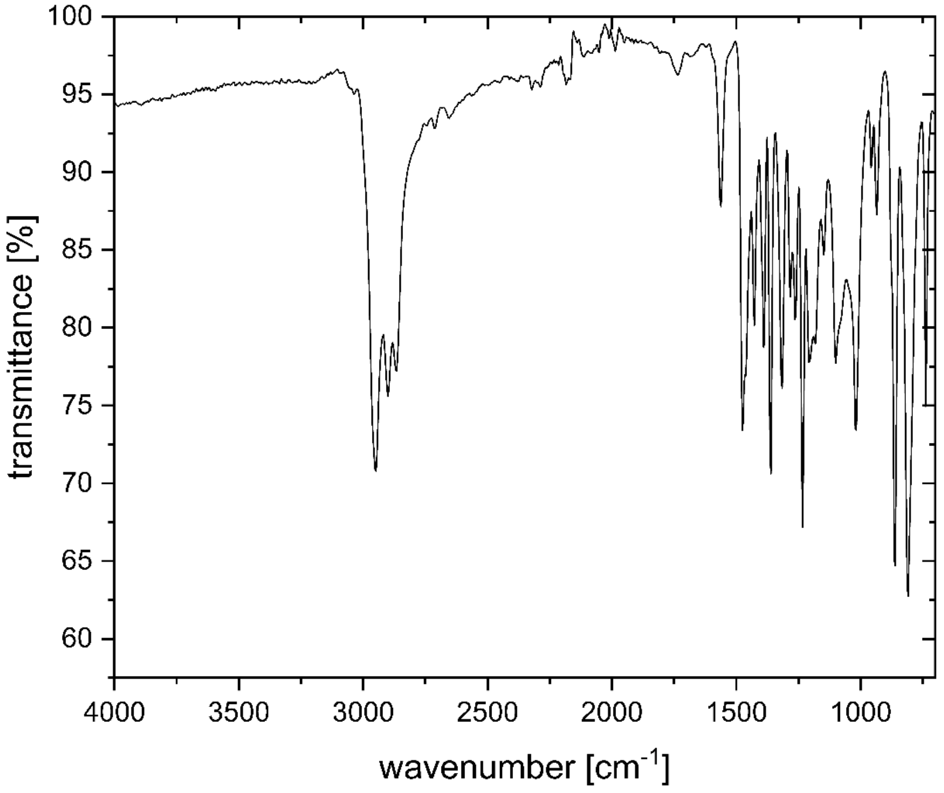

Figure 78: IR (ATR) spectrum of $^{Cbz}[^{tBu}PNP]Pt^{II}D$ (3-D) at 298 K.

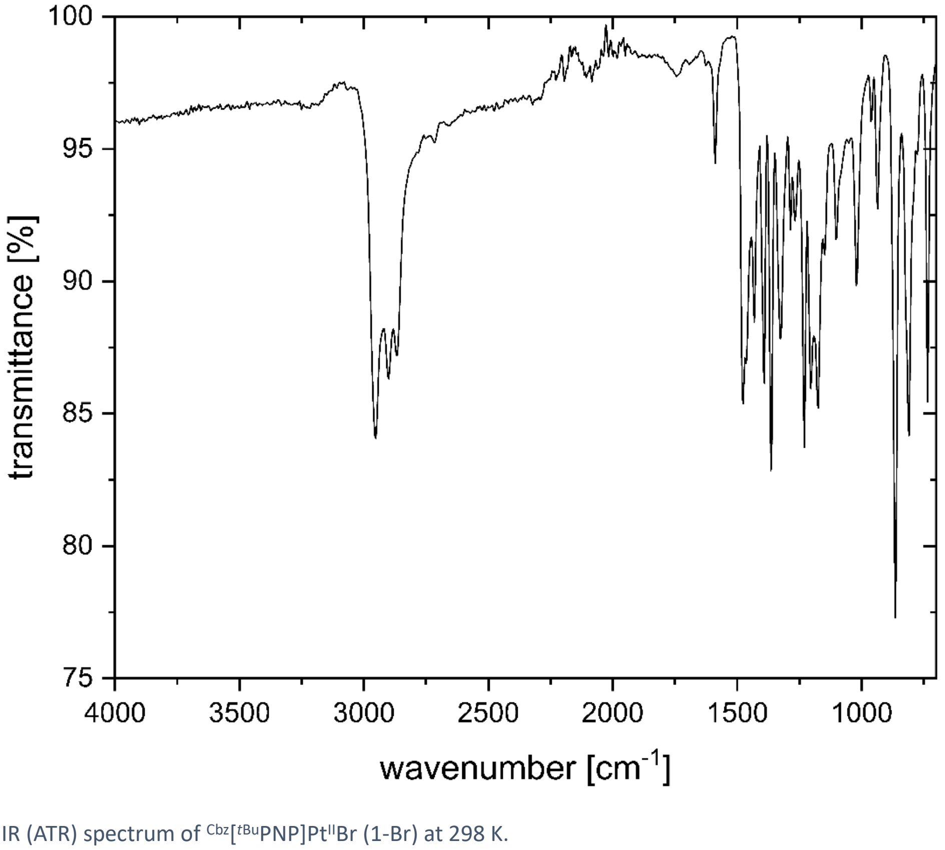

Figure 79: IR (ATR) spectrum of $^{Cbz}[^{tBu}PNP]Pt^{II}Br$ (1-Br) at 298 K.

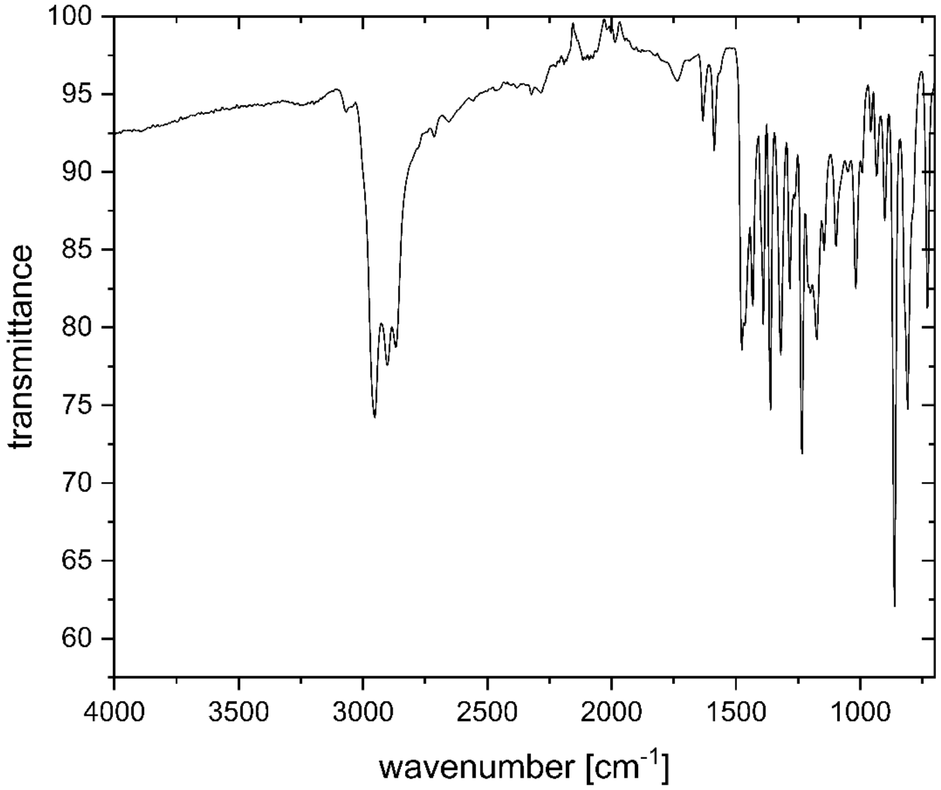

Figure 80: IR (ATR) spectrum of $^{Cbz}[^{tBu}PNP]Pt^{II}CH_2CH_2CH{=}CH_2$ (4a) at 298 K.

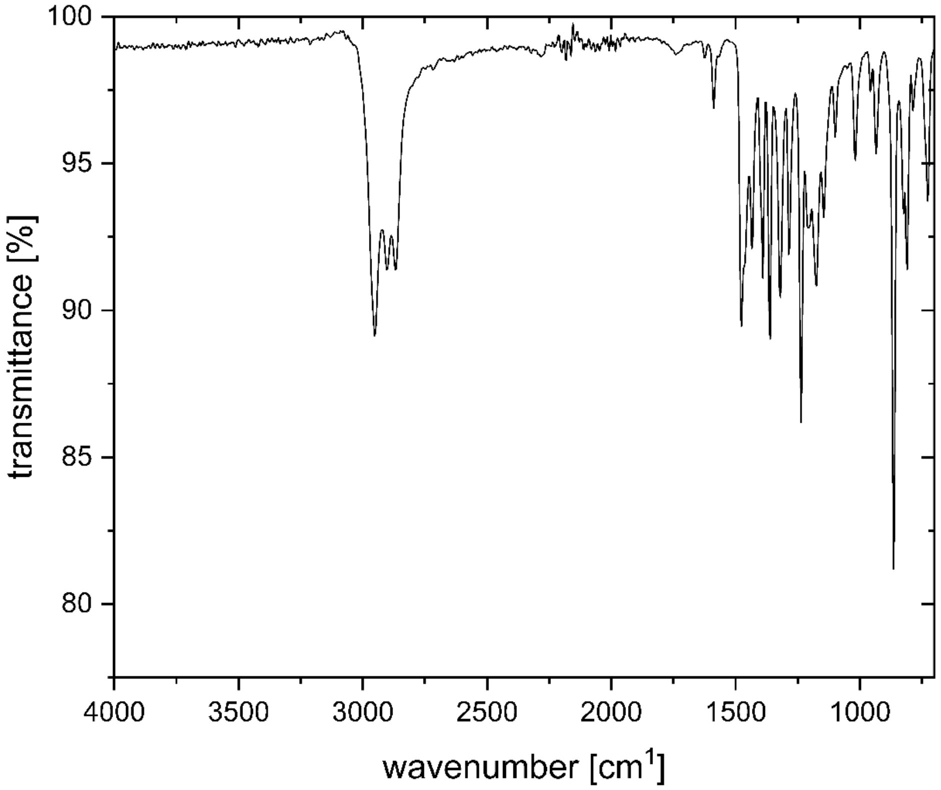

Figure 81: IR (ATR) spectrum of $^{Cbz}[^{tBu}PNP]Pt^{II}CH_2CH(CH_2)_4$ (4b) at 298 K.

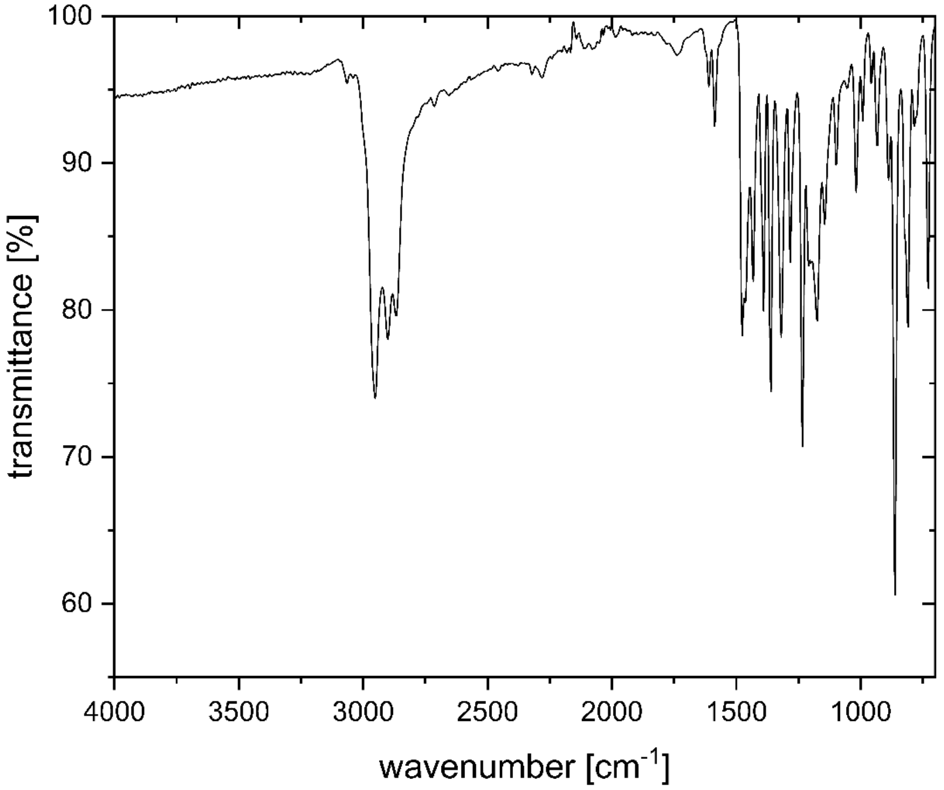

Figure 82: IR (ATR) spectrum of $^{Cbz}[^{tBu}PNP]Pt^{II}CH_2CH{=}CH_2$ (4c) at 298 K.

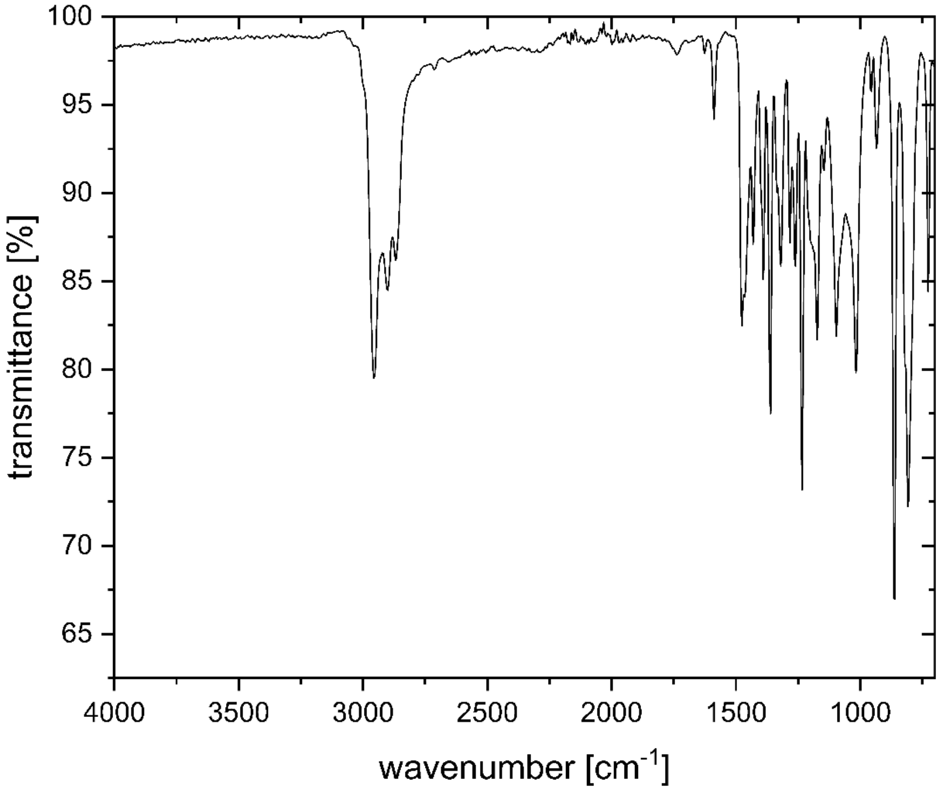

Figure 83: IR (ATR) spectrum of $^{Cbz}[^{tBu}PNP]Pt^{II}CH_2CH_2CH_2CH_3$ (4d) at 298 K.

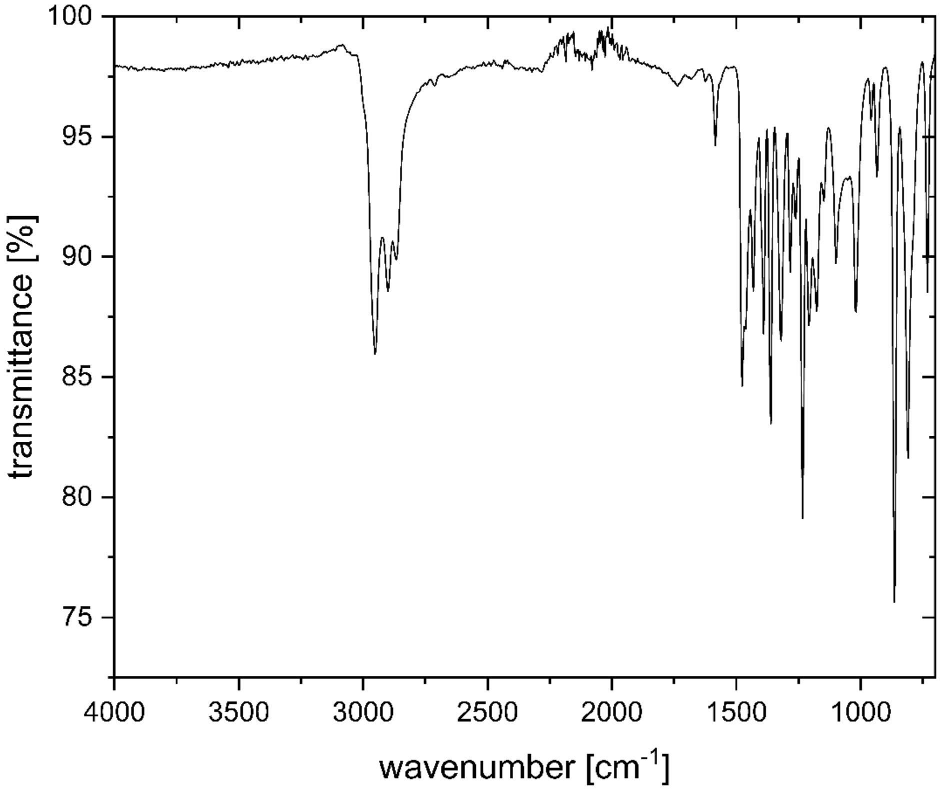

Figure 84: IR (ATR) spectrum of ^{Cbz}[^{tBu}PNP]Pt^{II}CH$_3$ (4e) at 298 K.

6.) X-ray data

Crystal data and details of the structure determinations are compiled in Table 4-9. Full shells of intensity data were collected at 120(2) K with an Agilent Technologies Supernova-E CCD diffractometer (Cu-K_α radiation, microfocus X-ray tube, multilayer mirror optics). Detector frames (ω-scans, 1.0° width) were integrated by profile fitting.[9] Data were corrected for air and detector absorption, Lorentz and polarization effects[10-11] and scaled essentially by application of appropriate spherical harmonic functions.[12-14] Absorption by the crystal was treated numerically (Gaussian grid).[14-15] An illumination correction was performed as part of the numerical absorption correction.[14]

Using OLEX2,[16] the structures were solved with SHELXT[17] and refined with SHELXL[18] by full-matrix least squares methods based on F^2 against all unique reflections. Final refinement was performed in the SHELXle gui.[19] All non-hydrogen atoms were refined with anisotropic displacement parameters. Hydrogen atoms bond to carbon atoms were set at calculated positions and refined with a riding model.[20-23] Split atom models were used to refine disordered groups and/or solvent molecules.[20-24] The CIF file were generated using FinalCif.[25]

CCDC 2530297-2530302 contains the supplementary crystallographic data for this paper. These data can be obtained free of charge from the Cambridge Crystallographic Data Centre's and FIZ Karlsruhe's joint Access Service via https://www.ccdc.cam.ac.uk.

For **2-Mg**: The crystal crystallizes as an inversion twin. The fractional contribution of the minor domain refined to 0.482(10). The structure crystallizes with three thf molecules in the asymmetric unit. One of these solvent molecules and one of the donating thf molecules are disordered. The disorder was refined with distance restraints and restraints for the anisotropic displacement parameters. The occupancies of the minor component refined to 0.216(11) and 0.353(19).

For **3-H**: One *tert*-butyl groups is disordered. The disorder was refined with distance restraints and restraints for the anisotropic displacement parameters. The occupancy of the minor component refined to 0.39(2). The H atom bond to the Pt atom was refined freely.

For **1-Br**: **1-Br** co-crystallized with **1-Cl**. The occupancy of **1-Cl** refined to 0.307(5). Two *tert*-Butyl groups are disordered. The disorder was refined with distance restraints and restraints for the anisotropic displacement parameters. The occupancies of the minor component refined to 0.30(2) and 0.194(6).

For **4a**: Two *tert*-Butyl groups are disordered. The disorder was refined with distance restraints and restraints for the anisotropic displacement parameters. The occupancies of the minor component refined to 0.355(8) and 0.134(12).

For **4b**: One *tert*-Butyl and the 5-hexenyl group are disordered. The disorder was refined with distance restraints and restraints for the anisotropic displacement parameters. The occupancies of the minor component refined to 0.169(16) and 0.073(8).

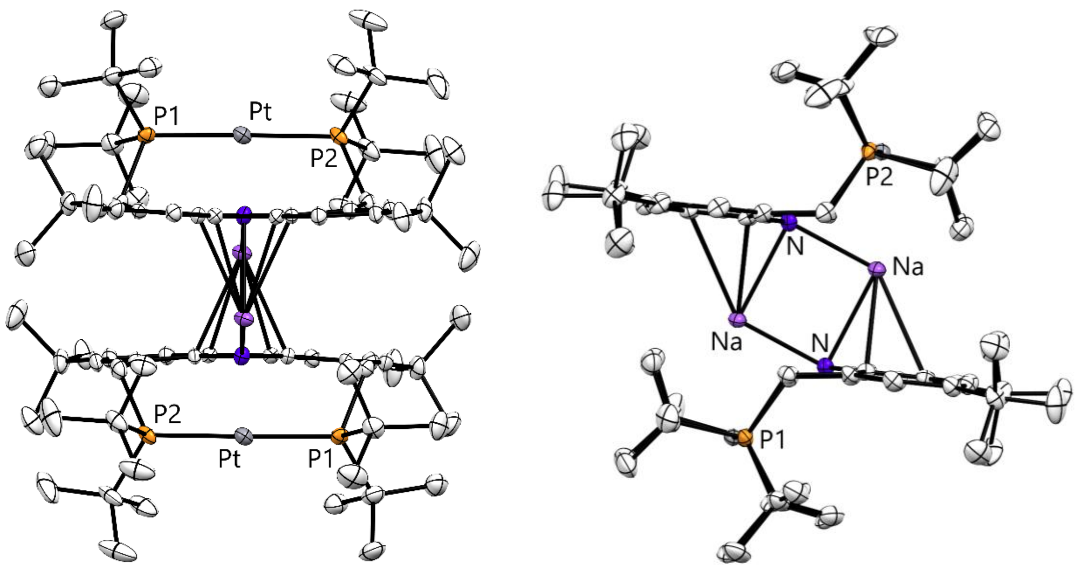

Figure 85: Molecular structure of **2-Na** (left: top view, right: view from the side) with anisotropic displacement parameters set to 30 % probability. Hydrogen atoms and a disorder of the whole molecule about a mirror plane are omitted for clarity. Selected bond parameters are discussed in the main part. The disorder was refined with distance restraints and restraints for anisotropic displacement parameters.

Table 4: Crystal data and structure refinement for **2-Na**.

CCDC Number	2530297
Empirical formula	$C_{76}H_{124}N_2Na_2P_4Pt_2$; C_6H_{14} (*n*-hexane)
Formula weight	1711.98
Temperature [K]	120(2)
Crystal system	orthorhombic
Space group	Ibam
a [Å]	16.345(2)
b [Å]	21.584(3)
c [Å]	23.874(3)
α [°]	90
β [°]	90
γ [°]	90
Volume [Å^3]	8422.5(19)
Z	4
ρ_{calc} [g/cm^3]	1.350
μ [mm^{-1}]	7.238
transmission factors (min, max)	0.584, 1.000
F(000)	3528.0
Radiation	Cu K_α (λ = 1.54184)
2Θ range for data collection [°]	6.784 to 145.042
Index ranges	-19 ≤ h ≤ 20, -24 ≤ k ≤ 26, -29 ≤ l ≤ 29
Reflections collected	74308
Independent reflections	4165 [R_{int} = 0.0632, R_{sigma} = 0.0181]
observed [I≥2σ(I)]	3908
Completeness to θ = 67.684°	1.00
Data/restraints/parameters	4165/1072/460
Goodness-of-fit on F^2	1.066
Final R indexes [I≥2σ (I)]	R_1 = 0.0298, wR_2 = 0.0745
Final R indexes [all data]	R_1 = 0.0320, wR_2 = 0.0768
Largest diff. peak/hole [eÅ^{-3}]	0.76/-1.46

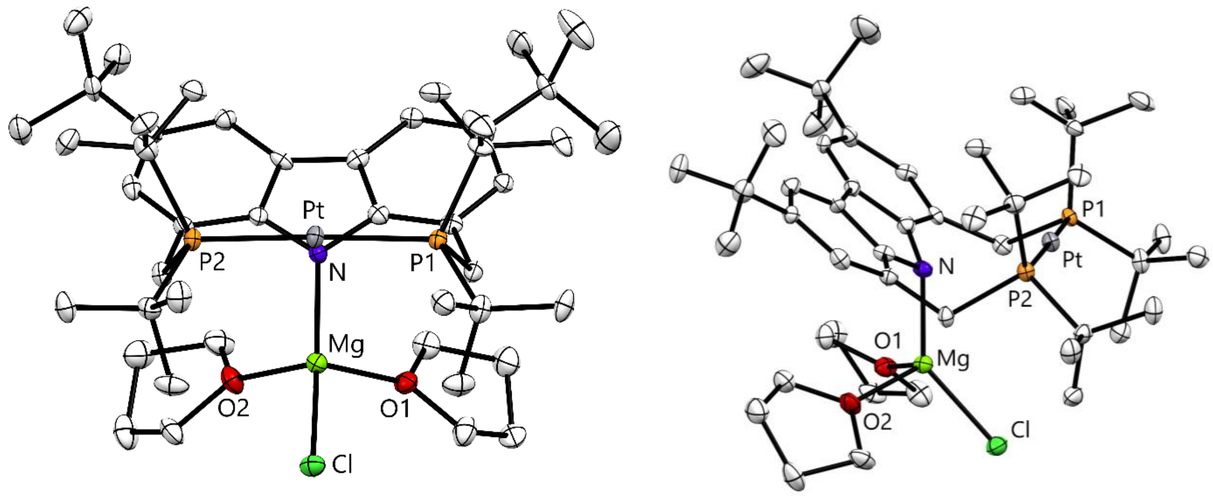

Figure 86: Molecular structure of **2-Mg** (left: top view, right: view from the side) with anisotropic displacement parameters set to 30 % probability. Hydrogen atoms, disorders and a $(thf)_4MgCl_2$ unit are omitted for clarity. Selected bond parameters are discussed in the main part.

Table 5: Crystal data and structure refinement for **2-Mg**.

CCDC Number	2530298
Empirical formula	$C_{74}H_{134}Cl_3Mg_2NO_9P_2Pt$
Formula weight	1593.81
Temperature [K]	120(2)
Crystal system	orthorhombic
Space group	$P2_12_12_1$
a [Å]	17.149(2)
b [Å]	21.147(2)
c [Å]	21.841(3)
α [°]	90
β [°]	90
γ [°]	90
Volume [Å^3]	7920.85(6)
Z	4
ρ_{calc} [g/cm^3]	1.337
μ [mm^{-1}]	5.189
transmission factors (min, max)	0.578, 1.000
F(000)	3360.0
Radiation	Cu K_α (λ = 1.54184)
2Θ range for data collection [°]	5.818 to 142.26
Index ranges	-20 ≤ h ≤ 20, -25 ≤ k ≤ 25, -26 ≤ l ≤ 26
Reflections collected	116640
Independent reflections	15254 [R_{int} = 0.0531, R_{sigma} = 0.0231]
observed [I≥2σ(I)]	15140
Completeness to θ = 67.684°	1.00
Data/restraints/parameters	15259/557/913
Goodness-of-fit on F^2	1.114
Absolute structure parameter[26]	0.482(10)
Final R indexes [I≥2σ (I)]	R_1 = 0.0398, wR_2 = 0.1040
Final R indexes [all data]	R_1 = 0.0400, wR_2 = 0.1042
Largest diff. peak/hole [eÅ^{-3}]	2.32/-1.06

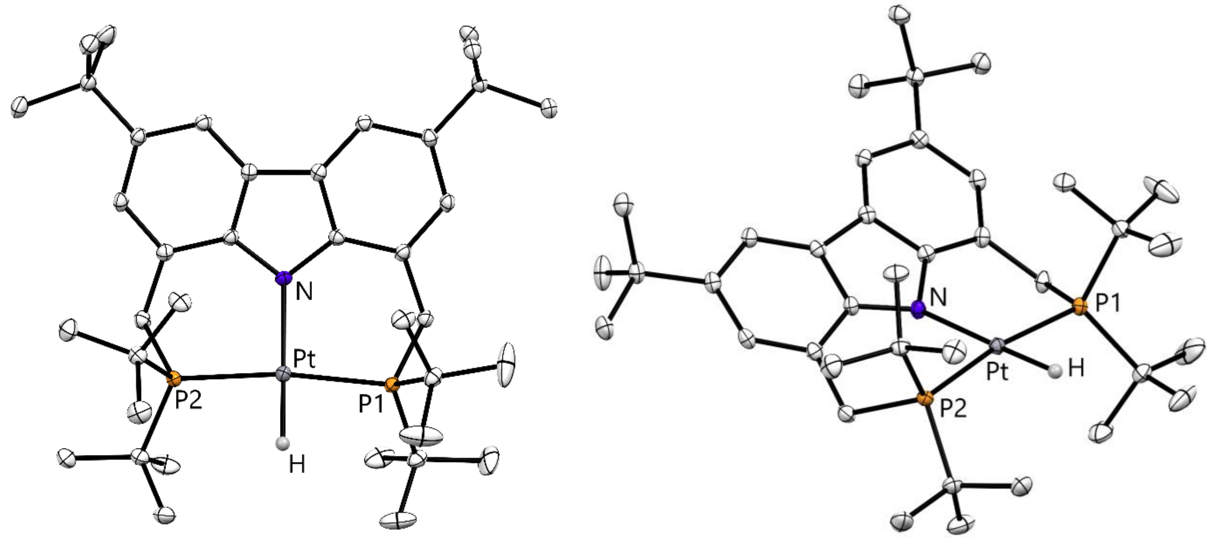

Figure 87: Molecular structure of **3-H** (left: top view, right: view from the side) with anisotropic displacement parameters set to 30 % probability. Hydrogen atoms (except the metal bonded) and disorders are omitted for clarity. Selected bond parameters in [Å] and [°]: Pt-P1 2.2736(6); Pt-P2 2.2731(6); Pt-N 2.1309(19); Pt-H 1.58(4); P2-Pt-P1 171.62(2); N-Pt-P1 93.72(6); N-Pt-P2 93.30(6); N-Pt-H 177.8(13); P1-Pt-H 86.8(13); P2-Pt-H 86.3(13).

Table 6: Crystal data and structure refinement for **3-H**.

CCDC Number	2530299
Empirical formula	$C_{38}H_{63}NP_2Pt$
Formula weight	790.92
Temperature [K]	120(2)
Crystal system	orthorhombic
Space group	Pbca
a [Å]	24.002(3)
b [Å]	11.877(2)
c [Å]	26.668(3)
α [°]	90
β [°]	90
γ [°]	90
Volume [Å^3]	7602.3(18)
Z	8
ρ_{calc} [g/cm^3]	1.382
μ [mm^{-1}]	7.870
transmission factors (min, max)	0.252, 0.825
F(000)	3248.0
Radiation	Cu K_α (λ = 1.54184)
2Θ range for data collection [°]	6.628 to 141.999
Index ranges	-29 ≤ h ≤ 29, -14 ≤ k ≤ 14, -32 ≤ l ≤ 32
Reflections collected	376833
Independent reflections	7330 [R_{int} = 0.0727, R_{sigma} = 0.0136]
observed [I≥2σ(I)]	7227
Completeness to θ = 67.684°	1.00
Data/restraints/parameters	7330/135/427
Goodness-of-fit on F^2	1.112
Final R indexes [I≥2σ (I)]	R_1 = 0.0233, wR_2 = 0.0632
Final R indexes [all data]	R_1 = 0.0236, wR_2 = 0.0634
Largest diff. peak/hole [eÅ^{-3}]	0.71/-0.81

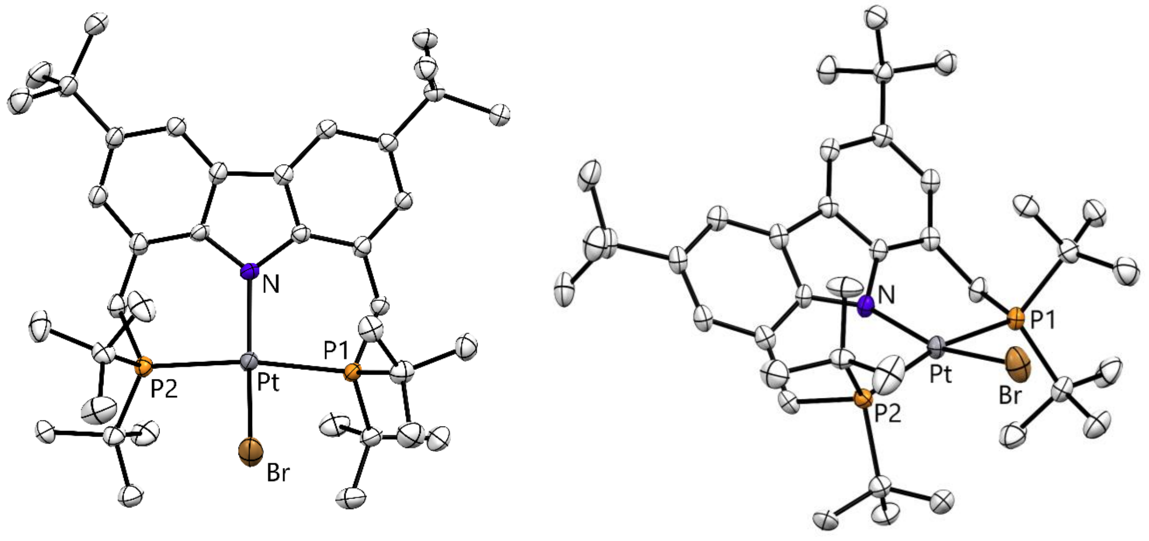

Figure 88: Molecular structure of **1-Br** (left: top view, right: view from the side) with anisotropic displacement parameters set to 30 % probability. Hydrogen atoms and disorders are omitted for clarity. Selected bond parameters in [Å] and [°]: Pt-Br 2.479(2); Pt-P1 2.3187(11); Pt-P2 2.3228(11); Pt-N 2.094(6); Br-Pt-P1 89.50(6); P2-Pt-Br 87.9(3); P2-Pt-P1 167.44(4); N-Pt-Br 161.31(11); N-Pt-P1 92.40(10); N-Pt-P2 92.92(10).

Table 7: Crystal data and structure refinement for **1-Br**.

CCDC Number	2530300
Empirical formula	$C_{38}H_{62}Br_{0.69}Cl_{0.31}NP_2Pt$
Formula weight	856.16
Temperature [K]	120(1)
Crystal system	orthorhombic
Space group	Pbca
a [Å]	23.585(2)
b [Å]	12.039(2)
c [Å]	27.068(3)
α [°]	90
β [°]	90
γ [°]	90
Volume [Å^3]	7685.7(17)
Z	8
ρ_{calc} [g/cm^3]	1.480
μ [mm^{-1}]	8.835
transmission factors (min, max)	0.616, 0.799
F(000)	3476.0
Radiation	Cu K_α (λ = 1.54184)
2Θ range for data collection [°]	6.53 to 142.524
Index ranges	-28 ≤ h ≤ 28, -14 ≤ k ≤ 14, -33 ≤ l ≤ 33
Reflections collected	106385
Independent reflections	7423 [R_{int} = 0.0597, R_{sigma} = 0.0196]
observed [I≥2σ(I)]	6655
Completeness to θ = 67.684°	1.00
Data/restraints/parameters	7423/110/449
Goodness-of-fit on F^2	1.041
Final R indexes [I≥2σ (I)]	R_1 = 0.0479, wR_2 = 0.0901
Final R indexes [all data]	R_1 = 0.0425, wR_2 = 0.0932
Largest diff. peak/hole [eÅ^{-3}]	1.54/-1.64

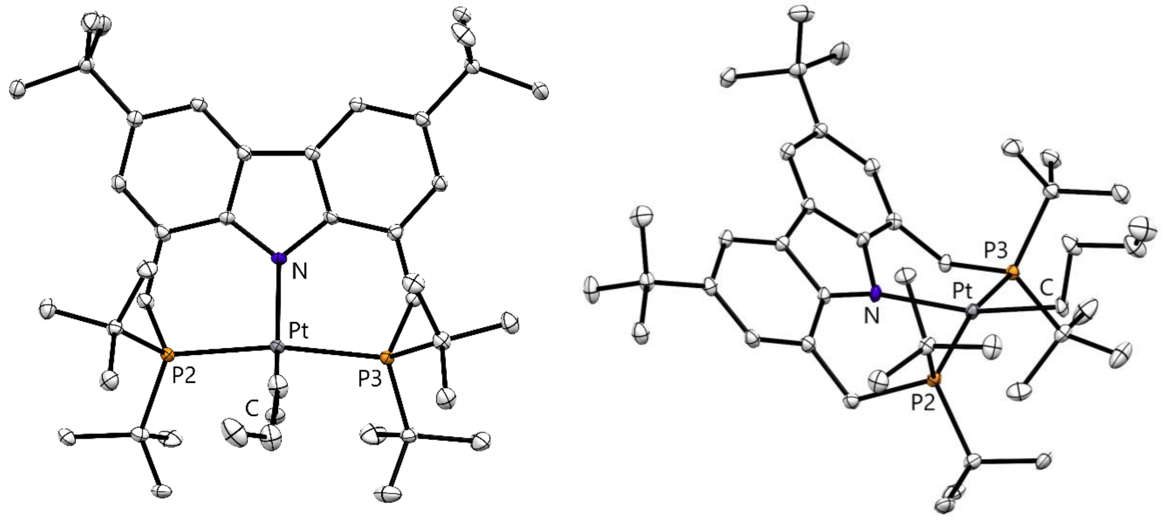

Figure 89: Molecular structure of **4a** (left: top view, right: view from the side) with anisotropic displacement parameters set to 30 % probability. Hydrogen atoms and disorders are omitted for clarity. Selected bond parameters in [Å] and [°]: Pt-C 2.103(3); Pt-P1 2.3277(7); Pt-P2 2.3280(7); Pt-N 2.171(2); C-Pt-P1 92.26(8); P2-Pt-C 91.13(8); P2-Pt-P1 162.89(2); N-Pt-C 165.26(10); N-Pt-P1 90.36(6); N-Pt-P2 90.62(6).

Table 8: Crystal data and structure refinement for **4a**.

CCDC Number	2530301
Empirical formula	$C_{42}H_{69}NP_2Pt$
Formula weight	845.01
Temperature [K]	120(2)
Crystal system	monoclinic
Space group	$P2_1/c$
a [Å]	12.514(2)
b [Å]	15.216(2)
c [Å]	21.949(3)
α [°]	90
β [°]	104.99(2)
γ [°]	90
Volume [$Å^3$]	4037.2(11)
Z	5
ρ_{calc} [g/cm^3]	1.390
μ [mm^{-1}]	7.446
transmission factors (min, max)	0.208, 1.000
F(000)	1744.0
Radiation	Cu K_α (λ = 1.54184)
2Θ range for data collection [°]	7.15 to 142.856
Index ranges	-15 ≤ h ≤ 15, -18 ≤ k ≤ 18, -26 ≤ l ≤ 26
Reflections collected	69627
Independent reflections	7813 [R_{int} = 0.0352, R_{sigma} = 0.0133]
observed [I≥2σ(I)]	7747
Completeness to θ = 67.684 °	1.00
Data/restraints/parameters	7813/246/487
Goodness-of-fit on F^2	1.040
Final R indexes [I≥2σ (I)]	R_1 = 0.0253, wR_2 = 0.0625
Final R indexes [all data]	R_1 = 0.0254, wR_2 = 0.0626
Largest diff. peak/hole [$eÅ^{-3}$]	1.62/-1.63

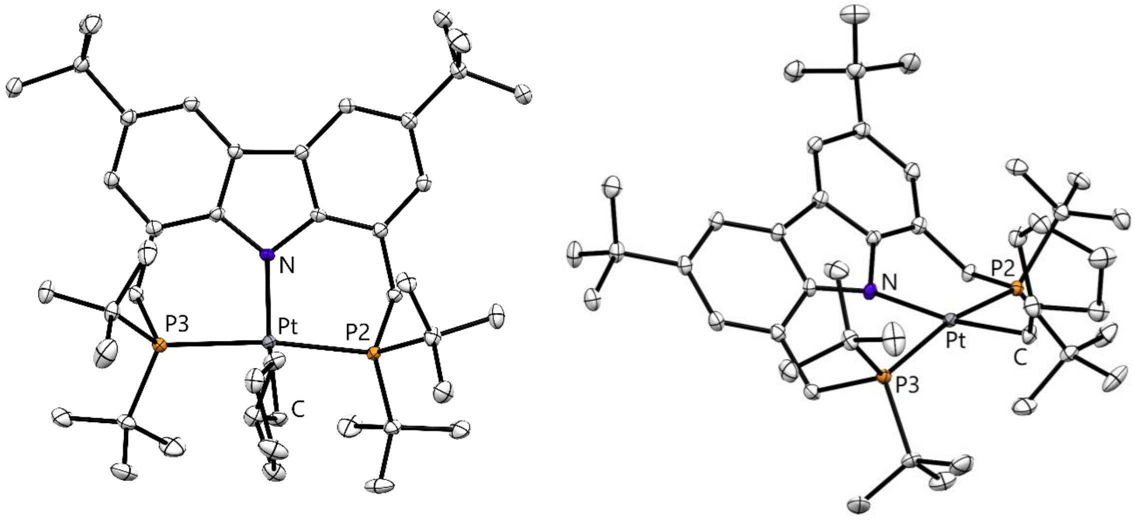

Figure 90: Molecular structure of **4b** (left: top view, right: view from the side) with anisotropic displacement parameters set to 30 % probability. Hydrogen atoms and disorders are omitted for clarity. Selected bond parameters in [Å] and [°]: Pt-C 2.089(2); Pt-P2 2.3605(6); Pt-P1 2.3281(6); Pt-N 2.170(2); C-Pt-P2 93.77(8); P1-Pt-C 87.89(8); P1-Pt-P2 165.38(2); N-Pt-C 171.92(9); N-Pt-P2 90.31(6); N-Pt-P1 89.92(6).

Table 9: Crystal data and structure refinement for **4b**.

CCDC Number	2530302
Empirical formula	$C_{44}H_{73}NP_2Pt$
Formula weight	873.06
Temperature [K]	110(2)
Crystal system	monoclinic
Space group	$P2_1/c$
a [Å]	12.634(2)
b [Å]	15.421(2)
c [Å]	22.003(3)
α [°]	90
β [°]	105.94(2)
γ [°]	90
Volume [Å^3]	4122.06(5)
Z	4
ρ_{calc} [g/cm^3]	1.407
μ [mm^{-1}]	7.311
transmission factors (min, max)	0.440, 0.633
F(000)	1808.0
Radiation	Cu K_α (λ = 1.54184)
2Θ range for data collection [°]	7.094 to 142.600
Index ranges	-15 ≤ h ≤ 15, -18 ≤ k ≤ 18, -26 ≤ l ≤ 27
Reflections collected	59833
Independent reflections	7978 [R_{int} = 0.0330, R_{sigma} = 0.0143]
observed [I≥2σ(I)]	7866
Completeness to θ = 67.684 °	1.00
Data/restraints/parameters	7978/285/490
Goodness-of-fit on F^2	1.078
Final R indexes [I≥2σ (I)]	R_1 = 0.0235, wR_2 = 0.0590
Final R indexes [all data]	R_1 = 0.0238, wR_2 = 0.0593
Largest diff. peak/hole [eÅ^{-3}]	1.09/-0.94

7.) Cyclic voltammetry

All cyclic voltammetry (CV) measurements were performed inside a glovebox under an argon atmosphere at 298 K using a PalmSens EmStat3 potentiostat controlled by PSTrace 5.9 software. A three-electrode setup was employed consisting of a glassy carbon working electrode (MF-2012), a platinum wire counter electrode (MW-1032), and a silver wire as reference electrode. The glassy carbon electrode was polished prior to each measurement. The silver wire was mechanically polished after every ten measurements, and the platinum wire was flame-annealed with a Bunsen burner after every five measurements until no coloration of the flame was observed. Measurements were conducted in tetrahydrofuran (5 mL). Tetrabutylammonium hexafluorophosphate (0.1 M) was used as the supporting electrolyte, and analyte concentrations were 5 mM. Due to reactivity of ferrocene with the analytes, external referencing was applied using a separate solution of ferrocene (5 mM) under identical conditions. The Fc/Fc^+ redox couple was determined to be at 0.32 V at a scan rate of 0.1 V/s, and all potentials were referenced accordingly. For each measurement, three consecutive cycles were recorded, and the second cycle was used for analysis. Scan rates were varied between 1.00 and 0.01 V/s.

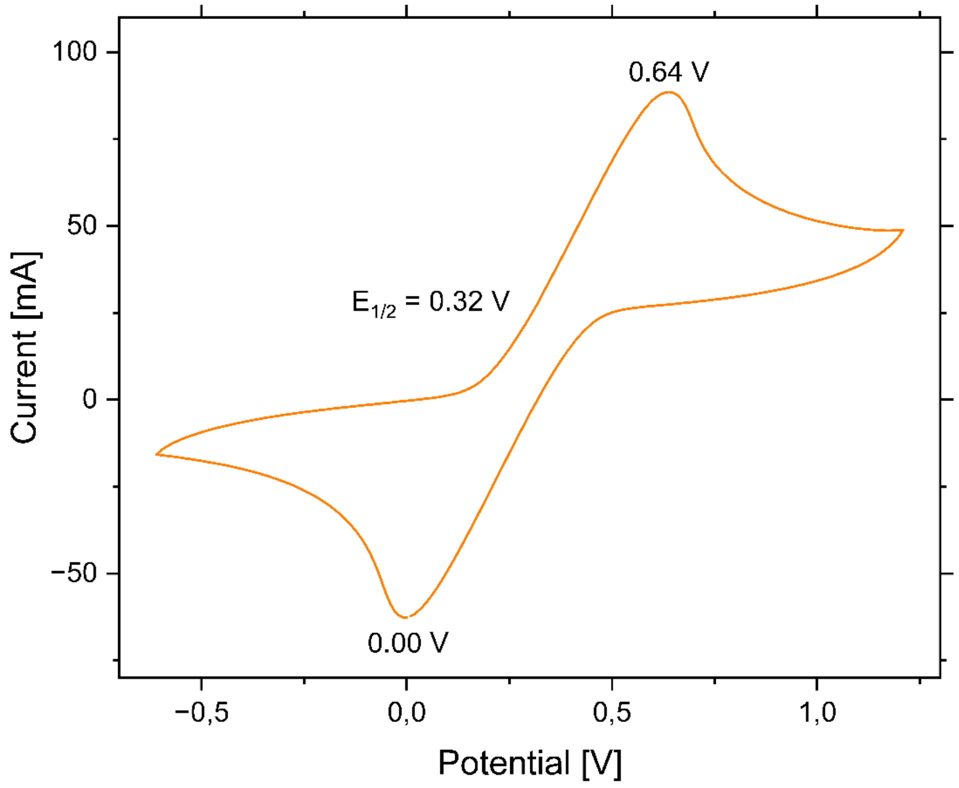

Figure 91: Cyclic voltammogram of Ferrocene in THF at a scan rate of 0.10 V/s.

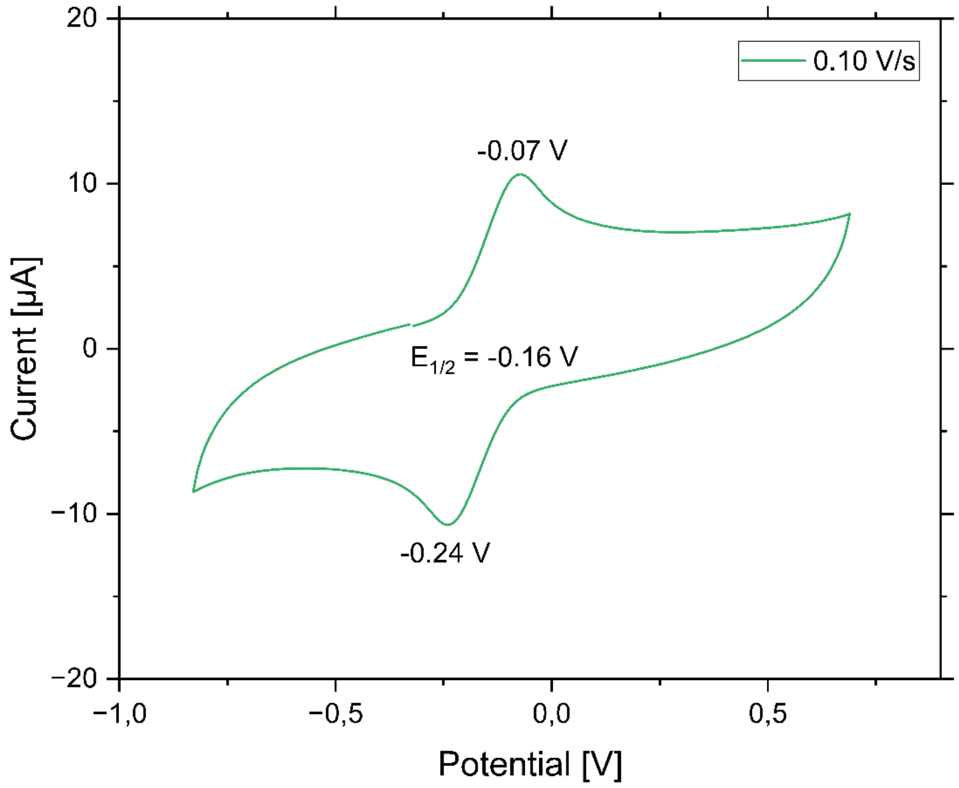

Figure 92: Cyclic voltammogram of $^{Cbz}[^{tBu}PNP]Pt^0Na$ (2-Na) in THF at a scan rate of 0.10 V/s.

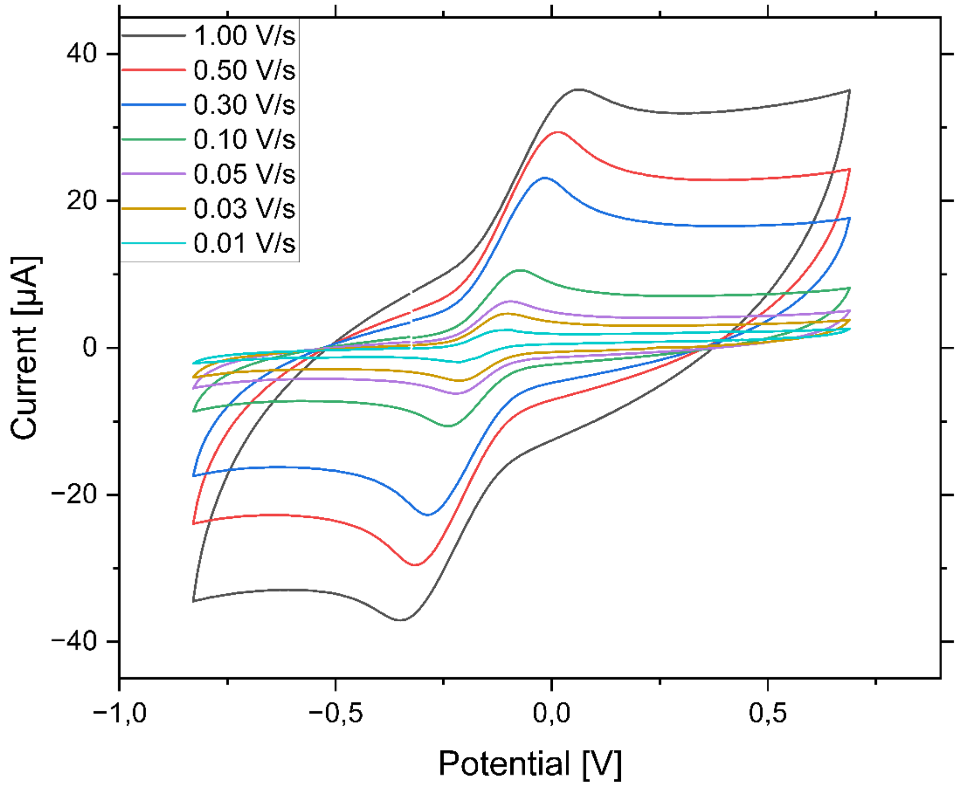

Figure 93: Cyclic voltammogram of $^{Cbz}[^{tBu}PNP]Pt^0Na$ (2-Na) in THF at various scan rates.

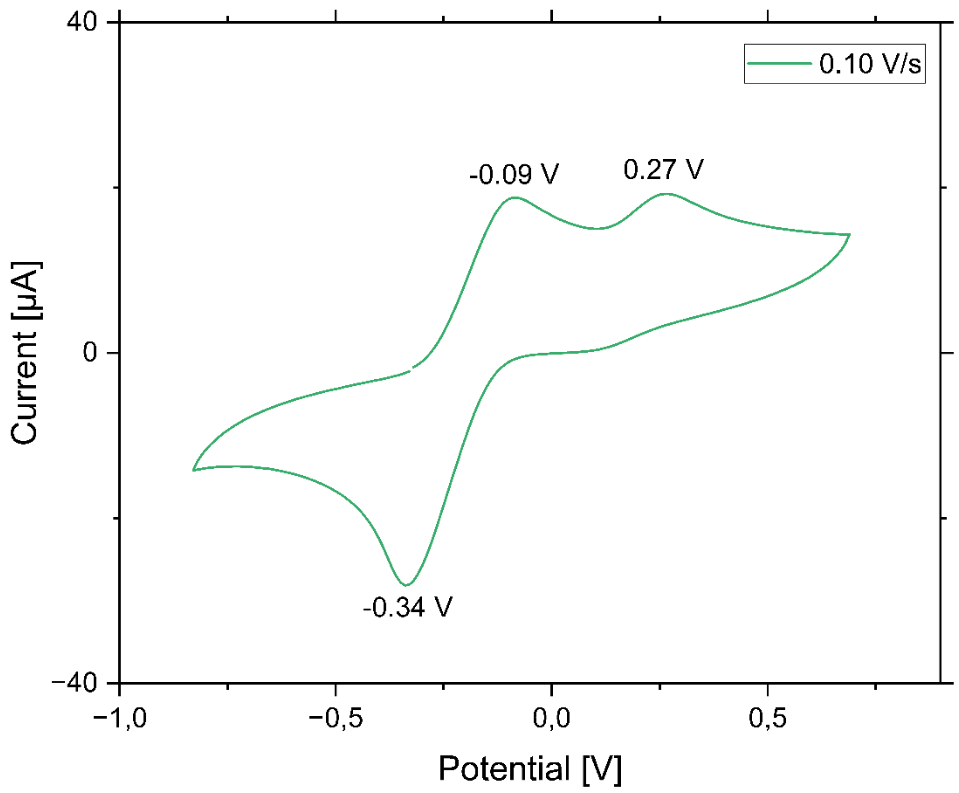

Figure 94: Cyclic voltammogram of $^{Cbz}[^{tBu}PNP]Pt^0MgCl$ (2-Mg) in THF at a scan rate of 0.10 V/s.

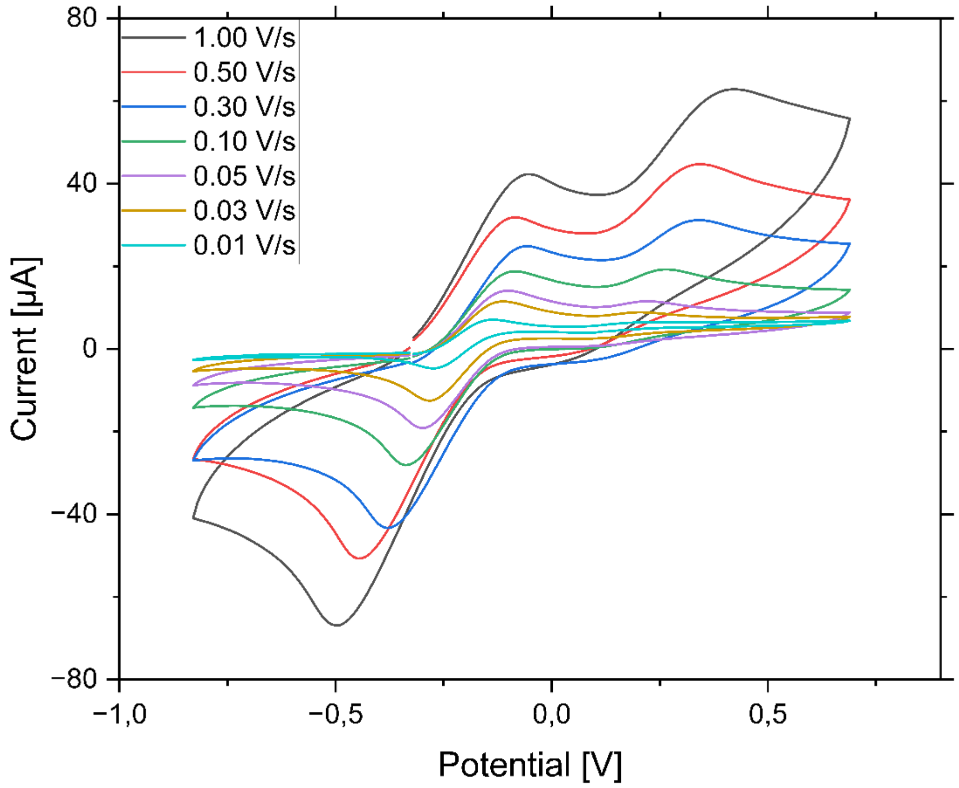

Figure 95: Cyclic voltammogram of $^{Cbz}[^{tBu}PNP]Pt^0MgCl$ (2-Mg) in THF at various scan rates.

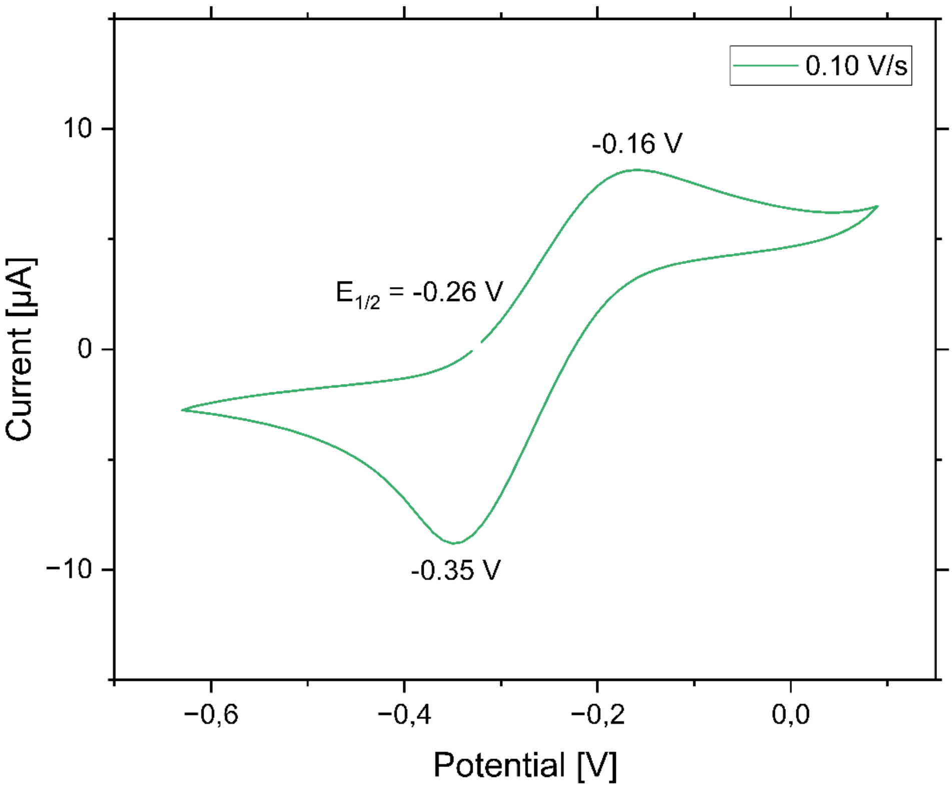

Figure 96: Cyclic voltammogram (first redox event only) of $^{Cbz}[^{tBu}PNP]Pt^0MgCl$ (2-Mg) in THF at a scan rate of 0.10 V/s.

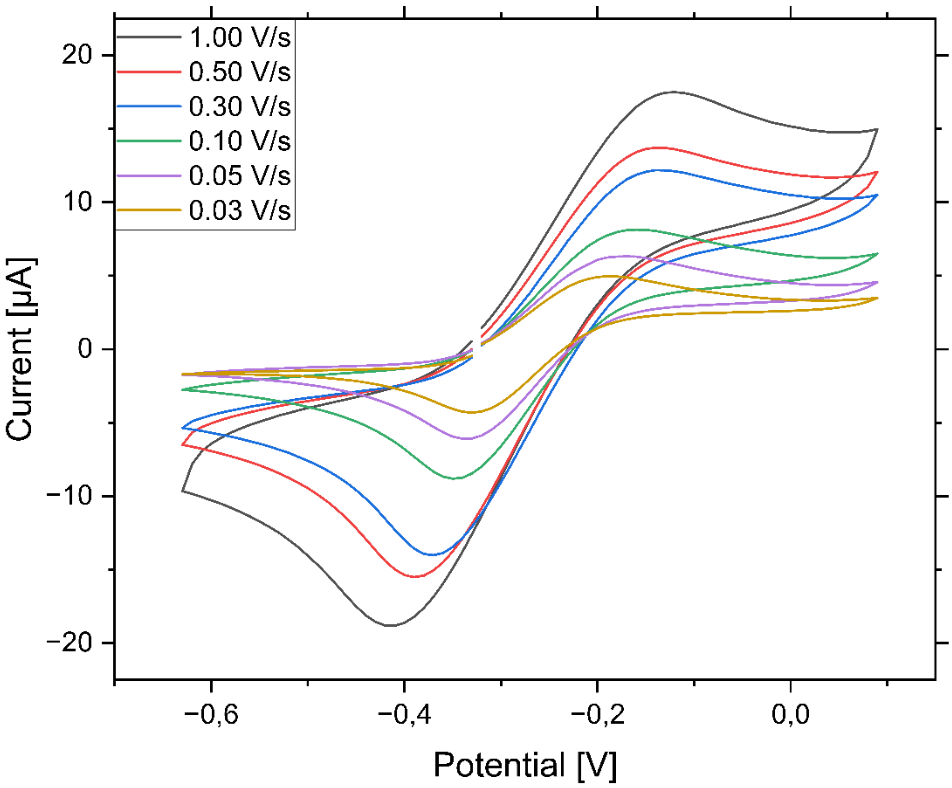

Figure 97: Cyclic voltammogram (first redox event only) of $^{Cbz}[^{tBu}PNP]Pt^0MgCl$ (2-Mg) in THF at various scan rates.

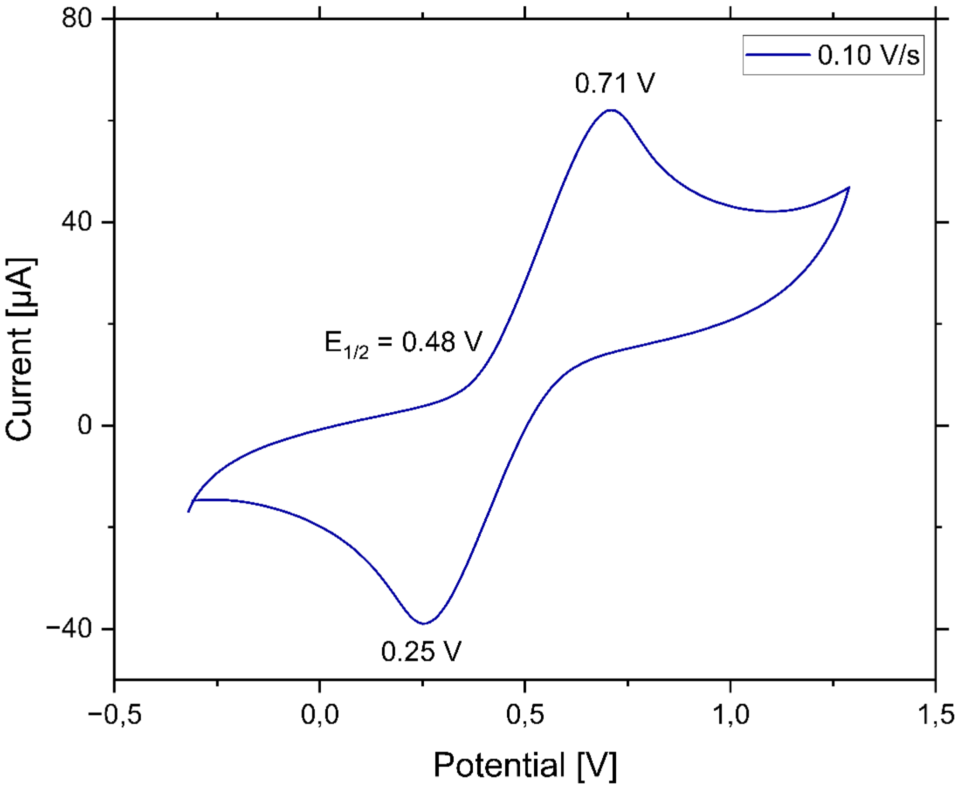

Figure 98: Cyclic voltammogram of Cbz[tBuPNP]PtIICl (1-Cl) in THF at a scan rate of 0.10 V/s.

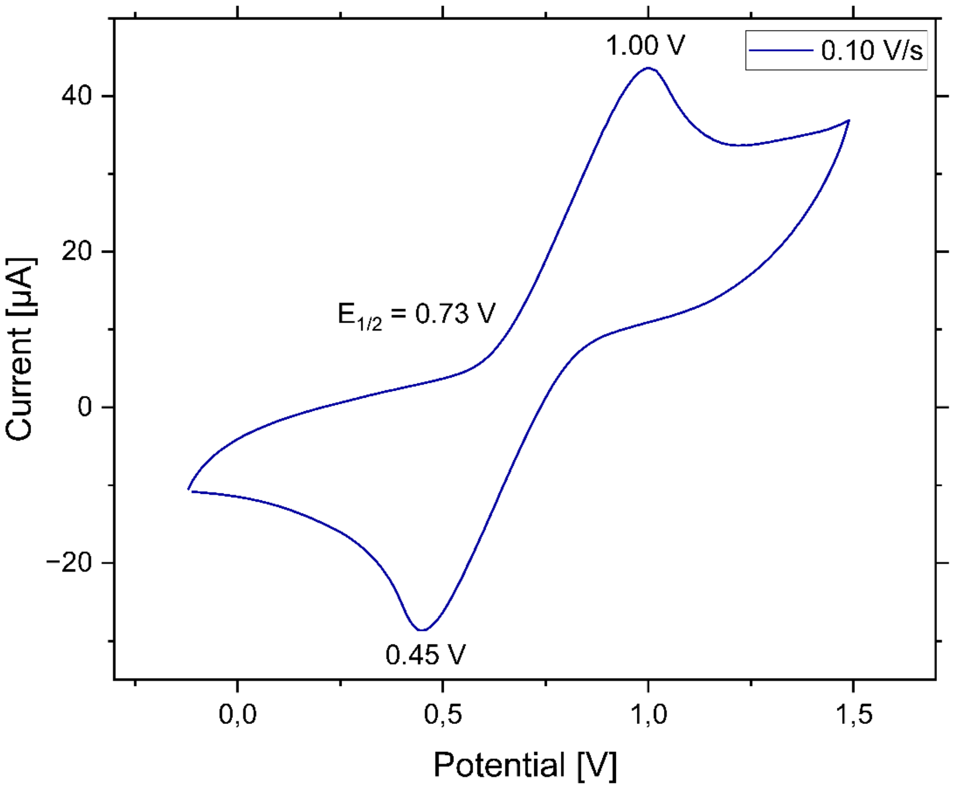

Figure 99: Cyclic voltammogram of Cbz[tBuPNP]PtIIBr (1-Br) in THF at a scan rate of 0.10 V/s.

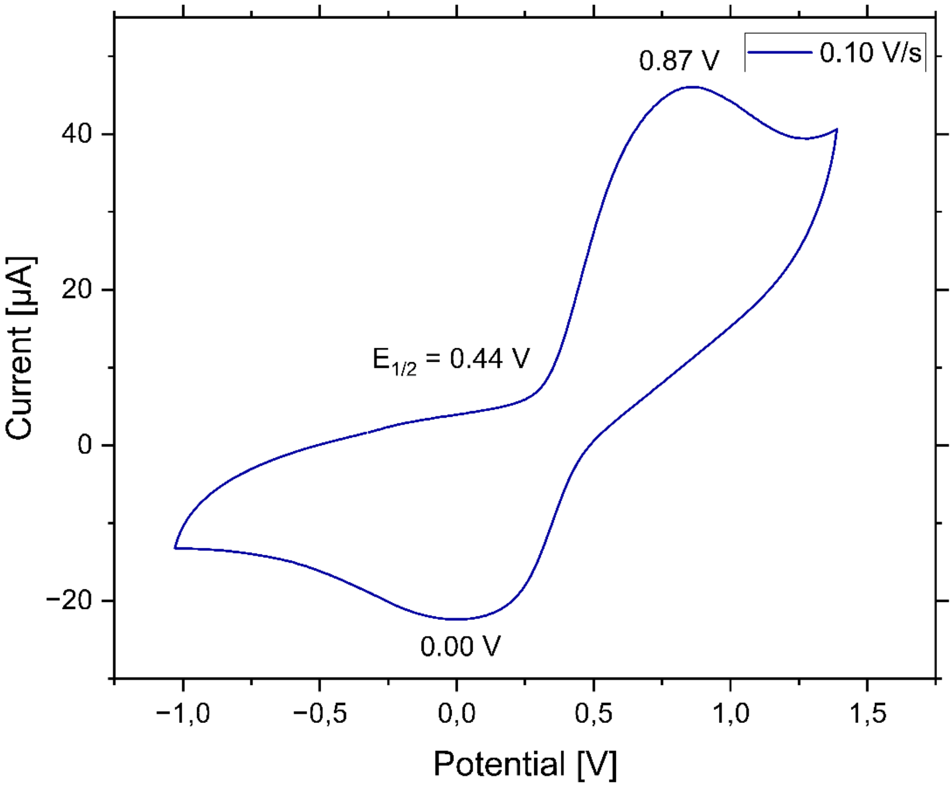

Figure 100: Cyclic voltammogram of ^{Cbz}[^{tBu}PNP]Pt^{II}H (3-H) in THF at a scan rate of 0.10 V/s.

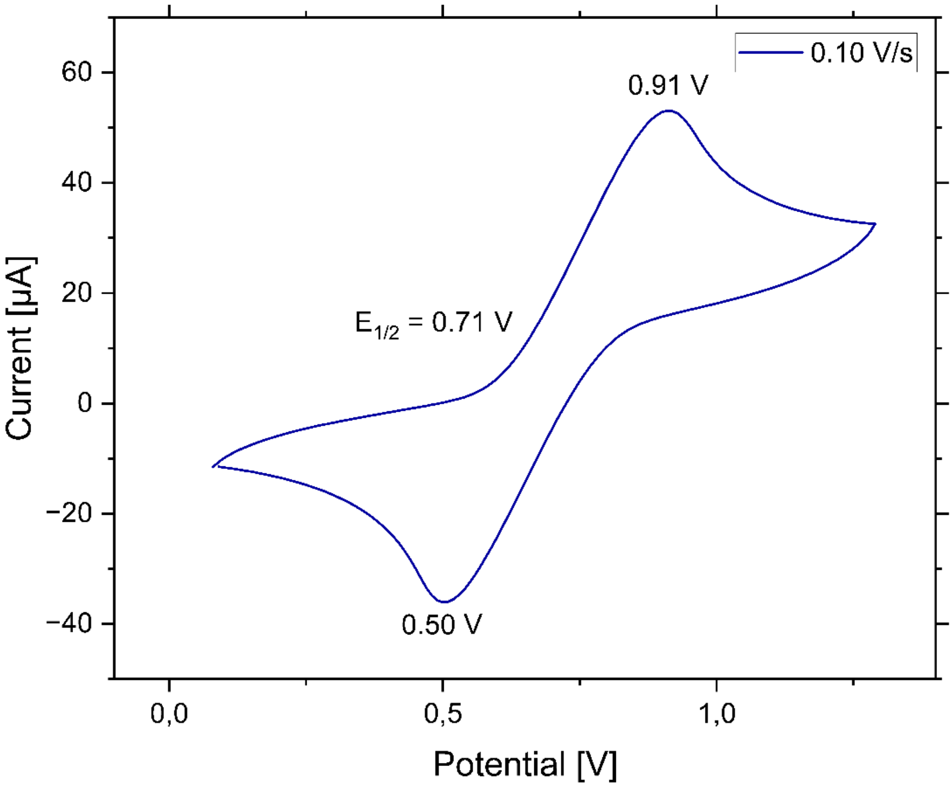

Figure 101: Cyclic voltammogram of ^{Cbz}[^{tBu}PNP]Pt^{II}CH_3 (4e) in THF at a scan rate of 0.10 V/s.

8.) Computational Details

All calculations were performed using ORCA 6.0.1.[27-29] Geometry optimizations were carried out using the r²SCAN-3c[30-32] composite with the def2-mTZVP[33-34] basis set and the corresponding def2/J auxiliary basis set. A spin-unrestricted Kohn-Sham (UKS) formalism was applied, and the RIJCOSX[35] approximation was used for an efficient evaluation of Coulomb and exchange integrals.

Single-point energy calculations were performed using the PWPB95[36] double-hybrid functional in conjunction with Grimme's D3 dispersion correction with Becke-Johnson damping (D3BJ)[37-40]. The def2-TZVP[33-34] basis set was employed together with the corresponding def2/J Coulomb-fitting and def2-TZVP/C correlation auxiliary basis sets. All calculations were carried out within the spin-unrestricted Kohn-Sham (UKS) framework using the RIJCOSX[35] approximation. Implicit solvation effects were taken into account using the CPCM[41] model with SMD parametrization for THF.

Quantum theory of atoms in molecules (QTAIM)[42-43] analyses were carried out on optimized structures using the B3LYP[38, 44-46] functional including Grimme's D3 dispersion correction with Becke-Johnson damping (D3BJ).[37-40] Calculations were performed within the spin-unrestricted Kohn-Sham (UKS) framework employing the RIJCOSX[35] approximation. Scalar relativistic effects were taken into account using the zero-order regular approximation (ZORA)[47-49] in conjunction with ZORA-def2-TZVP[33] basis sets for light atoms, while the SARC-ZORA-TZVP[50] basis set was applied to the platinum center together with the corresponding SARC/J auxiliary basis set. Spin-orbit coupling was considered using the SOMF(1X)[51-52] approach. For evaluating the QTAIM results Multiwfn 3.8.[53] was used.

Multireference calculations were carried out using the complete active space self-consistent field (CASSCF)[54-55] method. Scalar relativistic effects were treated using the zero-order regular approximation (ZORA)[47-49] in conjunction with the SARC-ZORA-TZVP[50] basis set for the platinum center and ZORA-def2-TZVP[33] basis sets for all other atoms, together with the corresponding SARC/J auxiliary basis set. The RIJCOSX[35] approximation was employed, and auxiliary basis sets were generated automatically using AutoAux.[56] The active space consisted of nine electrons distributed in five orbitals [CAS(9,5)], corresponding to the five 5d orbitals of the platinum center. Five doublet roots were included in the CASSCF calculations. Spin-orbit coupling effects were taken into account using the spin-orbit mean-field approach (SOMF(1X)).[51-52] Dynamic electron correlation was accounted for by second-order N-electron valence perturbation theory (SC-NEVPT2).[57-59] *Ab initio* ligand field theory (AILFT)[60] parameters were derived from the converged CASSCF wavefunctions. For visualization and analysis, spin and electron density cube files were generated from the CASSCF results.

Electron paramagnetic resonance (EPR) parameters were computed using the B3LYP[38, 44-46] functional including Grimme's D3 dispersion correction with Becke-Johnson damping (D3BJ). [37-40] All calculations were carried out within the spin-unrestricted Kohn-Sham (UKS) framework. Scalar relativistic effects were accounted for using the zero-order regular approximation (ZORA).[47-49] The SARC-ZORA-TZVP[50] basis set was employed for the platinum center, while ZORA-def2-TZVP[33] basis sets were used for all other elements, together with the corresponding SARC/J auxiliary basis set. The RIJCOSX[35] approximation was applied for an efficient evaluation of Coulomb and exchange integrals. Spin-orbit coupling effects were included using the spin-orbit mean-field (SOMF(1X))[51-52] approach. Implicit solvation effects were taken into account using the CPCM[41] model with tetrahydrofuran (THF) as solvent. The g tensor and hyperfine coupling constants were calculated including both one- and two-electron contributions to the g tensor. Isotropic and dipolar hyperfine coupling constants were evaluated for all C, N, P, and Pt nuclei, with additional orbital contributions considered for platinum.

All geometry and orbital pictures were created using Chemcraft 1.8.[61]

i. Geometry optimizations of complexes

Compound: $^{Cbz}[^{tBu}PNP]Pt^{II}Cl$ (1-Cl)

Pt	13.604880000	5.685568000	16.525088000
Cl	15.656154000	5.589965000	17.725300000
P	14.472990000	7.435982000	15.215607000
P	12.999466000	3.607150000	17.446160000
N	11.630215000	6.105813000	15.935579000
C	13.119051000	7.980506000	14.076707000
H	13.443026000	8.789317000	13.416226000
H	12.902310000	7.103740000	13.457923000
C	11.882240000	8.356446000	14.818969000
C	11.282897000	9.604117000	14.652714000
H	11.777034000	10.304567000	13.987917000
C	10.072702000	9.968000000	15.264683000
C	9.430157000	9.006779000	16.043875000
H	8.469421000	9.221282000	16.504457000
C	10.000601000	7.750680000	16.223438000
C	11.256378000	7.423266000	15.649762000
C	10.553437000	5.587930000	16.661735000
C	9.544288000	6.558304000	16.875021000
C	8.363322000	6.247316000	17.549714000
H	7.621650000	7.025455000	17.693102000
C	8.142124000	4.953378000	18.007176000
C	9.135704000	3.990800000	17.744080000
H	8.963357000	2.960051000	18.044069000
C	10.326442000	4.272060000	17.085024000
C	11.319355000	3.203405000	16.777345000
H	11.454100000	3.147085000	15.692147000
H	10.965433000	2.229521000	17.126037000
C	9.440389000	11.351615000	15.081721000
C	10.296164000	12.277487000	14.210949000
H	9.810082000	13.254685000	14.128822000
H	10.418661000	11.885202000	13.196297000
H	11.289315000	12.438163000	14.643548000
C	9.270691000	12.018211000	16.458229000
H	8.627511000	11.428521000	17.116855000
H	8.819836000	13.011381000	16.350073000
H	10.239992000	12.132739000	16.953891000
C	8.062571000	11.206182000	14.412099000
H	7.382477000	10.597265000	15.013759000
H	8.157465000	10.729939000	13.431104000
H	7.598660000	12.189536000	14.273677000
C	6.873100000	4.534256000	18.753467000
C	7.252997000	3.978730000	20.138144000
H	7.904952000	3.104036000	20.059205000
H	6.355223000	3.676764000	20.689353000
H	7.779728000	4.736417000	20.726948000
C	6.135124000	3.446327000	17.952620000
H	5.862289000	3.816634000	16.959570000
H	5.218210000	3.146157000	18.472347000
H	6.750028000	2.551956000	17.818843000
C	5.909096000	5.706576000	18.960396000
H	6.368309000	6.510732000	19.544054000
H	5.025879000	5.361996000	19.507419000
H	5.566269000	6.122282000	18.007675000
C	15.797030000	6.833588000	13.975247000
C	16.340568000	7.979096000	13.112750000
H	15.549297000	8.540211000	12.608098000
H	16.981397000	7.550864000	12.332366000
H	16.952423000	8.678886000	13.685760000
C	16.960684000	6.123377000	14.675252000
H	17.462327000	6.744249000	15.416722000
H	17.698143000	5.843700000	13.912723000
H	16.624275000	5.217061000	15.178673000
C	15.113165000	5.804765000	13.056681000
H	14.528268000	5.079873000	13.631035000
H	15.888078000	5.255592000	12.509898000
H	14.461180000	6.271881000	12.315172000
C	15.007531000	9.007716000	16.156682000
C	16.461485000	8.926456000	16.636290000
H	16.648612000	8.025147000	17.220590000
H	16.653363000	9.792215000	17.281813000
H	17.177049000	8.974071000	15.811777000
C	14.823420000	10.274135000	15.311966000
H	15.388684000	10.251685000	14.377238000
H	15.187821000	11.129797000	15.893041000
H	13.772549000	10.459841000	15.085094000
C	14.089576000	9.092372000	17.388333000
H	13.032320000	9.123942000	17.113300000
H	14.323808000	10.013484000	17.936689000
H	14.250012000	8.243530000	18.056968000
C	14.038988000	2.150965000	16.764762000
C	13.849433000	2.149195000	15.237787000
H	12.859566000	1.802282000	14.933395000
H	14.584460000	1.466562000	14.796229000
H	14.018966000	3.144690000	14.816994000
C	15.538507000	2.312515000	17.035620000
H	15.944153000	3.188512000	16.530977000
H	16.048874000	1.422605000	16.646578000
H	15.780772000	2.400939000	18.094016000
C	13.568749000	0.803262000	17.326589000
H	13.795495000	0.694350000	18.389348000
H	14.101987000	0.003768000	16.798136000
H	12.499669000	0.629976000	17.178640000
C	12.819419000	3.557828000	19.346416000
C	14.148068000	3.234045000	20.039797000
H	14.951576000	3.898228000	19.719122000
H	14.456027000	2.197185000	19.883812000
H	14.008388000	3.370720000	21.119054000
C	11.756618000	2.549566000	19.799476000
H	11.736757000	2.540805000	20.895855000
H	11.967976000	1.529728000	19.469670000
H	10.759674000	2.832262000	19.459355000
C	12.379474000	4.970952000	19.767171000
H	11.440457000	5.268526000	19.294596000
H	13.143076000	5.712565000	19.519834000
H	12.228016000	4.979636000	20.853763000

Compound: $^{Cbz}[^{tBu}PNP]Pt^{0}Na$ (2-Na) (as dimer)

Pt	3.131409000	7.165924000	13.380268000
P	4.751149000	5.574753000	13.212741000
P	1.494843000	8.739635000	13.216347000
N	4.074991000	8.139620000	10.916779000
C	5.529506000	5.524022000	11.473902000
H	6.249838000	4.699792000	11.431908000
H	4.718697000	5.244390000	10.791224000
C	6.179036000	6.792020000	10.998510000
C	7.550540000	6.868099000	10.784049000
H	8.128033000	5.956290000	10.910837000
C	8.236654000	8.057055000	10.443526000
C	7.499474000	9.229959000	10.318818000
H	7.985790000	10.173021000	10.092644000
C	6.112186000	9.203965000	10.502688000

C	5.435287000	7.976010000	10.798216000
C	3.872826000	9.494967000	10.800332000
C	5.080461000	10.207060000	10.504393000
C	5.066423000	11.594952000	10.322619000
H	5.994893000	12.108261000	10.095845000
C	3.873211000	12.298292000	10.450036000
C	2.704889000	11.578004000	10.790931000
H	1.776603000	12.128381000	10.919229000
C	2.668867000	10.204805000	11.003990000
C	1.420115000	9.519551000	11.480021000
H	1.162920000	8.703322000	10.795571000
H	0.576589000	10.217343000	11.439969000
C	9.760812000	8.010438000	10.293136000
C	10.388823000	7.657543000	11.654186000
H	10.111022000	8.399232000	12.409613000
H	11.482148000	7.633360000	11.579536000
H	10.055477000	6.678598000	12.011339000
C	10.165615000	6.946015000	9.258006000
H	9.811061000	5.948295000	9.530473000
H	11.256699000	6.897338000	9.170530000
H	9.756512000	7.187617000	8.272870000
C	10.338145000	9.352770000	9.833440000
H	9.916171000	9.662784000	8.871668000
H	11.422012000	9.263988000	9.709590000
H	10.154993000	10.147108000	10.563447000
C	3.782545000	13.820771000	10.302264000
C	3.408185000	14.435673000	11.663466000
H	3.353834000	15.528050000	11.590816000
H	4.154976000	14.177094000	12.420682000
H	2.437745000	14.074835000	12.017004000
C	2.709016000	14.196372000	9.265530000
H	1.721709000	13.811685000	9.535311000
H	2.964455000	13.796590000	8.280061000
H	2.628047000	15.285714000	9.180354000
C	5.108530000	14.438141000	9.847608000
H	5.434176000	14.028735000	8.885542000
H	5.905748000	14.276525000	10.579630000
H	4.988421000	15.519337000	9.726352000
C	4.051590000	3.796965000	13.373773000
C	3.215836000	3.713964000	14.659670000
H	3.822609000	3.761191000	15.564008000
H	2.675557000	2.758642000	14.672348000
H	2.491521000	4.533588000	14.693202000
C	5.096419000	2.680940000	13.355096000
H	5.730704000	2.710798000	12.463751000
H	4.586706000	1.709228000	13.356796000
H	5.738175000	2.706607000	14.239136000
C	3.076370000	3.597576000	12.202814000
H	2.306668000	4.378886000	12.213773000
H	2.553034000	2.642593000	12.331573000
H	3.556761000	3.562184000	11.221623000
C	6.244554000	5.855348000	14.368796000
C	5.854370000	5.477992000	15.801925000
H	4.891048000	5.915869000	16.081871000
H	6.616045000	5.866261000	16.489312000
H	5.807151000	4.395453000	15.945922000
C	7.523030000	5.115851000	13.969705000
H	7.398742000	4.032061000	13.932711000
H	8.301256000	5.331335000	14.712899000
H	7.900689000	5.456339000	13.004948000
C	6.512904000	7.370374000	14.334531000
H	6.775464000	7.727547000	13.336458000
H	7.351007000	7.595000000	15.007176000
H	5.634140000	7.927043000	14.672901000
C	-0.263055000	7.990935000	13.376780000
C	-0.325090000	7.155070000	14.663733000

H	0.519887000	6.461073000	14.705825000
H	-1.259989000	6.580131000	14.670591000
H	-0.307908000	7.765540000	15.566768000
C	-0.432071000	7.009198000	12.206460000
H	-0.476989000	7.487014000	11.224282000
H	-1.372936000	6.460414000	12.332705000
H	0.370644000	6.261946000	12.221704000
C	-1.408438000	9.003508000	13.355782000
H	-1.399568000	9.648361000	14.237891000
H	-2.365212000	8.466220000	13.360724000
H	-1.398303000	9.635464000	12.462307000
C	1.737397000	10.237009000	14.375362000
C	1.377409000	9.830996000	15.808484000
H	0.297119000	9.755272000	15.957059000
H	1.749048000	10.599148000	16.497825000
H	1.840938000	8.877750000	16.081732000
C	0.960251000	11.495128000	13.983210000
H	1.285457000	11.884196000	13.017702000
H	1.157253000	12.277433000	14.727271000
H	-0.119733000	11.340298000	13.951103000
C	3.244007000	10.549126000	14.335111000
H	3.828636000	9.685954000	14.666693000
H	3.447298000	11.390966000	15.009869000
H	3.587772000	10.825933000	13.336266000
Na	2.434027000	6.453289000	10.763400000
Pt	3.511652000	7.563559000	5.805610000
P	5.148666000	5.990358000	5.969494000
P	1.891443000	9.154240000	5.973574000
N	2.568396000	6.589634000	8.269673000
C	5.223319000	5.210810000	7.706025000
H	6.067423000	4.513730000	7.746763000
H	5.479333000	6.027534000	8.390266000
C	3.975003000	4.524856000	8.181938000
C	3.939398000	3.151668000	8.395060000
H	4.867837000	2.601532000	8.266861000
C	2.771241000	2.431015000	8.735744000
C	1.577727000	3.133892000	8.862914000
H	0.649460000	2.620118000	9.089469000
C	1.563307000	4.521826000	8.681341000
C	2.770824000	5.234240000	8.385774000
C	1.208015000	6.752830000	8.388040000
C	0.531342000	5.524634000	8.683206000
C	-0.855928000	5.498228000	8.867072000
H	-1.341934000	4.554940000	9.092957000
C	-1.593466000	6.670928000	8.742553000
C	-0.907709000	7.860050000	8.402019000
H	-1.485395000	8.771751000	8.275257000
C	0.463816000	7.936551000	8.187696000
C	1.112810000	9.204852000	7.712128000
H	1.923009000	9.485291000	8.395282000
H	0.391933000	10.028628000	7.753617000
C	2.862388000	0.908582000	8.883493000
C	3.237174000	0.293765000	7.522384000
H	2.490414000	0.551988000	6.765022000
H	3.291995000	-0.798598000	7.595006000
H	4.207512000	0.655123000	7.169064000
C	3.935819000	0.533473000	9.920529000
H	4.923170000	0.918037000	9.650858000
H	4.016814000	-0.555842000	10.005965000
H	3.680043000	0.933608000	10.905765000
C	1.536577000	0.290767000	9.338123000
H	1.211089000	0.700146000	10.300265000
H	1.656911000	-0.790407000	9.459200000
H	0.739175000	0.452249000	8.606286000
C	-3.117639000	6.716993000	8.893018000
C	-3.745830000	7.069306000	7.531897000

H	-4.839162000	7.093047000	7.606567000
H	-3.467764000	6.327564000	6.776616000
H	-3.412943000	8.048315000	7.174497000
C	-3.522735000	7.781602000	9.927848000
H	-3.168483000	8.779350000	9.655036000
H	-3.113473000	7.540451000	10.913038000
H	-4.613834000	7.830025000	10.015395000
C	-3.694397000	5.374584000	9.353222000
H	-3.272041000	5.065364000	10.315066000
H	-3.511066000	4.580019000	8.623516000
H	-4.778282000	5.462986000	9.477163000
C	6.906446000	6.739116000	5.809296000
C	6.967779000	7.576357000	4.523173000
H	6.947896000	6.967234000	3.619306000
H	7.903604000	8.149807000	4.515518000
H	6.123807000	8.271679000	4.483366000
C	8.051853000	5.726548000	5.828643000
H	8.041857000	5.093132000	6.721081000
H	9.008599000	6.263891000	5.824475000
H	8.042917000	5.083023000	4.945558000
C	7.076170000	7.719573000	6.980542000
H	6.272689000	8.466076000	6.967840000
H	8.016141000	8.269680000	6.853396000
H	7.122880000	7.240492000	7.961971000
C	4.906541000	4.492943000	4.810454000
C	5.266465000	4.899111000	3.377377000
H	4.802539000	5.852206000	3.104231000
H	4.895187000	4.130876000	2.687936000
H	6.346727000	4.975315000	3.228850000
C	5.683928000	3.234959000	5.202607000
H	6.763908000	3.389768000	5.234010000
H	5.486410000	2.452387000	4.458980000
H	5.359250000	2.846300000	6.168458000
C	3.399997000	4.180434000	4.850670000
H	3.056428000	3.903204000	5.849459000
H	3.196880000	3.338767000	4.175664000
H	2.815049000	5.043535000	4.519454000
C	2.590648000	10.932255000	5.812713000
C	3.426253000	11.015635000	4.526764000
H	4.151239000	10.196643000	4.493384000
H	3.965708000	11.971431000	4.513860000
H	2.819401000	10.967727000	3.622554000
C	3.566009000	11.131474000	6.983650000
H	3.085577000	11.166704000	7.964833000
H	4.089053000	12.086611000	6.854951000
H	4.335801000	10.350321000	6.972348000
C	1.545604000	12.048033000	5.831846000
H	0.903787000	12.022766000	4.947812000
H	2.055239000	13.019774000	5.830668000
H	0.911337000	12.017638000	6.723179000
C	0.398148000	8.873292000	4.817392000
C	0.788321000	9.250864000	3.384287000
H	0.834805000	10.333440000	3.240265000
H	0.026988000	8.862099000	2.696806000
H	1.751941000	8.813600000	3.104408000
C	-0.880507000	9.612505000	5.216379000
H	-1.258151000	9.271692000	6.181043000
H	-1.658631000	9.397033000	4.473067000
H	-0.756541000	10.696322000	5.253615000
C	0.130169000	7.358217000	4.851428000
H	1.009059000	6.801937000	4.512746000
H	-0.707976000	7.133481000	4.178879000
H	-0.132060000	7.000671000	5.849457000
Na	4.205791000	8.277915000	8.422601000

Compound: $^{Cbz}[^{tBu}PNP]Pt^{0}MgCl$ (2-Mg) (with two units THF)

Pt	8.609558000	11.864349000	9.093882000
Cl	9.066857000	8.663702000	6.797169000
P	10.890545000	12.136774000	9.112327000
P	6.344517000	12.039887000	9.052490000
Mg	8.691955000	10.953748000	6.463274000
O	10.030173000	10.991245000	4.764314000
O	7.088005000	10.645817000	5.065382000
N	8.695644000	13.034565000	6.513060000
C	11.600195000	12.234321000	7.361282000
H	12.695112000	12.194499000	7.413313000
H	11.288449000	11.292954000	6.897937000
C	11.184710000	13.432407000	6.552078000
C	12.155160000	14.353774000	6.164571000
H	13.192499000	14.096788000	6.366801000
C	11.874626000	15.606781000	5.581649000
C	10.539155000	15.970446000	5.436814000
H	10.265492000	16.951175000	5.062609000
C	9.532429000	15.074075000	5.798847000
C	9.842147000	13.765936000	6.267818000
C	7.647079000	13.922813000	6.365062000
C	8.102641000	15.181150000	5.873051000
C	7.214439000	16.224005000	5.607207000
H	7.601271000	17.161521000	5.222259000
C	5.856230000	16.054447000	5.852913000
C	5.435284000	14.826205000	6.402076000
H	4.379740000	14.696590000	6.627759000
C	6.282202000	13.759389000	6.697490000
C	5.712593000	12.523041000	7.339756000
H	5.938400000	11.646430000	6.730077000
H	4.619632000	12.603019000	7.372184000
C	13.037666000	16.526552000	5.197896000
C	13.854988000	16.878304000	6.454005000
H	13.222945000	17.376733000	7.195838000
H	14.682369000	17.550580000	6.199293000
H	14.281685000	15.988319000	6.925096000
C	13.944130000	15.810758000	4.181209000
H	14.357574000	14.882972000	4.587288000
H	14.784807000	16.453148000	3.895039000
H	13.383292000	15.558777000	3.275095000
C	12.556069000	17.834830000	4.563179000
H	11.966231000	17.651542000	3.659393000
H	13.419173000	18.445556000	4.279858000
H	11.949418000	18.423743000	5.258100000
C	4.814374000	17.144572000	5.585193000
C	4.105059000	17.518871000	6.899457000
H	3.376540000	18.319565000	6.727742000
H	4.829233000	17.865884000	7.643087000
H	3.567373000	16.668523000	7.328449000
C	3.774206000	16.625791000	4.576115000
H	3.259747000	15.735554000	4.948746000
H	4.254595000	16.363169000	3.628015000
H	3.016269000	17.392039000	4.376676000
C	5.443373000	18.416110000	5.007595000
H	5.950846000	18.220741000	4.057577000
H	6.165096000	18.861243000	5.699722000
H	4.662649000	19.160015000	4.819466000
C	11.893701000	10.699662000	9.887570000
C	11.093736000	10.174734000	11.088421000
H	10.973009000	10.922508000	11.875336000
H	11.619584000	9.313151000	11.519756000
H	10.094588000	9.861027000	10.774480000
C	13.318798000	11.046293000	10.321303000
H	13.905551000	11.497481000	9.514883000
H	13.828213000	10.119553000	10.613937000

H	13.343909000	11.711942000	11.186687000
C	11.964155000	9.570672000	8.851394000
H	10.981361000	9.310629000	8.453068000
H	12.365766000	8.675210000	9.341141000
H	12.633858000	9.810021000	8.019215000
C	11.342039000	13.807747000	9.932023000
C	10.976290000	13.700527000	11.418218000
H	9.940832000	13.367885000	11.548789000
H	11.080324000	14.687501000	11.885429000
H	11.629182000	13.012798000	11.961567000
C	12.802707000	14.244687000	9.778925000
H	13.499517000	13.641946000	10.359345000
H	12.892064000	15.280067000	10.131968000
H	13.118458000	14.240344000	8.732846000
C	10.462026000	14.899411000	9.290832000
H	10.870104000	15.237825000	8.338676000
H	10.429140000	15.762602000	9.967678000
H	9.439719000	14.556844000	9.116746000
C	5.371340000	10.422903000	9.387566000
C	6.020943000	9.725579000	10.591621000
H	7.089278000	9.573375000	10.414068000
H	5.544167000	8.748198000	10.739966000
H	5.910604000	10.292951000	11.516950000
C	5.571545000	9.507716000	8.171744000
H	5.020921000	9.856479000	7.293056000
H	5.192461000	8.508452000	8.417870000
H	6.630170000	9.394044000	7.919258000
C	3.869308000	10.594311000	9.622161000
H	3.645019000	11.126813000	10.548813000
H	3.408828000	9.601413000	9.701566000
H	3.375201000	11.113540000	8.794516000
C	5.727314000	13.443172000	10.193165000
C	5.767368000	12.979338000	11.653344000
H	4.978613000	12.261288000	11.890417000

H	5.623574000	13.850812000	12.304074000
H	6.735328000	12.531562000	11.900124000
C	4.332068000	13.967601000	9.840590000
H	4.328365000	14.454169000	8.863445000
H	4.045899000	14.726596000	10.579457000
H	3.561902000	13.195584000	9.850377000
C	6.727000000	14.600399000	10.044157000
H	7.711496000	14.310425000	10.417613000
H	6.367483000	15.453648000	10.634312000
H	6.838134000	14.928725000	9.007688000
C	11.155306000	10.127754000	4.473012000
H	10.845775000	9.391581000	3.717697000
H	11.414419000	9.591712000	5.386052000
C	12.219390000	11.081930000	3.938561000
H	12.907560000	10.577071000	3.256350000
H	12.802225000	11.495556000	4.765472000
C	11.403119000	12.204026000	3.253877000
H	11.529643000	12.205166000	2.168252000
H	11.694084000	13.183533000	3.635820000
C	9.950346000	11.887667000	3.631458000
H	9.367541000	12.757861000	3.933867000
H	9.434632000	11.361299000	2.815237000
C	6.570574000	9.347457000	4.656823000
H	6.618365000	8.686970000	5.519897000
H	7.233518000	8.947517000	3.879479000
C	5.156020000	9.626252000	4.123868000
H	4.394360000	9.288564000	4.829988000
H	4.990691000	9.097539000	3.182110000
C	5.104818000	11.160728000	3.939471000
H	4.692900000	11.461572000	2.973404000
H	4.502358000	11.629708000	4.722145000
C	6.558509000	11.579401000	4.089609000
H	7.108176000	11.451160000	3.146919000
H	6.709842000	12.590449000	4.467386000

Compound: ^{Cbz}[^{tBu}PNP]Pt^{II}H (3-H)

Pt	10.170452000	5.465854000	10.276943000
H	8.667987000	5.196832000	9.852915000
P	9.393026000	7.228603000	11.541295000
P	10.745541000	3.535136000	9.155206000
N	12.252196000	5.962697000	10.680529000
C	10.785633000	7.806729000	12.623701000
H	10.475608000	8.624639000	13.282134000
H	11.018909000	6.940040000	13.251866000
C	12.010042000	8.181443000	11.849475000
C	12.597425000	9.436523000	12.006166000
H	12.107887000	10.129363000	12.683001000
C	13.786555000	9.823280000	11.365001000
C	14.420216000	8.881233000	10.554514000
H	15.359027000	9.120965000	10.061402000
C	13.865316000	7.617843000	10.378825000
C	12.633898000	7.260332000	10.995609000
C	13.284229000	5.470545000	9.892781000
C	14.290894000	6.449827000	9.661882000
C	15.412187000	6.164426000	8.889855000
H	16.157477000	6.940028000	8.731607000
C	15.584002000	4.894779000	8.338416000
C	14.598728000	3.929392000	8.606049000
H	14.723602000	2.921197000	8.224528000
C	13.459503000	4.181042000	9.369983000
C	12.476897000	3.089752000	9.658431000
H	12.418540000	2.938106000	10.741732000
H	12.797210000	2.147017000	9.203220000
C	14.405004000	11.213933000	11.545523000
C	13.562131000	12.119463000	12.449771000
H	12.555741000	12.272401000	12.045779000

H	14.038505000	13.101724000	12.530357000
H	13.471726000	11.713658000	13.462449000
C	14.529049000	11.899699000	10.173413000
H	15.160572000	11.325708000	9.490156000
H	14.970789000	12.897251000	10.280960000
H	13.544839000	12.007644000	9.706280000
C	15.802454000	11.079886000	12.175925000
H	15.739357000	10.592956000	13.154248000
H	16.259210000	12.067249000	12.311261000
H	16.472032000	10.483741000	11.550098000
C	16.825483000	4.591627000	7.492305000
C	16.865915000	5.541977000	6.282279000
H	15.973766000	5.412819000	5.661130000
H	17.748482000	5.338984000	5.664707000
H	16.907646000	6.589606000	6.592122000
C	18.091774000	4.797073000	8.342528000
H	18.167752000	5.823680000	8.710671000
H	18.990195000	4.584748000	7.751349000
H	18.086290000	4.131506000	9.211560000
C	16.839482000	3.153802000	6.962257000
H	16.853992000	2.420381000	7.774780000
H	17.738583000	2.994865000	6.358495000
H	15.972933000	2.948444000	6.324884000
C	8.060592000	6.708097000	12.802990000
C	6.917920000	5.948630000	12.116883000
H	6.403935000	6.539636000	11.358541000
H	6.179498000	5.667696000	12.877618000
H	7.286036000	5.037602000	11.642694000
C	7.489351000	7.887652000	13.596564000
H	8.269814000	8.490830000	14.069336000

H	6.848764000	7.498864000	14.397463000
H	6.872128000	8.543285000	12.977761000
C	8.740657000	5.730573000	13.777522000
H	9.303143000	4.959096000	13.240841000
H	7.966390000	5.233312000	14.373345000
H	9.413561000	6.236135000	14.473852000
C	8.871002000	8.739105000	10.510835000
C	7.457220000	8.543371000	9.954476000
H	7.357675000	7.584808000	9.436385000
H	7.255124000	9.337770000	9.226122000
H	6.688204000	8.608313000	10.727940000
C	8.955686000	10.054253000	11.291432000
H	9.982739000	10.271661000	11.591912000
H	8.627632000	10.871913000	10.638071000
H	8.319663000	10.069776000	12.178903000
C	9.844398000	8.808442000	9.320880000
H	10.880020000	8.944282000	9.638662000
H	9.566283000	9.662837000	8.690954000
H	9.790555000	7.898702000	8.716585000
C	9.736890000	2.015834000	9.714864000
C	8.229809000	2.284437000	9.621628000
H	7.944998000	3.129333000	10.250201000
H	7.695217000	1.394996000	9.976733000
H	7.891747000	2.488996000	8.605505000
C	10.078170000	0.743746000	8.932006000
H	9.730235000	0.790193000	7.897377000
H	9.576019000	-0.108907000	9.405001000
H	11.150230000	0.527053000	8.928026000
C	10.071361000	1.798077000	11.200716000
H	11.075669000	1.394880000	11.349383000
H	9.362508000	1.073439000	11.618040000
H	9.978588000	2.729553000	11.769337000
C	10.785410000	3.727137000	7.262548000
C	9.367561000	3.615907000	6.691821000
H	8.980450000	2.594500000	6.726570000
H	9.391544000	3.919037000	5.638409000
H	8.669660000	4.278640000	7.212512000
C	11.711407000	2.719246000	6.574141000
H	12.751422000	2.863460000	6.874254000
H	11.660284000	2.880639000	5.490336000
H	11.431452000	1.681180000	6.763484000
C	11.301114000	5.148208000	6.975200000
H	10.655631000	5.901431000	7.435522000
H	11.301323000	5.305611000	5.889301000
H	12.318003000	5.303918000	7.339523000

Compound: $^{Cbz}[^{tBu}PNP]Pt^{II}D$ (3-D)

Pt	10.170516000	5.465888000	10.277241000
D	8.667871000	5.197007000	9.853092000
P	9.393081000	7.228475000	11.541786000
P	10.745478000	3.535421000	9.155019000
N	12.252244000	5.962656000	10.680745000
C	10.785819000	7.806988000	12.623798000
H	10.475836000	8.624998000	13.282134000
H	11.019286000	6.940446000	13.252093000
C	12.010124000	8.181607000	11.849340000
C	12.597457000	9.436762000	12.005705000
H	12.107885000	10.129755000	12.682358000
C	13.786587000	9.823394000	11.364456000
C	14.420333000	8.881125000	10.554301000
H	15.359187000	9.120719000	10.061203000
C	13.865475000	7.617682000	10.378933000
C	12.633999000	7.260309000	10.995683000
C	13.284325000	5.470325000	9.893181000
C	14.291081000	6.449523000	9.662236000
C	15.412377000	6.163991000	8.890255000
H	16.157732000	6.939533000	8.732008000
C	15.584096000	4.894308000	8.338855000
C	14.598711000	3.929040000	8.606479000
H	14.723451000	2.920833000	8.224952000
C	13.459472000	4.180822000	9.370352000
C	12.476637000	3.089675000	9.658574000
H	12.418015000	2.938005000	10.741858000
H	12.796893000	2.146909000	9.203392000
C	14.404948000	11.214133000	11.544586000
C	13.561967000	12.119891000	12.448507000
H	12.555588000	12.272640000	12.044411000
H	14.038273000	13.102210000	12.528812000
H	13.471530000	11.714399000	13.461309000
C	14.529019000	11.899490000	10.172275000
H	15.160635000	11.325351000	9.489228000
H	14.970667000	12.897113000	10.279544000
H	13.544830000	12.007209000	9.705045000
C	15.802371000	11.080378000	12.175106000
H	15.739255000	10.593743000	13.153576000
H	16.259050000	12.067815000	12.310164000
H	16.472026000	10.484088000	11.549500000
C	16.825577000	4.591003000	7.492808000
C	16.866179000	5.541340000	6.282775000
H	15.974028000	5.412311000	5.661599000
H	17.748733000	5.338213000	5.665230000
H	16.908056000	6.588970000	6.592604000
C	18.091848000	4.796319000	8.343088000
H	18.167908000	5.822918000	8.711241000
H	18.990276000	4.583909000	7.751950000
H	18.086261000	4.130747000	9.212116000
C	16.839436000	3.153175000	6.962765000
H	16.853789000	2.419750000	7.775289000
H	17.738565000	2.994122000	6.359075000
H	15.972914000	2.947924000	6.325322000
C	8.061082000	6.707699000	12.803828000
C	6.918743000	5.947461000	12.118044000
H	6.404436000	6.537968000	11.359517000
H	6.180492000	5.666428000	12.878909000
H	7.287274000	5.036442000	11.644160000
C	7.489342000	7.887228000	13.597080000
H	8.269557000	8.490953000	14.069567000
H	6.849043000	7.498380000	14.398182000
H	6.871713000	8.542344000	12.978134000
C	8.741737000	5.730850000	13.778615000
H	9.304632000	4.959504000	13.242169000
H	7.967773000	5.233350000	14.374634000
H	9.414384000	6.237004000	14.474764000
C	8.870388000	8.738746000	10.511339000
C	7.456601000	8.542480000	9.955196000
H	7.357349000	7.583865000	9.437142000
H	7.254090000	9.336786000	9.226856000
H	6.687676000	8.607127000	10.728781000
C	8.954706000	10.053961000	11.291858000
H	9.981729000	10.271786000	11.592153000
H	8.626218000	10.871469000	10.638519000
H	8.318832000	10.069283000	12.179441000
C	9.843585000	8.808369000	9.321240000
H	10.879225000	8.944450000	9.638874000
H	9.565172000	9.662719000	8.691379000
H	9.789874000	7.898630000	8.716922000
C	9.736410000	2.016155000	9.714022000
C	8.229381000	2.284812000	9.620066000
H	7.944293000	3.129810000	10.248374000

H	7.694604000	1.395445000	9.975082000
H	7.891757000	2.489185000	8.603763000
C	10.078003000	0.744100000	8.931245000
H	9.730555000	0.790632000	7.896454000
H	9.575582000	-0.108562000	9.403939000
H	11.150051000	0.527344000	8.927755000
C	10.070113000	1.798298000	11.200025000
H	11.074363000	1.395144000	11.349207000
H	9.361076000	1.073597000	11.616928000
H	9.976991000	2.729734000	11.768659000
C	10.785939000	3.728046000	7.262439000
C	9.368247000	3.617327000	6.691237000

H	8.980935000	2.595977000	6.725437000
H	9.392616000	3.920890000	5.637956000
H	8.670308000	4.279978000	7.211985000
C	11.711923000	2.720194000	6.573952000
H	12.751874000	2.864020000	6.874473000
H	11.661213000	2.882028000	5.490194000
H	11.431641000	1.682125000	6.762781000
C	11.302053000	5.149089000	6.975730000
H	10.656655000	5.902314000	7.436180000
H	11.302544000	5.306889000	5.889888000
H	12.318905000	5.304422000	7.340333000

Compound: $^{\mathrm{Cbz}}[^{t\mathrm{Bu}}\mathrm{PNP}]\mathrm{Pt^{II}Br}$ (1-Br)

Pt	13.619921000	5.698138000	16.568710000
Br	15.744524000	5.641022000	17.895420000
P	14.482627000	7.446201000	15.239940000
P	13.000528000	3.602453000	17.457453000
N	11.639572000	6.111176000	15.957511000
C	13.123715000	7.957456000	14.088271000
H	13.446459000	8.751045000	13.409426000
H	12.909118000	7.065144000	13.491498000
C	11.888226000	8.348643000	14.822958000
C	11.286992000	9.593110000	14.640225000
H	11.774942000	10.283076000	13.960213000
C	10.081126000	9.965469000	15.255413000
C	9.444522000	9.016497000	16.054874000
H	8.486448000	9.237836000	16.517678000
C	10.015394000	7.762670000	16.247843000
C	11.266741000	7.428113000	15.669130000
C	10.563353000	5.600041000	16.689311000
C	9.558935000	6.573850000	16.906813000
C	8.377649000	6.263548000	17.581117000
H	7.638691000	7.043308000	17.729399000
C	8.150847000	4.966898000	18.028966000
C	9.137780000	4.000258000	17.755516000
H	8.957681000	2.967359000	18.042982000
C	10.329908000	4.282135000	17.099278000
C	11.320558000	3.218239000	16.772511000
H	11.458526000	3.186368000	15.686865000
H	10.965421000	2.237561000	17.099610000
C	9.445977000	11.345260000	15.054185000
C	10.294395000	12.257288000	14.161896000
H	9.806779000	13.232554000	14.067189000
H	10.409729000	11.848351000	13.153014000
H	11.290528000	12.425954000	14.584454000
C	9.285885000	12.034305000	16.420811000
H	8.648107000	11.455041000	17.093764000
H	8.833416000	13.025187000	16.299562000
H	10.258757000	12.157648000	16.907234000
C	8.063502000	11.187262000	14.397208000
H	7.388714000	10.587048000	15.013414000
H	8.152003000	10.695585000	13.423268000
H	7.597093000	12.167600000	14.246505000
C	6.880617000	4.549031000	18.774059000
C	7.260448000	3.976490000	20.151796000
H	7.905301000	3.097667000	20.061637000
H	6.362086000	3.675853000	20.702716000
H	7.795035000	4.723854000	20.746658000
C	6.132882000	3.474640000	17.963989000
H	5.860024000	3.857093000	16.975577000
H	5.215251000	3.175600000	18.483074000
H	6.741041000	2.577356000	17.819360000
C	5.925473000	5.725861000	18.995939000
H	6.392396000	6.521073000	19.585673000
H	5.041943000	5.381969000	19.542855000

H	5.581865000	6.153243000	18.048700000
C	15.809252000	6.839811000	14.001378000
C	16.326155000	7.975147000	13.109176000
H	15.522147000	8.511388000	12.598127000
H	16.967569000	7.539387000	12.333475000
H	16.931430000	8.697254000	13.660869000
C	16.992401000	6.167338000	14.704894000
H	17.490940000	6.815476000	15.425069000
H	17.726813000	5.881835000	13.941649000
H	16.678354000	5.267612000	15.234233000
C	15.136959000	5.779412000	13.110880000
H	14.566910000	5.059634000	13.705584000
H	15.917085000	5.229249000	12.572618000
H	14.473179000	6.218195000	12.362608000
C	15.000517000	9.050724000	16.138672000
C	16.459781000	9.006939000	16.606774000
H	16.669359000	8.122397000	17.208423000
H	16.640825000	9.889688000	17.231992000
H	17.166708000	9.050280000	15.774595000
C	14.793858000	10.293862000	15.264590000
H	15.351098000	10.255366000	14.325683000
H	15.155231000	11.166339000	15.822066000
H	13.739664000	10.464845000	15.043899000
C	14.088828000	9.153209000	17.373297000
H	13.030145000	9.169493000	17.102507000
H	14.317029000	10.088164000	17.900296000
H	14.259810000	8.320922000	18.059790000
C	14.030891000	2.152330000	16.745769000
C	13.845982000	2.183833000	15.218859000
H	12.855296000	1.848839000	14.904217000
H	14.578964000	1.507333000	14.764693000
H	14.021303000	3.186980000	14.820000000
C	15.529623000	2.298168000	17.025701000
H	15.940974000	3.186820000	16.547692000
H	16.038608000	1.417999000	16.613559000
H	15.768114000	2.355913000	18.087285000
C	13.550373000	0.795427000	17.276354000
H	13.769487000	0.663158000	18.337760000
H	14.083674000	0.004982000	16.734558000
H	12.481753000	0.630217000	17.117120000
C	12.800240000	3.494984000	19.355906000
C	14.115403000	3.130942000	20.054685000
H	14.931365000	3.792774000	19.763270000
H	14.410936000	2.095007000	19.870663000
H	13.965188000	3.237260000	21.135924000
C	11.722637000	2.484177000	19.767331000
H	11.695757000	2.440862000	20.862676000
H	11.926133000	1.473207000	19.406901000
H	10.731208000	2.788032000	19.430906000
C	12.369618000	4.898523000	19.815938000
H	11.439490000	5.219854000	19.341169000
H	13.143513000	5.639597000	19.602361000

H	12.203930000	4.873853000	20.900164000

Compound: $^{Cbz}[^{tBu}PNP]Pt^{II}CH_2CH_2CHCH_2$ (4a)

Pt	13.634390000	5.577590000	16.630941000
P	14.498394000	7.291642000	15.271294000
P	12.918247000	3.464396000	17.361227000
N	11.579511000	6.023291000	15.900217000
C	13.139515000	7.824937000	14.115493000
H	13.493860000	8.589695000	13.418511000
H	12.896752000	6.925496000	13.541597000
C	11.916177000	8.268111000	14.837930000
C	11.377757000	9.545381000	14.693382000
H	11.893026000	10.232159000	14.030048000
C	10.196855000	9.958267000	15.333967000
C	9.521924000	9.022672000	16.119954000
H	8.585502000	9.285144000	16.605718000
C	10.025746000	7.734153000	16.270348000
C	11.250491000	7.352501000	15.656448000
C	10.503574000	5.546638000	16.641712000
C	9.537950000	6.549561000	16.915058000
C	8.376583000	6.257494000	17.632055000
H	7.667771000	7.054115000	17.831241000
C	8.129678000	4.957360000	18.063148000
C	9.074614000	3.964431000	17.732890000
H	8.873811000	2.932297000	18.010260000
C	10.248376000	4.228284000	17.038759000
C	11.219319000	3.167545000	16.656743000
H	11.358223000	3.201429000	15.572183000
H	10.857074000	2.171238000	16.925265000
C	9.629597000	11.373681000	15.178126000
C	10.508329000	12.265162000	14.294041000
H	10.068834000	13.265769000	14.231941000
H	10.586801000	11.876387000	13.273700000
H	11.518454000	12.372824000	14.703243000
C	9.526735000	12.036790000	16.563117000
H	8.873812000	11.472347000	17.234086000
H	9.121185000	13.051434000	16.474469000
H	10.513159000	12.100487000	17.033789000
C	8.230422000	11.304446000	14.541117000
H	7.535253000	10.725676000	15.155085000
H	8.278430000	10.830842000	13.555462000
H	7.813252000	12.310946000	14.419883000
C	6.880220000	4.561824000	18.855229000
C	7.301411000	3.939725000	20.199105000
H	7.910990000	3.042784000	20.056678000
H	6.419431000	3.654878000	20.784170000
H	7.888946000	4.652692000	20.786387000
C	6.059767000	3.535314000	18.053817000
H	5.748585000	3.957210000	17.093104000
H	5.160978000	3.244268000	18.609738000
H	6.634038000	2.627389000	17.848976000
C	5.977103000	5.762724000	19.152996000
H	6.498913000	6.524708000	19.740594000
H	5.107988000	5.433382000	19.731429000
H	5.605730000	6.228426000	18.234829000
C	15.789890000	6.644200000	14.010571000
C	16.391822000	7.737350000	13.122863000
H	15.630389000	8.327008000	12.605533000
H	17.005900000	7.256066000	12.351522000
H	17.043624000	8.416481000	13.676244000
C	16.910859000	5.897653000	14.732921000
H	17.463852000	6.534596000	15.425332000
H	17.623289000	5.513916000	13.992509000
H	16.501858000	5.051124000	15.286825000
C	15.067496000	5.629301000	13.107254000
H	14.452393000	4.939194000	13.690727000
H	15.819176000	5.039609000	12.569864000
H	14.436263000	6.112434000	12.358614000
C	15.077916000	8.920959000	16.092724000
C	16.516469000	8.808817000	16.617355000
H	16.686480000	7.931386000	17.243181000
H	16.730189000	9.690842000	17.232836000
H	17.248143000	8.797390000	15.805524000
C	14.996912000	10.126784000	15.146461000
H	15.596965000	10.006757000	14.243644000
H	15.378153000	11.006812000	15.678817000
H	13.967455000	10.344460000	14.860321000
C	14.114377000	9.188060000	17.264369000
H	13.084447000	9.305807000	16.920878000
H	14.416095000	10.121882000	17.754963000
H	14.128531000	8.394451000	18.012467000
C	13.882359000	2.033315000	16.519086000
C	13.633866000	2.155699000	15.005750000
H	12.642930000	1.802883000	14.713211000
H	14.369120000	1.536773000	14.478718000
H	13.754054000	3.186988000	14.663771000
C	15.385559000	2.182774000	16.747538000
H	15.737616000	3.144708000	16.371236000
H	15.910287000	1.386823000	16.205049000
H	15.659289000	2.097035000	17.800349000
C	13.438783000	0.640258000	16.977187000
H	13.719825000	0.434473000	18.012010000
H	13.940981000	-0.107412000	16.350754000
H	12.362806000	0.482338000	16.867750000
C	12.693203000	3.169059000	19.238959000
C	14.029449000	2.830596000	19.911256000
H	14.815480000	3.553694000	19.689182000
H	14.387133000	1.834729000	19.636978000
H	13.884075000	2.833791000	20.998195000
C	11.681503000	2.061507000	19.561750000
H	11.638243000	1.943676000	20.651387000
H	11.949716000	1.091977000	19.139770000
H	10.677375000	2.325311000	19.227981000
C	12.141103000	4.483008000	19.822111000
H	11.166038000	4.733030000	19.399850000
H	12.806983000	5.329956000	19.651818000
H	12.018627000	4.357686000	20.905197000
C	15.180971000	5.747569000	18.062044000
H	15.325172000	6.827311000	18.119592000
H	14.653967000	5.491639000	18.986706000
C	16.561790000	5.116172000	18.151251000
H	17.159960000	5.317979000	17.256784000
H	16.501050000	4.024397000	18.197620000
C	17.373604000	5.561667000	19.338996000
H	18.370034000	5.118089000	19.397680000
C	17.007863000	6.413573000	20.294163000
H	16.033704000	6.892917000	20.304862000
H	17.680858000	6.660795000	21.109563000

Compound: $^{Cbz}[^{tBu}PNP]Pt^{II}CH_2CH(CH_2)_4$ (4b)

Pt	13.834871000	5.677995000	16.650380000
P	14.599129000	7.403337000	15.232232000
P	13.059323000	3.676443000	17.615918000
N	11.722073000	6.134565000	16.004472000
C	13.160252000	7.968473000	14.194924000
H	13.477345000	8.743307000	13.492827000

H	12.872782000	7.084733000	13.618385000
C	11.970503000	8.398564000	14.975394000
C	11.382689000	9.653550000	14.833544000
H	11.869916000	10.363407000	14.173282000
C	10.172627000	10.008125000	15.456201000
C	9.511623000	9.026968000	16.196088000
H	8.541817000	9.233734000	16.641891000
C	10.067858000	7.758888000	16.339295000
C	11.340104000	7.452081000	15.784525000
C	10.619517000	5.577888000	16.639551000
C	9.591374000	6.525628000	16.890527000
C	8.395474000	6.154308000	17.506383000
H	7.642235000	6.913222000	17.689123000
C	8.172339000	4.826385000	17.858205000
C	9.188138000	3.894049000	17.567525000
H	9.032160000	2.846571000	17.816564000
C	10.398916000	4.239776000	16.979829000
C	11.467677000	3.232962000	16.759318000
H	11.736382000	3.178238000	15.695866000
H	11.126330000	2.245025000	17.072139000
C	9.551461000	11.401888000	15.311195000
C	10.416696000	12.346681000	14.470220000
H	9.940877000	13.331263000	14.419550000
H	10.535045000	11.986120000	13.443355000
H	11.411673000	12.482246000	14.907219000
C	9.383592000	12.030286000	16.705804000
H	8.738643000	11.423179000	17.346607000
H	8.936552000	13.028223000	16.626924000
H	10.353366000	12.126163000	17.204735000
C	8.173746000	11.291030000	14.634439000
H	7.484971000	10.674671000	15.218158000
H	8.266133000	10.838969000	13.641805000
H	7.721369000	12.283090000	14.520420000
C	6.887794000	4.344994000	18.538436000
C	7.234361000	3.716575000	19.900874000
H	7.907673000	2.861484000	19.792096000
H	6.326587000	3.365541000	20.405126000
H	7.726419000	4.449080000	20.548953000
C	6.193210000	3.293364000	17.654924000
H	5.943059000	3.716693000	16.677183000
H	5.266926000	2.947461000	18.128158000
H	6.828936000	2.419388000	17.487763000
C	5.897267000	5.487509000	18.782688000
H	6.323342000	6.261970000	19.428242000
H	5.002664000	5.097592000	19.278674000
H	5.579950000	5.954982000	17.845303000
C	15.756899000	6.808699000	13.815813000
C	16.200232000	7.935621000	12.877821000
H	15.363129000	8.496107000	12.454933000
H	16.749466000	7.492478000	12.037729000
H	16.876962000	8.637709000	13.371423000
C	16.997614000	6.101915000	14.353588000
H	17.658480000	6.771119000	14.907967000
H	17.571807000	5.703381000	13.508042000
H	16.720240000	5.268519000	14.998059000
C	14.960129000	5.762324000	13.012526000
H	14.466904000	5.038804000	13.670183000
H	15.658243000	5.213967000	12.369781000

H	14.208980000	6.213286000	12.361943000
C	15.173044000	9.044942000	16.049531000
C	16.631573000	8.998211000	16.507640000
H	16.796425000	8.226121000	17.257696000
H	16.879886000	9.959904000	16.972610000
H	17.332193000	8.846952000	15.681472000
C	14.984677000	10.264248000	15.137750000
H	15.547648000	10.195677000	14.205322000
H	15.346610000	11.150454000	15.672944000
H	13.935311000	10.439543000	14.904005000
C	14.278817000	9.224125000	17.288297000
H	13.221970000	9.297002000	17.024076000
H	14.566881000	10.153083000	17.796724000
H	14.395877000	8.397548000	17.992794000
C	14.099509000	2.129500000	17.121367000
C	14.671373000	2.454501000	15.732941000
H	13.877442000	2.589533000	14.992031000
H	15.301991000	1.621386000	15.397815000
H	15.262736000	3.371024000	15.745812000
C	15.238797000	1.827249000	18.100522000
H	15.830816000	2.703309000	18.357101000
H	15.916328000	1.099199000	17.637842000
H	14.860884000	1.378043000	19.022793000
C	13.260844000	0.846947000	16.993653000
H	12.719266000	0.595756000	17.908496000
H	13.945887000	0.016796000	16.783304000
H	12.548182000	0.890654000	16.168556000
C	12.626036000	3.718288000	19.486826000
C	13.860100000	3.864554000	20.390019000
H	14.359127000	4.822231000	20.238328000
H	14.592410000	3.065282000	20.281578000
H	13.510300000	3.843777000	21.429375000
C	11.847506000	2.469645000	19.911006000
H	11.529093000	2.590518000	20.953630000
H	12.461528000	1.565762000	19.861598000
H	10.944788000	2.319911000	19.315164000
C	11.770267000	4.976404000	19.729802000
H	10.770858000	4.902661000	19.306488000
H	12.252537000	5.870442000	19.321374000
H	11.670359000	5.113066000	20.813663000
C	15.554072000	5.554865000	17.885518000
H	17.184124000	5.731316000	19.780041000
C	17.763586000	4.334242000	17.139212000
C	18.611763000	3.861967000	18.325649000
C	19.086506000	5.179476000	18.938564000
C	17.807736000	6.018197000	18.922595000
H	17.061705000	3.576868000	16.787286000
H	18.435864000	4.552076000	16.300765000
H	19.424146000	3.190094000	18.030267000
H	17.991959000	3.326211000	19.057713000
H	19.851732000	5.631202000	18.293507000
H	19.517086000	5.070487000	19.939290000
H	17.987440000	7.095584000	19.007400000
C	17.062334000	5.643484000	17.604204000
H	15.450884000	4.675147000	18.522200000
H	15.308304000	6.401736000	18.550228000
H	17.263720000	6.416513000	16.868486000

Compound: $^{Cbz}[^{tBu}PNP]Pt^{II}CH_2CHCH_2$ (4c)

Pt	13.704235000	5.653470000	16.707123000
P	14.525747000	7.401622000	15.369878000
P	12.983942000	3.526979000	17.444996000
N	11.637112000	6.093880000	16.019604000
C	13.184481000	7.838992000	14.155981000
H	13.533032000	8.586928000	13.439302000

H	12.992341000	6.907792000	13.612324000
C	11.926939000	8.276540000	14.825170000
C	11.338478000	9.515439000	14.574955000
H	11.832193000	10.166989000	13.861685000
C	10.134634000	9.931276000	15.168776000
C	9.489367000	9.036357000	16.023171000

H	8.538511000	9.298909000	16.479855000
C	10.037044000	7.781814000	16.272189000
C	11.280298000	7.397490000	15.696214000
C	10.557869000	5.630501000	16.763589000
C	9.569276000	6.624169000	16.977085000
C	8.397473000	6.336354000	17.679341000
H	7.670352000	7.126215000	17.834562000
C	8.159907000	5.045688000	18.142559000
C	9.119024000	4.054421000	17.848855000
H	8.915791000	3.024542000	18.132329000
C	10.302698000	4.316885000	17.172133000
C	11.267366000	3.258882000	16.771834000
H	11.384386000	3.308206000	15.685755000
H	10.905424000	2.260512000	17.031104000
C	9.510785000	11.303162000	14.889339000
C	10.365230000	12.155883000	13.945736000
H	9.884392000	13.127385000	13.793228000
H	10.478085000	11.687341000	12.962840000
H	11.362621000	12.342095000	14.358117000
C	9.356057000	12.072637000	16.213246000
H	8.713987000	11.538195000	16.918360000
H	8.911106000	13.058322000	16.034377000
H	10.330190000	12.216297000	16.691764000
C	8.126619000	11.119135000	14.242641000
H	7.449345000	10.556771000	14.891057000
H	8.211862000	10.573764000	13.297418000
H	7.665282000	12.092380000	14.038399000
C	6.900654000	4.654744000	18.920888000
C	7.304951000	4.074987000	20.288752000
H	7.932006000	3.185212000	20.181344000
H	6.416310000	3.790667000	20.863949000
H	7.869114000	4.812671000	20.868363000
C	6.108605000	3.595589000	18.133160000
H	5.815755000	3.984202000	17.152876000
H	5.200117000	3.313067000	18.677461000
H	6.695266000	2.687090000	17.970588000
C	5.976132000	5.850772000	19.168160000
H	6.477226000	6.637237000	19.741374000
H	5.101975000	5.526192000	19.741648000
H	5.613815000	6.283708000	18.230523000
C	15.912931000	6.813030000	14.195182000
C	16.405709000	7.909295000	13.245082000
H	15.598888000	8.346969000	12.651217000
H	17.119246000	7.463570000	12.541258000
H	16.928842000	8.712923000	13.767010000
C	17.084558000	6.259290000	15.004744000
H	17.553969000	7.014180000	15.636542000
H	17.851852000	5.883380000	14.316522000
H	16.768352000	5.434078000	15.642429000
C	15.339928000	5.656290000	13.362710000
H	14.875047000	4.903064000	14.004233000
H	16.162878000	5.177953000	12.818592000
H	14.608496000	5.987815000	12.622274000
C	14.969178000	9.079480000	16.200534000
C	16.423310000	9.177229000	16.683881000
H	16.712274000	8.382335000	17.372067000
H	16.531375000	10.123668000	17.227198000
H	17.137464000	9.197830000	15.857803000
C	14.717524000	10.265999000	15.259899000
H	15.290430000	10.203920000	14.331886000
H	15.028198000	11.183587000	15.774177000
H	13.661593000	10.373885000	15.017807000
C	14.028052000	9.203396000	17.409980000
H	12.977114000	9.165560000	17.113120000
H	14.206092000	10.168584000	17.900784000
H	14.202164000	8.410644000	18.141620000
C	13.882667000	2.065548000	16.567044000
C	13.576279000	2.203092000	15.064217000
H	12.571657000	1.861168000	14.808381000
H	14.283551000	1.579937000	14.505578000
H	13.692444000	3.234758000	14.720810000
C	15.399626000	2.149578000	16.730222000
H	15.786332000	3.111829000	16.389661000
H	15.861867000	1.359297000	16.126277000
H	15.718706000	1.991196000	17.761901000
C	13.409753000	0.688412000	17.043540000
H	13.717772000	0.478646000	18.070425000
H	13.869483000	-0.075887000	16.404764000
H	12.326733000	0.562170000	16.969704000
C	12.742486000	3.239019000	19.329450000
C	14.056332000	2.889699000	20.032668000
H	14.783140000	3.698734000	19.965294000
H	14.512532000	1.971179000	19.653875000
H	13.849564000	2.733596000	21.098160000
C	11.708088000	2.150448000	19.643295000
H	11.627015000	2.059454000	20.733212000
H	11.982510000	1.168869000	19.254310000
H	10.717823000	2.413362000	19.272205000
C	12.222990000	4.570517000	19.899659000
H	11.255129000	4.846534000	19.478233000
H	12.918582000	5.391891000	19.715342000
H	12.102494000	4.461307000	20.984844000
C	15.405804000	5.799856000	17.977447000
H	15.892127000	6.753071000	17.777751000
H	14.853646000	5.925921000	18.922755000
C	16.469736000	4.798910000	18.204140000
H	16.153504000	3.828965000	18.567833000
C	17.785139000	4.992677000	18.068910000
H	18.195852000	5.939778000	17.727760000
H	18.499761000	4.209264000	18.304416000

Compound: $^{Cbz}[^{tBu}PNP]Pt^{II}CH_2CH_2CH_2CH_3$ (4d)

Pt	13.682901000	5.573997000	16.623457000
P	14.513123000	7.314466000	15.270718000
P	12.941962000	3.473683000	17.374381000
N	11.607146000	6.049724000	15.950323000
C	13.147850000	7.807684000	14.105288000
H	13.495643000	8.558330000	13.390133000
H	12.915248000	6.891179000	13.553475000
C	11.921019000	8.260945000	14.816564000
C	11.361854000	9.522618000	14.620913000
H	11.860797000	10.186481000	13.922735000
C	10.178317000	9.945644000	15.249778000
C	9.522617000	9.035426000	16.080087000
H	8.584886000	9.304337000	16.559778000
C	10.043486000	7.760221000	16.277399000
C	11.269432000	7.368395000	15.670715000
C	10.532418000	5.586121000	16.700384000
C	9.564154000	6.591259000	16.955059000
C	8.401630000	6.308413000	17.674299000
H	7.691362000	7.106999000	17.860211000
C	8.153288000	5.013478000	18.119664000
C	9.095076000	4.015080000	17.796298000
H	8.887588000	2.984616000	18.074894000
C	10.269946000	4.271055000	17.101573000
C	11.226043000	3.204206000	16.702247000
H	11.344197000	3.240821000	15.615304000
H	10.858823000	2.210738000	16.973807000
C	9.585335000	11.342044000	15.031240000
C	10.449081000	12.209498000	14.109595000

H	9.991718000	13.198269000	14.002641000
H	10.536252000	11.777695000	13.107437000
H	11.456484000	12.353246000	14.514556000
C	9.466610000	12.063549000	16.385343000
H	8.821875000	11.517236000	17.078835000
H	9.042792000	13.065594000	16.251003000
H	10.450332000	12.165871000	16.854836000
C	8.189050000	11.217729000	14.396298000
H	7.505366000	10.649106000	15.032375000
H	8.248618000	10.706009000	13.430486000
H	7.750906000	12.209520000	14.234326000
C	6.902633000	4.627248000	18.914326000
C	7.322303000	4.016364000	20.263806000
H	7.930775000	3.117483000	20.129132000
H	6.439741000	3.737832000	20.851079000
H	7.910808000	4.733546000	20.844965000
C	6.080607000	3.594915000	18.121976000
H	5.771617000	4.008049000	17.156753000
H	5.180427000	3.311366000	18.679561000
H	6.652735000	2.683519000	17.926846000
C	6.001543000	5.832388000	19.200717000
H	6.525222000	6.599553000	19.779857000
H	5.132408000	5.510424000	19.783307000
H	5.629977000	6.289233000	18.278176000
C	15.835755000	6.717922000	14.025766000
C	16.395752000	7.832934000	13.136927000
H	15.612345000	8.383042000	12.609008000
H	17.038446000	7.376357000	12.374013000
H	17.009446000	8.546546000	13.689807000
C	16.974084000	6.018663000	14.769700000
H	17.445231000	6.646145000	15.528654000
H	17.747686000	5.722554000	14.050655000
H	16.595536000	5.119458000	15.255890000
C	15.167265000	5.666071000	13.124179000
H	14.590564000	4.947411000	13.713239000
H	15.949071000	5.114475000	12.588865000
H	14.511161000	6.112945000	12.374135000
C	15.035472000	8.963749000	16.101382000
C	16.495557000	8.958889000	16.574355000
H	16.738435000	8.116196000	17.221471000
H	16.667864000	9.872686000	17.155782000
H	17.203903000	8.967410000	15.742947000
C	14.843504000	10.172321000	15.175205000
H	15.413416000	10.095418000	14.247241000
H	15.196275000	11.067675000	15.701682000
H	13.793331000	10.333218000	14.934321000
C	14.105510000	9.137333000	17.314702000
H	13.055446000	9.176995000	17.015786000
H	14.350290000	10.083223000	17.814166000
H	14.217797000	8.329003000	18.040462000
C	13.849221000	2.008124000	16.526748000
C	13.585097000	2.137452000	15.016444000
H	12.578685000	1.821025000	14.735676000
H	14.290898000	1.491176000	14.482001000
H	13.739148000	3.163672000	14.670929000
C	15.358962000	2.105351000	16.733760000
H	15.740201000	3.057708000	16.363285000
H	15.847068000	1.296424000	16.176408000
H	15.645278000	2.000300000	17.780934000
C	13.366392000	0.632809000	16.998047000
H	13.653750000	0.425991000	18.031263000
H	13.838291000	-0.134206000	16.371426000
H	12.284938000	0.506798000	16.902912000
C	12.721350000	3.205742000	19.259100000
C	14.050700000	2.850102000	19.930369000
H	14.829588000	3.582870000	19.720122000
H	14.414298000	1.860884000	19.639858000
H	13.901685000	2.835555000	21.016924000
C	11.685628000	2.127814000	19.603172000
H	11.644598000	2.028157000	20.694712000
H	11.931670000	1.146012000	19.195868000
H	10.686194000	2.407465000	19.269528000
C	12.216049000	4.544327000	19.826767000
H	11.252772000	4.828894000	19.400049000
H	12.918392000	5.360289000	19.647525000
H	12.087489000	4.435714000	20.911075000
C	15.337685000	5.687692000	17.944884000
H	15.653111000	6.730358000	17.878435000
H	14.808294000	5.622973000	18.903758000
C	16.622848000	4.867635000	18.072565000
H	17.115016000	4.729981000	17.107159000
H	16.414431000	3.861164000	18.444007000
C	17.640336000	5.521232000	19.022363000
H	17.905958000	6.516765000	18.639397000
H	18.566534000	4.931001000	19.000420000
C	17.162220000	5.645897000	20.466003000
H	16.884506000	4.666098000	20.872327000
H	17.946235000	6.059297000	21.108351000
H	16.288998000	6.299224000	20.550196000

Compound: $^{Cbz}[^{tBu}PNP]Pt^{II}CH_3$ (4e)

Pt	13.677433000	5.672927000	16.582851000
P	14.494348000	7.432377000	15.285635000
P	13.014870000	3.595035000	17.418860000
N	11.610384000	6.107955000	15.943581000
C	13.142099000	7.920792000	14.104948000
H	13.477715000	8.699705000	13.414364000
H	12.944414000	7.013350000	13.525292000
C	11.886829000	8.324558000	14.801536000
C	11.297809000	9.571637000	14.599156000
H	11.799567000	10.253967000	13.920903000
C	10.086061000	9.958996000	15.196685000
C	9.433969000	9.026993000	16.004796000
H	8.477553000	9.268122000	16.461977000
C	9.986998000	7.767237000	16.213952000
C	11.238796000	7.411012000	15.638602000
C	10.540512000	5.615724000	16.680704000
C	9.532526000	6.591362000	16.896753000
C	8.367522000	6.285303000	17.602062000
H	7.625425000	7.062010000	17.753028000
C	8.159588000	4.995666000	18.080785000
C	9.146450000	4.026528000	17.807088000
H	8.974830000	2.998264000	18.117084000
C	10.322124000	4.302920000	17.120817000
C	11.309050000	3.242128000	16.770165000
H	11.427203000	3.228978000	15.682112000
H	10.960812000	2.254440000	17.086030000
C	9.461575000	11.340026000	14.968618000
C	10.326679000	12.236003000	14.076101000
H	9.845045000	13.212199000	13.960511000
H	10.454882000	11.811993000	13.075013000
H	11.317621000	12.405801000	14.510541000
C	9.284788000	12.050728000	16.322131000
H	8.633908000	11.485009000	16.994030000
H	8.839735000	13.042416000	16.179865000
H	10.251257000	12.175603000	16.821058000
C	8.087720000	11.180998000	14.293764000
H	7.402489000	10.590104000	14.907469000
H	8.188526000	10.676563000	13.327544000

H	7.626984000	12.161246000	14.124226000
C	6.907124000	4.584064000	18.859509000
C	7.316861000	4.043579000	20.241616000
H	7.971689000	3.171508000	20.157008000
H	6.432232000	3.743003000	20.814834000
H	7.852676000	4.809520000	20.811395000
C	6.151347000	3.487213000	18.087773000
H	5.856000000	3.847288000	17.097383000
H	5.246550000	3.190955000	18.630986000
H	6.764367000	2.592335000	17.948742000
C	5.946099000	5.757225000	19.075610000
H	6.417728000	6.568660000	19.638956000
H	5.075966000	5.417625000	19.646556000
H	5.581251000	6.161792000	18.126315000
C	15.865477000	6.881650000	14.064911000
C	16.395685000	8.033692000	13.204276000
H	15.599290000	8.571177000	12.683008000
H	17.062269000	7.619665000	12.437679000
H	16.978225000	8.753201000	13.784046000
C	17.039144000	6.208305000	14.785523000
H	17.534008000	6.860871000	15.506534000
H	17.786479000	5.921710000	14.035625000
H	16.712916000	5.304439000	15.302187000
C	15.234039000	5.822707000	13.144082000
H	14.681700000	5.074182000	13.720493000
H	16.034504000	5.308965000	12.599082000
H	14.562961000	6.259130000	12.401383000
C	14.947550000	9.051091000	16.198170000
C	16.378933000	9.030756000	16.747290000
H	16.560514000	8.184015000	17.410553000
H	16.530911000	9.943037000	17.336760000
H	17.133082000	9.029257000	15.956703000
C	14.774845000	10.293310000	15.315736000
H	15.389650000	10.267809000	14.413593000
H	15.080359000	11.173456000	15.894673000
H	13.733437000	10.441353000	15.029247000
C	13.971035000	9.150870000	17.382529000
H	12.929880000	9.185568000	17.053878000
H	14.182921000	10.073662000	17.937300000
H	14.082350000	8.304483000	18.065189000
C	13.993437000	2.129991000	16.662496000
C	13.709056000	2.140614000	15.149429000
H	12.717182000	1.752672000	14.908402000
H	14.441575000	1.494797000	14.651432000
H	13.804583000	3.146396000	14.728654000
C	15.505210000	2.303141000	16.838341000
H	15.856454000	3.191726000	16.311284000
H	16.007688000	1.429699000	16.405061000
H	15.811582000	2.372614000	17.883572000
C	13.572041000	0.770498000	17.231146000
H	13.877686000	0.640246000	18.271707000
H	14.063128000	-0.018568000	16.648338000
H	12.494631000	0.599983000	17.160786000
C	12.845007000	3.448452000	19.316750000
C	14.189462000	3.138115000	19.985142000
H	14.961525000	3.862552000	19.721963000
H	14.551929000	2.134803000	19.748700000
H	14.052807000	3.184838000	21.072402000
C	11.817335000	2.392751000	19.742200000
H	11.795985000	2.352552000	20.838064000
H	12.057972000	1.390183000	19.383658000
H	10.812449000	2.655345000	19.409653000
C	12.356719000	4.823568000	19.805682000
H	11.397692000	5.098371000	19.361570000
H	13.076102000	5.612484000	19.575010000
H	12.226786000	4.781729000	20.894429000
C	15.447473000	5.614203000	17.687989000
H	15.192407000	6.078494000	18.652288000
H	15.843245000	4.622910000	17.902360000
H	16.274238000	6.184863000	17.271501000

Compound: ^{Cbz}[^{tBu}PNP]Pt^{I} (2*)

Pt	10.211003000	5.443487000	10.344174000
P	9.411973000	7.215378000	11.581330000
P	10.768282000	3.540716000	9.170283000
N	12.302980000	5.921552000	10.785477000
C	10.801310000	7.798730000	12.671884000
H	10.493628000	8.620340000	13.327432000
H	11.033446000	6.933455000	13.302196000
C	12.026249000	8.167121000	11.892375000
C	12.598661000	9.433061000	12.013962000
H	12.107968000	10.134803000	12.680385000
C	13.771335000	9.823372000	11.345293000
C	14.404682000	8.876820000	10.538356000
H	15.331257000	9.121263000	10.025049000
C	13.863385000	7.604392000	10.394093000
C	12.654585000	7.240684000	11.048147000
C	13.309562000	5.435556000	9.959410000
C	14.292104000	6.427383000	9.687729000
C	15.401043000	6.144993000	8.898345000
H	16.132530000	6.927034000	8.710948000
C	15.576659000	4.868088000	8.363001000
C	14.607014000	3.895695000	8.659644000
H	14.731159000	2.887344000	8.279031000
C	13.478484000	4.143594000	9.440822000
C	12.482249000	3.057924000	9.712591000
H	12.398594000	2.906306000	10.794207000
H	12.802806000	2.113175000	9.260995000
C	14.372148000	11.225825000	11.488957000
C	13.532599000	12.136163000	12.391598000
H	12.516981000	12.267266000	12.003443000
H	13.996196000	13.126326000	12.444767000
H	13.466714000	11.748505000	13.413214000
C	14.461342000	11.887503000	10.102242000
H	15.088428000	11.310185000	9.417715000
H	14.890672000	12.892814000	10.183850000
H	13.467333000	11.973465000	9.651600000
C	15.782541000	11.122684000	12.096299000
H	15.743879000	10.650779000	13.083156000
H	16.226014000	12.118939000	12.207722000
H	16.450071000	10.526927000	11.467940000
C	16.799647000	4.570191000	7.488798000
C	16.784183000	5.496354000	6.259286000
H	15.872995000	5.340854000	5.672772000
H	17.647785000	5.293362000	5.615597000
H	16.820116000	6.550518000	6.546984000
C	18.085624000	4.817147000	8.297161000
H	18.152005000	5.851278000	8.645360000
H	18.969735000	4.612572000	7.682316000
H	18.120294000	4.166518000	9.176743000
C	16.826757000	3.122034000	6.988197000
H	16.868746000	2.406468000	7.815518000
H	17.716775000	2.965293000	6.370634000
H	15.952137000	2.889266000	6.371675000
C	8.065672000	6.688949000	12.826498000
C	6.979835000	5.889331000	12.095249000
H	6.432573000	6.483501000	11.362652000
H	6.255874000	5.515190000	12.829514000
H	7.416505000	5.031818000	11.575609000
C	7.433701000	7.850973000	13.596168000

H	8.182404000	8.472653000	14.096484000
H	6.772561000	7.447961000	14.373474000
H	6.825396000	8.491916000	12.953883000
C	8.746178000	5.737885000	13.825632000
H	9.339267000	4.974081000	13.310849000
H	7.971962000	5.228518000	14.411302000
H	9.392180000	6.268177000	14.529364000
C	8.893115000	8.729557000	10.551483000
C	7.497304000	8.504570000	9.961134000
H	7.433533000	7.544328000	9.439081000
H	7.292245000	9.296038000	9.230364000
H	6.709194000	8.547739000	10.716572000
C	8.930507000	10.042477000	11.338870000
H	9.942187000	10.274798000	11.678275000
H	8.614874000	10.859072000	10.677758000
H	8.262245000	10.044243000	12.202388000
C	9.889166000	8.825670000	9.382421000
H	10.911039000	9.006169000	9.720657000
H	9.591649000	9.662075000	8.737176000
H	9.885502000	7.908599000	8.786269000
C	9.703414000	2.040488000	9.672112000
C	8.216728000	2.398804000	9.545565000
H	7.989585000	3.314786000	10.098370000
H	7.616335000	1.583897000	9.968331000
H	7.898582000	2.540645000	8.512446000
C	10.002718000	0.763201000	8.884321000
H	9.693879000	0.838824000	7.839024000
H	9.443441000	-0.069984000	9.327855000
H	11.063008000	0.494522000	8.913702000
C	9.992699000	1.792405000	11.162211000
H	10.984971000	1.366788000	11.329680000
H	9.258761000	1.076171000	11.549722000
H	9.905077000	2.717495000	11.742449000
C	10.867027000	3.759804000	7.279997000
C	9.456930000	3.714210000	6.681632000
H	9.028148000	2.709097000	6.694985000
H	9.511124000	4.031111000	5.633360000
H	8.777095000	4.397197000	7.201221000
C	11.762296000	2.723986000	6.593530000
H	12.798235000	2.805718000	6.928758000
H	11.754627000	2.913380000	5.512929000
H	11.423612000	1.697572000	6.748004000
C	11.442378000	5.163197000	7.020335000
H	10.838351000	5.933734000	7.508312000
H	11.434439000	5.347484000	5.938593000
H	12.469994000	5.264559000	7.372097000

ii. QTAIM

Electron density ρ(r): The electron density at a point r in space, representing the probability of finding an electron at that location. It provides information about bonding and charge distribution in molecules.

Laplacian of the electron density $\nabla^2\rho(r)$: The second derivative of the electron density with respect to spatial coordinates. It indicates regions of electron concentration ($\nabla^2\rho < 0$) and depletion ($\nabla^2\rho > 0$), helping to distinguish between covalent and non-covalent interactions.

Energy density analysis H(r) (H(r) = V(r) + G(r)): The total energy density at a point r, where V(r) is the potential energy density and G(r) is the kinetic energy density. It helps assess the nature of chemical bonds; H(r) < 0 usually indicates shared (covalent) interactions, while H(r) > 0 corresponds to closed-shell (ionic or van der Waals) interactions.

Ellipticity ε: A measure of the deviation of the electron density from cylindrical symmetry around a bond path. It provides information about π-character and bond multiplicity; higher ellipticity indicates stronger π-contributions or bond instability.

Table 10: Summary of decisive parameters for classification of BCPs: Electron density ρ(r) [a_0^{-3}], Laplacian of the electron density $\nabla^2\rho(r)$ [a_0^{-5}], energy density analysis H(r) [Ha/a_0^3] (H(r)= V(r) + G(r)) and ellipticity ε.

BCP	ρ(r) [a_0^{-3}]	$\nabla^2\rho(r)$ [a_0^{-5}]	H(r) [Ha/a_0^3]	ε [a.u.]
322 (Pt-Na)	0.0182	0.0799	0.0025	0.0550
457 (Pt-Na)	0.0182	0.0800	0.0025	0.0552
375 (Pt-N)	0.0283	0.0830	-0.0001	0.1304
403 (Pt-N)	0.0283	0.0829	-0.0001	0.1304
390 (N-N)	0.0077	0.0194	0.0006	1.3767
363 (N-Na)	0.0239	0.1342	0.0053	0.1471
415 (N-Na)	0.0239	0.1346	0.0053	0.1466
334 (N-Na)	0.0169	0.0934	0.0040	0.3125
445 (N-Na)	0.0169	0.0933	0.0040	0.3122
203 (Pt-Mg)	0.0217	0.0680	-0.0007	0.6651
237 (Pt-N)	0.0290	0.0834	-0.0011	0.6245
228 (N-Mg)	0.0462	0.2721	0.0048	0.1115
176 (Cl-Mg)	0.0381	0.1907	0.0023	0.0056

iii. CAS-SCF

Table 11: Configurations for the complete active space used for the T-shaped platinum(I) intermediate **2*** (CAS(9,5)).

CASSCF states for (MULT=2 NROOTS=5)	NEVPT2 Results
ROOT 0: E= -21249.1535518134 Eh<br>0.93433 [3]: 22212<br>0.06559 [0]: 12222	Total Energy Correction : dE = -7.60793219749030<br>Reference Energy : E0 = 21249.15355181335690<br>Total Energy (E0+dE) : E = -21256.76148401084720
ROOT 1: E= -21249.1120136082 Eh<br>0.99581 [1]: 21222<br>0.00404 [0]: 12222	Total Energy Correction : dE = -7.59616596715205<br>Reference Energy : E0 = -21249.11201360815176<br>Total Energy (E0+dE) : E = -21256.70817957530380
ROOT 2: E= -21249.1008772364 Eh<br>0.93035 [0]: 12222<br>0.06560 [3]: 22212<br>0.00401 [1]: 21222	Total Energy Correction : dE = -7.58964273533275<br>Reference Energy : E0 = -21249.10087723640754<br>Total Energy (E0+dE) : E = -21256.69051997174029
ROOT 3: E= -21249.0793857869 Eh<br>0.99922 [4]: 22221	Total Energy Correction : dE = -7.58980863079705<br>Reference Energy : E0 = -21249.07938578690300<br>Total Energy (E0+dE) : E = -21256.66919441770006
ROOT 4: E= -21249.0714980159 Eh<br>0.99930 [2]: 22122	Total Energy Correction : dE = -7.58889146885485<br>Reference Energy : E0 = -21249.07149801593914<br>Total Energy (E0+dE) : E = -21256.66038948479400

Table 12: SC-NEVPT2 corrected AILFT (one electron eigenfunctions) results (CAS(9,5)). SOC constant = 3498.8 cm^{-1}.

Orbital	Energy [ev]	Energy [cm^{-1}]	d_{z^2}	d_{xz}	d_{yz}	$d_{x^2-y^2}$	d_{xy}
1	0.000	0.0	-0.003956	0.000300	-0.999649	-0.003517	-0.025974
2	0.240	1932.5	-0.003416	-0.009516	-0.025962	-0.000284	0.999612
3	0.820	6612.9	0.964544	0.063363	-0.004799	0.256128	0.003847
4	1.300	10488.7	0.063569	-0.997905	-0.000337	0.007609	-0.009289
5	2.751	22187.7	-0.256098	-0.008936	-0.002371	0.966606	-0.000747

9.) References

[1] J. Higuchi, S. Kuriyama, A. Eizawa, K. Arashiba, K. Nakajima, Y. Nishibayashi, Preparation and reactivity of iron complexes bearing anionic carbazole-based PNP-type pincer ligands toward catalytic nitrogen fixation, *Dalton Trans.* **2018**, *47*, 1117–1121.

[2] C. W. Stillwell, W. K. Robinson, Sodium-Lead Alloys the Structure of the Compound known as Na4Pb, *J. Am. Chem. Soc.* **1933**, *55*, 127–129.

[3] C. Lichtenberg, L. Viciu, M. Vogt, R. E. Rodriguez-Lugo, M. Adelhardt, J. Sutter, M. M. Khusniyarov, K. Meyer, B. de Bruin, E. Bill, H. Grutzmacher, Low-valent iron: an Fe(I) ate compound as a building block for a linear trinuclear Fe cluster, *Chem. Commun.* **2015**, *51*, 13890–13893.

[4] X. Tao, C. G. Daniliuc, O. Janka, R. Pottgen, R. Knitsch, M. R. Hansen, H. Eckert, M. Lubbesmeyer, A. Studer, G. Kehr, G. Erker, Reduction of Dioxygen by Radical/B(p-C(6) F(4) X)(3) Pairs to Give Isolable Bis(borane)superoxide Compounds, *Angew. Chem. Int. Ed. Engl.* **2017**, *56*, 16641–16644.

[5] E. J. Ko, G. P. Savage, C. M. Williams, J. Tsanaktsidis, Reducing the Cost, Smell and Toxicity of the Barton Reductive Decarboxylation: Chloroform as the Hydrogen Atom Source, *Org. Lett.* **2011**, *13*, 1944–1947.

[6] T. N. Gieshoff, U. Chakraborty, M. Villa, A. Jacobi von Wangelin, Alkene Hydrogenations by Soluble Iron Nanocluster Catalysts, *Angew. Chem. Int. Ed. Engl.* **2017**, *56*, 3585–3589.

[7] C. Lichtenberg, T. P. Spaniol, J. Okuda, Reactivity of Tris(allyl)aluminum toward Pyridine: Coordination versus Carbometalation, *Organometallics* **2011**, *30*, 4409–4417.

[8] T. Yamazaki, M. Nabeshima, T. Saito, T. Yamaji, K. Hayamizu, M. Yanagisawa, O. Yamamoto, National Institute of Advanced Industrial Science and Technology (AIST).

[9] K. Kabsch, in International Tables for Crystallography (Eds. M. G. Rossmann, E. Arnold), Vol. F, Ch. 11.3, Kluwer Academic Publishers, Dordrecht, The Netherlands, **2001**.

[10] SAINT, Bruker AXS GmbH, Karlsruhe, Germany 1997-2013 and SAINT V8.40A, Bruker AXS Inc., Madison, Wisconsin, USA, **2018**.

[11] CrysAlisPro, Agilent Technologies UK Ltd., Oxford, UK 2011-2014 and Rigaku Oxford Diffraction, Rigaku Polska Sp.zo.o., Wrocław, Poland, **2015–2020**.

[12] R. H. Blessing, An empirical correction for absorption anisotropy, *Acta Cryst.* **1995**, *A51*, 33–38.

[13] G. M. Sheldrick, SADABS, Bruker AXS GmbH, Karlsruhe, Germany, **2004–2014**.

[14] SCALE3, ABSPACK, CrysAlisPro, Agilent Technologies UK Ltd., Oxford, UK 2011-2014 and Rigaku Oxford Diffraction, Rigaku Polska Sp.z o.o., Wrocław, Poland **2015–2020**.

[15] W. R. Busing, H. A. Levy, High-speed computation of the absorption correction for single-crystal diffraction measurements, *Acta Cryst.* **1957**, *10*, 180–182.

[16] O. V. Dolomanov, L. J. Bourhis, R. J. Gildea, J. A. K. Howard, H. Puschmann, OLEX2: a complete structure solution, refinement and analysis program, *J. Appl. Cryst.* **2009**, *42*, 339–341.

[17] G. M. Sheldrick, SHELXT – Integrated space-group and crystal-structure determination, *Acta Cryst.* **2015**, *A71*, 3–8.

[18] G. M. Sheldrick, Crystal structure refinement with SHELXL *Acta Cryst.* **2015**, *C71*, 3–8.

[19] C. B. Hübschle, G. M. Sheldrick, B. Dittrich, ShelXle: a Qt graphical user interface for SHELXL, *J. Appl. Crystallogr.* **2011**, *44*, 1281–1284.

[20] J. S. Rollett, in Crystallographic Computing (Eds.: F. R. Ahmed, S. R. Hall, H. C. P.), Munksgaard, Copenhagen, Denmark, **1970**.

[21] D. Watkin, in Crystallographic Computing 4 (Eds.: N. W. Isaaks, M. R. Taylor), IUCr and Oxford University Press, Oxford, UK, **1988**.

[22] P. Müller, R. Herbst-Irmer, A. L. Spek, T. R. Schneider, M. R. Sawaya, in Crystal Structure Refinement (Ed.:P. Müller), Ch. 5, Oxford University Press, Oxford, UK, **2006**.

[23] D. Watkin, Structure refinement: some background theory and practical strategies, *J. Appl. Cryst.* **2008**, *41*, 491–522.

[24] A. Thorn, B. Dittrich, G. M. Sheldrick, Enhanced rigid-bond restraints, *Acta Cryst.* **2012**, *A68*, 448–451.

[25] D. Kratzert, FinalCif, V132, https://dkratzert.de/finalcif.html.

[26] S. Parsons, H. Flack, T. Wagner, Use of intensity quotients and differences in absolute structure refinement, *Acta Crystallogr.* **2013**, *B69*, 249–259.

[27] F. Neese, The ORCA program system, *WIREs Comput. Mol. Sci.* **2011**, *2*, 73–78.

[28] F. Neese, F. Wennmohs, U. Becker, C. Riplinger, The ORCA quantum chemistry program package, *J. Chem. Phys.* **2020**, *152*, 224108.

[29] F. Neese, Software Update: The ORCA Program System—Version 6.0, *WIREs Comput. Mol. Sci.* **2025**, *15*.

[30] J. W. Furness, A. D. Kaplan, J. Ning, J. P. Perdew, J. Sun, Accurate and Numerically Efficient r(2)SCAN Meta-Generalized Gradient Approximation, *J. Phys. Chem. Lett.* **2020**, *11*, 8208–8215.

[31] S. Ehlert, U. Huniar, J. Ning, J. W. Furness, J. Sun, A. D. Kaplan, J. P. Perdew, J. G. Brandenburg, r(2)SCAN-D4: Dispersion corrected meta-generalized gradient approximation for general chemical applications, *J. Chem. Phys.* **2021**, *154*, 061101.

[32] S. Grimme, A. Hansen, S. Ehlert, J.-M. Mewes, r2SCAN-3c: A “Swiss army knife” composite electronic-structure method, *J. Chem. Phys.* **2021**, *154*.

[33] F. Weigend, R. Ahlrichs, Balanced basis sets of split valence, triple zeta valence and quadruple zeta valence quality for H to Rn: Design and assessment of accuracy, *Phys. Chem. Chem. Phys.* **2005**, *7*, 3297–3305.

[34] F. Weigend, Accurate Coulomb-fitting basis sets for H to Rn, *Phys. Chem. Chem. Phys.* **2006**, *8*, 1057–1065.

[35] F. Neese, An improvement of the resolution of the identity approximation for the formation of the Coulomb matrix, *J. Comput. Chem.* **2003**, *24*, 1740–1747.

[36] L. Goerigk, S. Grimme, Efficient and Accurate Double-Hybrid-Meta-GGA Density Functionals—Evaluation with the Extended GMTKN30 Database for General Main Group Thermochemistry, Kinetics, and Noncovalent Interactions, *J. Chem. Theory Comput* **2011**, *7*, 291–309.

[37] S. Grimme, Accurate description of van der Waals complexes by density functional theory including empirical corrections, *J .Comput. Chem.* **2004**, *25*, 1463–1473.

[38] S. Grimme, J. Antony, S. Ehrlich, H. Krieg, A consistent and accurate ab initio parametrization of density functional dispersion correction (DFT-D) for the 94 elements H-Pu, *J. Chem. Phys.* **2010**, *132*, 154104.

[39] A. D. Becke, E. R. Johnson, A density-functional model of the dispersion interaction, *J. Chem. Phys.* **2005**, *123*, 154101.

[40] E. R. Johnson, A. D. Becke, A post-Hartree-Fock model of intermolecular interactions, *J. Chem. Phys.* **2005**, *123*, 24101.

[41] A. V. Marenich, C. J. Cramer, D. G. Truhlar, Universal Solvation Model Based on Solute Electron Density and on a Continuum Model of the Solvent Defined by the Bulk Dielectric Constant and Atomic Surface Tensions, *J. Phys. Chem. B* **2009**, *113*, 6378–6396.

[42] R. F. W. Bader, Atoms in molecules, *Acc. Chem. Res.* **1985**, *18*, 9–15.
[43] R. F. W. Bader, A quantum theory of molecular structure and its applications, *Chem. Rev.* **1991**, *91*, 893–928.
[44] C. Lee, W. Yang, R. G. Parr, Development of the Colle-Salvetti correlation-energy formula into a functional of the electron density, *Phys. Rev. B Condens. Matter.* **1988**, *37*, 785–789.
[45] A. D. Becke, Density-functional thermochemistry. III. The role of exact exchange, *J. Chem. Phys.* **1993**, *98*, 5648–5652.
[46] S. Grimme, S. Ehrlich, L. Goerigk, Effect of the damping function in dispersion corrected density functional theory, *J. Comput. Chem.* **2011**, *32*, 1456–1465.
[47] E. van Lenthe, E. J. Baerends, J. G. Snijders, Relativistic regular two-component Hamiltonians, *J. Chem. Phys.* **1993**, *99*, 4597–4610.
[48] E. van Lenthe, E. J. Baerends, J. G. Snijders, Relativistic total energy using regular approximations, *J. Chem. Phys.* **1994**, *101*, 9783–9792.
[49] C. van Wüllen, Molecular density functional calculations in the regular relativistic approximation: Method, application to coinage metal diatomics, hydrides, fluorides and chlorides, and comparison with first-order relativistic calculations, *J. Chem. Phys.* **1998**, *109*, 392–399.
[50] J. D. Rolfes, F. Neese, D. A. Pantazis, All-electron scalar relativistic basis sets for the elements Rb-Xe, *J. Comput. Chem.* **2020**, *41*, 1842–1849.
[51] F. Neese, Efficient and accurate approximations to the molecular spin-orbit coupling operator and their use in molecular g-tensor calculations, *.J Chem. Phys.* **2005**, *122*, 34107.
[52] B. A. Heß, C. M. Marian, U. Wahlgren, O. Gropen, A mean-field spin-orbit method applicable to correlated wavefunctions, *Chem. Phys. Lett.* **1996**, *251*, 365–371.
[53] T. Lu, F. Chen, Multiwfn: A multifunctional wavefunction analyzer, *J. Comp. Chem.* **2011**, *33*, 580–592.
[54] B. O. Roos, P. R. Taylor, P. E. M. Siegbahn, A complete active space SCF method (CASSCF) using a density matrix formulated super-CI approach, *Chem. Phys.* **1980**, *48*, 157–173.
[55] P. E. M. Siegbahn, A. Heiberg, B. O. Roos, B. Levy, A Comparison of the Super-CI and the Newton-Raphson Scheme in the Complete Active Space SCF Method, *Phys. Scr.* **1980**, *21*, 323–327.
[56] G. L. Stoychev, A. A. Auer, F. Neese, Automatic Generation of Auxiliary Basis Sets, *J. Chem. Theory Comput.* **2017**, *13*, 554–562.
[57] C. Angeli, R. Cimiraglia, S. Evangelisti, T. Leininger, J.-P. Malrieu, Introduction of n-electron valence states for multireference perturbation theory, *J. Chem. Phys.* **2001**, *114*, 10252–10264.
[58] C. Angeli, R. Cimiraglia, J.-P. Malrieu, N-electron valence state perturbation theory: a fast implementation of the strongly contracted variant, *Chem. Phys. Lett.* **2001**, *350*, 297–305.
[59] C. Angeli, R. Cimiraglia, J.-P. Malrieu, n-electron valence state perturbation theory: A spinless formulation and an efficient implementation of the strongly contracted and of the partially contracted variants, *J. Chem. Phys.* **2002**, *117*, 9138–9153.
[60] L. Lang, M. Atanasov, F. Neese, Improvement of Ab Initio Ligand Field Theory by Means of Multistate Perturbation Theory, *J. Phys. Chem. A* **2020**, *124*, 1025–1037.
[61] Chemcraft, graphical software for visualization of quantum chemistry computations (www.chemcraftprog.com).
